# Effect of Micro-Osteoperforations on Orthodontic Tooth Movement: A Systematic Review and Meta-Analysis

**DOI:** 10.1016/j.identj.2026.109536

**Published:** 2026-04-28

**Authors:** Ali Heidari, Sepideh Mojiri, Masood Azarbayjani, Nima Khamisi

**Affiliations:** School of Dentistry, Islamic Azad University (Khorasgan Branch), Isfahan, Iran

**Keywords:** Accelerated orthodontics, Anterior crowding, Treatment duration, Bone remodelling, Alveolar bone, Orthodontic appliances, Randomized controlled trials

## Abstract

**Background:**

While micro-osteoperforations (MOPs) have been proposed as a potential solution for the slow pace of orthodontic treatment, their effectiveness is widely debated. This meta-analysis was designed to assess whether MOPs are a clinically effective and safe tool, and to explore how they might be best applied.

**Methods:**

Based on the protocol registered prospectively in PROSPERO (CRD42025633088), a systematic search was conducted in PubMed, Embase, Scopus, Cochrane CENTRAL and key grey literature. Randomized controlled trials were synthesized using random-effects meta-analysis; extensive subgroup and meta-regression analyses were conducted to explore heterogeneity. The certainty of evidences was rated with the GRADE framework.

**Results:**

Thirty-nine trials encompassing 1025 participants suggested a substantial acceleratory effect (SMD = 1.66; 95% CI: 1.27-2.05). Meta-regression suggested that the standardized effect may decrease with longer follow-up (β = −0.168 SMD units per week) and protocols that repeated MOPs approximately every 4 weeks were associated with ∼0.54 mm of cumulative movement (study-level association). In metric-specific analyses, MOPs appeared to improve anterior alignment measured by Little’s Irregularity Index by 1.24 mm during the first month with no statistical heterogeneity (I² = 0%). No clear evidence of increased root resorption or clinically important periodontal deterioration was observed. Pain was minimal overall, though it was associated with the number of perforations performed.

**Conclusion:**

Moderate-certainty of evidence suggests that a protocol-driven approach-specifically, repeating a trio of shallow buccal perforations every 4 weeks is associated with accelerated tooth movement. However, substantial heterogeneity warrants cautious interpretation of the precise magnitude of this effect.

## Background

Orthodontic treatment realigns malposed teeth by stimulating alveolar bone remodelling around the periodontal ligament, a biologic process that is inherently slow.^1^ A meta-analysis of 11 cohort studies estimated the average duration of comprehensive fixed-appliance therapy at 25 months, and many courses exceed 2 years.^2^ Prolonged appliance wear hinders plaque control, heightens enamel demineralization and caries risk,^3^ causes gingival enlargement,^4^ and raises the likelihood of external apical root resorption.^5^ Lengthy treatment can also demotivate patients and erode satisfaction.^6^^,^^7^ These sequelae underscore the clinical and patient-driven imperative to safely accelerate OTM.

Several adjuncts have been explored. Conventional corticotomy and periodontally accelerated osteogenic orthodontics reliably induce the regional acceleratory phenomenon but require full-thickness flaps and entail surgical morbidity.^8^ Piezocision avoids a flap yet shows inconsistent, low-certainty gains.^9^ Physical modalities, such as low-level laser therapy, deliver only small and protocol-sensitive effects.^10^^,^^11^ Pharmacologic mediators-including prostaglandins, RANKL, vitamin D3 and parathyroid hormone-face safety or delivery barriers.^12^

MOPs are a minimally invasive, flapless technique proposed to accelerate OTM by creating multiple perforations through the gingiva into cortical bone, thereby amplifying the inflammatory response to orthodontic force.^13^ Localized micro-injury from shallow cortical perforations is thought to provoke a local release cytokine that recruits osteoclasts, induces transient osteopenia and thereby hastens tooth movement.^14^

Early split-mouth trials recorded 2-fold faster canine retraction during the first month after a single MOP session, with negligible adverse effects.^15^ Later randomized controlled trials corroborated this early boost but noted that the advantage often plateaus after 4 to 8 weeks unless the procedure is repeated.^16^^,^^17^ Reported efficacy varies with perforation number, depth, repetition interval and the type of movement studied.^18^ Safety signals remain favourable, although 1 trial observed a small, non-significant trend toward increased root resorption adjacent to perforation sites.^19^ Thus, although MOPs are simple and well tolerated, their true clinical value is still debated.

Systematic reviews published to date have reached divergent conclusions-from large to negligible effects-largely because of methodological heterogeneity and limited power.^20^^,^^21^ Hence clinicians remain uncertain whether MOPs offer a clinically meaningful advantage.

The present systematic review and meta-analysis aims to resolve this uncertainty by synthesizing all available randomized controlled trials evaluating the effect of micro-osteoperforations on the rate of orthodontic tooth movement. By focusing exclusively on RCTs, adhering to PRISMA guidelines, and performing dose and technique-oriented subgroup analyses, we seek to deliver conclusive, practice-oriented evidence on the value of MOP.

## Methods

This review followed a protocol registered prospectively in PROSPERO (CRD42025633088) and is reported in accordance with the Preferred Reporting Items for Systematic Reviews and Meta-Analyses (PRISMA) 2020 statement.

### Inclusion and exclusion criteria

Parallel-arm or split-mouth randomized controlled trials in human orthodontic patients that compared any MOP protocol-regardless of instrument, perforation depth, number, site or repetition-with identical fixed-appliance mechanics delivered without MOPs were eligible. Trials employing additional acceleration methods, non-randomized designs or patients with systemic bone-metabolism disorders were excluded.

### Outcome definitions

The specific primary outcome was OTM, defined as the change in mean or standardized mean position between intervention and control groups. Where authors reported tooth movement directly, those values were used. When only ‘space remaining’ was given, movement was calculated as the reduction in space and the standard deviation of change was imputed assuming a within-subject correlation of 0.5. Movement was analysed over 2 intervals: first, from baseline to 4 weeks, which was the most frequent time point for measurement of OTM (capturing a single MOP session; only trials without repeat MOPs before week 4 were included), and second, from baseline to each study’s last reported follow-up (including trials with repeated MOPs). If a study reported a rate in mm month⁻¹ for the first month, that value was used in the 4-week synthesis.

In addition to the secondary outcomes that were discussed in the protocol, any additional outcome measured in 3 or more trials was also pooled. These outcomes were pain intensity (visual-analogue scale immediately post-procedure, and change from baseline to 24 h and 7 days), root resorption, gingival index, probing pocket depth, and anchorage loss.

### Search strategy

PubMed, Embase, Scopus and Cochrane CENTRAL, together with ClinicalTrials.gov, Google Scholar and ProQuest, were searched from inception to 14 December 2024 with no language restriction; full search strings are provided in Appendix Table 1. Reference lists of eligible papers and relevant reviews were hand-searched, and corresponding authors were contacted for missing data.

### Data collection, extraction, and management

Search results were imported into EndNote X9 and de-duplicated. First, selecting based on titles and abstracts was conducted by 2 reviewers ([anonymised] and [anonymised]). Following this, the full texts of potentially eligible records were screened independently by 2 different reviewers ([anonymised]). Any disagreements were resolved by a third reviewer ([anonymised]).

Data extraction was done by 2 reviewers ([anonymised]) to extract data on study setting, participant demographics, malocclusion class, appliance details, applied force, MOP protocol, follow-up times and etc**.** *Any disagreement was resolved by* [anonymised]**.**

### Risk of bias assessment (quality assessment)

Two reviewers ([anonymised]) assessed each trial with the Cochrane Risk of Bias 1 tool independently, judging sequence generation, allocation concealment, blinding of participants and personnel, blinding of outcome assessment, incomplete outcome data, selective reporting, and other bias. Because micro-osteoperforations are procedural, operator blinding is not feasible and participant blinding may be compromised even with sham procedures. We therefore documented this limitation explicitly and incorporated it outcome-specifically: for patient-reported outcomes (eg, pain), lack of participant/personnel blinding was considered a meaningful source of bias; for objective outcomes (tooth movement and periodontal measures assessed on models/CBCT), we did not automatically downgrade solely for lack of operator/participant blinding but relied on allocation concealment and blinded outcome assessment as key safeguards. Any disagreements were resolved by a third reviewer ([anonymised]).

### Data synthesis and statistical analysis

For each comparison, change of MD was calculated in the natural units of each outcome. The change SMD was also computed using Hedges g (due to the small number of participants in some groups) to allow unit-free interpretation of effect strength. Both change of MD and SMD were pooled with a random-effects model (DerSimonian-Laird), applying the Knapp-Hartung adjustment when at least ten trials contributed to a given synthesis (specifically for secondary outcomes). All SMD were interpreted using Cohen’s thresholds, where values of 0.2, 0.5, and 0.8 generally indicate small, medium, and large effect sizes, respectively.

We aim to address multiplicity at 2 levels. First, to minimize within-study effect-size multiplicity, we followed a reductionist strategy as described in methodological guidance by López-López and colleagues,^22^ selecting a single eligible comparison per study per synthesis to help ensure that no more than 1 pairwise comparison from any 1 dataset contributed to each meta-analysis (see Supplementary Methods – Multiplicity Control). Second, to help control the risk of false-positive findings across the prespecified moderator analyses for the primary 4-week outcome, we applied a Bonferroni correction to the family of planned subgroup and meta-regression tests and report Bonferroni-adjusted *P* values.

### Heterogeneity, subgroup analysis, and meta-regression

Heterogeneity was assessed with I². Subgroup analyses and meta-regression were performed mainly to attempt to explain the considerable inconsistency observed in the 4-week primary outcome. The covariates used for subgroup analysis and meta-regression were prespecified in the PROSPERO protocol and included clinically plausible procedural and methodological factors, such as perforation depth, perforation number, perforation site, arch, study design, and measurement method. In accordance with the Cochrane Handbook (Higgins et al), meta-regression was conducted only when at least 10 studies were available for a specific covariate. To enhance interpretability and transparency, subgroup categories supported by at least 3 studies are reported in the main manuscript, while categories supported by 1 or 2 studies are reported in the Appendix and clearly identified as exploratory (See Supplementary Methods – Heterogeneity & Moderator Analysis, online Appendix).

### Sensitivity analyses

Robustness was tested in 3 ways: first, by conducting a leave-one-out analysis; second, by recalculating the pooled results after excluding studies judged as ‘high risk’ in allocation concealment. This restriction did not appear to materially alter the primary outcome (SMD remained >1.5), suggesting that the findings may be robust to potential selection bias; and third, by comparing outcomes across key subgroups: parallel-arm versus split-mouth designs, studies that directly reported change means and standard deviations versus those where SDs were calculated (assuming r=0.5), and studies in which change scores were averaged across some calculation (for example, maxilla and mandible, different tooth regions such as incisal and mesial, or arms with varying numbers of perforations) versus those providing raw change data. (All of these steps were undertaken to help avoid multiplicity by aiming to ensure no more than 2 arms-or a single pairwise comparison-from any 1 study were included in the meta-analysis).

### Small-study effects and cumulative evidence

Publication bias was examined with funnel plots, Egger’s test, and the trim-and-fill method. Also, a cumulative forest plot ordered by publication year illustrates the evolution of the pooled effect.

### Certainty of evidence (GRADE)

Each outcome started at high certainty (RCT evidence) and was then downgraded for risk of bias, inconsistency, imprecision, indirectness, or publication bias; conversely, it could be upgraded for a large effect or a clear dose-response gradient (See Supplementary Methods – GRADE Criteria and Procedures, online Appendix).

### Software and data sharing

All statistical analyses were conducted in Stata 17. Data files will be shared as online supplementary material upon publication.

## Results

### Study selection

**Fig. 1:**
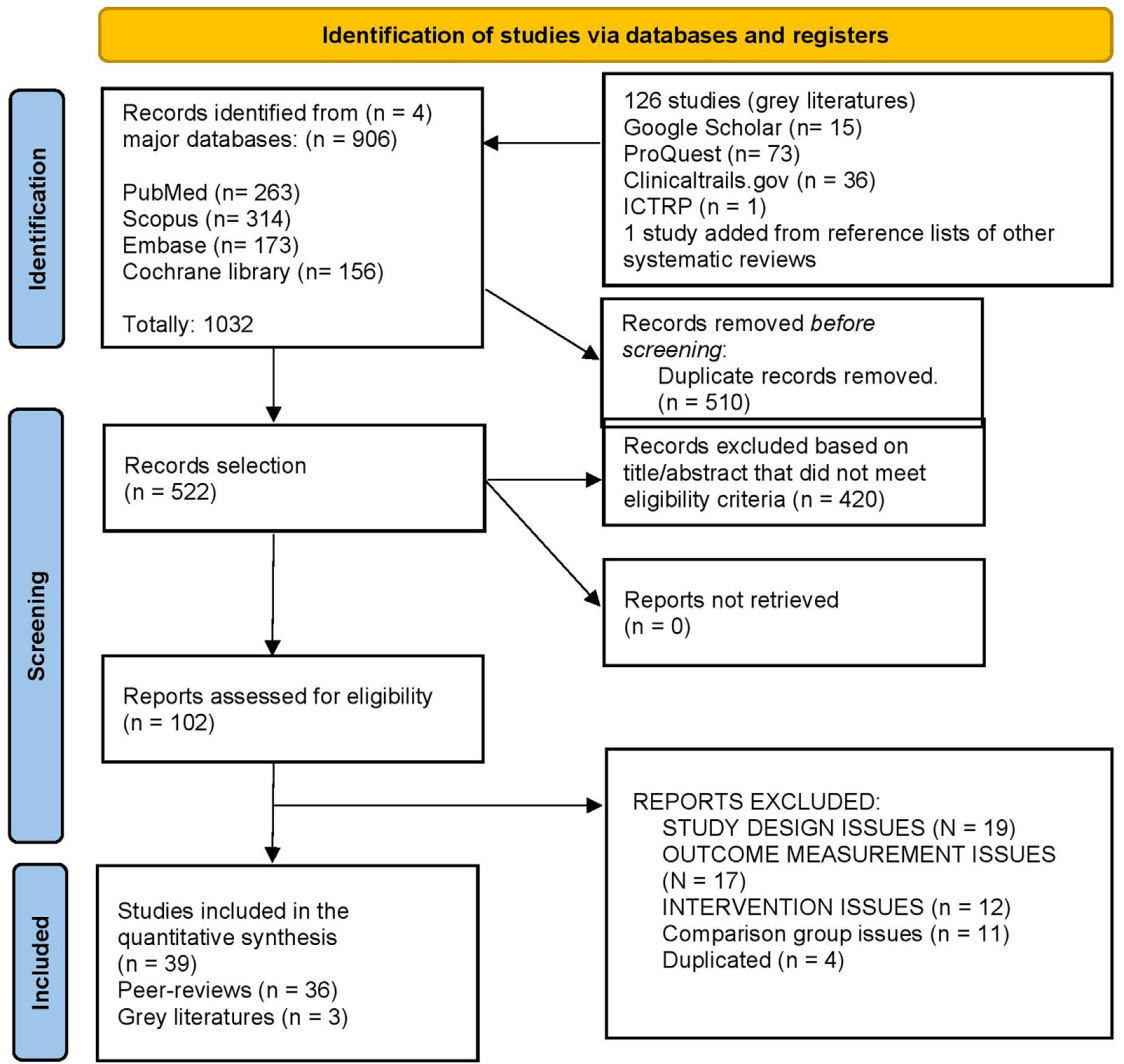
PRISMA flowchart.

Searches yielded 1 032 records, and 126 additional records were found through grey-literature sources. After de-duplication, 522 titles and abstracts were screened and 13 emails that were sent to corresponding authors to clarify missing data (only 1 author replied,^23^ which allowed their article to be included) 98 full texts were assessed and 39 randomized controlled trials (1025 participants) met the criteria (Figure 1).

### Characteristics of the included trials

Across the 39 trials published between 2013 and 2024, roughly half followed a split-mouth and the remainder a parallel-arm design, with sample sizes ranging from 8 to 60. Participant ages were typically 16 to 28. Most studies originated from South-West Asia or North Africa. Operators generally used standard orthodontic miniscrews rather than the disposable Propel perforator; protocols were diverse, producing anywhere from 2 to 21 perforations per session, with diameters of 0.9 to 2 mm and depths of 1 to 8 mm. Twenty-nine trials performed a single perforation session, whereas ten repeated the procedure at 2- to 8-week intervals; 32 trials targeted only the buccal cortex, while 7 perforated both buccal and palatal/lingual cortices. All investigations paired MOP with conventional fixed appliances, most often applying 100 to 200 g of force, although 1 molar-distalization study applied 500 g. Follow-up periods stretched from 4 weeks to 12 months, with fewer than half of the measurements falling between 8 and 12 weeks. Nearly every study reported tooth movement in millimetres. Full study characteristics appear in Table 1 and Appendix Table 2.

**Table 1:** Characteristics of the included studies.

Study	Country	Study design	Duration	Participants	Age	Sex	Details of MOP	tooth movement, jaw	Method of OTM measurement, Device
**Golshah et al**^40^	Iran	split mouth	5 months	25 (losses to follow up: 0)	16-25	11F, 14M	Five MOPs (3 buccal, 2 palatal) were created using G2 miniscrews (8 × 1.6 mm) with a handpiece at 15 N·cm torque, placed 3 mm distal to the canine and 6-7 mm from the gingival margin, with perforations 3 mm apart, 3-4 mm deep, 1.6 mm wide, made perpendicular to the distal of the canine tooth, and not repeated at each interval.	Canine retraction, Maxilla	Method 1: from the centre of the canine’s distal prominence on superimposed models to its new position to quantify the distal movement of the canine tooth over time. Method 2: from the canine cusp tip to a perpendicular line drawn from the miniscrews relative to the occlusal plane on initial and subsequent models to assess the positional change of the canine cusp tip as an alternative measure of OTM. Device: Digital Scanning & Software (model superimposition)
**Thomas et al**^57^	India	split mouth	90 days	33 (losses to follow up: 3)	19-25 (22.1 ± 2.19)	24F, 9M	Six MOPs (all buccal) were placed 3 mm apart vertically on the mesial and distal aspects of the canine root (each site 3 mops), starting 6 mm apical from the alveolar crest, using a lance drill (AGDLC; Osteem Implant Corporation, South Korea) attached to a physiodispenser handpiece (500 rpm speed, 50 Ncm torque; Equinox, Netherlands); perforations measured 4 mm in depth and 2 mm in width, placed 2 mm distal to the canine root on the distal side and in the inter-radicular bone between the canine and lateral incisor on the mesial side, and MOP was not repeated at each interval.	Canine retraction, Maxilla	Method 1 (D1): from the molar tube slot to the canine bracket slot to assess tooth movement over time (mean of observations used for analysis). Method 2 (D2): from the molar tube hook to the micro-implant. Canine angulation (CA): measured via CBCT as the angle between the canine’s long axis and the palatal plane. Inter-radicular bone width between the lateral incisor and canine was also measured preoperatively using CBCT. Device: Digital Caliper for D1 and D2; CBCT imaging for CA and bone width.
**Martina et al**^51^	India	parallel arm	90 days	40 (losses to follow up: 0) 3MOPs:10 2MOPs:10 Control:20	15-25	22F, 18M	In this study, MOPs were placed distal to the canines using FavAnchor miniscrews (1.6 mm diameter, 8 mm length) to create vertical perforations 3 mm deep and 1.6 mm wide, spaced 2 mm apart; Group-I A received 3 buccal MOPs and Group-II A received 2 buccal MOPs, with MOPs repeated every 30 days for 3 months.	Canine retraction, Maxilla	The linear distance moved by the canine was measured on study casts by drawing 2 perpendicular lines; from the mesial point of the third palatal rugae (reference point) and from the distal side of the canine to the mid-palatine suture. Device: Digital Caliper (Vernier)
**Gulduren et al**^42^	Northern Cyprus	parallel arm	12 weeks	20 (losses to follow up: 2) MOP:10 (losses to follow up: 1) Control:10 (losses to follow up: 1)	16-24 MOP: 16.5-23.8 (21.8) Control: 16.5-23.7 (17.7)	7F, 11M	Six MOPs (all buccal) were performed in the maxillary molar alveolar regions, with 2 perforations at each of 3 sites, between the second premolars and first molars, between the first and second molars, and distal to the second molars, using a 1.4 mm drill; perforations were 5-6 mm deep and 1.4 mm wide to penetrate the cortical plate and reach the spongious bone, the distance between perforations was N/A, and the procedure was repeated every 3 weeks for a total of 3 sessions.	Molar distalization, Maxilla	Digital scans were taken at baseline (T0) and subsequent intervals (T1, T2, T3, T4) using a CEREC Omnicam. The scans were analysed with Geomagic Studio 2014 software. 3D models were aligned along the Y-axis, and the distance between the midpoint of the first miniscrew and the distal margin of the maxillary first molar was measured and changes in this indicating tooth movement, verified from occlusal and lateral views. Device: Digital Scanning & Software (CEREC Omnicam + Geomagic Studio)
**Feizbakhsh et al**^39^	Iran	split mouth	28 days	20 (losses to follow up: 0)	18-33^28^	8F, 12M	Four MOPs were performed distal to the canines, 2 buccal in the maxilla and 2 buccal in the mandible, using a screwdriver device (Jeil Medical Corporation, Seoul, Korea) and bony screws measuring 1.6 mm in diameter and 3.0 mm in length; perforations were 3 mm deep, 1.6 mm wide, spaced 3 mm apart, located 5 mm from the alveolar crest, and the procedure was not repeated at each interval.	Canine retraction, Maxilla and Mandible	Models were digitized using a 3D scanner (3Shape Trios; 3Shape Dental Systems, Copenhagen, Denmark) and were analysed with Ortho Analyzer software (v.2016-1; 3Shape), where tooth movement between phases was measured. Distances between the canine and second premolar were assessed in 3 areas: the centre of the canine and premolar brackets, the canine cusp tip and premolar buccal cusp tip, and the shortest distance between the canine and premolar at the cervicogingival line. The average distance from these 3 areas was used for data analysis. (Note: distances were not reported separately by jaw.) Device: Digital Scanning & Software (3Shape Trios with Ortho Analyzer)
**Fattori et al**^38^	Brazil	parallel arm	N/A	24 (losses to follow up: 6) MOP: 12 (losses to follow up: 3) Control:12 (losses to follow up: 3)	MOP: (27.8 ± 6.3) Control: (20.4 ± 2.6)	11F, 7M	Three buccal MOPs were performed vertically in the space midway between the canine and second premolar, 6 mm deep and spaced 4 mm apart; the width of the perforations was not reported, and the site was 4 mm from the alveolar crest. The procedure was repeated every 28 days at each activation session for an average of 7 sessions.	Alignment, Maxilla and Mandible	Alginate impressions were taken at each activation session until space closure. A pre-retraction model (T0) was made 28 days post-archwire placement and digitized using XCAD 3D Scanner, True Image Belo Horizonte, Brazil, and Maestro 3D OrthoStudio, AGE Solutions, Pisa, Italy. Extraction space was measured linearly in 3D (Q3DC tool SlicerCMF) between the canine’s most distal point and second premolar’s most mesial point, with left-right means used for rate of tooth movement calculations. Device: Digital Scanning & Software (XCAD 3D Scanner, Maestro 3D OrthoStudio, Q3DC tool)
**Farag et al**^37^	Egypt	split mouth	>3 months	20 (preferred arm: 10) MOP:10 Control:10	15-25	N/A	Twelve MOPs (6 buccal and 6 palatal) were performed using mini-screws measuring 1.6 mm in diameter and 8 mm in length to achieve a perforation depth of 6 mm, accounting for an average gingival thickness of 2 mm; perforations were placed between the canine and lateral incisor roots and between the canine root and the socket of the extracted premolar on both buccal and palatal sides. The distance between perforations was N/A. MOPs were repeated every 2 weeks per session.	Canine retraction, Maxilla	The measurements were taken from the canine cusp tip to the mesiobuccal cusp tip of the maxillary first molar using digital intraoral caliper (IOS, China), which were taken immediately before the beginning of canine retraction and every 2 weeks along the following 3 months. Device: Digital Caliper (IOS, China)
**El Awady et al**^36^	Egypt	split mouth	6 months	12 (losses to follow up: 0)	13-19 (16.17 ± 2.29)	8F, 4M	Three vertical buccal MOPs were performed, using miniscrews (HUBIT, Korea) of 1.6 mm diameter and 8 mm length to create perforations 4 mm deep and 1.6 mm wide; the first insertion point was 6 mm from the free gingival margin, with subsequent points spaced 5 mm apart, positioned equidistantly between the canine and second premolar in the extraction space. MOP was not repeated at each interval.	Canine retraction, Maxilla	The measurement was based on assessing the bilateral distance between the distal contact points of the canines and the mesial contact points of the second premolars, using a digital caliper (Digimatic Caliper, Mitutoyo, China). The rate of canine retraction (mm/month) was calculated by dividing the total amount of retraction (in millimetres) by the total duration of retraction (in months).
**Bansal et al**^34^	India	parallel arm	15 + B (until alignment of mandibular anterior teeth) weeks B MOP: 7.40 ± 1.55 weeks B Control: 13.20 ± 1.52 weeks	30 (losses to follow up: 0)	14-19 (15.6 ± 1.476) MOP: (15.87 ± 1.727) Control: (15.33 ± 1.175)	16F, 14M MOP: 8F, 7M Control: 8F, 7M	Six buccal MOPs were performed on the labial aspect of the mandible at 3 sites, between the mandibular canine and lateral incisor on both sides and between the central incisors in the midline, using a 1.6 × 8 mm self-drilling orthodontic mini-implant (DENTAURUM GmbH & Co KG) and an MI screwdriver (Tomas, DENTAURUM GmbH & Co KG); at each site, 2 vertically aligned perforations were made, one 2 mm apical to the alveolar crest and the other 2 mm below it, with depths of 3-5 mm, a width of 1.6 mm, and a 2 mm distance between the perforations. MOP was not repeated at each interval.	Alignment, Mandible	Little’s Irregularity Index (LII) was measured at each visit using a digital caliper (Insize Digimatic 1108 150, 0.03 mm accuracy). Treatment completion required LII ≤1 mm with <0.5 mm change between consecutive visits. LII measures the horizontal linear displacement of anatomic contact points of each mandibular incisor from the adjacent anatomic point and sums the 5 displacement together, Which represents the degree of anterior irregularity. Device: Digital Caliper (Insize Digimatic 1108 150)
**Al-Hayek et al**^28^	Egypt	parallel arm	6 months	26 (losses to follow up: 5) MOP:13 (losses to follow up: 2) Control:13 (losses to follow up: 3)	18-25	21F, 0M	Twenty-one buccal MOPs were performed across 7 areas (left and right sides combined), with 3 linear MOPs at each site: mesial to the mandibular central incisors, distal to the mandibular central incisors, distal to the mandibular lateral incisors, and distal to the mandibular canines. Depth, width, and distance between perforations were N/A. MOP was not repeated at each interval.	Alignment, Mandible	Little’s Irregularity Index (LII) was measured on digital casts at each visit until LII = 0 mm. Additionally, an ‘alignment improvement percentage’ was presented at each time point. LII measures the horizontal linear displacement of anatomic contact points of each mandibular incisor from the adjacent anatomic point and sums the 5 displacement together, Which represents the degree of anterior irregularity. Device: Digital Scanning & Software (digital orthodontic cast analysis)
**Attri et al**^32^	India	parallel arm	Until space closure was completed	60 (losses to follow up: 0)	13-20 MOP: (17.5 ± 2.52) Control: (18.16 ± 1.48)	33F, 27M MOP: 18F, 12M Control: 15F, 15M	Three buccal MOPs were performed in the extraction space at equal distances from the canine and the second premolar in the cortical bone using the ‘Propel’ device (Propel Orthodontics, Ossining, New York); the perforations were 2-3 mm deep and 1.5 mm wide. The distance between perforations was not reported. MOP was repeated every 28 days until space closure was completed.	Canine retraction, Maxilla and Mandible	The extraction space was measured digitally by constructing a mid-palatine reference line and perpendicular measurement lines from the canine's distal surface to the second premolar's mesial surface. All measurements were performed on digital models obtained by 3D scanning plaster casts using a White Light Scanner (COMET5, Steinbichler Optotechnik, Germany) with reverse modelling software. Device: Digital Scanning & Software (White Light Scanner COMET5 with 3D reverse modelling)
**Babanouri et al**^33^	Iran	split mouth	84 days	28 (losses to follow up: 3) MOP1 (3 MOPs):14 (losses to follow up: 2) MOP2 (6 MOPs):14 (loss to follow up: 1) Control:25	16.3-35.2 MOP1: (26.08 ± 9.15) MOP2: (25.31 ± 9.03)	14F, 11M MOP1: 7F, 5M MOP2: 7F, 6M	In the MOP1 group, 3 buccal MOPs were created, and in the MOP2 group, 6 MOPs (3 buccal and 3 palatal) were created between the distal of the canine and the mesial of the second premolar at the extraction site in the vertical direction, spaced 3 mm apart. The first MOP was located 5 mm from the free gingival margin. Perforations were made using 1.2 mm diameter orthodontic mini-screws (Dual Top Anchor System; Jeil Medical Corporation, Seoul, South Korea) inserted 1 mm deep into the cortical bone under local anaesthesia. The perforation width was 1.2 mm. MOP was not repeated at each interval.	Canine retraction,Maxilla	Measurements were taken on plaster models using a digital caliper with 0.01 mm accuracy. Alginate impressions were made at baseline (T0: before retraction) and at 3 subsequent intervals (T1, T2, T3), totalling 84 days. Vertical reference lines were drawn on the palatal surfaces of the canine and lateral incisor, and distances were measured at the incisal, middle, and cervical thirds of the crowns at each time point. Device: Digital Caliper (on plaster models, 0.01 mm accuracy)
**Mahmoudi.,2016**^50^	United States of America	split mouth	3 months	10 (losses to follow up: 0)	18-37 (25.5)	8F, 2M	Three manual buccal MOPs were performed distal to the maxillary canines using the PROPEL® System (Propel Orthodontics, the Excellerator), creating perforations approximately 5 mm deep and 0.25 mm in diameter. The distance between perforations was N/A. MOP was not repeated at each interval.	Canine retraction,Maxilla	Cephalometric measurements were obtained from initial and final CBCTs using Anatomage Invivo5 software, including TAD-canine distal curvature (TAD-U3D) and second premolar mesial curvature-canine distal curvature (U5M-U3D) distances, with the TAD as a stable reference point. Digital models (T0, T2) were created using OrthoCAD 3.5.0.38, with bilateral measurements of^1^ canine-premolar cusp tips (U3-U5),^2^ extraction space width (U5M-U3D),^3^ lateral incisor midpoint-canine cusp tip distance (U2-U3), and^4^ first molar mesiopalatal-canine cusp tips distance (U6-U3). All analyses were performed in OrthoCAD 3.5.0.38. Device: Digital Scanning & Software
**GOWDA S V et al**^41^	India	parallel arm	4 weeks	46 (losses to follow up: 0)	14-31 MOP: (19.8 ± 3.8) Control: (20.7 ± 3.7)	32F, 14M Mop: 15F, 8M Control: 17F, 6M	Three buccal MOPs were performed within the extraction space, equidistant from the canine and second premolar, using 1.5 × 8 mm miniscrews and a hand driver (SK Surgicals). The depth of the MOPs was 3 mm. The perforation width was 1.5 mm. The distance between perforations was N/A. MOP was not repeated at each interval.	Canine retraction,Maxilla	Vertical reference lines were marked on the palatal surfaces of canines and lateral incisors (from incisal edge midpoint to cervical line midpoint). Inter-tooth distances were measured at incisal, middle, and cervical crown levels using a digital caliper (0.01 mm accuracy) before and after retraction. Device: Digital Caliper (0.01 mm accuracy)
**RASHEED. V et al**^54^	India	split mouth	114.5 ± 9.5 days	10 (losses to follow up: 0)	16-30	5F, 5M	Three vertical buccal MOPs were performed distal to the canine using 1.2 × 8 mm mini implants, spaced 3 mm apart. The mini implants were inserted 6 mm (3-quarters of their length) and then removed to achieve the desired MOP depth of 6 mm. The perforation width was 1.2 mm. MOP was repeated after 2 months with the same pattern.	Canine retraction,Maxilla	Vertical lines were drawn along the long axes of the canine and lateral incisor on dental casts to measure the distance between these lines at the incisal edge. Measurements were taken at time points T0, 45 days, and at completion (∼114 days). All measurements were made using a digital Vernier caliper. Device: Digital Caliper (Vernier)
**Alfailany et al**^27^	Syria	parallel arm	Till the end of the canine retraction phase	51 (losses to follow up: 0)	18-27 (20.98 ± 1.95) TC: (21.23 ± 2.33) FCAPs (MOP): (21.09 ± 2.06) Control: (20.62 ± 1.48) *P*-value: .647	29F, 22M TC: 11F, 6M FCAPs (MOP): 8F, 9M Control: 10F, 7M *P*-value: .571	Eleven MOPs were performed using mini-implant drills (0.9 × 4 × 22 mm; Aarhus, American Orthodontics) on a contra-angle handpiece with irrigation at slow speed. The perforations included 6 buccal (5 distal and 1 mesial to the canine) and 5 palatal (distal to the canine). The perforation depth was 4 mm, and the width was 0.9 mm. Distobuccal perforations were spaced 1.5-2 mm apart. Sites of perforation were between the canine and second premolar (distobuccal), between the canine and lateral incisor 4 mm from the papilla (mesiobuccal), and between the canine and second premolar on the palatal side. MOP was not repeated at each interval.	Canine retraction,Maxilla	Dental casts were obtained at 5 time points (T0-T4). Measurements were performed manually using a digital caliper (REF 042-751-00, Dentaurum, Ispringen, Germany) to assess: 1)The anteroposterior movement of the canine by measuring the distance between the medial ending of the third palatal rugae and the tip of the canine cusp tip. 2)The anteroposterior movement of the first molar by measuring the distance between the medial ending of the third palatal rugae and the central fossa of the first molar. 3)Canine rotation by measuring the angle between the mid-palatal suture and the line through the mesial and distal edges of the canine. Device: Digital Caliper (REF 042‑751‑00, Dentaurum)
**Rashid et al**^55^	India	parallel arm	6 months (till closing of the extraction space from initial month of retraction)	20 (losses to follow up: 0)	18-30	N/A	Three buccal MOPs were performed gingival to the extraction site distal to the canines at the coronal, middle, and apical regions using S.K. Surgicals Microimplants (1.5 mm diameter, 8.0 mm length). Each perforation was 1.5 mm wide and 3 mm deep. The distance between perforations was N/A. MOP was not repeated at each interval.	Canine retraction, Maxilla	The canine-premolar distance was measured to 0.1 mm precision using a sliding caliper, and the retraction rate was calculated as distance divided by time. Device: Digital Caliper (sliding caliper with 0.1 mm accuracy)
**Aboalnaga et al**^23^	Egypt	split mouth	4 months	18 (losses to follow up: 0)	16-25 (20.5 ± 3.85)	18F, 0M	Three vertical buccal MOPs were performed at equal distances from the canine and second premolar in the extraction space using an L-shaped wire guide to mark the sites. TADs (Unitek™ TAD, 1.8 × 8 mm) were inserted perpendicularly into the alveolar bone until slight blanching of the surrounding soft tissue indicated full penetration. Each perforation was 8 mm deep and 1.8 mm wide. The distance between perforations was N/A. MOP was not repeated at each interval.	Canine retraction, Maxilla	Stone models (T0-T4) were scanned with a 3Shape R900 scanner and digitized. The T0 model was superimposed on pre-retraction CBCT using 3 occlusal points to establish a frontal reference plane (FP), while subsequent models (T1-T4) were aligned to T0 using third rugae points. Using 3Shape Analyzer, monthly canine retraction was measured as the sagittal distance from the canine's cusp tip/centre/root apex to FP, while molar anchorage loss was measured from the first molar's mesiobuccal cusp tip/centre/root apex to FP. Total 3D displacement was calculated from pre and post-retraction CBCTs using In vivo 5 (v5.3). Device: Digital Scanning & Software (3Shape R900 with 3Shape Analyzer plus CBCT superimposition via In vivo 5)
**Alikhani et al**^15^	United States of America	parallel arm	28 days	20 (losses to follow up: 0)	19.5-33.1 MOP: (26.8) Control: (24.7)	12F, 8M MOP: 5F, 5M Control: 7F, 3M	Three buccal MOPs were performed distal to the canines before retraction using a disposable MOP device from PROPEL Orthodontics (Ossining, NY). Each perforation was 2-3 mm deep and 1.5 mm wide. The distance between perforations was N/A. MOP was not repeated at each interval.	Canine retraction, Maxilla	Vertical lines were drawn on the palatal surfaces of the canine and lateral incisor on dental casts, extending from the middle of the incisal edge to the middle of the cervical line to divide the crowns into equal halves. Tooth movement was assessed by measuring the distance between these two vertical lines at 3 locations: incisal, middle, and cervical thirds of the crowns. All measurements were performed on casts using an electronic digital caliper (Orthopli Corp, Philadelphia, PA) with 0.01 mm accuracy. Device: Digital Caliper (electronic, Orthopli Corp, 0.01 mm accuracy)
**Alqadasi et al**^30^	China	split mouth	3 months	8 (losses to follow up: 0)	15-40	N/A	Three buccal MOPs were made in the middle of the extraction space using automated mini-implant instrumentation. The perforations were 5-7 mm deep and 1.5-2 mm wide. The distance between perforations was N/A. MOP was not repeated at each interval.	Canine retraction, Maxilla	Dental cast models were scanned using a 3Shape scanner. Measurements were conducted with Geomagic software. a midline was drawn, and perpendicular lines were extended from the buccal cusp tips of the canines and second premolars on both sides. The distance from the canine cusp tip to the perpendicular line drawn from the second premolar cusp tip was used to measure tooth movement. Device: Digital Scanning & Software (3Shape scanner with Geomagic)
**Kundi et al**^48^	Saudi Arabia	parallel arm	12 months Evaluation: 28days	28 (losses to follow up: 0) MOP:28 Control:28	20-40 MOP: (28.4 ± 4.2) Control: (26.4 ± 4.1)	16F, 12M	Three buccal MOPs were performed distal to the canines using a disposable MOP device (PROPEL Orthodontics, Ossining, NY) with a diameter of 1.5 mm. The perforation depth and distance between perforations were not reported. MOP was N/A at each interval.	Canine retraction, Maxilla	Measurements were performed directly on unsoaped plaster casts using an electronic digital Vernier caliper (Mitu toyo, Japan) with sharpened tips. The canine retraction was quantified by two measurements: Tip distance: between the canine cusp tip and lateral incisor's incisal edge midpoint, and Cervical distance: between the cervical midpoints on the height of contour at both teeth's cinguli. Device: Digital Caliper (electronic digital Vernier, Mitu toyo)
**Venkatachalapathy et al**^58^	India	split mouth	84 days	20 (losses to follow up: 0)	15-25	N/A	Five buccal MOPs were performed using a hand-held TAD (1.5 × 9 mm) loaded in an implant driver (SK SURGICALS, INDIA) with a rubber stopper to ensure each perforation was 3 mm deep and 1.5 mm wide; two perforations were placed distal to the canine at heights of 7 mm and 12 mm from the alveolar crest, and 3 were placed in the middle of the extraction socket at 5 mm, 10 mm, and 15 mm from the crest (5 mm apart), repeated every 28 days (at day 28 and day 56).	Canine retraction, Maxilla and Mandible	Vertical reference lines were drawn on the palatal canine surface from the cervical line midpoint. Canine-lateral incisor distances were measured at incisal, middle, and cervical crown thirds pre- and post-retraction using a digital caliper (0.01 mm accuracy). Device: Digital Caliper (electronic, 0.01 mm accuracy)
**RAGHAV et al**^17^	India	split mouth	16 weeks	30 (losses to follow up: 2)	17-30 (20.26 ± 2.13)	18F, 12M	Three buccal MOPs were performed distal to the canine root using a Lance Pilot Drill (Alpha-Bio Tec, Simplantology Alpha Bio Tec LTD), with each perforation measuring 5 mm in depth and 2 mm in width; MOP was not repeated at each interval.	Canine retraction, Maxilla and Mandible	Alginate impressions were taken at baseline (T0) and subsequent intervals (T1-T4) and poured in Type II dental stone to create study models. An acrylic palatal plug with 0.9 mm SS reference wires was fabricated on the T0 model. Canine retraction was measured by transferring this plug to subsequent models and recording the displacement from the canine long-axis midpoint to the reference wire using a digital caliper (0.01 mm accuracy), which was carefully aligned parallel to both. Force levels were monitored using a Dontrix gauge. Device: Digital Caliper (digital Vernier, 0.01 mm)
**Singh et al**^56^	India	split mouth	56 days	22 (losses to follow up: 2) MOP1 (3 MOPs):11 (loss to follow up: 1) MOP2 (6 MOPs):11 (loss to follow up: 1) Control:20	18-30	N/A	MOPs were performed using a TAD (1.5 × 8 mm) with a rubber stopper standardizing the perforation depth at 6 mm and width at 1.5 mm. In the MOP1 group, 3 buccal perforations were made, while in the MOP2 group, 3 buccal and 3 palatal perforations were created. All perforations were placed distal to the canine, within the extraction site, vertically aligned, spaced 3 mm apart, with the first perforation positioned 5 mm from the free gingival margin. MOP was not repeated at any interval.	Canine retraction, Mandible	On dental casts at 3 time points (T0, T1, T2) vertical reference lines were drawn on the palatal surfaces of canines and lateral incisors, from the incisal edge to the cervical line midpoints. Using a digital caliper (0.01 mm accuracy), inter-tooth distances were measured at 3 levels (incisal, middle, and cervical thirds). Device: Digital Caliper (electronic vernier, 0.01 mm)
**Abdelhameed et al**^24^	Egypt	split mouth	3 months	30 (preferred arm: 10 which has 1 loss to follow up at 2 intervals; T2 and T5; 4 and 10 weeks respectively) MOP: 10 (loss to follow up only at T2 and T5: 1) Control: 10 (loss to follow up only at T2 and T5: 1)	15-25	N/A	MOPs were performed using mini-screws (1.6 × 8 mm), with an effective perforation depth of 6 mm in the alveolar bone, accounting for a gingival thickness of 2 mm. A total of 12 perforations were made per intervention session, 6 buccal and 6 palatal, placed between the canine and lateral incisor roots, as well as between the canine root and the socket of the extracted first premolar, on both buccal and palatal sides. MOP was repeated every two weeks, totalling 6 sessions over 3 months.	Canine retraction, Maxilla	Intraoral measurements were taken using a digital caliper to record the distance between the canine cusp tip and first molar mesiobuccal cusp tip. Measurements were performed immediately before retraction (baseline) and biweekly for 3 months. Device: Digital Caliper (direct intra‑oral measurement)
**Ahsan et al**^25^	Pakistan	split mouth	6 months	30 (losses to follow up: 0)	18-30 (21.7 ± 2.98)	13F, 17M	MOPs were performed using a TAD screw (1.6 mm diameter) to create 3 perforations on the buccal side of the right distal canine. Each perforation measured 3 mm in depth and 1.6 mm in width, targeting the buccal cortex. MOP was not repeated at any interval.	Canine retraction, Maxilla and Mandible	Vernier callipers measured the second premolar-canine distance at incisal, middle, and cervical regions pre- and post-retraction (3-week interval). Device: Digital Caliper (Vernier)
**Alqadasi et al**^31^	China	split mouth	3 months	24 (preferred arm: 12 which has 2 losses to follow up) MOP: 10 Control: 10	15-40 (20.89 ± 4.46)	12F, 9M MOP: 6F, 4M Piezo: 6F, 5M	Three buccal MOPs were performed using an automated mini-implant driver at equal distances from the canine and the second premolar within the extraction space. Each perforation measured approximately 5-7 mm in depth and 1.5-2 mm in diameter. MOP was not repeated at any interval.	Canine retraction, Maxilla	Researchers utilized 3D digital models of teeth captured at multiple time points. Using Ortho Analyzer software, they established a stable reference based on the midpalatine raphe (palatal midline). Reference lines were drawn from the premolar and canine teeth to this midline. The distance between these lines was measured at each time point. Device: Digital Scanning & Software (Ortho Analyzer)
**Alkebsi et al**^29^	Jordan	split mouth	3 months	35 (losses to follow up: 3)	16-24.6 (19.26 ± 2.48)	24F, 8M	MOPs were performed using miniscrews (Aarhus Mini-Implant System, American Orthodontics, 1.5 × 6 mm), utilizing a handpiece to create 3 buccal perforations distal to the canine tooth. Each perforation measured 3-4 mm in depth and 1.5 mm in width, spaced 3 mm apart. MOP was not repeated at any interval.	Canine retraction, Maxilla	3D digital models were superimposed at the rugae area from baseline to subsequent intervals using the Ceramill Map 400 scanner and Ceramill Mind design software. Canine displacement was measured on these superimposed models by assessing the middle projection of the distal surface of the maxillary canines. Additionally, intraoral measurements were taken using a digital caliper by measuring the distance from the upper mesial wing of the canine bracket to the upper distal wing of the second premolar bracket. Device: Combined; Digital Caliper (for bracket-to-bracket measurement) and Digital Scanning & Software (3Shape R900) (Ceramill Map 400 and Ceramill Mind software)
**Hashem et al**^44^	Egypt	split mouth	4 months	18 (losses to follow up: 0) MOP I (single): 9 MOP II (repeated): 9 Control: 18	15-22 MOP I (single): (16.78 ± 2.22) MOP II (repeated): (17.56 ± 2.65)	N/A	Three MOPs were performed distal to the maxillary canines, positioned halfway between the canine and the second premolar within the extraction space, each measuring 4 mm in depth and 1.6 mm in diameter, and repeated monthly for 4 months in Group II.	Canine retraction, Maxilla	The primary outcome was 4 month canine distal movement measured using 3D digital models created from alginate impressions (baseline and 4 month) scanned with a Primescan (Sirona Primescan Scanner, Dentsply Sirona, USA with a precision of 0.0021 mm precision) and analysed in Dolphin software (Dolphin Imaging version 11.9, Patterson Inc, Chatsworth, California, USA) where they were virtually positioned in a mutual position similar to the baseline scanned model, superimposing the baseline 3D digital model (T0) on the 4th month's 3D digital model. The most medial end of the third rugae area, which was perpendicular to the mid-palatal raphe line at T0, defined the rugae line. Device: Digital Scanning & Software (Dolphin software)
**Joseph et al**^45^	India	split mouth	10 months	11 (losses to follow up: 0)	18-30 (19 ± 4.21)	9F, 2M	Three MOPs were performed in the extraction space of the first premolar, positioned at equal distances from the canine and the second premolar. A 2 × 6 mm mini-implant was used to create perforations, each measuring 2-3 mm in depth and 1.5 mm in width. MOPs was not repeated at any interval.	Alignment, Maxilla and Mandible	A digital Vernier caliper measured the distance between the canine cusp tip and first molar's mesiobuccal cusp tip. Device: Digital Caliper (Vernier)
**Kilinc and Baka**^46^	Turkey	parallel arm	16 weeks	45 (losses to follow up:1) Piezocision: 15 MOP: 15 (losses to follow up:1) Control: 15	≥14 Piezocision: 14.40-21.10 (16.99 ± 1.73) MOP: 15.18-22.42 (17.51 ± 2.79) Control: 14.55-21.46 (17.39 ± 2.16) *P*-value: .914	26F, 19M Piezocision: 9F, 6M MOP: 9F, 5M Control: 7F, 8M *P*-value: .603	MOPs were performed vertically using a mini-implant system equipped with a rubber stop for precise depth control, along with a disposable MOP device (manufactured by Propel Orthodontics). A total of 9 buccal perforations were made on the right and left sides of the mandible combined. The first perforation was positioned 2 mm apical to the alveolar crest. Each perforation measured 3 mm in depth and 1.5 mm in width and spaced 1 mm apart, and they were located between the mandibular canines and lateral incisors, as well as between the mandibular central incisors. MOPs was not repeated at any interval.	Alignment, Mandible	Little’s irregularity index measured from plaster models using digital caliper. LII measures the horizontal linear displacement of anatomic contact points of each mandibular incisor from the adjacent anatomic point and sums the 5 displacement together, Which represents the degree of anterior irregularity. Device: Digital Caliper (on plaster models)
**Bavikati et al**^35^	India	split mouth	114.5 ± 9.5 days	22 (losses to follow up: 0)	18-25	22F, 0M	Three vertical MOPs were performed using a TAD (Unitek™ TAD, 1.8 × 8 mm), placed distal to the canine, with a 2 mm gap between them. Each perforation measured 5 mm in depth and 1.2 mm in width. MOPs was not repeated at any interval.	Canine retraction, Maxilla	A digital Vernier caliper measured distances between vertical reference lines drawn along the long axes of canines and lateral incisors on dental casts. Device: Digital Caliper (Vernier)
**Kumar et al**^47^	India	parallel arm	4 months	20 (losses to follow up: 0)	18-35 MOP: (19.5 ± 2.67) Control: (20.3 ± 2.23)	13F, 7M	Three vertical MOPs were performed by FavAnchor (S H Pitkar Orthotools Pvt, Pune, India; diameter 1.5 × 4 mm), consisting of a hand-held driver with stainless steel MOP screws for single use. MOPs placed in the labial cortical plate along the long axis of the 6 anterior teeth in each interdental region and distal to the root of the canines on both sides; spaced 5 mm apart while the first MOP was positioned 6 mm from the free gingival margin. totalling 21 buccal MOPs per arch at the start of space closure (T0), with the procedure repeated once at the first monthly follow-up (T1).	Alignment, Maxilla and Mandible	Digital models created from scanned plaster casts; extraction space measurements performed digitally. Device: Digital Scanning & Software (scanned plaster models)
**Al-Attar et al**^26^	Iraq	parallel arm	Approximately 20 weeks MOP: 10.41 (CI: 9.92-10.89) weeks Control: 16.62 (CI: 16.11-17.13)	35 (losses to follow up: 2) MOP:19 (losses to follow up: 2) Control:16	17-22 (18.97), (95% CI: 18.35-19.54)	20F, 13M	Self-drilling mini-implants (1.6 × 6 mm) (Dentaurum GmbH & Co KG, Ispringen, Germany) were utilized for the MOPs procedure. Two buccal MOPs were performed at each site: bilaterally between the mandibular canine and lateral incisor, as well as between the mandibular central incisors. The perforations were spaced 2 mm apart, with the first perforation positioned 2 mm from the crest. The depth of each perforation was standardized at 3 mm using an endodontic rubber stopper. The MOP procedure was not repeated at any subsequent interval.	Alignment, Mandible	Little's irregularity index measured on digital 3D scanned models. LII measures the horizontal linear displacement of anatomic contact points of each mandibular incisor from the adjacent anatomic point and sums the 5 displacement together. Which represents the degree of anterior irregularity. Device: Digital Scanning & Software
**Gümüş et al**^43^	Turkey	split mouth	3 months	20 (losses to follow up: 0)	15.9-25.0 (16.5)	17F, 3M	Three vertical MOPs were performed using miniscrews (1.5 × 8 mm length, American Orthodontics, Sheboygan, WI, USA,) with a screwdriver distal to the canine in the centre of the extraction region, which were 1.5 mm wide, 3-4 mm deep (determined by the alveolar bone and mucosa thickness). The perforations were spaced 3 mm apart, with the first perforation positioned 5 mm from the crest. MOPs were repeated every 28 days, for a total of 3 times.	Canine retraction, Maxilla	The distance between the most distal point of the canine and the most mesial point of the second premolar was measured using 3D digital models. Device: Digital Scanning & Software
**Ozkan et al**^53^	Turkey	parallel arm	28 days	24 (losses to follow up: 0) MOP I (4 mm): 12 MOP II (7 mm): 12 Control: 12	16-21 MOP: (17.27 ± 1.22) Control: (18.13 ± 1.28)	12F, 12M	The MOP procedure involved 3 vertically aligned perforations (4 mm and 7 mm deep) per side, created using a modified 1.6 × 8 mm miniscrew with depth-marking elastomeric ligatures. Perforations were placed 3 mm distal to the canine, following its root axis. The MOP procedure was not repeated at any subsequent interval.	Canine retraction, Maxilla	The primary outcome was the monthly rate of canine retraction. Alginate impressions were taken before retraction and again at 28 days, when the study concluded. Plaster models from these two time points were scanned and digitized using Orthoanalyzer software (3Shape, Copenhagen, Denmark) and superimposed. Superimposition was performed on the third palatal rugae of the maxilla to ensure a stable reference. Canine movement was measured by calculating the distance between the most distal point of the canine and the most mesial point of the second premolar from an occlusal view. Molar mesialization was assessed by measuring the position of the mesiopalatal cusps of the first molars. Device: 3D digital models using Orthoanalyzer software
**Sahin et al**^16^	Turkey	parallel arm	Approximately 4 months MOP: 105.57 ± 18.34 days Control: 135.86 ± 15.12 days	28 (losses to follow up: 0)	16.25 ± 3.05	16F, 12M	The MOP protocol was carried out using the Propel® driver (Ossining, New York, USA), which is equipped with a 1.5 mm thick perforation tip and adjustable depth settings of 3, 5, and 7 mm, along with an LED depth indicator that signals when the desired depth is reached. Perforations were made directly in the keratinized gingiva, with depths of 3 mm in the anterior region (canine to canine), 5 mm in the premolar region (canine to molar), and 7 mm in the posterior region. Vertically aligned perforations were created at two points between the mandibular central incisors and 3 points between the lateral incisors and canines (total:8). In cases of crowding, additional perforations were performed in the posterior areas where tooth movement was necessary. The MOP procedure was not repeated at any subsequent interval.	Alignment, Mandible	Digital model analysis was performed on scanned plaster models to measure Little’s Irregularity Index (LII) on a monthly basis. LII measures the horizontal linear displacement of anatomic contact points of each mandibular incisor from the adjacent anatomic point and sums the 5 displacement together. Which represents the degree of anterior irregularity. Device: Digital Scanning & Software (scanned plaster models)
**Li et al**^49^	Australia	split mouth	12 weeks	20 (losses to follow up at T2-T3: 3)	12-25 (16.38)	11F, 9M	The Excellerator appliance (Propel Orthodontics, Propel Company, USA) was used to create two buccal perforations. Each perforation was performed at a depth of 5 mm in a vertical alignment, located 1-2 mm distal to the upper canines. The two single perforations were positioned 5 mm apart and placed at depths of 5 mm and 10 mm from the alveolar crest. The MOP procedure was not repeated at any subsequent interval.	Canine retraction, Maxilla	Distance between the contact points of the canine and second premolar was measured from the casts using digital callipers. Device: Digital Caliper
**Mordente et al**^52^	Brazil	parallel arm	4 weeks	42 (losses to follow up: 5) MOP: 19 Control: 18	16-40 MOP: (24.3 ± 8.1) Control: (22.2 ± 4.2)	20F 17M	The MOP procedure was performed using CBCT-guided surgical guides made from mm Essix ACE thermoformed plastic. A total of 18 perforations (9 buccal and 9 palatal) were created using a 1.6 mm SS surgical bur, with depths of 3 mm on buccal surfaces and 5 mm on palatal surfaces, controlled by a patented depth cursor. The perforations were placed perpendicular to the alveolar bone in specific interproximal locations: between canines and lateral incisors, between central and lateral incisors, and between central incisors (both buccal and palatal aspects). Two vertically aligned perforations were placed distal to each maxillary incisor, spaced 5 mm apart, with the first perforation positioned 6 mm from the gingival margin. However, between the central incisors, only the most apical perforation was made due to cervical root proximity concerns. The MOP procedure was not repeated during subsequent intervals.	Alignment, Maxilla	Tooth movement was assessed through digital model superimposition using the palatal rugae as a stable reference. CBCT scans were used to evaluate changes in root length and tooth inclination. Device: Digital Scanning & Software

Studies measured tooth movement in several ways. Across the 39 studies, we classified the techniques of distance measuring to better understand heterogeneity.8 studies fell into 1 of 3 camps: canine-to-lateral-incisor (CL), canine-to-second-premolar (C2P), or model-based/palatal-rugae superimposition (C-Model) (Full classification details are shown in Appendix Table 3).

Across the included literature, pain was assessed in 20 trials: on day 1 mean scores ranged from 12 mm to 67.7 mm on a 100-mm visual-analogue. values generally reverted to baseline by day 7. Root resorption was evaluated in 10 studies, with the greatest root-length shortening in a micro-osteoperforation (MOP) cohort reaching 0.87 mm. Nine trials tracked molar anchorage: although 1 reported significantly less mesial drift (1.3-1.5 mm) in MOP groups, most detected no statistically significant anchorage loss. Periodontal health stayed comparable on both sides except for a brief uptick in the gingival index at 1 month. Finally, 2 mechanistic investigations showed spikes in inflammatory markers at 24 h-most notably a roughly 3-fold rise in IL-1β, with smaller surges in IL-6 and TNF-α-followed by a return toward baseline within 4 weeks. Full outcome details appear in Appendix Table 5.

### Risk of bias assessment (quality assessment)

Using the Cochrane RoB 1 tool, the high proportion of studies appeared to demonstrate low risk in random sequence generation (28 studies), management of incomplete outcome data (attrition bias) (38 studies), and control of other biases (33 studies). Blinding of outcome assessment (detection bias) was also largely rated as low risk (27 studies). However, allocation concealment (selection bias) presented a mixed profile: while 51% (20 studies) were classified at low risk, 44% (17 studies) were judged as unclear risk and 5% (2 studies) as high risk. These proportions were carefully verified to ensure full consistency with the study‑level judgments reported in Table 2. Similarly, selective reporting (reporting bias) appeared to be a notable concern; although 46% (18 studies) were assessed as low risk, 51.3% (20 studies) were rated as unclear risk and 2.6% (1 study) as high risk, suggesting a potential for bias in these latter domains across the included literature.

**Table 2:** Results of studies’ risk of bias assessment.

(ROB assessment results appear in table 2, full table with justifications for each domain is in Appendix Table 6).

### Quantitative synthesis: overall effect of micro-osteoperforation (MOP) on tooth movement speed

The meta-analysis first quantified MOP’s early impact on tooth displacement: pooling 31 trials yielded a 4-week SMD of 1.64 (95% CI: 1.18 to 2.10) with a wide prediction interval from –0.893 to 4.181, signifying a robust but not universally guaranteed acceleration. Extending the horizon to the final follow-up (3 weeks to ten months across 39 trials) produced a similar overall SMD of 1.66 (95% CI: 1.27 to 2.05) and a prediction interval of −0.714 to 4.037. Both pooled estimates were accompanied by pronounced inconsistency-I² reached 91.05% at 4 weeks and 89.56% at the last assessment (Forest plots in Appendix Figures 1 and 2).

### Heterogeneity in primary outcome: tooth movement speed

Subgrouping and meta-regression were undertaken to probe the pronounced inconsistency in pooled SMDs, concentrating on the 4-week window. After Bonferroni adjustment, several study and intervention attributes appeared to emerge as significant effect modifiers. Geography, the metric used to quantify OTM, the anatomical landmarks chosen, and bracket prescription all appeared to associate with variations in outcomes, while intricate interactions among perforation depth, arch, and perforation count seemed to further stratified efficacy. Within certain narrowly defined strata heterogeneity all but disappeared appeared notably reduced-for example, Roth brackets (k = 5) yielded an SMD of 0.51 with I² = 0%, perforations ≥5 mm in the maxilla (k = 6) produced an SMD of 0.27 with I² = 0%, and protocols deploying ≤3 perforations <5 mm deep in both arches (k = 2) showed an SMD of 1.62, again with I² = 0%. Yet most broader subgroups still displayed considerable residual variability (table 3, full forest plots are in Appendix Figure 3 to 29 and Table 7). We emphasize that subgroup strata supported by very few studies (eg, k ≤ 2 per subgroup) may produce imprecise estimates and should be considered hypothesis-generating; accordingly, these results are now presented in the Appendix and are explicitly described as exploratory.

**Table 3:** Subgroup analyses for MOP effect on OTM.

Subgroup	Items	Study numbers	Change of SMD	CI (95%)	I^2^ (%)	*P*-value(between groups)
**Based on WHO classifications**	EMRO	11	2.10	[1.16-3.04]	94.37	Raw: <0.001 B*: <0.022
	EURO	4	0.73	[0.21-1.26]	42.47	
	PAHO	3	2.41	[−0.17 to 4.99]	94.22	
	SEARO	10	0.52	[0.39-0.66]	86.10	
	WPRO	3	0.05	[−0.40 to 0.51]	0	
**Study design**	Parallel Arms	17	2.27	[1.50-3.04]	92.26	Raw: 0.01 B: 0.220
	Split Mouth	14	1.08	[0.55-1.61]	87.70	
**Instrument for MOP**	Propel	7	2.97	[1.44-4.49]	95.72	Raw: 0.08 B: 1.000
	Miniscrew/Mini-implant	22	1.52	[1.05-1.99]	88.81	
**OTM measurement**	Digital Caliper	16	2.43	[1.70-3.16]	92.64	Raw: <0.001 B: <0.022
	Digital Scanning	14	0.97	[0.46-1.48]	83.41	
				
**Malocclusion**						Raw: 0.03 B: 0.660
	Class 1 only	3	2.12	[0.74-3.50]	86.57	
	Class 2 only	7	3.06	[1.33-4.80]	96.40	
	Crowding	5	1.71	[0.64-2.78]	86.83	
	Mixed/Multiple	11	1.21	[0.64-1.78]	85.97	
	Other	4	1.05	[0.10-2.00]	86.63	
**Measured distance**	Canine-Second Premolar	7	1.05	[0.43-1.66]	77.66	Raw: <0.001 B: <0.022
	Canine-Lateral Incisor	6	4.94	[3.09-6.79]	94.84	
	Model-based custom references	8	0.63	[0.10-1.16]	82.11	
				
	Extraction space	3	1.96	[0.08-3.84]	91.65	
	LII Little’s Irregularity Index	5	1.71	[0.64-2.78]	86.83	
**Type of bracket**						Raw: <0.001 B: <0.022
	MBT	17	1.69	[1.01-2.37]	91.19	
				
	Roth	5	0.51	[0.17-0.85]	0	
				
**Working wire dimension**	0.019 × 0.025	18	1.72	[1.18-2.26]	89.27	0.01 B: 0.220
	0.017 × 0.025	4	1.04	[0.12-1.97]	87.10	
				
				
**Jaws**	Maxilla	20	1.43	[0.86-2.00]	91.64	0.49 B: 1.000
	Mandible	6	2.07	[0.93-3.21]	89.78	
	Maxilla and Mandible	5	1.95	[0.92-2.98]	86.85	
**Type of OTM**	Alignment	8	1.58	[0.64-2.51]	89.06	Raw: <0.001 B: <0.022
	Canine Retraction	22	1.75	[1.20-2.31]	92.06	
				
**Jaws and type of OTM**	Mandibular Alignment	5	1.71	[0.64-2.78]	86.83	Raw: <0.001 B: <0.022
				
				
	Maxillary Canine Retraction	18	1.60	[0.99-1.66]	92.17	
				
	Maxillary and Mandibular Canine Retraction	3	1.91	[1.27-2.55]	60.01	
				
**Perforation on palatal**	Yes	5	1.87	[0.56-3.19]	93.75	Raw: 0.70 B: 1.000
	No	26	1.60	[1.10-2.10]	90.73	
**Depth**	<5 mm	19	1.77	[1.25-2.29]	88.86	Raw: 0.01 B: 0.220
	≥5 mm	9	0.63	[0.00-1.26]	83.58	
**Female**	≤60%	18	1.77	[1.14-2.40]	92.01	Raw: 0.35 B: 1.000
	>60%	9	1.30	[0.55-2.05]	88.94	
**Total perforation**	≤3	18	1.45	[0.86-2.05]	91.40	Raw: 0.37 B: 1.000
	>3	13	1.89	[1.15-2.64]	90.59	
**Depth and instrument**	<5 mm with Miniscrew/Mini-implant	15	1.78	[1.22-2.33]	87.93	Raw: 0.05 B: 1.000
	<5 mm with Propel	4	2.00	[0.45-3.56]	93.03	
	≥5 mm with Miniscrew/Mini-implant	6	0.75	[−0.19 to 1.70]	88.85	
				
**Depth and jaws**	<5 mm in Mandible	3	2.26	[0.36-4.46]	92.10	Raw: <0.001 B: <0.022
	<5 mm in Maxilla	12	1.42	[0.81-2.03]	88.53	
	<5 mm in Maxilla and Mandible	4	2.36	[1.44-3.29]	79.61	
				
	≥5 mm in Maxilla	6	0.27	[−0.02 to 0.56]	0	
				
**Depth and total perforations**	<5 mm and ≤3	10	1.44	[0.83-2.05]	85.95	Raw: <0.001 B: <0.022
	<5 mm and >3	9	2.11	[1.18-3.03]	91.41	
	≥5 mm and ≤3	6	0.27	[−0.01 to 0.56]	0	
	≥5 mm and >3	3	1.63	[−0.41 to 3.67]	93.83	
**Study design and total perforations**	≤3 in parallel arms study	8	3.05	[1.71-4.39]	94.36	Raw: <0.001 B: <0.022
	≤3 in split mouth study	10	0.67	[0.19-1.14]	81.26	
	>3 in parallel arms study	9	1.76	[0.83-2.68]	89.98	
	>3 split mouth study	4	2.21	[0.77-3.64]	93.25	
**Total perforations and jaw**	≤3 in Maxilla	15	1.58	[0.88-2.27]	92.41	Raw: 0.16 B: 1.000
	≤3 in Maxilla and Mandible	3	1.14	[0.23-2.05]	79.68	
	>3 in Mandible	6	2.07	[0.93-3.21]	89.78	
	>3 in Maxilla	5	1.13	[0.09-2.16]	90.62	
				
**Jaw and total perforations and depth**	≤3, <5 mm in Maxilla	8	1.45	[0.68-2.21]	87.85	Raw: <0.001 B: <0.022
				
	≤3, ≥5 mm in Maxilla	5	0.30	[−0.01 to 0.60]	0	
				
	>3, <5 mm in Mandible	3	2.26	[0.36-4.16]	92.10	
	>3, <5 mm in Maxilla	4	1.39	[0.17-2.61]	92.13	
				
				
				
Repeat of MOP*	Yes	9	1.04	[0.44-1.64]	78.64	Raw: 0.07 B: 1.000
	No	30	1.73	[1.28-2.17]	90.05	

Note: The ‘*P*-value (between groups)’ refers to the overall subgroup comparison as originally computed for each subgroup family; the tables are split for reporting clarity based on evidentiary strength (≥3 studies in main text; 1 to 2 studies in Appendix).

B, Bonferroni-adjusted *P*-value for between-group differences; CI, confidence interval; I², heterogeneity; SMD, standardized mean difference (Hedges’s g).

⁎ ‘Repeat of MOP’ pertains to baseline to last measurement, whereas other rows are baseline to 4 week.

Measurement technique appeared to be a dominant source of divergence. Stratifying by method (Appendix Table 8) allowed pooling of mean differences (MDs) within homogeneous strata: 5 trials using Little’s Irregularity Index yielded a pooled MD of 1.24 mm (95% CI: 1.06-1.43) with I² = 0%, whereas 3 trials gauging extraction-space closure produced 0.33 mm (95% CI: 0.27-0.39) with low heterogeneity (I² = 13.15%). In stark contrast, measurements between the canine and lateral incisor returned 0.65 mm but with I² = 98.81%, underscoring how the choice of metric may drive much of the aggregate heterogeneity when raw MDs are considered.

Meta-regression (Appendix Table 9 and Figures 30 to 34) suggested that longer follow-up was associated with smaller effect sizes (β = −0.168; 95% CI: −0.228 to −0.096; adjusted *P* < .007), potentially explaining 26% of the between-study variance (residual I² = 86%). In models expressed in millimetres, each additional MOP session was associated with an incremental 0.54 mm of cumulative displacement (95% CI: 0.04-1.03), with reduced residual heterogeneity (residual I² = 57%). Because these analyses are study-level, findings are interpreted as associative and hypothesis-generating rather than causal.

### Quantitative synthesis: secondary outcomes

Under restricted-maximum-likelihood random-effects framework (Knapp–Hartung correction), it revealed no statistically significant MOP-related increases in postoperative pain on the VAS or in periodontal parameters such as gingival index, probing depth, or attachment loss; yet some estimates, notably pain at day 7 (MD 0.79, 95% CI: −5.28 to 6.85; I² = 98.4%), were accompanied by very wide confidence intervals and extreme heterogeneity, signalling substantial imprecision. By contrast, root-resorption data were remarkably uniform: a pooled mean difference of 0.01 mm (95% CI: −0.04 to 0.07; I² = 0%) indicated that MOP did not exacerbate this risk. Heterogeneity was further explored for immediate postoperative pain. Subgrouping by jaw, instrument, or study design produced no between-group differences after multiplicity adjustment, although uncorrected *P*-values suggested possible effects. Meta-regression robustly identified the number of perforations as a statistically significant covariate: each additional perforation raised the immediate VAS score by 0.463 units (Bonferroni-adjusted *P* = .0015), whereas age and sex did not remain significant after correction. Results and accompanying forest plots are provided in Appendix Tables 10 to 12 and Figures 35 to 50.

### Publication bias

According to the funnel plot (Appendix Figure 51) nearly all studies lie to the right of the pooled effect, with two small, extreme outliers beyond the 95% limits. Assessment for publication bias for the primary SMD outcome using Egger’s test was significant (*P* < .0001) (Appendix Table 13). However, the trim-and-fill analysis did not alter the pooled estimate (Appendix Table 14).

### Sensitivity analyses

Sensitivity analyses indicated robustness of the primary SMD outcome. Leave-one-out analysis showed no single study unduly influenced the pooled SMD (Hedges's g range 1.402 to 1.706) (Appendix Table 15 and Figure 52). Cumulative meta-analysis showed the MOP effect (SMD) was consistently large and significant from early studies onwards (Appendix Table 16 and Figure 53). Also, calculated change vs available change analysis modestly lowered the estimate (g = 1.1 vs 1.8; *P* = .18). Calculated average vs available average comparisons introduced greater heterogeneity but preserved a substantial benefit (g = 3.0 vs 1.0; *P* < .001). Collectively, these checks support a consistently large and robust MOP effect (Appendix Figure 54 to 56).

### Quality of evidence (GRADE)

For the primary tooth movement SMD outcomes, certainty was rated Moderate. This rating primarily reflected serious inconsistency (substantial heterogeneity) despite a consistently favourable direction of effect and plausible methodological explanations (eg, measurement method and follow-up duration). Meta-regression and subgroup analyses were used to explore heterogeneity and are interpreted as associative and hypothesis-generating; they were not used to increase certainty ratings. For mean differences within consistent measurement subgroups (eg, Little’s Irregularity Index), certainty pertains to that specific outcome and should not be interpreted as eliminating uncertainty in the broader pooled analysis (Table 4).

**Table 4:** Summary of findings (quality of evidence [GRADE]).

Outcome or sub-group (effect metric)	Effect Size	k	I²%	Point estimate[95% CI]	Risk-of-Bias	Inconsistency	Imprecision	Publication bias	Up-grades	Net score	Certainty
Tooth-movement, 4 weeks, pooled	Change of SMD	31	91	1.64 [1.18-2.10]	0	−1	0	0	-	3	⨁⨁⨁◯
Tooth-movement, 4 weeks, sb: C2P	Change of MD	7	75.5	0.51 [0.26-0.76]	−1	−2	0	0	-	1	⨁◯◯◯
	Change of SMD	7	77.7	1.05 [0.43-1.66]	−1	−2	0	0	-	1	⨁◯◯◯
Tooth-movement, 4 weeks, sb: CL – MD	Change of MD	6	98.8	0.65 [0.38-0.93]	−1	−2	0	0	-	1	⨁◯◯◯
	Change of SMD	6	94.8	4.94 [3.09-6.79]	−1	−2	0	0	-	1	⨁◯◯◯
Tooth-movement, 4 weeks, sb: Model-based – MD	Change of MD	8	89.1	0.27 [0.01-0.53]	0	−2	0	0	-	2	⨁⨁◯◯
	Change of SMD	8	82.1	0.63 [0.10-1.16]	0	−2	0	0	-	2	⨁⨁◯◯
Tooth-movement, 4 weeks, sb: C-TAD / Appliance – MD	Change of MD	2	67.6	0.29 [−0.12 to 0.70]	0	−1	−1	0	-	2	⨁⨁◯◯
	Change of SMD	2	77.7	0.65 [−0.40 to 1.70]	0	−2	−1	0	-	1	⨁◯◯◯
Tooth-movement, 4 weeks, sb: Extraction space – MD	Change of MD	3	13.2	0.33 [0.27-0.39]	0	0	0	0	-	4	⨁⨁⨁⨁
	Change of SMD	3	91.7	1.96 [0.08-3.84]	0	−2	0	0	-	2	⨁⨁◯◯
Tooth-movement, 4 weeks, sb: LII	Change of MD	5	0	1.24 [1.06-1.43]	0	0	0	0	-	4	⨁⨁⨁⨁
	Change of SMD	5	86.8	1.71 [0.64-2.78]	0	−2	0	0	-	2	⨁⨁◯◯
Tooth-movement, final	Change of SMD	39	90	1.66 [1.27-2.05]	0	−1	0	0	-	3	⨁⨁⨁◯
Root resorption	Change of MD	6	0	0.01 [−0.04 to 0.07]	0	0	−1	0	-	3	⨁⨁⨁◯
	Change of SMD	6	0	0.02 [−0.03 to 0.07]	0	0	−1	0	-	3	⨁⨁⨁◯
Attachment loss	Change of MD	6	89	−0.26 [−0.86 to 0.33]	0	−2	−1	0	-	1	⨁◯◯◯
	Change of SMD	6	83	−0.36 [−1.28 to 0.56]	0	−2	−1	0	-	1	⨁◯◯◯
Gingival index	Change of MD	4	67	0.03 [−0.30 to 0.37]	0	−1	−1	0	-	2	⨁⨁◯◯
	Change of SMD	4	59	0.06 [−0.79 to 0.91]	0	−1	−1	0	-	2	⨁⨁◯◯
Pocket depth	Change of MD	3	84	0.19 [−0.63 to 1.00]	0	−2	−1	0	-	1	⨁◯◯◯
	Change of SMD	3	87	0.60 [−2.00 to 3.20]	0	−2	−1	0	-	1	⨁◯◯◯
Immediate pain	MD	8	83	0.96 [−0.08 to 1.99]	−1	−2	−1	0	-	1	⨁◯◯◯
	Change of SMD	8	82	0.37 [−0.35 to 1.08]	−1	−2	−1	0	-	1	⨁◯◯◯
24 h pain-change	Change of MD	7	15	−0.13 [−0.63 to 0.36]	−1	0	−1	0	-	2	⨁⨁◯◯
	Change of SMD	7	0	−0.15 [−0.44 to 0.14]	−1	0	−1	0	-	2	⨁⨁◯◯
7-day pain-change	Change of MD	4	98	0.79 [−5.28 to 6.85]	−1	−2	−1	0	-	1	⨁◯◯◯
	Change of SMD	4	94	0.16 [−2.34 to 2.66]	−1	−2	−1	0	-	1	⨁◯◯◯

CI, confidence interval; sb, subgroup; CL, canine - lateral incisor; C2P, canine - second premolar; C1M, canine - first molar; ; C-Model, model-based custom references; C-TAD / Appliance, any measurement referencing a TAD; EXT, extraction space; I², heterogeneity; K, number of studies; LII only, Little’s Irregularity Index only; MD, mean difference; SMD, standardized mean difference (Hedges's g).

Downgrading display, 0 = no downgrade; −1 = downgrade one level (serious); −2 = downgrade two levels (very serious). Certainty is floored at Very low.

Net score: Shown as a transparency aid mapping the starting level (High = 4) minus downgrades (and plus upgrades, if any). In this revision, we did not use upgrading to increase certainty.

Risk of bias: Because MOP is procedural, operator blinding is not feasible and participant blinding can be compromised. We therefore applied outcome-specific judgments: we downgraded risk-of-bias for patient-reported pain outcomes (where lack of blinding can influence reporting). For objective outcomes (tooth movement and periodontal measures assessed on models/CBCT), we did not automatically downgrade solely for operator/participant blinding but relied on allocation concealment and blinded outcome assessment as key safeguards.

Inconsistency: Inconsistency judgments considered I² alongside direction/overlap of effects and plausible methodological explanations (eg, measurement method and follow-up duration). For the primary pooled tooth-movement outcomes, we downgraded one level because heterogeneity remained substantial but effects consistently favoured MOP and moderator analyses suggested plausible sources.

Imprecision: Downgraded when confidence intervals were wide and/or crossed clinically important thresholds, or when the available information size was limited.

Publication bias: Formal small-study effect methods were interpreted cautiously; certainty was not downgraded when evidence was inconclusive.

Meta-regression: Meta-regression was interpreted as associative and hypothesis-generating (study-level) and used to explore heterogeneity; it was not used to increase certainty. Given substantial heterogeneity, the expected effect in a new setting may vary widely (wide prediction interval).

## Discussion

This systematic review and meta-analysis synthesized evidence from 39 randomized controlled trials published between 2013 and 2024, together enrolling more than 1000 participants. When MOPs were compared with true no-intervention controls, the pooled SMDs were 1.64 during the first treatment month and 1.66 over each study’s total follow-up, suggesting a substantial standardized acceleration of tooth movement. Because these SMDs synthesize outcomes derived from different measurement methods, they should not be interpreted as exact millimetre gains. Clinically interpretable estimates are perhaps better represented by method-consistent mean differences, such as a 1.24-mm greater improvement in Little’s Irregularity Index in the first month and a 0.33-mm greater extraction-space closure in relevant trials (Appendix Table 8). Any translation of these findings into time savings should be viewed as approximate and dependent on baseline movement rates and modelling assumptions.

Previous systematic reviews reported considerably smaller effects. Shahabee et al (6 studies) found a monthly increment of approximately 0.5 mm, grading the certainty of evidence as ‘very low’.^21^ A stricter appraisal by Sivarajan et al (8 studies) reduced the average gain to a negligible value.^20^ Mohaghegh et al, (15 studies) obtained an SMD of 0.42, though the signal disappeared when high-risk studies were excluded.^59^ Santos et al likewise reported no clinically meaningful acceleration: across twelve RCTs, subgroup meta-analyses showed no difference in monthly displacement for Propel-assisted movement (95% CI: −0.01 to 0.75) or other mini-screw systems (−0.02 to 0.31), with certainty rated low for tooth-movement outcomes.^60^ Bardideh et al reached a similar conclusion in an exploratory review of 4 RCTs, detecting no change in the rate or amount of molar distalization (MD 0.1 mm/month; *P* > .05), aside from transient procedure-day pain.^61^ Even analyses reporting positive findings were modest: Volodymyr et al synthesized 8 studies and calculated a pooled MD of 0.56 mm, a value still markedly below the magnitude observed in the present review.^62^

Two methodological refinements may help explain the larger, more precise estimate obtained in the present review: all included trials compared MOPs with untreated controls, and the model explicitly accounted for the potential weekly decline of approximately 0.17 mm in displacement, thereby distinguishing protocols that repeated MOPs from those that applied the procedure once only. This approach appeared to nearly doubled the headline effect and reduce residual heterogeneity, suggesting that earlier, flatter estimates were mainly possibly artefactual.

### Interpretation of effect size and biological context

A controlled cortical perturbation triggers a brief catabolic-anabolic burst: osteoclastic activity, inflammatory mediators and regional perfusion all surge, cresting within the first fortnight before drifting back to baseline. Accordingly, the present data show the steepest tooth-movement gains in weeks 1 to 4, tapering thereafter unless the stimulus is renewed. Finite-element models echo this timeline, depicting a parallel collapse in strain gradients by week 4. The fading effect therefore reflects an expected physiological reset, not therapeutic failure, and can be restored by periodic re-application of the stimulus.

### Sources of heterogeneity

Between-study inconsistency was pronounced (I² > 89%), underscoring the potential role of contextual modifiers. Three factors appear to account for most of this scatter. First, the measurement method: studies gauging movement by the interdental canine–lateral-incisor distance returned an outsized SMD of 4.94, whereas digital-model superimpositions or TAD-based reference systems produced more restrained yet still favourable effects (SMDs: 0.63-0.65). Second, follow-up length: meta-regression suggests a potential decline in the magnitude of the standardized effect with longer follow-up (β = −0.168 SMD units per week). Repeating MOPs at roughly 4-week intervals appeared associated with a larger standardized benefit and reduced residual heterogeneity. These findings may support a transient, renewable biological effect rather than a fixed millimetre-per-week decrement across all measurement approaches. By contrast, perforation diameter, spacing, and patient age appear to lose significance after multiplicity correction, and heavier orthodontic forces actually blunt the benefit, hinting at a possible biological ceiling once baseline loading is high.

### Safety profile and patient-reported outcomes

Across 8 trials, immediate postoperative pain was associated with an increase by about 1 VAS unit (MD 0.96) yet normalized within 24 h (24-h SMD −0.15) and was statistically indistinguishable from controls at day 7 (MD 0.79, wide CI). Periodontal parameters stayed clinically stable: gingival index (MD 0.03), pocket depth (MD 0.19) and attachment loss (MD −0.26), all cantered on zero with little heterogeneity. Root resorption was negligible at 0.01 mm with perfect homogeneity (I² = 0). Collectively, these secondary outcomes suggest that MOP is associated with only brief, mild discomfort while periodontal parameters and root resorption did not show clinically important deterioration in the included trials.

### Methodological strengths and limitations

Strengths of this review include preregistration in PROSPERO, adherence to PRISMA guidelines, comprehensive database searches, independent dual screening, duplicate data extraction and the use of random-effects models with influence diagnostics.

Nevertheless, limitations must be acknowledged. Allocation concealment was inadequately reported in nearly half of the trials, and the Egger’s regression intercept suggests the possibility of small-study publication bias. Furthermore, most participants were adolescents or young adults enrolled in centres within the EMRO and SEARO regions, limiting generalizability to older populations and other ethnic backgrounds. Median follow-up was approximately 6 months; thus, the capacity of MOPs to shorten total treatment time remains uncertain.

### Clinical implications

Clinically, repeating every 4 weeks a triad of buccal MOP (≈ 1.5-1.6 mm in diameter, 3-5 mm deep, 3-5 mm apart and placed distal to the moving tooth) under light-to-moderate forces appears to facilitate a substantial acceleration of OTM. The pooled 4-week SMD is 1.64 (95% CI: 1.18-2.10) and the overall SMD 1.66 suggests a strong standardized benefit across heterogeneous measurement approaches. For clinical magnitude, we note the method-specific mean differences observed in homogeneous subsets (eg, 1.24 mm improvement in Little’s Irregularity Index in the first month; 0.33 mm greater extraction-space closure). Any implications for shortening particular treatment phases should be considered approximate and based on these comparable outcomes rather than on direct conversion of the overall SMD. The effect seems more homogeneous when ≤3 perforations are kept shallower than 5 mm (subgroup SMD 1.62; I² = 0; k = 2, exploratory); drilling deeper (≥5 mm) or adding extra-including palatal-holes may reduce the gain to a small, non-significant SMD of ≈ 0.30 and may be associated with immediate pain by about 0.46 VAS units per additional perforation (study-level association).

Our prespecified meta-regression suggested that longer follow-up was associated with smaller standardized effects (β = −0.168 SMD units per week), and that trials repeating MOP sessions tended to report greater cumulative movement (study-level association). These patterns are hypothesis-generating and should not be interpreted causally. Given the substantial between-study heterogeneity, the expected effect in a new clinical setting may vary widely (ie, a wide prediction interval), and the magnitude of benefit may be smaller than the pooled mean.

Also, it should be mentioned when MOPs are used to treat anterior crowding, as measured by Little’s Irregularity Index, it appears to accelerate alignment by about 1.24 mm during the first month (MD 1.24; I² = 0) which rated high certainty by GRADE.

### Directions for future research

Future investigations should prioritize large, multicentre trials that monitor participants through debonding and retention to determine whether repeated MOPs reduce overall treatment duration, appointment frequency or aligner refinements. The Development of a core outcome set would mitigate measurement-driven heterogeneity. Head-to-head comparisons testing different perforation numbers, depths and spacings-and stratifying by bone density and periodontal phenotype-are required to optimize protocols.

## Conclusion

Moderate-certainty evidence suggests that micro-osteoperforations may accelerate orthodontic tooth movement without clear evidence of clinically important periodontal harm in the included trials. The effect appears transient and context-dependent, and substantial heterogeneity implies that the magnitude of benefit may vary across settings. Larger multicentre trials using standardized outcomes are needed to determine whether early acceleration translates into shorter overall treatment time.

## Author contributions

*A.H. (Ali Heidari):* Conceptualization, methodology, protocol registration (PROSPERO), full-text screening, data extraction oversight and dispute resolution, risk of bias assessment, data synthesis and statistical analysis (Stata), GRADE certainty assessment, and drafting/critical revision of the manuscript. Prepared the response to reviewers. *M.A. (Masood Azarbayjani):* Title and abstract screening, risk of bias assessment, drafting the manuscript, and prepared the response to reviewers. *S.M. (Sepideh Mojiri):* Full-text screening, data extraction, and drafting the manuscript. *N.K. (Nima Khamisi):* Title and abstract screening, dispute resolution for screening and risk of bias assessment, data extraction, and drafting the manuscript. All authors Read and approved the final manuscript and agree to be accountable for all aspects of the work.

## Ethical approval

Ethical approval was not required for this meta-analysis as it was based exclusively on the analysis of previously published data. There was no direct patient involvement in this research. The data that support the findings of this study are available within the articles included in the systematic review.

## Data availability statement

All data generated or analysed during this study are included in this published article and its supplementary information files. The data are also available from the corresponding author upon reasonable request.

## Conflict of interest

None disclosed.

## Appendix Supplementary materials

Image, application 1

## Footnotes

Supplementary material associated with this article can be found in the online version at doi:10.1016/j.identj.2026.109536.
