# Supplementary material for: Effect of Micro-Osteoperforations on Orthodontic Tooth Movement: A Systematic Review and Meta-Analysis
Source: Int Dent J. 2026 Apr 28;76(4):109536. doi: 10.1016/j.identj.2026.109536 (PMC13140058; doi:10.1016/j.identj.2026.109536)
Supplement: Supplementary file 1 [file mmc1.docx]

Contents

[PRISMA Check List	5](#_Toc216875757)

[Appendix Table 1. Detailed search queries for different databases. December 11th - 14th, 2024	9](#_Toc216875758)

[Supplementary Methods – Multiplicity Control	13](#_Toc216875759)

[Supplementary Methods – Heterogeneity & Moderator Analysis	13](#_Toc216875760)

[Supplementary Methods – GRADE Criteria and Procedures	14](#_Toc216875761)

[Appendix Table 2. Characteristics of the Included Studies (Full Version)	15](#_Toc216875762)

[Appendix Table 3. Standardizing Measured Distances	65](#_Toc216875763)

[**Appendix Table 4 Exploratory Subgroup Levels for MOP Effect on OTM (subgroup levels with 1–2 studies)**	67](#_Toc216875764)

[Appendix Table 5. Outcomes’ Summarizes	69](#_Toc216875765)

[Appendix Table 6. Risk of Bias Assessment (Quality Assessment)	180](#_Toc216875766)

[Appendix Figure 1. Change of SMD from Baseline to 4th week Forest Plot	214](#_Toc216875767)

[Appendix Figure 2. Change of SMD from Baseline to Last Measurements Forest Plot	215](#_Toc216875768)

[Appendix Figure 3. Subgroup Analysis Based on Countries (WHO Classification) Forest Plot	216](#_Toc216875769)

[Appendix Figure 4. Subgroup Analysis Based on Study Designs Forest Plot	217](#_Toc216875770)

[Appendix Figure 5. Subgroup Analysis Based on Instrument that is Used for MOP Forest Plot	218](#_Toc216875771)

[Appendix Figure 6. Subgroup Analysis Based on Instruments that is used for Distance Measurement Forest Plot	219](#_Toc216875772)

[Appendix Figure 7. Subgroup Analysis Based on Type of Malocclusion Forest Plot	220](#_Toc216875773)

[Appendix Figure 8. Subgroup Analysis Based on Measured Distance Forest Plot	221](#_Toc216875774)

[Appendix Figure 9. Subgroup Analysis Based on Brackets Forest Plot	222](#_Toc216875775)

Appendix Figure 10. Subgroup Analysis Based on Wires Dimensions Forest Plot	223

[Appendix Figure 11. Subgroup Analysis Based on Jaw Forest Plot	224](#_Toc216875777)

[Appendix Figure 12. Subgroup Analysis Based on Type of Orthodontic Tooth Movement Forest Plot	225](#_Toc216875778)

[Appendix Figure 13. Subgroup Analysis Based on Jaw and Type of Orthodontic Tooth Movement Forest Plot	226](#_Toc216875779)

[Appendix Figure 14. Subgroup Analysis Based on Palatal Perforations Forest Plot	227](#_Toc216875780)

[Appendix Figure 15. Subgroup Analysis Based on Depth of Perforations Forest Plot	228](#_Toc216875781)

[Appendix Figure 16. Subgroup Analysis Based on Percentage of Females Forest Plot	229](#_Toc216875782)

[Appendix Figure 17. Subgroup Analysis Based on Number of Perforations Forest Plot	230](#_Toc216875783)

[Appendix Figure 18. Subgroup Analysis Based on Depth of Perforations and the Instrument that is Used for MOP Forest Plot	231](#_Toc216875784)

[Appendix Figure 19. Subgroup Analysis Based on Depth of Perforations and the Jaw(s) Forest Plot	232](#_Toc216875785)

[Appendix Figure 20. Subgroup Analysis Based on Depth and Total number of Perforations Forest Plot	233](#_Toc216875786)

[Appendix Figure 21. Subgroup Analysis Based on Total Number of Perforations and Study Designs Forest Plot	234](#_Toc216875787)

[Appendix Figure 22. Subgroup Analysis Based on Number of Total Perforations and Jaw(s) Forest Plot	235](#_Toc216875788)

[Appendix Figure 23. Subgroup Based on Total Number and Depth of Perforations and Jaw(s) Forest Plot	236](#_Toc216875789)

[Appendix Figure 24. Subgroup Analysis Based on Repeat of MOP Forest Plot	237](#_Toc216875790)

[Appendix Figure 25. Subgroup Analysis Based on ROB Domain (Random Sequence Generation Domain) Forest Plot	238](#_Toc216875791)

[Appendix Figure 26. Subgroup Analysis Based on ROB Domain (Allocation Concealment) Forest Plot	239](#_Toc216875792)

[Appendix Figure 27. Subgroup Analysis Based on ROB Domain (Blinding of Outcome Assessment) Forest Plot	240](#_Toc216875793)

[Appendix Figure 28. Subgroup Analysis Based on ROB Domain (Incomplete Outcome Data) Forest Plot	241](#_Toc216875794)

[Appendix Figure 29. Subgroup Analysis Based on ROB Domain (Selective Reporting) Forest Plot	242](#_Toc216875795)

[Appendix Table 7. Subgroup Analyses	243](#_Toc216875796)

[Appendix Table 8. Subgroup Analyses Based on Tooth movement	246](#_Toc216875797)

[Appendix Table 9. Meta Regression for Different Covariates that Affect Relation of MOP and OTM	247](#_Toc216875798)

[Appendix Figure 30. Bubble Plot: SMD and Number of Repeats	248](#_Toc216875799)

[Appendix Figure 31. Bubble Plot: SMD and Age of Participants (years)	249](#_Toc216875800)

[Appendix Figure 32. Bubble Plot: SMD and Perforations' Distance Apart (mm)	250](#_Toc216875801)

[Appendix Figure 33. Bubble Plot: SMD and Diameters of Perforations (mm)	251](#_Toc216875802)

[Appendix Figure 34. Bubble Plot: SMD and Force Magnitutde for Orthodontic Treatment (gr)	252](#_Toc216875803)

[Appendix Table 10. Secondary Outcomes	253](#_Toc216875804)

[Appendix Figure 35. Change MD of Gingival Index Forest Plot	254](#_Toc216875805)

[Appendix Figure 36. Change SMD of Gingival Index Forest Plot	254](#_Toc216875806)

[Appendix Figure 37. Change MD of Pocket Depth Forest Plot	255](#_Toc216875807)

[Appendix Figure 38. Change SMD of Pocket Depth Forest Plot	255](#_Toc216875808)

[Appendix Figure 39. Immediate Pain (VAS) SMD Forest Plot	256](#_Toc216875809)

[Appendix Figure 40. Immediate Pain (VAS) MD Forest Plot	256](#_Toc216875810)

[Appendix Figure 41. Change MD of Pain After 24hours Forest Plot	257](#_Toc216875811)

[Appendix Figure 42. Change SMD of Pain After 24hours Forest Plot	257](#_Toc216875812)

[Appendix Figure 43. Change SMD of Pain After 7Days Forest Plot	258](#_Toc216875813)

[Appendix Figure 44. Change MD of Pain After 7Days Forest Plot	258](#_Toc216875814)

[Appendix Figure 45. Change MD of Root Resorption Forest Plot	259](#_Toc216875815)

[Appendix Figure 46. Change SMD of Root Resorption Forest Plot	259](#_Toc216875816)

[Appendix Figure 47. Change SMD of Attachment Loss Forest Plot	260](#_Toc216875817)

[Appendix Figure 48. Change MD of Attachment Loss Forest Plot	260](#_Toc216875818)

[Appendix Table 11. Immediate VAS: Subgroup Analyses	261](#_Toc216875819)

[Appendix Table 12. Immediate VAS: Meta-regression	261](#_Toc216875820)

[Appendix Figure 49. Subgroup Analysis for Immediate Pain (VAS) Based on Palatal Perforation Forest Plot	262](#_Toc216875821)

[Appendix Figure 50. Subgroup Analysis for Immediate Pain (VAS) Based on Jaw(s) Forest Plot	263](#_Toc216875822)

[Appendix Figure 51. Funnel Plot	264](#_Toc216875823)

[Appendix Table 13. Egger Test	265](#_Toc216875824)

[Appendix Table 14. Trim-And-Fill analysis	265](#_Toc216875825)

[Appendix Table 15. Leave-one-out analysis	266](#_Toc216875826)

[Appendix Figure 52. Leave-one-out Analysis Forest Plot	267](#_Toc216875827)

[Appendix Table 16. Cumulative meta-analysis	268](#_Toc216875828)

[Appendix Figure 53. Cumulative Meta-Analysis Forest Plot	269](#_Toc216875829)

[Appendix Figure 54. Calculated Vs. Available Change Forest Plot	270](#_Toc216875830)

[Appendix Figure 55. Calculated Vs. Available Average (1) Forest Plot	271](#_Toc216875831)

[Appendix Figure 56. Calculated Vs. Available Average (2) Forest Plot	272](#_Toc216875832)

# PRISMA Check List

| **Section and Topic** | **Item #** | **Checklist item** | **Location where item is reported** |
| --- | --- | --- | --- |
| **TITLE** | | |  |
| Title | 1 | Identify the report as a systematic review. | p. 1 |
| **ABSTRACT** | | |  |
| Abstract | 2 | See the PRISMA 2020 for Abstracts checklist. | p. 2 – Abstract |
| **INTRODUCTION** | | |  |
| Rationale | 3 | Describe the rationale for the review in the context of existing knowledge. | p.3 – Introduction |
| Objectives | 4 | Provide an explicit statement of the objective(s) or question(s) the review addresses. | p. 3 – Introduction, last paragraph |
| **METHODS** | | |  |
| Eligibility criteria | 5 | Specify the inclusion and exclusion criteria for the review and how studies were grouped for the syntheses. | p. 4 – "Inclusion and exclusion criteria" section |
| Information sources | 6 | Specify all databases, registers, websites, organisations, reference lists and other sources searched or consulted to identify studies. Specify the date when each source was last searched or consulted. | p. 4 – "Search strategy" section |
| Search strategy | 7 | Present the full search strategies for all databases, registers and websites, including any filters and limits used. | p. 4 – "Search strategy" section, noting details are in "Appendix Table 1" |
| Selection process | 8 | Specify the methods used to decide whether a study met the inclusion criteria of the review, including how many reviewers screened each record and each report retrieved, whether they worked independently, and if applicable, details of automation tools used in the process. | pp. 4-5 – “Data collection, extraction, and management”, 1^st^ paragraph |
| Data collection process | 9 | Specify the methods used to collect data from reports, including how many reviewers collected data from each report, whether they worked independently, any processes for obtaining or confirming data from study investigators, and if applicable, details of automation tools used in the process. | pp. 4-5 – “Data collection, extraction, and management”, 2^nd^ paragraph |
| Data items | 10a | List and define all outcomes for which data were sought. Specify whether all results that were compatible with each outcome domain in each study were sought (e.g. for all measures, time points, analyses), and if not, the methods used to decide which results to collect. | p. 4 – “Outcome definitions” |
|  | 10b | List and define all other variables for which data were sought (e.g. participant and intervention characteristics, funding sources). Describe any assumptions made about any missing or unclear information. | pp. 4-5 – “Data collection, extraction, and management” & p. 4 – "Outcome definitions" |
| Study risk of bias assessment | 11 | Specify the methods used to assess risk of bias in the included studies, including details of the tool(s) used, how many reviewers assessed each study and whether they worked independently, and if applicable, details of automation tools used in the process. | p. 5 – “Risk of bias assessment (Quality assessment)” |
| Effect measures | 12 | Specify for each outcome the effect measure(s) (e.g. risk ratio, mean difference) used in the synthesis or presentation of results. | p. 5 – “Data synthesis and statistical analysis”, 1^st^ paragraph |
| Synthesis methods | 13a | Describe the processes used to decide which studies were eligible for each synthesis (e.g. tabulating the study intervention characteristics and comparing against the planned groups for each synthesis (item #5)). | p. 5 – “Data synthesis and statistical analysis” 2^nd^ paragraph |
|  | 13b | Describe any methods required to prepare the data for presentation or synthesis, such as handling of missing summary statistics, or data conversions. | p. 4 – closing lines of “Outcome definitions” & p. 6 – "Sensitivity analyses" |
|  | 13c | Describe any methods used to tabulate or visually display results of individual studies and syntheses. | pp. 5-6 – “Heterogeneity, subgroup analysis...” & p. 6 – "Small-study effects..." |
|  | 13d | Describe any methods used to synthesize results and provide a rationale for the choice(s). If meta-analysis was performed, describe the model(s), method(s) to identify the presence and extent of statistical heterogeneity, and software package(s) used. | pp. 5-6 – “Data synthesis…”, “Heterogeneity, subgroup analysis…”, & p. 6 – “Software and data sharing” |
|  | 13e | Describe any methods used to explore possible causes of heterogeneity among study results (e.g. subgroup analysis, meta-regression). | pp. 5-6 – “Heterogeneity, subgroup analysis, and meta-regression” |
|  | 13f | Describe any sensitivity analyses conducted to assess robustness of the synthesized results. | p. 6 – “Sensitivity analyses” |
| Reporting bias assessment | 14 | Describe any methods used to assess risk of bias due to missing results in a synthesis (arising from reporting biases). | p. 6 – “Small-study effects and cumulative evidence” |
| Certainty assessment | 15 | Describe any methods used to assess certainty (or confidence) in the body of evidence for an outcome. | P. 6 – “Certainty of evidence (GRADE)” |
| **RESULTS** | | |  |
| Study selection | 16a | Describe the results of the search and selection process, from the number of records identified in the search to the number of studies included in the review, ideally using a flow diagram. | pp. 7-8 – “Study selection” plus Figure 1 |
|  | 16b | Cite studies that might appear to meet the inclusion criteria, but which were excluded, and explain why they were excluded. | pp. 7-8 – “Study selection” plus Figure 1 |
| Study characteristics | 17 | Cite each included study and present its characteristics. | p. 9 – “Characteristics of the included trials” & Table 1 |
| Risk of bias in studies | 18 | Present assessments of risk of bias for each included study. | p. 34 – “Risk of bias assessment (Quality Assessment)” and Table 2 |
| Results of individual studies | 19 | For all outcomes, present, for each study: (a) summary statistics for each group (where appropriate) and (b) an effect estimate and its precision (e.g. confidence/credible interval), ideally using structured tables or plots. | Summary data for each study are presented in Table 1. The text also states that "full outcome details appear in Appendix Table 5". |
| Results of syntheses | 20a | For each synthesis, briefly summarise the characteristics and risk of bias among contributing studies. | p. 36 – “Quantitative Synthesis: Overall Effect...” |
|  | 20b | Present results of all statistical syntheses conducted. If meta-analysis was done, present for each the summary estimate and its precision (e.g. confidence/credible interval) and measures of statistical ogeneity. If comparing groups, describe the direction of the effect. | p. 36 – “Quantitative Synthesis: Overall Effect…” & p. 40– “Quantitative Synthesis: Secondary Outcomes” |
|  | 20c | Present results of all investigations of possible causes of heterogeneity among study results. | p. 36 – “Heterogeneity in Primary Outcome: Tooth Movement Speed” & Table 3 |
|  | 20d | Present results of all sensitivity analyses conducted to assess the robustness of the synthesized results. | p. 40 – “Sensitivity analyses” |
| Reporting biases | 21 | Present assessments of risk of bias due to missing results (arising from reporting biases) for each synthesis assessed. | p. 40 – “Publication bias”; funnel / Egger |
| Certainty of evidence | 22 | Present assessments of certainty (or confidence) in the body of evidence for each outcome assessed. | pp. 40-41 –Quality of Evidence (GRADE)” & Table 4 |
| **DISCUSSION** | | |  |
| Discussion | 23a | Provide a general interpretation of the results in the context of other evidence. | p. 43 – Discussion section |
|  | 23b | Discuss any limitations of the evidence included in the review. | p. 44 – “Methodological Strengths and Limitations”, 2^nd^ paragraph |
|  | 23c | Discuss any limitations of the review processes used. | p. 44 – 2^nd^ paragraph (allocation concealment, publication bias) |
|  | 23d | Discuss implications of the results for practice, policy, and future research. | p. 45 – “Clinical Implications” & “Directions for Future Research” |
| **OTHER INFORMATION** | | |  |
| Registration and protocol | 24a | Provide registration information for the review, including register name and registration number, or state that the review was not registered. | p. 4 – Methods (“PROSPERO CRD42025633088”) |
|  | 24b | Indicate where the review protocol can be accessed, or state that a protocol was not prepared. | p. 4 – Methods (sentence citing PROSPERO record) |
|  | 24c | Describe and explain any amendments to information provided at registration or in the protocol. | NA |
| Support | 25 | Describe sources of financial or non-financial support for the review, and the role of the funders or sponsors in the review. | p. 47 – “Funding” (“no funding”) |
| Competing interests | 26 | Declare any competing interests of review authors. | p. 46 – “Declaration of Conflicting Interests” (“none declared”) |
| Availability of data, code and other materials | 27 | Report which of the following are publicly available and where they can be found: template data collection forms; data extracted from included studies; data used for all analyses; analytic code; any other materials used in the review. | p. 6 – “Software and data sharing” (Stata 17; data available on request/online) & p. 47 – “Data Availability Statement” |

# Appendix Table 1. Detailed search queries for different databases. December 11th - 14th, 2024

| Database | Keywords | Results |
| --- | --- | --- |
| Pubmed | ("micro osteoperforation"[All Fields] OR "micro osteoperforations"[All Fields] OR "micro perforations"[All Fields] OR "micro perforation"[All Fields] OR "osteoperforation"[All Fields] OR "mop"[All Fields] OR "mops"[All Fields] OR "minimally invasive"[All Fields] OR "corticotomy assisted"[All Fields] OR "corticotomy facilitated"[All Fields] OR "periodontal associated"[All Fields] OR "wilckodontics"[All Fields]) AND ("tooth movement techniques"[MeSH Terms] OR "tooth movement technique"[All Fields] OR "tooth movement"[All Fields] OR "orthodontic tooth movement"[All Fields] OR "tooth intrusion"[All Fields] OR "tooth depression"[All Fields] OR "tooth uprighting"[All Fields] OR "minor tooth movements"[All Fields] OR "minor tooth movement"[All Fields] OR "tooth movement orthodontic"[All Fields] OR "tooth displacement"[All Fields] OR "movement orthodontic tooth"[All Fields] OR "uprighting tooth"[All Fields] OR "accelerated tooth movement"[All Fields] OR "movement orthodontic tooth"[All Fields] OR "intrusion tooth"[All Fields] OR "tooth intrusions"[All Fields]) | 263 |
| Scopus | ( TITLE-ABS-KEY ( "micro osteoperforation" OR "micro osteoperforations" OR "micro perforations" OR "micro perforation" OR "osteoperforation" OR "mop" OR "mops" OR "minimally invasive" OR "corticotomy assisted" OR "corticotomy facilitated" OR "periodontal associated" OR "wilckodontics" ) ) AND ( TITLE-ABS-KEY ( "tooth movement techniques" OR "tooth movement technique" OR "tooth movement" OR "orthodontic tooth movement" OR "tooth intrusion" OR "tooth depression" OR "tooth uprighting" OR "minor tooth movements" OR "minor tooth movement" OR "tooth movement orthodontic" OR "tooth displacement" OR "movement orthodontic tooth" OR "uprighting tooth" OR "accelerated tooth movement" OR "intrusion tooth" OR "tooth intrusions" ) ) AND PUBYEAR > 2000 AND PUBYEAR < 2025 | 314 |
| Embase | ('micro osteoperforation':ti,ab,kw OR 'micro osteoperforations':ti,ab,kw OR 'micro perforations':ti,ab,kw OR 'micro perforation':ti,ab,kw OR 'osteoperforation':ti,ab,kw OR 'mop':ti,ab,kw OR 'mops':ti,ab,kw OR 'minimally invasive':ti,ab,kw OR 'corticotomy assisted':ti,ab,kw OR 'corticotomy facilitated':ti,ab,kw OR 'periodontal associated':ti,ab,kw OR 'wilckodontics':ti,ab,kw)  AND  ('tooth movement techniques':ti,ab,kw OR 'tooth movement technique':ti,ab,kw OR 'tooth movement':ti,ab,kw OR 'orthodontic tooth movement':ti,ab,kw OR 'tooth intrusion':ti,ab,kw OR 'tooth depression':ti,ab,kw OR 'tooth uprighting':ti,ab,kw OR 'minor tooth movements':ti,ab,kw OR 'minor tooth movement':ti,ab,kw OR 'tooth movement orthodontic':ti,ab,kw OR 'tooth displacement':ti,ab,kw OR 'movement orthodontic tooth':ti,ab,kw OR 'uprighting tooth':ti,ab,kw OR 'accelerated tooth movement':ti,ab,kw OR 'intrusion tooth':ti,ab,kw OR 'tooth intrusions':ti,ab,kw) | 173 |
| Cochrane library | ("micro osteoperforation" OR "micro osteoperforations" OR "micro perforations" OR "micro perforation" OR "osteoperforation" OR "mop" OR "mops" OR "minimally invasive" OR "corticotomy assisted" OR "corticotomy facilitated" OR "periodontal associated" OR "wilckodontics"):ti,ab,kw  AND  ("tooth movement techniques" OR "tooth movement technique" OR "tooth movement" OR "orthodontic tooth movement" OR "tooth intrusion" OR "tooth depression" OR "tooth uprighting" OR "minor tooth movements" OR "minor tooth movement" OR "tooth movement orthodontic" OR "tooth displacement" OR "movement orthodontic tooth" OR "uprighting tooth" OR "accelerated tooth movement" OR "intrusion tooth" OR "tooth intrusions"):ti,ab,kw | 156 |
| ProQuest | abstract("micro osteoperforation" OR "micro osteoperforations" OR "micro perforations" OR "micro perforation" OR "osteoperforation" OR "mop" OR "mops" OR "minimally invasive" OR "corticotomy assisted" OR "corticotomy facilitated" OR "periodontal associated" OR "wilckodontics") AND abstract("tooth movement techniques" OR "tooth movement technique" OR "tooth movement" OR "orthodontic tooth movement" OR "tooth intrusion" OR "tooth depression" OR "tooth uprighting" OR "minor tooth movements" OR "minor tooth movement" OR "tooth movement orthodontic") | 73 |
| Google Scholar | allintitle: micro-osteoperforation AND tooth movement | 15 |

# Supplementary Methods – Multiplicity Control

To avoid multiplicity, following a reductionist approach where a study contained several MOP arms distinguished only by perforation number, the arms were merged and compared with a single control to preserve independence. When maxilla and mandible were reported separately, they were pooled with weighted means (and its effect assessed within subgroup analysis). In any other studies with more than two arms, the most relevant comparison arms were selected. Also, to limit false-positive findings, every p-value that first reached nominal significance (p<0.05) was re-checked with Bonferroni adjustment. A finding was declared significant only if it remained below both adjusted thresholds.

# Supplementary Methods – Heterogeneity & Moderator Analysis

Heterogeneity was quantified by the I^2^ statistic and visualized with forest plots including prediction intervals. Pre-specified subgroup analyses were conducted by age group, female proportion, perforation number, perforation site (buccal, palatal, both), treated jaw, style of movement, measurement method, repetition of MOPs, and overall risk-of-bias category. When ten or more studies were available, random-effects meta-regression was performed. Almost all subgroup analysis and meta-regression (except for duration of study and number of MOP repeats) was done for the baseline to 4thweek measurement of OTM.

Depth of the perforations (< 5 mm, 5-8 mm, > 8 mm), total perforations (≤3 vs > 3) and female proportion (≤60% vs > 60%) were analyzed as categorical moderators in separate random-effects subgroup models because the biological response is step-like around these thresholds. Residual heterogeneity was then explored with a mixed-effects meta-regression that retained all studies and included the continuous moderators mean age, applied force magnitude and follow-up time, together with study-level methodological covariates (split-mouth vs parallel design, measurement technique, jaw, and instrument type).

Due to the variety of methods used across trials to measure tooth movement (ranging from direct caliper measurements to indices like Little’s Irregularity Index), change of SMD, using Hedges's g, were used for the overall pooled analysis of tooth movement speed to ensure comparability. Change of MD in millimeters are considered later within subgroups defined by consistent measurement methodology.

# Supplementary Methods – GRADE Criteria and Procedures

# Certainty of evidence was appraised using the GRADE framework. Because all included studies were randomized trials, each outcome started at high certainty. We then assessed the five downgrading domains for each outcome/comparison: risk of bias, inconsistency, imprecision, indirectness, and publication bias. Risk of bias was informed by the Cochrane Risk of Bias tool (RoB 1). Because micro-osteoperforations are procedural, operator blinding is not feasible and participant blinding can be compromised. We therefore handled this limitation explicitly and outcome-specifically: for patient-reported outcomes (e.g., pain), lack of blinding was considered a meaningful source of bias and downgraded when appropriate; for objective outcomes (tooth movement and periodontal measures assessed on models/CBCT), we did not automatically downgrade solely for lack of operator/participant blinding but emphasized allocation concealment and blinded outcome assessment as key safeguards. Inconsistency was judged using GRADE principles, considering heterogeneity metrics (including I²) alongside direction of effects, overlap of confidence intervals, and plausible methodological explanations (e.g., measurement method and follow-up duration). Imprecision was assessed by the width of 95% confidence intervals relative to clinically important effects and the available information size. Indirectness was considered when populations, interventions, comparators, or outcomes diverged materially from the review question. Publication bias was explored when feasible, but formal small-study effect tests were interpreted cautiously and were not used to downgrade when evidence was inconclusive.

Meta-regression and subgroup analyses were prespecified and used to explore heterogeneity; because they are study-level, they are interpreted as associative and hypothesis-generating and were not used to increase certainty ratings. Detailed justifications for each judgment are provided in the footnotes of the Summary of Findings table (Table 4).

# Appendix Table 2. Characteristics of the Included Studies (Full Version)

| **Study** | **Country** | **Eligibility criteria** | **Study design** | **Duration** | **Samples** | **Age** | **Gender** | **Malocclusion** | **Method of orthodontics treatment** | **Details on MOP intervention** | **Type of tooth movement, jaw** | **Method of OTM measurement, Device** |
| --- | --- | --- | --- | --- | --- | --- | --- | --- | --- | --- | --- | --- |
| Golshah et al. ,2021 | Iran | Inclusion:  age: 16-25, skeletal class II division 1 malocclusion with molars in a full cusp class II relationship who required canine retraction following the maxillary first premolar extraction.  Exclusion:  systemic diseases, syndromic patients, craniofacial anomalies, previous orthodontic treatment, vertical skeletal discrepancy (62% ˂ Jarabak index ˂ 65%, and 228˚ ˂ Frankfort mandibular plane angle < 288˚), presence of high buccal frenum, inadequate attached gingiva, gingivitis, inadequate space between the roots, poor oral hygiene, tobacco use. | split mouth | 5 months | 25 (losses to follow up: 0) | 16-25 | 11F, 14M | skeletal Class II Division 1 | The treatment used Roth prescription brackets with Roth22 bands. The archwire sequence was 0.016-inch NiTi, 0.017 × 0.025-inch NiTi, and the working wire was 0.017 × 0.025-inch SS. Anchorage was reinforced using miniscrews placed buccally between the second premolar and first molar on both sides. | Five MOPs (3 buccal, 2 palatal) were created using G2 miniscrews (8 × 1.6mm) at 15 N·cm torque, positioned 3mm distal to the canine and 6-7mm from the gingival margin. Perforations were 3mm apart, 3-4mm deep, and 1.6mm wide, made perpendicular to the distal of the canine, without repetition at each interval. | Canine retraction,  Maxilla | Method 1: from the center of the canine’s distal prominence on superimposed models to its new position to quantify the distal movement of the canine tooth.  Method 2: from the canine cusp tip to a perpendicular line drawn from the miniscrews relative to the occlusal plane on initial and subsequent models to assess the positional change of the canine cusp tip as an alternative measure of OTM. |
| Thomas et al. ,2021 | India | Inclusion:  age: 19-25, class I bimaxillary protrusion or class II division 1 malocclusion requiring individual canine retraction with anchorage from the micro-implant following bilateral maxillary first premolar extraction, undergone first premolar extraction and micro implant placement before 6 months, completed alignment leveling stage, average Frankfort Mandibular Plane (FMA) angle :23-28˚, intact periodontium (< 10% bleeding sites with prob ing depths ≤ 3 mm), good oral hygiene maintenance (OHI-S score of 0.0–1.2).  Exclusion:  previous orthodontic treatment, tobacco smoking, systemic diseases, syndromes, and long-term corticosteroid therapy or other medications that slow down bone metabolism. | split mouth | 90 days | 33 (losses to follow up: 3) | 19-25 (22.1 ± 2.19) | 24F, 9M | Class I bimaxillary protrusion, Class II Division 1 malocclusion | The treatment used MBT prescription brackets with a 0.018-inch slot. The maxillary arch was treated using a 0.016 × 0.022-inch SS retraction archwire. Anchorage was supported by micro-implants (8 × 1.5mm) placed 5 mm apical to the alveolar crest between the maxillary second premolar and first molar. | Six buccal MOPs were placed 3mm apart vertically on the mesial and distal aspects of the canine root (3 MOPs per site), starting 6mm apical from the alveolar crest. A lance drill attached to a physiodispenser handpiece (500 rpm, 50 Ncm torque) was used. Perforations were 4mm deep and 2mm wide, positioned 2mm distal to the canine root on the distal side and in the inter-radicular bone between the canine and lateral incisor on the mesial side, without repetition at each interval. | Canine retraction,  Maxilla | Method 1 (D1): from the molar tube slot to the canine bracket slot to assess tooth movement. Method 2 (D2): from the molar tube hook to the micro-implant. |
| Martina et al. ,2022 | India | Inclusion:  both genders; age: 15-25, patients with an angle’s class I malocclusion, bilateral extraction of maxillary first premolars, Orthodontic patients with levelling and alignment completed in the maxillary arch, patients with a periodontally sound dentition, patients without systemic disease.  Exclusion:  craniofacial anomalies, history of bleeding disorders, signs and symptoms of gingival or periodontal disease, the use of analgesics, phenytoin, cyclosporine, anti-inflammatory drugs, systemic corticosteroids, and calcium channel blockers for more than three months, active metabolic bone disease, severe skeletal malocclusion requiring surgery. | parallel arm | 90 days | 40 (losses to follow up: 0)  3MOPs:10  2MOPs:10  Control:20 | 15-25 | 22F, 18M | Class I malocclusion | The treatment used MBT prescription brackets with a 0.022 × 0.028-inch slot. A standardized six-weekly archwire sequence was followed, ending with a 0.019 × 0.025-inch SS working wire. Buccal mini-implants (1.6mm) placed between the first molar and second premolar provided anchorage. Canine retraction began (immediately after MOP) using NiTi closed coil springs applying a standardized force of 150g per side, measured with a Dontrix gauge. | MOPs were placed distal to the canines using FavAnchor miniscrews (1.6 × 8mm) to create vertical perforations 3mm deep and 1.6mm wide, spaced 2mm apart. Group-I A received 3 buccal MOPs, while Group-II A received 2 buccal MOPs. The procedure was repeated every 30 days for 3 months. | Canine retraction,  Maxilla | The linear distance moved by the canine was measured by digital caliper (vernier) on study casts by drawing two perpendicular lines; from the mesial point of the third palatal rugae (reference point) and from the distal side of the canine to the mid-palatine suture. |
| Gulduren et al. ,2020 | Northern Cyprus | Inclusion:  both genders, age: 16-24, bilateral class II molar relationship, skeletal class I or mild class II relation ship, normodivergent skeletal pattern, no posterior crowding or diastemas, fully erupted maxillary first and second molars, congenitally missing or extracted third molars, good oral hygiene, absence of any periodontal diseases and alveolar bone loss, no untreated caries, and no smoking.  Exclusion:  systemic diseases, use of any medications that might have affected the biology of tooth movement, unilateral class II molar relationship, extreme skeletal class II relationship (ANB >7°), hypodivergent or hyperdivergent skeletal pattern, posterior crowding or diastemas, absence of maxillary second molars, presence of maxillary third molars, poor oral hygiene, periodontal disease or alveolar bone loss, untreated caries, and smoking | parallel arm | 12 weeks | 20 (losses to follow up: 2)  MOP:10 (losses to follow up: 1)  Control:10 (losses to follow up: 1) | 16-24  MOP: 16.5-23.8 (21.8)  Control: 16.5-23.7 (17.7) | 7F, 11M | Bilateral Class II molar relationship, skeletal Class I or mild Class II relationship | Two miniscrews (anterior: 2×11mm, posterior: 2×9mm) were inserted into the anterior palate for anchorage. Molar bands were placed on the maxillary first molars, and custom distalization mechanics were created. NiTi springs delivered a consistent bilateral force of 500g to the molars, with reactivation every 3 weeks. | Six buccal MOPs were performed in the maxillary molar alveolar regions, with two perforations at three sites: between the second premolars and first molars, between the first and second molars, and distal to the second molars, using a 1.4mm drill. Perforations were 5-6mm deep and 1.4mm wide. The procedure was repeated every three weeks for a total of three sessions. | Molar distalization,  Maxilla | Digital scans were taken at baseline and subsequent intervals using a CEREC Omnicam. The scans were analyzed with Geomagic Studio 2014 software. 3D models were aligned along the Y-axis, and the distance between the midpoint of the first miniscrew and the distal margin of the maxillary first molar was measured and changes in this indicating tooth movement, verified from occlusal and lateral views. |
| Feizbakhsh et al. ,2018 | Iran | Inclusion:  both genders, age: 18-33, class I dental protrusion.  Exclusion:  long-term use of antibiotics, phenytoin, cyclosporine, anti-inflammatory drugs, systemic corticosteroids, and calcium channel blockers, poor oral hygiene, class II and III dental malocclusion, skeletal problem, systemic disease, periodontal disease, Smoking, gingivitis and caries, probing depth > 4mm , dental crossbite and deviation in closure due to occlusal interference. | split mouth | 28 days | 20 (losses to follow up: 0) | 18-33 (28) | 8F, 12M | Class I dental protrusion | The treatment used Roth prescription brackets (0.022 × 0.028-inch) and double buccal tubes on the second molars. Teeth were leveled and aligned prior to canine retraction, which began after placement of 0.019 × 0.025-inch SS archwires and used a sliding mechanism with a NiTi coil spring delivering a calibrated force of 200g for uniform retraction. Molar anchorage was reinforced with auxiliary hooks. | Four MOPs were performed distal to the canines: two buccal in the maxilla and two buccal in the mandible, using a screwdriver device and bony screws measuring 1.6 × 3.0mm. Perforations were 3mm deep, 1.6mm wide, spaced 3mm apart, and located 5mm from the alveolar crest, with no repetition at each interval. | Canine retraction,  Maxilla and Mandible | Models were digitized using a 3D scanner and were analyzed with Ortho Analyzer software, where tooth movement between phases was measured. Distances between the canine and second premolar were assessed in three areas: the center of the canine and premolar brackets, the canine cusp tip and premolar buccal cusp tip, and the shortest distance between the canine and premolar at the cervicogingival line. (Note: distances were not reported separately by jaw.) |
| Fattori et al. ,2020 | Brazil | maxillomandibular discrepancy indicating a need for orthognathic surgery, no previous dental extractions, the need for extraction of the upper or lower first premolars as part of the orthodontic treatment plan, good oral and general health, nonsmoker, no use of systemic corticosteroids or bisphosphonates. | parallel arm | N/A | 24 (losses to follow up: 6)  MOP: 12 (losses to follow up: 3)  Control:12 (losses to follow up: 3) | MOP: (27.8 ± 6.3)  Control: (20.4 ± 2.6) | 11F, 7M | maxillomandibular discrepancy indicating a need for orthognathic surgery | 10mm miniscrews were placed bilaterally between the first and second molars to provide anchorage. Anterior retraction was initiated 28 days after placement of 0.019 × 0.025-inch SS archwires. A 9mm NiTi closed coil spring was applied from the miniscrew to a hook distal to the canines, delivering 200g of force on each side. Implant-assisted sliding mechanics were used with self-ligating brackets. | Three buccal MOPs were performed vertically in the space midway between the canine and second premolar, 6mm deep and spaced 4mm apart; the width of the perforations was not reported, and the site was 4mm from the alveolar crest. The procedure was repeated every 28 days at each activation session for an average of 7 sessions. | Alignment, Maxilla and Mandible | A pre-retraction model was made 28 days post-archwire placement and digitized using XCAD 3D Scanner and Maestro 3D. Extraction space was measured linearly in 3D (Q3DC tool) between the canine’s most distal point and second premolar’s most mesial point. |
| Farag et al. ,2021 | Egypt | healthy periodontal condition, full unit class II canine relation, severe crowding or protrusion requiring maxillary first premolar extraction, normal canine and molar structures, no history of dental fillings or root canal treatment. | split mouth | > 3 months | 20 (preferred arm: 10)  MOP:10  Control:10 | 15-25 | N/A | Class I and Class II malocclusions | Fixed orthodontic appliances (MBT, 0.022-inch slot) were used to initiate the leveling and alignment phase, which was deemed complete when a 0.017 × 0.025-inch SS archwire fit passively into the bracket slots, indicating proper alignment. Following this, canine retraction was initiated on the control side using a mini screw for direct anchorage. A closed coil spring applied a consistent retraction force of 150g, measured precisely with a Coprex force gauge. | Twelve MOPs (6 buccal and 6 palatal) were performed using mini-screws measuring 1.6 × 8mm to achieve a perforation depth of 6mm, accounting for an average gingival thickness of 2mm; perforations were placed between the canine and lateral incisor roots and between the canine root and the socket of the extracted premolar on both buccal and palatal sides. MOPs were repeated every 2 weeks per session. | Canine retraction,  Maxilla | The measurements were taken from the canine cusp tip to the mesiobuccal cusp tip of the maxillary first molar using digital intraoral caliper which were taken immediately before the beginning of canine retraction and every 2 weeks along the following 3 months. |
| El Awady et al. ,2019 | Egypt | Inclusion:  age: 14-18, class II division 1 malocclusion or class I bimaxillary protrusion, mild or no crowding, required maxillary first premolar extractions.  Exclusion:  long-term systemic medication use, systemic diseases, any conditions affecting the rate of tooth movement. | split mouth | 6 months | 12 (losses to follow up: 0) | 13-19 (16.17 ± 2.29) | 8F, 4M | Class II division 1 malocclusion, Class I bimaxillary protrusion | All patients were treated with 0.022 × 0.028-inch slot Roth preadjusted edgewise metallic brackets. Anchorage was reinforced using a transpalatal arch connecting the first and second molars, with an additional 0.9mm SS wire soldered to the palatal side of the arch. Upper arches were leveled and aligned using NiTi archwires. The final working wire was a 0.016 × 0.022-inch SS archwire, placed for at least 3 weeks. Following MOP application, canine retraction began simultaneously on both sides using NiTi closed coil springs delivering 150g of force stretched between the hooks on the buccal surface of the first molar bands and the canine brackets, checked with a force gauge at 4-week intervals. | Three vertical buccal MOPs were performed, using miniscrews (1.6 × 8mm)to create perforations 4mm deep and 1.6mm wide; the first MOP was 6mm from the free gingival margin, with subsequent points spaced 5mm apart, positioned equidistantly between the canine and second premolar in the extraction space. The procedure was not repeated at each interval. | Canine retraction,  Maxilla | The measurement was based on assessing the bilateral distance between the distal contact points of the canines and the mesial contact points of the second premolars, using a digital caliper. The rate of canine retraction (mm/month) was calculated by dividing the total amount of retraction (in millimeters) by the total duration of retraction (in months). |
| Bansal et al. ,2019 | India | Inclusion:  4-6mm mandibular anterior irregularity index, age: 14-19, non extraction treatment in the mandibular arch, presence of full complement of dentition from first molar to first molar with no spacing in the mandibular arch, patient with healthy periodontium and no attachment loss more than 2mm.  Exclusion:  inability to place bracket or breakage of any of the mandibular anterior brackets that had not been replaced within a week, failure to provide oral and written consent to participation, previous orthodontic treatment, presence of primary or missing permanent teeth in the mandibular anterior area medical problems that affect tooth movement. | parallel arm | 15 + B (until alignment of mandibular anterior teeth) weeks  B MOP: 7.40 ± 1.55 weeks  B Control: 13.20 ± 1.52 weeks | 30 (losses to follow up: 0) | 14-19 (15.6 ± 1.476)  MOP: (15.87 ± 1.727)  Control: (15.33 ± 1.175) | 16F, 14M  MOP: 8F, 7M  Control: 8F, 7M | Mandibular anterior crowding | The orthodontic treatment began with the leveling phase using a 0.014-inch NiTi wire secured with 0.010-inch steel ligature wires. For the MOP group, this wire was ligated during the MOP procedure appointment, while for the control group, it was ligated during the bonding appointment. Both groups followed the same archwire sequence: 0.014-inch NiTi, 0.016-inch NiTi, 0.016 × 0.022-inch NiTi, 0.017 × 0.025-inch NiTi wires, and 0.019 × 0.025-inch SS wire. Archwires were replaced as soon as the desired improvement was achieved. The process was completed once the 0.019 × 0.025-inch SS wire fit passively. | Six buccal MOPs were performed on the labial aspect of the mandible at three sites: between the mandibular canine and lateral incisor on both sides and between the central incisors in the midline. A 1.6 × 8mm self-drilling orthodontic mini-implant and an MI screwdriver were used for the procedure. At each site, two vertically aligned perforations were made: one 2mm apical to the alveolar crest and the other 2mm below it, with depths ranging from 3-5mm, a width of 1.6mm, and a 2mm distance between the perforations. The procedure was not repeated at each interval. | Alignment,  Mandible | Little’s Irregularity Index (LII) was measured at each visit using a digital caliper (0.03 mm accuracy). Treatment completion required LII ≤ 1mm with < 0.5mm change between consecutive visits. |
| Al-Hayek et al. ,2019 | Egypt | Incusion:  adult female aged: 18-25, no previous orthodontic treatment, presence of permanent dentition from first molar to first molar, 4-10mm mandibular anterior irregularity index, no spaces in the mandibular arch, the lower incisor mandibular plane < 98˚, requiring non-extraction orthodontic treatment in the mandible, treatment plan did not require any therapeutic interventions involving intermaxillary or other intraoral or extraoral appliances (including elastics, lip bumpers, maxillary expansion appliances, or headgear) was needed prior to the complete alignment of mandibular anterior teeth.  Exclusion:  medical problems that affect tooth movement, presence of primary teeth, inability to place brackets on the mandibular anterior teeth (100% deep bite, blocked or severely rotated teeth), pregnancy | parallel arm | 6 months | 26 (losses to follow up: 5)  MOP:13 (losses to follow up: 2)  Control:13 (losses to follow up: 3) | 18-25 | 21F, 0M | Mandibular anterior crowding | For this study, the mandibular teeth, from the right first molar to the left first molar, were bonded using 0.022-inch conventional minidiamond brackets with a Roth prescription, with bands or tubes placed on the first molars. In both groups, the archwire progression was: 0.014-inch Cu-NiTi, 0.016-inch Cu-NiTi, 0.016 × 0.022-inch NiTi, and no final working wire for retraction was mentioned. The principle for changing the wires was: first to second (50% relief of the initial crowding), second to third (80% relief of the initial crowding) | Twenty-one buccal MOPs were performed across seven areas (left and right sides combined), with three linear MOPs at each site: mesial to the mandibular central incisors, distal to the mandibular central incisors, distal to the mandibular lateral incisors, and distal to the mandibular canines. The procedure was not repeated at each interval. | Alignment,  Mandible | Little’s Irregularity Index (LII) was measured on digital casts at each visit until LII = 0mm. Additionally, an “alignment improvement percentage” was presented at each time point. |
| Attri et al. ,2018 | India | Inclusion:  permanent dentition age: 13-20, Irregularity index of < 5mm, requiring extraction of mandibular and maxillary first premolars, periodontally sound dentition, having sound general health, Frankfurt mandibular plane angle (FMA): 20°-25°  Exclusion:  requiring orthognathic surgery, history of systemic and medical illness, periodontally compromised dentition, congenital orofacial deformities, previous history of orthodontic treatmen, Irregularity index > 5mm, pre-existing psychiatric or undergoing psychiatric therapy. | parallel arm | Until space closure was completed | 60 (losses to follow up: 0) | 13-20  MOP: (17.5 ± 2.52)  Control: (18.16 ± 1.48) | 33F, 27M  MOP: 18F, 12M  Control: 15F, 15M | N/A | Patients were treated using 0.022 × 0.028-inch slot brackets with the MBT prescription. The leveling and alignment phase followed the MBT archwire sequence, progressing until the working archwire, a 0.019 × 0.025-inch SS wire which was left passive for three weeks before retraction. Space closure was performed using active tie-backs in line with the MBT philosophy. Consistent force of 150g was ensured on each side using a Dontrix gauge. Retraction began immediately after performing MOPs. Conventional anchorage was established by banding the second molars and providing cross-arch stabilization with a trans-palatal arch. | Three buccal MOPs were performed in the extraction space at equal distances from the canine and the second premolar in the cortical bone using the ‘Propel’ device; the perforations were 2–3mm deep and 1.5mm wide. The procedure was repeated every 28 days until space closure was completed. | Canine retraction,  Maxilla and Mandible | The extraction space was measured digitally by constructing a mid-palatine reference line and perpendicular measurement lines from the canine's distal surface to the second premolar's mesial surface. All measurements were performed on digital models obtained by 3D scanning plaster casts using a White Light Scanner with reverse modeling software. |
| Babanouri et al. ,2020 | Iran | Inclusion:  age: 15-45, bilateral class II division 1 malocclusions or class I malocclusion with bimaxillary protrusion; mild or no crowding, no previous orthodontic treatment, no history of systemic disease, no radiographic evidence of bone loss in both sides of the mouth, no history of periodontal therapy or current periodontal disease, probing depth < 4mm across the entire dentition, no gingivitis or active carious lesion, gingival index and plaque index value < 1, and being a non-smoker.  Exclusion:  vertical skeletal discrepancy (62% ˂ Jarabak index ˂ 65% and 22˚ ˂ Frankfort mandibular plane angle < 28˚), systemic disease or medications that affect bone biology, poor oral hygiene. | split mouth | 84 days | 28 (losses to follow up: 3)  MOP1 (3 MOPs):14 (losses to follow up: 2)  MOP2 (6 MOPs):14 (loss to follow up: 1)  Control:25 | 16.3 - 35.2  MOP1: (26.08 ± 9.15)  MOP2: (25.31 ± 9.03)  P-value: 0.83 | 14F, 11M  MOP1: 7F, 5M  MOP2: 7F, 6M | bilateral class II division 1, class I malocclusion with bimaxillary protrusion | leveling and alignment phase used Mini Master Brackets (MBT, 0.022-inch slot). Archwire progression followed a planned 4-week sequence of NiTi wires, 0.014-inch NiTi, 0.016-inch NiTi, 0.018 SS, 0.016 × 0.022 SS. A 0.019 × 0.025-inch SS power arm was bonded mesial to the canine bracket to control rotation. One month prior to retraction, 1.6mm mini-screws were placed bilaterally in the buccal alveolar process and passively tied to the first molars to provide anchorage. Retraction was initiated using a 150g NiTi closed coil spring, connecting the TAD to the canine power arm. | In the MOP1 group, three buccal MOPs, and in the MOP2 group, six MOPs (three buccal and three palatal) were created between the distal of the canine and the mesial of the second premolar at the extraction site in the vertical direction, spaced 3mm apart. The first MOP was located 5mm from the free gingival margin. Perforations were made using 1.2mm diameter orthodontic mini-screws, which were inserted 1mm deep into the cortical bone. The perforation width and depth were 1.2mm and 1mm, respectively. The procedure was not repeated at each interval. | Canine retraction,  Maxilla | Measurements were taken on plaster models using a digital caliper with 0.01mm accuracy. Alginate impressions were made at baseline and at three subsequent intervals. Vertical reference lines were drawn on the palatal surfaces of the canine and lateral incisor, and distances were measured at the incisal, middle, and cervical thirds of the crowns at each time point. |
| Mahmoudi et al. ,2016 | United States of America | Incusion:  healthy adult patients, age: 18-44, minor to moderate crowding ≤ 6mm, orthodontic treatment plan requiring removal of bilateral first maxillary premolars.  Exclusion:  medical history of immune disorders or radiation therapy, severe crowding > 6mm, current active periodontal disease, patients who require prophylactic antibiotics. | split mouth | 3 months | 10 (losses to follow up: 0) | 18-37 (25.5) | 8F, 2M | N/A | Patients were bonded with 0.022-inch slot Unitek brackets with a metal slot and referred for extractions. Following the initial leveling and alignment phase, TADs (1.6 × 6mm) were placed bilaterally in the buccal region, mesial to the maxillary first molars. Stabilization was achieved by connecting the molars to the TADs with a 0.016 × 0.022-inch SS wire. Space closure was performed on a 0.017 × 0.025-inch SS archwire. Canine retraction was carried out using NiTi coil springs, which delivered 150g of force bilaterally, with the springs attached from the canine bracket hook to the first molar. | Three manual buccal MOPs were performed distal to the maxillary canines using Propel, creating perforations approximately 5mm deep and 0.25mm in diameter. The procedure was not repeated at each interval. | Canine retraction,  Maxilla | Cephalometric measurements were obtained from initial and final CBCTs using Anatomage Invivo5 software, including TAD-canine distal curvature (TAD-U3D) and second premolar mesial curvature-canine distal curvature (U5M-U3D) distances, with the TAD as a stable reference point. Digital models (T0, T2) were created using OrthoCAD, with bilateral measurements of (1) canine-premolar cusp tips (U3-U5), (2) extraction space width (U5M-U3D), (3) lateral incisor midpoint-canine cusp tip distance (U2-U3), and (4) first molar mesiopalatal-canine cusp tips distance (U6-U3). All analyses were performed in OrthoCAD. |
| GOWDA S V et al. ,2019 | India | Inclusion:  requiring orthodontic treatment with extraction of maxillary first premolars, age: 14-35, both genders, good general health, fully erupted maxillary teeth up to second molars.  Exclusion:  long-term use of antibiotics, phenytoin, cyclosporin, anti-inflammatory drugs, systemic corticosteroids, and calcium channel blockers, compromised periodontium, systemic diseases affecting periodontal status, evidence of alveolar bone loss. | parallel arm | 4 weeks | 46 (losses to follow up: 0) | 14-31  MOP: (19.8 ± 3.8)  Control: (20.7 ± 3.7) | 32F, 14M  Mop: 15F, 8M  Control: 17F, 6M | N/A | Treatment was initiated using fixed pre-adjusted edgewise appliances with a 0.022-inch slot MBT prescription in both arches. To enhance anchorage, trans palatal arches were placed in the maxillary arch. The leveling and alignment phase followed a standard sequence of archwires, concluding with a working archwire of 0.019 × 0.025-inch SS, which were left in place for one month before initiating retraction. Canine retraction was performed using NiTi closing-coil springs delivering 100g of force bilaterally. The springs were attached to facilitate controlled movement of the canines. | Three buccal MOPs were performed within the extraction space, equidistant from the canine and second premolar, using 1.5 × 8mm miniscrews and a hand driver. The depth of the MOPs was 3mm. The perforation width was 1.5mm. The procedure was not repeated at each interval. | Canine retraction,  Maxilla | Vertical reference lines were marked on the palatal surfaces of canines and lateral incisors (from incisal edge midpoint to cervical line midpoint). Inter-tooth distances were measured at incisal, middle, and cervical crown levels using a digital caliper (0.01 mm accuracy) before and after retraction. |
| RASHEED. V et al. ,2018 | India | requiring active fixed orthodontic appliance therapy, age: 16-30, class II division I malocclusion with treatment plan of extraction of both maxillary first premolars, healthy periodontal tissues with generalized probing depth ≤ 2 mm with minimal bleeding, patients who have signed the informed consent.  Exclusion:  long-term use of antibiotics, phenytoin, cyclosporine, anti-inflammatory drugs, systemic corticosteroids, and calcium channel blockers, systemic disease, radiographic evidence of bone loss, previous orthodontic treatment. | split mouth | 114.5 ± 9.5 days | 10 (losses to follow up: 0) | 16-30 | 5F, 5M | class II division 1 that required bilateral maxillary first premolars extraction | Fixed orthodontic appliances were placed, with brackets (0.022-inch slot MBT) bonded to both arches. Leveling and alignment were achieved through a sequential progression of NiTi archwires (0.014-inch NiTi, 0.016-inch NiTi, 0.017×0.025-inch NiTi, 0.019×0.025-inch NiTi). After alignment was completed, a 0.019 × 0.025-inch SS wire was placed and left passive for four weeks. Mini implants (1.2 × 8mm) were placed bilaterally in the buccal region between the second premolar and the first molar. Retraction force of 150g was applied using 6mm NiTi closed coil springs. | Three vertical buccal MOPs were performed distal to the canine using 1.2 × 8mm mini implants, spaced 3mm apart. The mini implants were inserted 6mm to achieve the desired MOP depth of 6mm. The perforation width was 1.2mm. The procedure was repeated after 2 months with the same pattern. | Canine retraction,  Maxilla | Vertical lines were drawn along the long axes of the canine and lateral incisor on dental casts to measure the distance between these lines at the incisal edge. Measurements were taken at time points T0, 45 days, and at completion (~114 days). All measurements were made using a digital Vernier caliper. |
| Alfailany et al. ,2023 | Syria | Inclusion:  age: 18-28, skeletal class II malocclusion (skeletal relationship in the sagittal plane (ANB: 5-9˚)), mild to moderate crowding of upper anterior teeth (2-6 mm), normal or vertical growth pattern (which was assessed radiographically), 5-10mm of overjet and 1-4mm of the overbite, the presence of a complete upper permanent dentition (except third molars).  Exclusion:  contraindications to anesthesia and surgical intervention, previous orthodontic treatment, poor oral hygiene, presence of periodontal diseases which was assessed clinically (probing depth > 4mm, plaque and gingival index >1). | parallel arm | Till the end of the canine retraction phase | 51 (losses to follow up: 0) | 18-27 (20.98 ± 1.95)  TC: (21.23 ± 2.33)  FCAPs (MOP): (21.09 ± 2.06)  Control: (20.62 ± 1.48)  P-value: 0.647 | 29F, 22M  TC: 11F, 6M  FCAPs (MOP): 8F, 9M  Control: 10F, 7M  P-value: 0.571 | skeletal class II division 1 required bilateral maxillary first premolars extraction | Patients were bonded with fixed appliances using the MBT system with 0.022-inch bracket slots. Leveling and alignment followed a standard archwire sequence, concluding with a working archwire of 0.019 × 0.025-inch SS. Moderate anchorage was achieved with soldered transpalatal arches placed at the start of treatment. Canine retraction commenced three days after flap corticotomy-assisted procedures (FCAPs) on the basal archwire, utilizing NiTi closed coil springs (NT3) stretched from the canine bracket hook to the first molar band, delivering 150g of force per side. | Eleven MOPs were performed using mini-implant drills (0.9 × 4 × 22mm) on a contra-angle handpiece. The perforations included six buccal (five distal and one mesial to the canine) and five palatal (distal to the canine). The perforation depth was 4mm, and the width was 0.9mm. Distobuccal perforations were spaced 1.5-2mm apart. Sites of perforation were between the canine and second premolar (distobuccal), between the canine and lateral incisor 4mm from the papilla (mesiobuccal), and between the canine and second premolar on the palatal side. The procedure was not repeated at each interval. | Canine retraction,  Maxilla | Dental casts were obtained at five time points. Measurements were performed manually using a digital caliper to assess:  1) The anteroposterior movement of the canine by measuring the distance between the medial ending of the third palatal rugae and the tip of the canine cusp tip.  2) The anteroposterior movement of the first molar by measuring the distance between the medial ending of the third palatal rugae and the central fossa of the first molar.  3) Canine rotation by measuring the angle between the mid-palatal suture and the line through the mesial and distal edges of the canine. |
| Rashid et al. ,2018 | India | Inclusion:  Age: 18-30 and/or who have completed their growth, Angle’s class I malocclusion with either bidental protrusion or angle’s class II, division 1 malocclusion with minimal crowding who require maxillary first premolars extraction and have completed levelling and alignment, average growth pattern, no systemic, bone/metabolic disorders.  Exclusion:  previous history of orthodontic treatment, class II division I malocclusion with extreme skeletal class II malocclusion, overjet > 10mm and indicated for orthognathic surgery, severe vertical growers long term use of analgesics, phenytoin, cyclosporine, anti inflammatory drugs, systemic corticosteroids, and calcium channel blockers, thin gingival biotype, history of extraction of any permanent teeth anterior to the 3rd molars, poor periodontal status (radiographic evidence of bone loss). | parallel arm | 6 months ( till closing of the extraction space from initial month of retraction) | 20 (losses to follow up: 0) | 18-30 | N/A | Class I malocclusion with either bidental protrusion or Class II, division 1 malocclusion with minimal crowding who require maxillary first premolar extraction | Following the completion of leveling and alignment, a 0.019 × 0.025-inch SS archwire was left in place for six weeks. Prior to the retraction phase, the maxillary anterior teeth were secured using a 0.010-inch SS ligature wire. En-masse retraction was performed on a 0.019 × 0.025-inch SS archwire utilizing active tie-backs, with a standardized force of 150g measured using a Dontrix gauge. Follow up sessions, every four weeks for activations and force consistency verification. | Three buccal MOPs were performed gingival to the extraction site distal to the canines at the coronal, middle, and apical regions using Surgicals Microimplants (1.5 × 8.0mm). Each perforation was 1.5mm wide and 3mm deep. The procedure was not repeated at each interval. | Canine retraction,  Maxilla | The canine-premolar distance was measured to 0.1mm precision using a sliding caliper, and the retraction rate was calculated as distance divided by time. |
| Aboalnaga et al. ,2019 | Egypt | Inclusion:  adult females aged: 16-30 requiring bilateral extraction of the maxillary first premolars and canine retraction with maximum anchorage, full permanent dentition with exception of the third molars, good oral hygiene, good periodontal condition.  Exclusion:  medically compromised patients, chronic use of medications affecting tooth movement, smoking, any radiographic evidence of bone loss. | split mouth | 4 months | 18 (losses to follow up: 0) | 16-25  (20.5 ± 3.85) | 18F, 0M | N/A | Subjects received straight wire appliances with Roth prescription, using 0.022-inch slot brackets on both arches. After initial leveling, self-drilling TADs (1.8 × 8mm) were placed buccally between upper second premolars and first molars bilaterally. Indirect anchorage was established with L-shaped 0.019 × 0.025-inch SS wires secured to TADs. A rigid 0.017 × 0.025-inch SS retraction archwire was placed, and canine retraction commenced using NiTi closing coil springs, applying a consistent force of 150g from the first molar to the canine hook. | Three vertical buccal MOPs were performed at equal distances from the canine and second premolar in the extraction space using an L-shaped wire guide to mark the sites. TADs (1.8 × 8mm) were inserted perpendicularly into the alveolar bone. Each perforation was 8mm deep and 1.8mm wide. The procedure was not repeated at each interval. | Canine retraction,  Maxilla | Stone models were scanned with a 3Shape R900 scanner and digitized. The T0 model was superimposed on pre-retraction CBCT using three occlusal points to establish a frontal reference plane (FP), while subsequent models (T1-T4) were aligned to T0 using third rugae points. Using 3Shape Analyzer, monthly canine retraction was measured as the sagittal distance from the canine's cusp tip/center/root apex to FP, while molar anchorage loss was measured from the first molar's mesiobuccal cusp tip/center/root apex to FP. Total 3D displacement was calculated from pre and post-retraction CBCTs using Invivo 5. |
| Alikhani et al. ,2013 | United States of America | Inclusion:  both genders, age: 18-45, class II division 1 malocclusion, no systemic disease, no radiographic evidence of bone loss, no history of periodontal therapy, no smoking, no gingivitis or untreated caries, probing depth < 4mm in all teeth, gingival index ≤ 1, plaque index ≤ 1.  Exclusion:  long-term use of antibiotics, phenytoin, cyclosporin, anti-inflammatory drugs, systemic corticosteroids, and calcium channel blockers, poor oral hygiene for more than 2 visits, extreme skeletal class II malocclusion, overjet > 10mm, Pg-Nper > 18mm, ANB > 7˚, SN-GoGn > 38˚, systemic disease, evidence of bone loss, past periodontal disease, current periodontal disease, smoking, gingivitis and caries, probing depth > 4mm in any tooth, patients with crossbite or deviation during closure caused by occlusal interference. | parallel arm | 28 days | 20 (losses to follow up: 0) | 19.5 - 33.1  MOP: (26.8)  Control: (24.7) | 12F, 8M  MOP: 5F, 5M  Control: 7F, 3M | Class II division 1 malocclusion that required bilateral maxillary first premolars extraction | Treatment commenced with bonding fixed appliances in both arches using 0.022-inch MBT prescription brackets with auxiliary vertical slots in maxillary canine brackets. Canine retraction utilized calibrated NiTi closing coil springs (100g force) attached from a TAD to a power arm on the canine bracket for precise force application. Six months into treatment, a self-drilling TAD was placed 5mm apical to the alveolar crest between the second premolar and first molar, and loaded immediately. | Three buccal MOPs were performed distal to the canines before retraction using a disposable MOP device from PROPEL Orthodontics. Each perforation was 2-3mm deep and 1.5mm wide. The procedure was not repeated at each interval. | Canine retraction,  Maxilla | Vertical lines were drawn on the palatal surfaces of the canine and lateral incisor on dental casts, extending from the middle of the incisal edge to the middle of the cervical line to divide the crowns into equal halves. Tooth movement was assessed by measuring the distance between these two vertical lines at three locations: incisal, middle, and cervical thirds of the crowns. All measurements were performed on casts using an electronic digital caliper (with 0.01 mm accuracy). |
| Alqadasi et al. ,2019 | China | Inclusion:  age: 15-40, both genders, class II division 1 malocclusion who required the extraction of both maxillary first premolars with full erupted canine, good oral hygiene, adequate attached gingiva thickness (1–2mm), no periodontal disease.  Exclusion:  history of previous orthodontic treatment, systemic disease (which effects bone cycle), history of periodontal disease treatment, radiographic bone loss, smoking. | split mouth | 3 months | 8 (losses to follow up: 0) | 15-40 | N/A | Class II division 1 malocclusion that required bilateral maxillary first premolars extraction | Fixed orthodontic appliances with the MBT self-ligating bracket system were utilized, with maximum anchorage provided by miniscrews placed in the buccal region. Direct anchorage forces were applied from miniscrews to the canines, while indirect anchorage was achieved by ligating the second premolars to the miniscrews.. Calibrated NiTi closing coil springs delivering 150g of force were attached from the TADs to the power arms on the maxillary canine brackets. | Three buccal MOPs were made in the middle of the extraction space using automated mini-implant instrumentation. The perforations were 5-7mm deep and 1.5-2mm wide. The procedure was not repeated at each interval. | Canine retraction,  Maxilla | Dental cast models were scanned using a 3Shape scanner. Measurements were conducted with Geomagic software. a midline was drawn, and perpendicular lines were extended from the buccal cusp tips of the canines and second premolars on both sides. The distance from the canine cusp tip to the perpendicular line drawn from the second premolar cusp tip was used to measure tooth movement. |
| Kundi et al. ,2018 | Saudi Arabia | Inclusion:  adult patients aged: 20-40, class II division 1 malocclusion, maxillary bilateral premolar extractions indicated, good oral hygiene.  Exclusion:  previous orthodontic treatment, long-term use of drugs, and systemic diseases. | parallel arm | 12 months  Evaluation: 28days | 28 (losses to follow up: 0)  MOP:28  Control:28 | 20-40  MOP: (28.4 ± 4.2)  Control: (26.4 ± 4.1) | 16F, 12M | Class II division 1 malocclusion | In both groups, canine retraction was initiated using NiTi coil springs, applying an approximate force of 100g. | Three buccal MOPs were performed distal to the canines using a disposable MOP device (PROPEL) with a diameter of 1.5mm. The perforation depth and distance between perforations were not reported. The procedure was not repeated at each interval. | Canine retraction,  Maxilla | Measurements were performed directly on unsoaped plaster casts using an electronic digital Vernier caliper with sharpened tips. The canine retraction was quantified by two measurements: Tip distance: between the canine cusp tip and lateral incisor's incisal edge midpoint, and Cervical distance: between the cervical midpoints on the height of contour at both teeth's cinguli. |
| Venkatachalapathy et al. ,2022 | India | Inclusion:  permanent dentition aged: 15-25, class I bimaxillary protrusion, fully erupted maxillary canine with closed apex, cases requiring extraction of both maxillary and mandibular first premolars, periodontally sound dentition, sound general health.  Exclusion:  long-term use of antibiotics, phenytoin, cyclosporine, anti inflammatory drugs, systemic corticosteroids, and calcium channel blockers, Skeletal class II tendency and ANB >2°, Skeletal class III tendency and ANB < 2°, cases requiring orthognathic surgery, history of systemic and medical illness, contraindication of extraction, previous history of orthodontic treatment, poor oral hygiene, smoking, nickel allergy. | split mouth | 84 days | 20 (losses to follow up: 0) | 15-25 | N/A | Class I bimaxillary protrusion | Fixed appliances were bonded in both arches using the MBT 0.022-inch prescription brackets, with auxiliary vertical slots in the canine brackets. Leveling and alignment were achieved through a sequential progression of 0.016-inch NiTi, 0.017 × 0.025-inch NiTi, and 0.019 × 0.025-inch NiTi wires. Following this, a 0.019 × 0.025-inch SS archwire was placed for four weeks. During the retraction phase, a 0.019 × 0.025-inch SS archwire was used in both arches. Retraction force was applied using a serpentine hook (0.016 × 0.016-inch SS wire), which was inserted into the auxiliary vertical slot of the canine bracket. Buccally, a TAD (1.5 × 9mm) was placed between the second premolar and first molar, positioned 6mm from the interdental papilla. | Five buccal MOPs were performed using a hand-held TAD (1.5 × 9mm) loaded in an implant driver with a rubber stopper to ensure each perforation was 3mm deep and 1.5mm wide; two perforations were placed distal to the canine at heights of 7mm and 12mm from the alveolar crest, and three were placed in the middle of the extraction socket at 5mm, 10mm, and 15mm from the crest (5mm apart), repeated every 28 days (at day 28 and day 56). | Canine retraction,  Maxilla and Mandible | Vertical reference lines were drawn on the palatal canine surface from the cervical line midpoint. Canine-lateral incisor distances were measured at incisal, middle, and cervical crown thirds pre- and post-retraction using a digital caliper (0.01mm accuracy). |
| RAGHAV et al. ,2020 | India | Inclusion:  both genders, age: 17-30, angles’ class I bimaxillary, class II div 1 malocclusion (ANB < 5º), no systemic disease, no radiographic evidence of bone loss, no history of periodontal therapy, no smoking, no gingivitis or untreated caries, gingival index <1, plaque index <1  Exclusion:  long term use of antibiotics, steroids, anti-inflammatory drugs, Prominent canine roots (in labial cortical bone), extreme skeletal class II malocclusion (ANB > 5º). | split mouth | 16 weeks | 30 (losses to follow up: 2) | 17-30  (20.26 ± 2.13) | 18F, 12M | Class I bimaxillary, Class II div 1 malocclusion (ANB < 5º) | All subjects were bonded with a 0.022 × 0.028-inch MBT prescription appliance. Leveling and alignment were achieved with NiTi archwires, leading to the placement of a 0.019 × 0.025-inch SS archwire, which was retained passively for four weeks. SS ligature wires were loosely tied to minimize friction. Canine retraction was performed using the 0.019 × 0.025-inch SS wire and NiTi closed coil springs (150g force), attached from the canine power arm to the molar tube hook. | Three buccal MOPs were performed distal to the canine root using a Lance Pilot Drill, with each perforation measuring 5mm in depth and 2mm in width. The procedure was not repeated at each interval. | Canine retraction,  Maxilla and Mandible | Alginate impressions were taken at baseline and subsequent intervals to creat study models. An acrylic palatal plug with 0.9mm SS reference wires was fabricated on the baseline model. Canine retraction was measured by transferring this plug to subsequent models and recording the displacement from the canine long-axis midpoint to the reference wire using a digital caliper (0.01mm accuracy), which was carefully aligned parallel to both. Force levels were monitored using a Dontrix gauge. |
| Singh et al. ,2023 | India | Inclusion:  age: 18-30, angle’s class II division 1 malocclusion-or class I bimaxillary protrusion.  Exclusion:  history of systemic or periodontal disease, gingivitis or active caries, smoking, or undergoing any long‑term medications, previous orthodontic treatment, craniofacial anomalies, pregnancy. | split mouth | 56 days | 22 (losses to follow up: 2)  MOP1 (3 MOPs):11 (loss to follow up: 1)  MOP2 (6 MOPs):11 (loss to follow up: 1)  Control:20 | 18-30 | N/A | Class II div 1 malocclusion, Class I Bimaxillary protrusion | Before MOPs, all subjects were bonded with pre-adjusted edgewise appliances following the MBT prescription with a 0.022-inch slot. After extracting the maxillary first premolars and completing initial alignment, segmental retraction of the maxillary canines began using a 0.019 × 0.025-inch SS archwire, retained passively for four weeks. A power arm made from 0.019 × 0.025-inch SS wire was bonded mesial to the canine bracket. After the MOP procedure, bilateral canine retraction was performed using pre-calibrated NiTi closed coil springs delivering 150g of force, which were attached from a TAD (1.5 × 8mm) placed between the maxillary second premolar and first molar. | MOPs were performed using a TAD (1.5 × 8mm) with a rubber stopper standardizing the perforation depth at 6mm and width at 1.5mm. In the MOP1 group, three buccal perforations were made, while in the MOP2 group, three buccal and three palatal perforations were created. All perforations were placed distal to the canine, within the extraction site, vertically aligned, spaced 3mm apart, with the first perforation positioned 5mm from the free gingival margin. The procedure was not repeated at any interval. | Canine retraction,  Mandible | On dental casts at three time points vertical reference lines were drawn on the palatal surfaces of canines and lateral incisors, from the incisal edge to the cervical line midpoints. Using a digital caliper (0.01mm accuracy), inter-tooth distances were measured at three levels (incisal, middle, and cervical thirds). |
| Abdelhameed et al. ,2018 | Egypt | age: 15–25 of either sex, healthy general and periodontal conditions, malocclusion requiring the extraction of maxillary first premolars followed by canine retraction, normal shape and structure of the maxillary canine (no history of restorations or root canal treatment) and first molar. | split mouth | 3 months | 30 (preferred arm: 10 which has 1 loss to follow up at 2 intervals; T2 and T5; 4 and 10 weeks respectively)  MOP: 10 (loss to follow up only at T2 and T5: 1)  Control: 10 (loss to follow up only at T2 and T5: 1) | 15-25 | N/A | Dental full unit Class II canine relationship, bi‑maxillary protrusion | After the leveling and alignment phase, mini-screws were placed between the maxillary first molar and second premolar to provide direct anchorage. Canine retraction was initiated using calibrated closing coil springs delivering a consistent force of 150g, which was verified with a force gauge. | MOPs were performed using mini-screws (1.6 × 8mm), with an effective perforation depth of 6mm in the alveolar bone, accounting for a gingival thickness of 2mm. A total of 12 perforations were made per intervention session, 6 buccal and 6 palatal, placed between the canine and lateral incisor roots, as well as between the canine root and the socket of the extracted first premolar, on both buccal and palatal sides. MOPs were repeated every two weeks, totaling 6 sessions over 3 months. | Canine retraction,  Maxilla | Intraoral measurements were taken using a digital caliper to record the distance between the canine cusp tip and first molar mesiobuccal cusp tip. Measurements were performed at baseline and every 2 weeks for three months. |
| Ahsan et al. ,2021 | Pakistan | Inclusion:  both genders, age: 18-30, without gingivitis or periodontitis, no radiographic evidence of bone loss and systemic illness or using any type of medication like (anti-inflammatory drugs, corticosteroids,calcium channel blockers, immunosuppressant, long term antibiotic therapy), skeletal Class I & II as conformed by cephalometric radiographs, and non-smokers.  Exclusion:  skeletal class III malocclusion, probing depth > 4mm, dental cross bites, temporomandibular joint problems (TMJ ankylosis), facial asymmetry and deviation in closing due to occlusal interferences, restorations or untreated carious lesions. | split mouth | 6 months | 30 (losses to follow up: 0) | 18-30  (21.7 ± 2.98) | 13F, 17M | Skeletal Class I & II | Fixed orthodontic appliances were employed, utilizing 0.017 × 0.025-inch SS archwires as the working wire. Canine retraction was initiated following MOP using NiTi coil springs with a 6mm length. | MOPs were performed using a TAD screw (1.6mm diameter) to create three perforations on the buccal side of the right distal canine. Each perforation measured 3mm in depth and 1.6mm in width. The procedure was not repeated at any interval. | Canine retraction,  Maxilla and Mandible | Vernier calipers measured the second premolar-canine distance at incisal, middle, and cervical regions pre- and post-retraction (3-week interval). |
| Alqadasi et al. ,2024 | China | Inclusion:  age: 15-40, diagnosed with class II division 1 malocclusion, fully erupted maxillary canines, indicated for extraction of both the maxillary first premolars and maximum anchorage, and good oral hygiene.  Exclusion:  history of periodontal disease/treatment, smoking, systemic diseases affecting tooth movement or bone condition, history of previous orthodontic treatment. | split mouth | 3 months | 24 (preferred arm: 12 which has 2 losses to follow up)  MOP: 10  Control: 10 | 15-40  (20.89 ± 4.46) | 12F, 9M  MOP: 6F, 4M  Piezo: 6F, 5M | Class II division 1 | A fixed labial orthodontic appliance (MBT metal bracket, 0.022-inch slot) was utilized. Alignment and leveling were achieved through a sequential progression of archwires, culminating in the placement of a 0.018 × 0.025-inch SS archwire as the working wire. Self-drilling thread-type TADs were inserted bilaterally in the buccal region between the second premolars and first molars. Canine retraction was performed using calibrated NiTi closed coil springs with loop ends and a passive length of 6mm, delivering a consistent force of 150g. | Three buccal MOPs were performed using an automated mini-implant driver at equal distances from the canine and the second premolar within the extraction space. Each perforation measured approximately 5-7mm in depth and 1.5-2mm in diameter. The procedure was not repeated at any interval. | Canine retraction,  Maxilla | 3D digital models of teeth were captured at multiple time points. Using Ortho Analyzer software, they established a stable reference based on the midpalatine raphe (palatal midline). Reference lines were drawn from the premolar and canine teeth to this midline. The distance between these lines was measured at each time point. |
| Alkebsi et al. ,2018 | Jordan | Inclusion:  both genders, age ≥ 16, class II division 1 malocclusion, class II canine relationship, and average lower facial height (53%- 57%) and maxillomandibular plane angle (23°- 31°) (based on Eastman cephalometric standards).  Exclusion:  diseases and medications affecting bone biology, poor oral hygiene, low or high angle skeletal patterns, previous orthodontic treatment, evidence of bone loss, active periodontal disease, and smoking. | split mouth | 3 months | 35 (losses to follow up: 3) | 16-24.6  (19.26 ± 2.48) | 24F, 8M | Class II division 1 | Orthodontic treatment was conducted using fixed preadjusted edgewise appliances (0.022-inch MBT prescription). Miniscrews (1.5 × 8mm) were placed to provide stable anchorage. Leveling and alignment were completed using a sequential progression of archwires, culminating in a 0.019 × 0.025-inch SS archwire as the working wire. Canine retraction was performed using 9mm NiTi closed coil springs calibrated to deliver a consistent force of 150g. | MOPs were performed using miniscrews (1.5 × 6mm), utilizing a handpiece to create three buccal perforations distal to the canine tooth. Each perforation measured 3-4mm in depth and 1.5mm in width, spaced 3mm apart. The procedure was not repeated at any interval. | Canine retraction,  Maxilla | 3D digital models were superimposed at the rugae area from baseline to subsequent intervals using the Ceramill Map 400 scanner and Ceramill Mind design software. Canine displacement was measured on these superimposed models by assessing the middle projection of the distal surface of the maxillary canines. Additionally, intraoral measurements were taken using a digital caliper by measuring the distance from the upper mesial wing of the canine bracket to the upper distal wing of the second premolar bracket. |
| Hashem et al. ,2024 | Egypt | Inclusion:  age: 15-22 years with complete permanent dentition and malocclusion requiring extraction of maxillary first premolars.  Exclusion:  compromised periodontium, craniofacial anomalies, trauma history, bruxism, previous orthodontic treatment. | split mouth | 4 months | 18 (losses to follow up: 0)  MOP I (single): 9  MOP II (repeated): 9  Control: 18 | 15-22  MOP I (single): (16.78 ± 2.22)  MOP II (repeated): (17.56 ± 2.65) | N/A | N/A | Orthodontic treatment involved fixed appliances using Damon Q self-ligating metallic brackets with a 0.022-inch slot bonded from the right to the left maxillary second premolars, excluding the maxillary first premolars. Single buccal molar tubes with hooks and a 0.022-inch slot were bonded directly to the maxillary first molars. Leveling and alignment were completed before the canine retraction phase using a sequence of round NiTi archwires, 0.014-inch, 0.016-inch, and 0.018-inch, rectangular 0.016 × 0.022-inch NiTi wires. The final working wire, a 0.016 × 0.022-inch straight archwire, was left in place for three weeks prior to retraction. Mini-screws (1.6 × 8mm) were placed between the upper first molar and second premolar, positioned at the mucogingival junction which provided both direct and indirect anchorage. Prior to retraction, the upper incisors were stabilized with a figure-of-eight ligation using 0.009-inch wire, while the maxillary second premolars were ligated to the first molars with a 0.009-inch wire on each side. Canine retraction was initiated using NiTi closed coil springs attached from the mini-screw head to the hook of the maxillary canine bracket. The springs were secured in place using a 0.009-inch ligature wire fastened to the distal wing of the canine bracket. A 0.009-inch ligature wire was also used to tie the canine bracket to the archwire. The applied force was standardized to 150g and measured using a Correx tension gauge. Force measurements were conducted at the beginning of retraction and monthly follow-ups. | Three MOPs were performed distal to the maxillary canines, positioned halfway between the canine and the second premolar within the extraction space, each measuring 4mm in depth and 1.6mm in diameter, and repeated monthly for four months in Group II. | Canine retraction,  Maxilla | The primary outcome was 4 month canine distal movement measured using 3D digital models created from alginate impressions (baseline and 4 month) scanned with a Primescan (with a precision of 0.0021mm precision) and analyzed in Dolphin software where they were virtually positioned in a mutual position similar to the baseline scanned model, superimposing the baseline 3D digital model on the 4th month's 3D digital model. The most medial end of the third rugae area, which was perpendicular to the mid-palatal raphe line at T0, defined the rugae line. |
| Joseph et al. ,2022 | India | Inclusion:  age: 18-30, patients with bimaxillary dentoalveolar protrusion requiring first premolar extractions, good oral hygiene and no periodontal disease or radiographic evidence of bone loss.  Exclusion:  systemic diseases or craniofacial syndromes, previous extractions affecting bone at the site, alcoholics or smokers, use of medications interfering with bone metabolism (e.g., NSAIDs, doxycycline). | split mouth | 10 months | 11 (losses to follow up: 0) | 18-30  (19 ± 4.21) | 9F, 2M | Bimaxillary dentoalveolar protrusion | Fixed appliances using a preadjusted edgewise system with the MBT prescription (0.022-inch slot) were bonded. En masse retraction was initiated after 21 days of engaging working 0.019×0.025-inch SS archwires. NiTi coil springs delivering 150g of force per side were used for retraction, which were changed after two months. | Three MOPs were conducted in the extraction space of the first premolar, evenly spaced between the canine and second premolar. A 2 × 6mm mini-implant created perforations of 2-3mm depth and 1.5mm width, with no repetitions at any interval. | Alignment,  Maxilla and Mandible | A digital Vernier caliper measured the distance between the canine cusp tip and first molar's mesiobuccal cusp tip. |
| Kilinc and Baka ,2023 | Turkey | Inclusion:  no systemic disease, age ≥ 14 years, permanent dentition, mandibular anterior crowding > 5mm according to Little’s Irregularity Index, good oral hygiene.  Exclusion:  any systemic disease, missing, supernumerary, or impacted teeth (except mandibular third molars), smoking. | parallel arm | 16 weeks | 45 (losses to follow up:1)  Piezocision: 15  MOP: 15 (losses to follow up:1)  Control: 15 | ≥ 14  Piezocision: 14.40-21.10 (16.99 ± 1.73)  MOP: 15.18-22.42 (17.51 ± 2.79)  Control: 14.55-21.46 (17.39 ± 2.16)  p-value: 0.914 | 26F, 19M  Piezocision: 9F, 6M  MOP: 9F, 5M  Control: 7F, 8M  p-value: 0.603 | Mandibular anterior crowding | All patients were bonded using a fixed appliance system with Discovery Smart braces featuring a 0.022-inch slot MBT prescription. a 0.014-inch NiTi archwire was placed for leveling and alignment (at the same time with MOP procedure), and it remained in place for 8 weeks. After that a 0.016-inch NiTi archwire was replaced to continue leveling. | MOPs were performed vertically using a mini-implant system with a rubber stop for depth control and a disposable MOP device from Propel Orthodontics. A total of 9 buccal perforations were made on both sides of the mandible, starting 2mm apical to the alveolar crest. Each perforation measured 3mm deep and 1.5mm wide, spaced 1mm apart, located between the mandibular canines and lateral incisors, and between the central incisors. MOPs were not repeated at any interval. | Alignment,  Mandible | Little’s irregularity index measured from plaster models using digital caliper. LII measures the horizontal linear displacement of anatomic contact points of each mandibular incisor from the adjacent anatomic point and sums the five displacement together. Which represents the degree of anterior irregularity. |
| Bavikati et al. ,2022 | India | Inclusion:  female patients aged 18-25, requiring extraction of maxillary first premolars  Exclusion:  chronic medical conditions or medication, poor oral hygiene, smoking, bone loss visible on radiographs. | split mouth | 114.5 ± 9.5 days | 22 (losses to follow up: 0) | 18-25 | 22F, 0M | N/A | The orthodontic treatment utilized MBT prescription brackets with a 0.022-inch slot. After initial alignment with a 0.019 × 0.025-inch SS working wire, self-drilling TADs (1.8 × 8mm) were placed bilaterally between the upper second premolar and first molar. One month later, MOPs were performed before initiating canine retraction. NiTi closing coil springs were used to apply a standardized force of 150g between the TADs and canine hooks, measured with a Dontrix gauge. | Three vertical MOPs were conducted using a TAD (1.8 × 8mm), positioned distally to the canine with a 2mm gap between them. Each perforation was 5mm deep and 1.2mm wide, with no repetitions at any interval. | Canine retraction,  Maxilla | A digital Vernier caliper measured distances between vertical reference lines drawn along the long axes of canines and lateral incisors on dental casts. |
| Kumar et al. ,2024 | India | Inclusion:  Age: 18-35, skeletal class I with bimaxillary dentoalveolar protrusion, healthy periodontal status.  Exclusion:  systemic diseases, poor oral hygiene, smoking, certain medications, skeletal class II and III malocclusions. | parallel arm | 4 months | 20 (losses to follow up: 0) | 18-35  MOP: (19.5 ± 2.67)  Control: (20.3 ± 2.23) | 13F, 7M | Class I malocclusion | Fixed metal brackets with a slot size of 0.022 × 0.028-inch (MBT prescription) were bonded. Leveling and alignment followed the MBT archwire sequence up to a 0.019 × 0.025-inch SS working wire, which was left passively for one month before anterior retraction. For anchorage reinforcement, TADs (titanium miniscrews, 1.4 × 7mm) were inserted in the interradicular space between the first molars and second premolars at the mucogingival junction. | Three vertical MOPs were performed using FavAnchor (1.5 × 4mm) with a hand-held driver and single-use SS screws. MOPs were placed in the labial cortical plate along the long axis of the six anterior teeth in each interdental region and distal to the canines, spaced 5mm apart, starting 6mm from the free gingival margin. This totaled 21 buccal MOPs per arch at the beginning of space closure (T0), with the procedure repeated once at the first monthly follow-up (T1). | Alignment,  Maxilla and Mandible | Digital models created from scanned plaster casts; extraction space measurements performed digitally. |
| Al-Attar et al. ,2022 | Iraq | Inclusion:  required orthodontic treatment using upper and lower fixed appliances, moderate crowding in mandibular arch (Little's irregularity index 4-6 mm), required non-extraction treatment in mandibular arch, Full set of permanent dentitions excluding third molar, no history of root resorption or trauma to mandibular anterior teeth.  Exclusion:  previous orthodontic treatment, severely displaced teeth (couldn't be bonded), significant vertical skeletal discrepancies, poor oral hygiene, active periodontal disease, systemic disease affecting tooth movement. | parallel arm | Approximately 20 weeks  MOP: 10.41 (CI: 9.92-10.89) weeks  Control: 16.62 (CI: 16.11-17.13) | 35 (losses to follow up: 2)  MOP:19 (losses to follow up: 2)  Control:16 | 17-22  (18.97), (95% CI: 18.35-19.54) | 20F, 13M | Moderate mandibular anterior crowding | Treatment began with the placement of pre-adjusted fixed appliances featuring an MBT prescription with a 0.022-inch slot. The archwire sequence included: 0.014-inch NiTi, 0.018-inch NiTi, and 0.017 × 0.025-inch NiTi, culminating in a final working wire of 0.019 × 0.025-inch SS. The initial 0.014 inch NiTi archwire was placed on the bonding day and fully tied to all brackets using elastomeric modules. Archwires were replaced, based on achieved improvements, allowing for the next wire to be inserted with minimal deflection. The study focused primarily on the alignment phase of treatment. | Self-drilling mini-implants (1.6 × 6mm) were used for the MOPs procedure. Two buccal MOPs were performed vertically at each site: bilaterally between the mandibular canine and lateral incisor, and between the central incisors. The perforations were spaced 2mm apart, with the first positioned 2mm from the crest and standardized at 3mm depth using an endodontic rubber stopper. The procedure was not repeated at any interval. | Alignment,  Mandible | Little's irregularity index measured on digital 3D scanned models. LII measures the horizontal linear displacement of anatomic contact points of each mandibular incisor from the adjacent anatomic point and sums the five displacement together. Which represents the degree of anterior irregularity. |
| Gümüş et al. ,2023 | Turkey | Inclusion:  age > 16, average Frankfort-mandibular plane angle (20-25°), good oral hygiene (gingival and plaque index < 1), no active carious lesion, nonsmoker status.  Exclusion:  medications and diseases affecting bone metabolism, radiographic evidence of bone loss. | split mouth | 3 months | 20 (losses to follow up: 0) | 15.9 - 25.0  (16.5) | 17F, 3M | Class II division 1 malocclusion that required bilateral maxillary first premolars extraction | All participants were treated with preadjusted edgewise appliances featuring a 0.022-inch slot. The initial archwire sequence included: 0.014-inch, 0.016-inch, and 0.016 × 0.022-inch NiTi wires. Canine retraction commenced four months later, utilizing NiTi closed coil springs that applied a force of 120g. For direct anchorage, miniscrews (1.5 × 8mm) were placed between the maxillary second premolars and first molars. MOPs were performed at the time of coil spring placement. | Three vertical MOPs were performed using miniscrews (1.5 × 8mm) with a screwdriver distal to the canine in the center of the extraction region, which were 1.5mm wide, 3-4mm deep, determined by the alveolar bone and mucosa thickness. The perforations were spaced 3mm apart, with the first perforation positioned 5mm from the crest. MOPs were repeated every 28 days, for a total of three times. | Canine retraction,  Maxilla | The distance between the most distal point of the canine and the most mesial point of the second premolar was measured using 3D digital models. |
| Ozkan et al. ,2021 | Turkey | Inclusion:  class I or II malocclusion requiring upper first premolar extraction, Age > 16, good general health, no medication, no radiographic/clinical bone loss, good periodontal condition, healthy mental condition.  Exclusion:  class III malocclusion, Sagittal/transversal skeletal problems, Age < 16, cross bite, abnormal chewing habit, previous orthodontic treatment, long-term drug use, Smoking, periodontal hygiene problems, orofacial deformity, mental/behavioral disorders. | parallel arm | 28 days | 24 (losses to follow up: 0)  MOP I (4mm): 12  MOP II (7mm): 12  Control: 12 | 16-21  MOP: (17.27 ± 1.22)  Control: (18.13 ± 1.28) | 12F, 12M | Class I and Class II Division 1 | Fixed MBT appliances (0.022×0.028-inch slots) were placed. A 6-month leveling phase used sequential archwires: 0.014-inch, 0.016-inch, 0.019×0.025-inch HANT NiTi to address RAP effects and occlusal interferences. After 4 weeks with passive 0.019×0.025-inch SS archwires, TADs were placed between second premolars and first molars, and canine retraction initiated using 150g Sentalloy nitinol coil springs measured with a force gauge. | The MOP procedure involved creating three vertically aligned perforations per side, measuring 4mm and 7mm in depth, using a modified 1.6 × 8mm miniscrew equipped with depth-marking elastomeric ligatures. The perforations were positioned 3mm distal to the canine, following the root axis. The procedure was not repeated at any subsequent interval. | Canine retraction,  Maxilla | The primary outcome was the monthly rate of canine retraction. Alginate impressions were taken pre- and post- retraction . Plaster models from these two time points were scanned and digitized using Orthoanalyzer software and superimposed. Superimposition was performed on the third palatal rugae of the maxilla to ensure a stable reference. Canine movement was measured by calculating the distance between the most distal point of the canine and the most mesial point of the second premolar from an occlusal view. Molar mesialization was assessed by measuring the position of the mesiopalatal cusps of the first molars. |
| Sahin et al. ,2023 | Turkey | Inclusion:  presence of all permanent teeth (except third molars), 5mm crowding in mandibular arch (Little Irregularity Index), good oral hygiene and gingival/periodontal indices < 1, no extractions needed.  Exclusion:  congenital missing or extracted teeth, severe skeletal discrepancies, systemic or periodontal disease, previous orthodontic treatment. | parallel arm | Approximately 4 months  MOP: 105.57 ± 18.34 days  Control: 135.86 ± 15.12 days | 28 (losses to follow up: 0) | 16.25 ± 3.05 | 16F, 12M | Mandibular crowding (> 5mm) | Bonding was performed using SS brackets featuring a 0.022 × 0.028-inch slot. Leveling commenced with a sequence of wires: 0.014-inch, 0.016-inch, 0.016 × 0.022-inch, and 0.019 × 0.025-inch NiTi wires. Archwires were secured using steel ligature wires. | The MOPs were performed using the Propel driver, which features a 1.5mm thick perforation tip and adjustable depth settings of 3, 5, and 7mm, along with an LED depth indicator. Perforations were made directly in the keratinized gingiva, with depths of 3mm in the anterior region (canine to canine), 5mm in the premolar region (canine to molar), and 7mm in the posterior region. Vertically aligned perforations were created at two points between the mandibular central incisors and three points between the lateral incisors and canines, resulting in a total of eight perforations. In cases of crowding, additional perforations were performed in the posterior areas. The procedure was not repeated at any subsequent interval. | Alignment,  Mandible | Digital model analysis was performed on scanned plaster models to measure Little’s Irregularity Index (LII) on a monthly basis. LII measures the horizontal linear displacement of anatomic contact points of each mandibular incisor from the adjacent anatomic point and sums the five displacement together. Which represents the degree of anterior irregularity. |
| Li et al. ,2022 | Austrailia | Inclusion:  age: 12-25, permanent dentition, good oral hygiene, mild/moderate crowding, bilateral maxillary first premolar extractions needed.  Exclusion:  medical history/conditions affecting bone turnover, periodontal disease, smoking, previous orthodontic treatment. | split mouth | 12 weeks | 20 (losses to follow up at T2-T3: 3) | 12-25  (16.38) | 11F, 9M | Class I and Class II that required bilateral maxillary first premolars extraction | The upper arch was bonded with 0.022-inch self-ligating SPEED brackets. A Nance transpalatal arch was placed on the upper second molars for anchorage. Alignment was achieved using a sequence of archwires: 0.014-inch NiTi, 0.016-inch NiTi, 0.018 × 0.018-inch, and finally a 0.019 × 0.025-inch. Leveling and alignment were deemed complete after the 0.019 × 0.025-inch archwire was left passively for six weeks. Canine retraction was commenced immediately after MOP procedure. Pre-calibrated super-elastic NiTi coil springs were secured on the power arms (bent from 0.016 × 0.016-inch SS which were placed in the accessory slots of maxillary canine and frst molar brackets) via ligature wires on both sides of the arch to apply 150g of distalization force on maxillary canines. Force magnitude was measured with a strain gauge. Occlusal stops were bonded to the occlusal surfaces of the maxillary frst molars to allow unimpeded distalization of canines. | The Excellerator appliance (Propel Orthodontics) was utilized to create two buccal perforations. Each perforation was performed at a depth of 5mm in a vertical alignment, located 1-2mm distal to the upper canines. The two single perforations were positioned 5mm apart, with first perforation at a depth of 5mm from the alveolar crest. The procedure was not repeated at any subsequent interval. | Canine retraction,  Maxilla | Distance between the contact points of the canine and second premolar was measured from the casts using digital callipers. |
| Mordente et al. ,2024 | Brazil | Inclusion:  both genders, Age ≥ 16, indication of orthodontic maxillary incisor retraction, and presence of all permanent maxillary teeth (except for the third molars).  Exclusion:  diseases and/or use of medications that could affect bone biology, pregnancy, poor oral hygiene, previous orthodontic treatment, evidence of bone loss, active periodontitis, smoking, presence of syndromes or cleft palate, severe crowding, severe class II malocclusion (ANB > 7º, overjet > 10mm), Hyperdivergency (SNGoGn > 38º) | parallel arm | 4 weeks | 42 (losses to follow up: 5)  MOP: 19  Control: 18 | 16-40  MOP: (24.3 ± 8.1)  Control: (22.2 ± 4.2) | 20F 17M | Class I, Class II division 1, Class II division 2, Anterior crowding, Deep bite, Open bite, Biprotrusion | Treatment began with fixed appliances in both arches, utilizing a 0.022-inch slot Edgewise system. Following alignment and leveling, interradicular TADs (1.5 × 6 mm) were placed at the buccal mucogingival margin between the maxillary second premolars and first molars. | The MOP procedure was performed using CBCT-guided surgical guides. A total of 18 perforations (9 buccal and 9 palatal) were created using a 1.6mm SS surgical bur, with depths of 3mm on buccal surfaces and 5mm on palatal surfaces. The perforations were placed perpendicular to the alveolar bone in specific interproximal locations: between canines and lateral incisors, between central and lateral incisors, and between central incisors (both buccal and palatal aspects).  Two vertically aligned perforations were placed distal to each maxillary incisor, spaced 5mm apart, with the first perforation positioned 6mm from the gingival margin. However, between the central incisors, only the most apical perforation was made due to cervical root proximity concerns. The procedure was not repeated during subsequent intervals. | Alignment,  Maxilla | Tooth movement was assessed through digital model superimposition using the palatal rugae as a stable reference. CBCT scans were used to evaluate changes in root length and tooth inclination. |

# Appendix Table 3. Standardizing Measured Distances

| **Study** | **Distance(s) Measured (Simplified)** | **Final Category** |
| --- | --- | --- |
| Golshah et al. ,2021 | (a) Canine movement via palatal rugae superimposition, (b) miniscrew line **Chosen**: C–Model | **C–Model** |
| Thomas et al. ,2021 | Bracket slot to bracket slot, bracket hook to micro‐implant (purely appliance references) | **C–TAD / Appliance** |
| Martina et al. ,2022 | Palatal rugae reference to canine (no standard tooth–tooth) | **C–Model** |
| Gulduren et al. ,2020 | Miniscrew midpoint to distal margin of 1st molar | **C–TAD / Appliance** |
| Feizbakhsh et al. ,2018 | Canine to second premolar (three sub-measures: bracket centers, cusp tips, cervicogingival) | **C2P** |
| Fattori et al. ,2020 | Distal canine to mesial second premolar (extraction space) | **EXT** |
| Farag et al. ,2021 | Canine cusp tip to mesiobuccal cusp tip of 1st molar | **C1M** |
| El Awady et al. ,2019 | Distal contact of canine to mesial contact of second premolar | **C2P** |
| Bansal et al. ,2019 | Little’s Irregularity Index only | **LII only** |
| Al-Hayek et al. ,2019 | Little’s Irregularity Index only | **LII only** |
| Attri et al. ,2018 | Extraction space: distal canine to mesial second premolar | **EXT** |
| Babanouri et al. ,2020 | Canine–lateral incisor (incisal, middle, cervical) | **CL** |
| Mahmoudi et al. ,2016 | Multiple standard pairs measured but **main** is “canine to second premolar” (C2P) | **C2P** |
| GOWDA S V et al. ,2019 | Canine to lateral incisor (vertical lines at incisal/middle/cervical) | **CL** |
| RASHEED. V et al. ,2018 | Canine to lateral incisor at incisal | **CL** |
| Alfailany et al. ,2023 | 3rd palatal rugae to canine cusp, rugae to 1st molar (model reference) | **C–Model** |
| Rashid et al. ,2018 | Canine to second premolar | **C2P** |
| Aboalnaga et al. ,2019 | Canine cusp tip & 1st molar cusp to custom “frontal plane” (not standard IRP/MRP) | **C–Model** |
| Alikhani et al. ,2013 | Canine to lateral incisor (incisal, middle, cervical) | **CL** |
| Alqadasi et al. ,2019 | Canine cusp tip to perpendicular from second premolar cusp tip | **C2P** |
| Kundi et al. ,2018 | (a) Canine cusp tip to lateral incisor incisal midpoint, (b) canine cervical midpoint to lateral incisor cervical midpoint | **CL** |
| Venkatachalapathy et al. ,2022 | Canine to lateral incisor (incisal, middle, cervical) | **CL** |
| RAGHAV et al. ,2020 | Midpoint of canine’s long axis → reference wire in an acrylic palatal plug | **C–Model** |
| Singh et al. ,2023 | Canine to lateral incisor (3 vertical points) | **CL** |
| Abdelhameed et al. ,2018 | Canine cusp tip to mesiobuccal cusp tip of 1st molar | **C1M** |
| Ahsan et al. ,2021 | Canine–second premolar (incisal, middle, cervical) | **C2P** |
| Alqadasi et al. ,2024 | Canine/premolar vs. midpalatine raphe (custom model reference) | **C–Model** |
| Alkebsi et al. ,2018 | 3D displacement of canines (model superimposition) + bracket to bracket references, but **main** measurement is 3D cast superimposition | **C–Model** |
| Hashem et al. ,2024 | 3D models (Dolphin), no specific distance | **NA** |
| Joseph et al. ,2022 | Canine cusp tip to mesiobuccal cusp tip of 1st molar | **C1M** |
| Kilinc and Baka ,2023 | Little’s Irregularity Index only | **LII only** |
| Bavikati et al. ,2022 | Canine to lateral incisor (vertical lines on long axes) | **CL** |
| Kumar et al. ,2024 | Extraction space (distal canine to mesial premolar) | **EXT** |
| Al-Attar et al. ,2022 | Little’s Irregularity Index only | **LII only** |
| Gümüş et al. ,2023 | Distal canine to mesial second premolar (extraction space) | **EXT** |
| Ozkan et al. ,2021 | (a) Distal canine → mesial 2nd premolar (C2P) plus molar mesialization, but **main** measure of interest is the C2P retraction | **C2P** |
| Sahin et al. ,2023 | Little’s Irregularity Index only | **LII only** |
| Li et al. ,2022 | Canine to second premolar contact points (new text confirms standard distance) | **C2P** |
| Mordente et al. ,2024 | Palatal rugae superimposition + CBCT for root length, no standard tooth–tooth | **C–Model** |

**Categories Key:**

CL = Canine–Lateral Incisor

C2P = Canine–Second Premolar

C1M = Canine–First Molar

EXT = Extraction space (distal canine → mesial premolar)

LII only = Little’s Irregularity Index only (no direct distance)

C–Model = Model-based custom references (palatal rugae, 3D cast superimposition, or acrylic plugs), not a standard tooth–tooth or tooth–plane

C–TAD / Appliance = Any measurement referencing a TAD, miniscrew, or bracket (but no standard tooth–tooth pair)

**Appendix Table 4 Exploratory Subgroup Levels for MOP Effect on OTM (subgroup levels with 1–2 studies)**

| Subgroup | Items | Study numbers | Change of SMD | CI (95%) | I2 (%) | p-value (Between Groups) |
| --- | --- | --- | --- | --- | --- | --- |
| OTM Measurement | Combined | 1 | -0.07 | [-0.55 to 0.42] | – | Raw: < 0.001; B: < 0.022 |
| Malocclusion | Bimax protrusion | 1 | 2.69 | [1.84 to 3.53] | – | Raw: 0.03; B: 0.660 |
| Measured Distance | C-TAD/Appliance | 2 | 0.65 | [-0.40 to 1.70] | 77.66 | Raw: < 0.001; B: < 0.022 |
| Type of Bracket | Edgewise | 1 | 0.17 | [-0.47 to 0.80] | – | Raw: < 0.001; B: < 0.022 |
|  | Mini Master | 1 | 1.02 | [0.25 to 1.79] | – |  |
|  | Self-ligating Speed | 1 | 0.19 | [-0.47 to 0.85] | – |  |
| Working Wire Dimension | 0.016 × 0.022 | 2 | 1.03 | [0.58 to 1.47] | 0 | Raw: 0.01; B: 0.220 |
|  | 0.018 × 0.025 | 1 | -0.08 | [-0.92 to 0.76] | – |  |
| Type of OTM | Molar Distalization | 1 | 0.06 | [-0.78 to 0.90] | – | Raw: < 0.001; B: < 0.022 |
| Jaws and Type of OTM | Mandibular Canine Retraction | 1 | 3.89 | [2.84 to 4.93] | – | Raw: < 0.001; B: < 0.022 |
|  | Maxillary Alignment | 1 | 0.17 | [-0.47 to 0.80] | – |  |
|  | Maxillary and Mandibular Alignment | 2 | 2.24 | [-2.14 to 6.62] | 95.50 |  |
|  | Maxillary Molar Distalization | 1 | 0.06 | [-0.78 to 0.90] | – |  |
| Depth and Instrument | ≥ 5mm with Propel | 2 | 0.58 | [-0.23 to 1.39] | 61.15 | Raw: 0.05; B: 1.000 |
| Depth and Jaws | ≥ 5mm in Mandible | 2 | 2.43 | [-0.38 to 5.24] | 94.67 | Raw: < 0.001; B: < 0.022 |
|  | ≥ 5mm in Maxilla and Mandible | 1 | 0.06 | [-0.82 to 0.94] | – |  |
| Total perforations and jaw | > 3 in Maxilla and Mandible | 2 | 3.47 | [1.68 to 5.25] | 73.98 | Raw: 0.16; B: 1.000 |
| Jaw and total perforations and depth | ≤ 3, < 5mm in Maxilla and Mandible | 2 | 1.62 | [1.17 to 2.07] | 0 | Raw: < 0.001; B: < 0.022 |
|  | ≤ 3, ≥ 5mm in Maxilla and Mandible | 1 | 0.06 | [-0.82 to 0.94] | – |  |
|  | > 3, < 5mm in Maxilla and Mandible | 2 | 3.47 | [1.68 to 5.25] | 73.98 |  |
|  | > 3, ≥ 5mm in Mandible | 2 | 2.34 | [-0.38 to 5.24] | 94.67 |  |
|  | > 3, ≥ 5mm in Maxilla | 1 | 0.06 | [-0.78 to 0.90] | – |  |

The “p-value (Between Groups)” refers to the overall subgroup comparison as originally computed for each subgroup family; the tables are split for reporting clarity based on evidentiary strength (≥3 studies in main text; 1–2 studies in Appendix).

B: Bonferroni-adjusted p-value for between-group differences.

SMD: Standardized Mean Difference (Hedges’s g).

CI: Confidence Interval.

I²: Heterogeneity.

** “Repeat of MOP” pertains to baseline to last measurement, whereas other rows are baseline to 4 weeks

# Appendix Table 5. Outcomes’ Summarizes

| Study | Primary Outcome | | Secondary Outcomes | |
| --- | --- | --- | --- | --- |
| 1.Abdelhameed et al. (2018) | Group A (MOP only) – Mean distance traveled by the maxillary canine (mm) | 2 weeks:; Control: N=10, Mean ± SD= 0.63 ± 0.62; MOP: N=10, Mean ± SD= 1.30 ± 0.12; MD = 0.67 mm; P < 0.001;  4 weeks:; Control: N=9, Mean ± SD= 1.31 ± 0.23; MOP: N=9, Mean ± SD= 2.16 ± 0.27; MD = 0.85 mm; P < 0.001;  6 weeks:; Control: N=10, Mean ± SD= 1.80 ± 0.66; MOp: N=10, Mean ± SD= 2.92 ± 0.73; MD = 1.12 mm; P < 0.001;  8 weeks:; Control: N=10, Mean ± SD= 1.97 ± 0.76; MOP: N=10, Mean ± SD= 3.43 ± 0.66; MD = 1.46 mm; P = 0.001;  10 weeks:; Control: N=9, Mean ± SD= 2.56 ± 0.83; MOP: N=9, Mean ± SD= 3.92 ± 0.88; MD = 1.36 mm; P = 0.001;  12 weeks:; Control: N=10, Mean ± SD= 2.82 ± 0.39; MOP: N=10, Mean ± SD= 4.33 ± 0.64; MD = 1.51 mm; P = 0.001 | 1.Anchorage loss (mm) (To calculate Anchorage loss, the distance between the medial end of the third palatal rugae and the central fossa of upper first molar is measured)  2. Canine rotation (in degrees) (To calculate canine rotation, the angle between the mid-palatal suture and the line through the mesial and distal edges of the maxillary canine is measured)  3. Canine rotation (in degrees) | T0-T1 (1st month)  TC: n = 17, mean = 0.57, SD = 0.24, CI (LL = 0.45, UL = 0.69)  FCAPs: n = 17, mean = 0.76, SD = 0.33, CI (LL = 0.60, UL = 0.94)  Control: n = 17 mean = 2.09, SD = 0.79, CI (LL = 1.68, UL = 2.50)  p-value < 0.001  T1-T2 (2nd month)  TC: n = 17, mean = 0.90, SD = 0.53, CI (LL = 0.62, UL = 1.17)  FCAPs: n = 17, mean = 0.93, SD = 0.58, CI (LL = 0.63, UL = 1.23)  Control: n = 17 mean = 0.94, SD = 0.60, CI (LL = 0.63, UL = 1.24)  p-value = 0.975  T2-T3 (3rd month)  TC: n = 17, mean = 1.93, SD = 0.68, CI (LL = 1.58, UL = 2.28)  FCAPs: n = 17, mean = 1.08, SD = 0.75, CI (LL = 0.70, UL = 1.47)  Control: n = 17 mean = 0.79, SD = 0.53, CI (LL = 0.52, UL = 1.06)  p-value < 0.001  T3-T4 (the end of canine retraction)  TC: n = 17, mean = 0.60, SD = 0.61, CI (LL = 0.29, UL = 0.92)  FCAPs: n = 17, mean = 0.34, SD = 0.42, CI (LL = 0.13, UL = 0.56)  Control: n = 17 mean = 0.59, SD = 0.30, CI (LL = 0.43, UL = 0.74)  p-value = 0.202  Anchorage loss (mm)  T0-T1 (1st month)  Comparison group  TCG vs. FCAPG : n = 34, mean difference = -0.19, p-value = 0.806  TCG vs. CG : n = 34, mean difference = -1.52, p-value < 0.001  FCAPG vs. CG : n = 34, mean difference = -1.33, p-value < 0.001  T2-T3 (3rd month)  TCG vs. FCAPG : n = 34, mean difference = 0.85, p-value = 0.001  TCG vs. CG : n = 34, mean difference = 1.14, p-value < 0.001  FCAPG vs. CG : n = 34, mean difference = 0.29, p-value =0.587  ,  Canine retraction: T0-T1 (1st month)  TC: n = 17, mean = 4.24̊ , SD = 2.99̊ , CI (LL = 2.7̊ , UL = 5.78̊ )  FCAPs: n = 17, mean = 8.89̊ , SD = 2.67̊ , CI (LL = 7.52̊ , UL = 10.27̊ )  Control: n = 17 mean = 12.95̊ , SD = 3.47̊ , CI (LL = 11.17̊ , UL = 14.74̊ )  p-value < 0.001  T1-T2 (2nd month)  TC: n = 17, mean = 7.02̊ , SD = 4.43̊ , CI (LL = 4.74̊ , UL = 9.30̊ )  FCAPs: n = 17, mean = 3.70̊ , SD = 1.67̊ , CI (LL = 2.84̊ , UL = 4.56̊ )  Control: n = 17 mean = 5.30̊ , SD = 1.78̊ , CI (LL = 4.38̊ , UL = 6.22̊ )  p-value = 0.007  T2-T3 (3rd month)  TC: n = 17, mean = 2.40̊ , SD = 2.37̊ , CI (LL = 1.18̊ , UL = 3.63̊ )  FCAPs: n = 17, mean = 4.02̊ , SD = 1.16̊ , CI (LL = 3.43̊ , UL = 4.62̊ )  Control: n = 17 mean = 3.29̊ , SD = 1.90̊ , CI (LL = 2.28̊ , UL = 4.30̊ )  p-value = 0.054  T3-T4 (the end of canine retraction)  TC: n = 17, mean = 5.76̊ , SD = 3.11̊ , CI (LL = 4.16̊ , UL = 7.37̊ )  FCAPs: n = 17, mean = 2.73̊ , SD = 1.62̊ , CI (LL = 1.90̊ , UL = 3.57̊ )  Control: n = 17 mean = 1.94̊ , SD = 0.91̊ , CI (LL = 1.47̊ , UL = 2.41̊ )  p-value <0.001  ,  Canine rotation: T0-T1 (1st month)  Comparison group  TCG vs. FCAPG : n = 34, mean difference = -4.65̊ , p-value < 0.001  TCG vs. CG : n = 34, mean difference = -8.71̊ , p-value < 0.001  FCAPG vs. CG : n = 34, mean difference = -4.06̊ , p-value = 0.001    T3-T4 (the end of canine retraction)  TCG vs. FCAPG : n = 34, mean difference = 3.03̊ , p-value < 0.001  TCG vs. CG : n = 34, mean difference = 3.82̊ , p-value < 0.001  FCAPG vs. CG : n = 34, mean difference = 0.79̊ , p-value =0.823 |
| 2.Aboalnaga et al. (2019) | Total distances moved by the canine (mm)  using pre- and post-retraction CBCT images at three points; canine cusp tip, center, and root apex, all relative to the frontal plane (FP). | Can-tip-FP:  MOP: N=18 , Mean ± SD: 3.34 ± 2.28  Control: N=18 , Mean ± SD: 3.29 ± 2.39  Mean difference: 0.06 , SD: 0.72 , CI (LL: -0.33 UL:0.44) , P-value: 0.75764  Can-C-FP:  MOP: N=18, Mean ± SD: 2.13 ± 0.75  Control: N=18, Mean ± SD: 1.76 ± 0.91  Mean difference: 0.37 , SD: 0.63 , CI (LL: 0.02 UL:0.71) , P-value: 0.04121  Can-apex-FP:  MOP: N=18, Mean ± SD: 1.51 ± 0.95  Control: N=18, Mean ± SD: 1.04 ± 0.87  Mean difference: 0.47 , SD: 0.56 , CI (LL: 0.17 UL:0.77) , P-value: 0.00419  Distance moved by the canines  using digital models, the distance between the canine cusp tip and the frontal plane (FP) was calculated.  (SD hasn’t been reported)  T1 (1st month)  MOP: N=18, Mean: 1.14  Control: N=18, Mean: 0.96  T2 (2nd month)  MOP: N=18, Mean: 1.09  Control: N=18, Mean: 1.41  T3 (3rd month)  MOP: N=18, Mean: 0.87  Control: N=18, Mean: 0.63  T4 (4th month)  MOP: N=18, Mean: 0.88  Control: N=18, Mean: 0.97 | 1.Pain/discomfort: The Numeric Pain Rating Scale (1–10) was explained to patients to assess pain after MOPs at T0 (immediately), T1 (1 day after), T2 (3 days after), and T3 (1 week after). Scores: 1–3 (mild), 4–6 (moderate), 7–10 (severe).  2.Root resorption: assessed using Anatomage software. CBCT images were reoriented to align sagittal and coronal cuts with the canine’s long axis. Two cross-sections showing maximum root length were selected and scored (0–4) using the Malmgren index for labio-lingual and mesio-distal views. Pre- and post-retraction score differences were calculated as follows: zero, the pre- and post- retraction scores were identical; one, the post-retraction score was 1 score greater than the pre-retraction score; two, the post-retraction score was 2 scores greater than the pre-retraction score, and analyzed statistically.   3.Anchorage loss:  Total distances moved by the canine (mm) using pre- and post-retraction CBCT images at three points; First molar mesiobuccal cusp tip, mesiobuccal root center, and mesiobuccal root apex, all relative to the frontal plane (FP). | Pain/discomfort:  MOP T0: N=18, mean: 4.88, SD: 0.56 T1: N=18, mean: 2.69, SD: 0.75 T2: N=18, mean: 1.31, SD: 0.57 T3: N=18, mean: 0.63, SD: 0.43 Control: N/A;  ,  Canine root resorption scores (chi-squared test) Control side: Pre- retraction  0: N=28 (82.4%) , 1: N=2 (5.9%) 2: N=3 (8.8%), 3: N=1(2.9%) Post- retraction  0: N=25 (73.5%) , 1: N=2 (5.9%) 2: N=6 (17.6%), 3: N=1(2.9%) Chi-squared: 1.17 P-value: 0.76025  MOP side: Pre- retraction  0: N=30 (88.2%) , 1: N=2 (5.9%) 2: N=2 (5.9%), 3: N=0 (0.0%) Post- retraction  0: N=23 (67.6%) , 1: N=3 (8.8%) 2: N=6 (17.6%), 3: N=2 (5.9%) Chi-squared: 5.12 P-value: 0.16290  Difference between MOP and Control: Pre- retraction  0: N=24 (70.6%) , 1: N=5 (14.7%) 2: N=5 (14.7%) Post- retraction  0: N=30 (67.6%) , 1: N=2 (8.8%) 2: N=2 (17.6%) Chi-squared: 3.24 P-value: 0.198;  ,  Anchorage loss:   Mol tip-FP: MOP: N=18, Mean ± SD: − 0.45 ± 0.59 Control: N=18, Mean ± SD: − 0.63 ± 0.49 Mean difference: − 0.18 , SD: 0.43 , CI (LL: − 0.41 UL: 0.05) , P-value: 0.11229  Mol C-FP: MOP: N=18, Mean ± SD: − 0.16 ± 0.55 Control: N=18, Mean ± SD: − 0.25 ± 1.82 Mean difference: − 0.09 , SD: 1.84 , CI (LL: − 1.07 UL: 0.89) , P-value: 0.84745  Mol apex-FP: MOP: N=18, Mean ± SD: 0.13 ± 0.80 Control: N=18, Mean ± SD: − 0.07 ± 0.73 Mean difference: − 0.20 , SD: 0.61 , CI (LL: − 0.53 UL: 0.13) , P-value: 0.21226 |
| 3.Ahsan et al. (2021) | Rate of Tooth Movement (Canine Retraction). rate reported as mm over 3 weeks period. MD is not reported but it can be calculated from group means. | Control: N = 30, Mean = 0.66, SD = 0.31; MOP: N = 30, Mean = 1.61, SD = 0.52; p-value (Experimental vs Control): 0.038 (Statistically Significant) | Pain/discomfort | Patients reported slightly elevated pain levels immediately after MOP, subsiding within a few days. |
| 4.Al-Attar et al. (2022) | Rate of Tooth Movement Little’s Irregularity Index (LII) | Baseline (T0):  MOP: N=17, M = 5.12 mm (95% CI = 4.90–5.32).  Control: N = 16, M = 5.08 mm (95% CI = 4.86–5.28).  p-value: 0.766 (no significant difference).  T1 (4 weeks):  MOP: N=17, M = 3.03 mm (95% CI = 2.86–3.17).  Control: N=16, M = 4.18 mm (95% CI = 4.05–4.30).  Mean Difference (MD) = -1.16 mm (95% CI = -1.36 to -0.96), p < 0.001.  T2 (8 weeks):  MOP: N=17, M = 1.27 mm (95% CI = 1.10–1.42).  Control: N=16, M = 3.03 mm (95% CI = 2.96–3.09).  MD = -1.77 mm (95% CI = -1.93 to -1.59), p < 0.001.  T3 (12 weeks):  MOP: N=17, M = 0.44 mm (95% CI = 0.38–0.48).  Control: N=16, M = 2.01 mm (95% CI = 1.92–2.09).  MD = -1.58 mm (95% CI = -1.67 to -1.48), p < 0.001.  Overall Treatment Time:  MOP: 10.41 weeks (95% CI = 9.92–10.89).  Control: 16.62 weeks (95% CI = 16.11–17.13).  MD = -6.21 weeks (95% CI = -6.88 to -5.53), p < 0.001. | 1.Pain/discomfort: VAS Scores (0-10)  2.Root resorption | 1.Pain/discomfort: VAS Scores (0-10)  MOP: N=17  Day 2:  Mean = 5.3 cm, SD = 1.1 cm.  95% CI: LL = 4.9, UL = 5.7.  Day 7:  Mean = 3.1 cm, SD = 0.9 cm.  95% CI: LL = 2.8, UL = 3.4.  Control: N=16  Day 2:  Mean = 4.8 cm, SD = 0.8 cm.  95% CI: LL = 4.5, UL = 5.1.  Day 7:  Mean = 3.0 cm, SD = 1.0 cm.  95% CI: LL = 2.6, UL = 3.4.  Statistical Analysis:  p-value (Day 2): 0.052 (not statistically significant).  2.Root resorption:  MOP: N=17 Pre-Treatment Root Resorption: Mean Rank: 17.29. Post-Treatment Root Resorption: Mean Rank: 18.41.  Control: N=16 Pre-Treatment Root Resorption: Mean Rank: 16.69. Post-Treatment Root Resorption: Mean Rank: 15.50. Statistical Analysis: Pre-Treatment p-value: 0.873 (not statistically significant). Post-Treatment p-value: 0.402 (not statistically significant). |
| 5.Alfailany et al. (2023) | Amount of canine retraction (mm) (To calculate canine retraction, the distance between the medial end of the third palatal rugae and the tip of the upper canine cusp is measured)  TC (traditional corticotomy)  FCAPs (flapless cortico-alveolar perforations) | T0-T1 (1st month)  TC: n = 17, mean = 2.79, SD = 0.39, CI (LL = 2.60, UL = 2.99)  FCAPs: n = 17, mean = 2.00, SD = 0.35, CI (LL = 1.82, UL = 2.19)  Control: n = 17 mean = 1.12, SD = 0.16, CI (LL = 1.04, UL = 1.20)  p-value < 0.001  T1-T2 (2nd month)  TC: n = 17, mean = 1.89, SD = 0.42, CI (LL = 1.68, UL = 2.11)  FCAPs: n = 17, mean = 2.45, SD = 0.59, CI (LL = 2.15, UL = 2.75)  Control: n = 17 mean = 1.22, SD = 0.32, CI (LL = 1.06, UL = 1.38)  p-value < 0.001  T2-T3 (3rd month)  TC: n = 17, mean = 1.05, SD = 0.40, CI (LL = 0.85, UL = 1.26)  FCAPs: n = 17, mean = 0.86, SD = 0.32, CI (LL = 0.70, UL = 1.03)  Control: n = 17 mean = 1.19, SD = 0.31, CI (LL = 1.03, UL = 1.35)  p-value = 0.029  T3-T4 (the end of canine retraction)  TC: n = 17, mean = 1.08, SD = 0.44, CI (LL = 0.17, UL = 2.33)  FCAPs: n = 17, mean = 1.68, SD = 0.59, CI (LL = 1.38, UL = 1.99)  Control: n = 17 mean = 1.52, SD = 0.58, CI (LL = 1.22, UL = 1.81)  p-value = 0.478  …………………………………………………………..  Canine retraction amount (mm)  T0-T1 (1st month)  Comparison group  TCG vs. FCAPG : n = 34, mean difference = 0.79, p-value < 0.001  TCG vs. CG : n = 34, mean difference = 1.67, p-value < 0.001  FCAPG vs. CG : n = 34, mean difference = 0.88, p-value < 0.001  T1-T2 (2nd month)  TCG vs. FCAPG : n = 34, mean difference = -0.56, p-value = 0.003  TCG vs. CG : n = 34, mean difference = 0.67, p-value < 0.001  FCAPG vs. CG : n = 34, mean difference = 1.23, p-value < 0.001 | 1.Anchorage loss: (mm) (To calculate Anchorage loss, the distance between the medial end of the third palatal rugae and the central fossa of upper first molar is measured)    2.Canine rotation (in degrees) (To calculate canine rotation, the angle between the mid-palatal suture and the line through the mesial and distal edges of the maxillary canine is measured) | T0-T1 (1st month) TC: n = 17, mean = 0.57, SD = 0.24, CI (LL = 0.45, UL = 0.69) FCAPs: n = 17, mean = 0.76, SD = 0.33, CI (LL = 0.60, UL = 0.94) Control: n = 17 mean = 2.09, SD = 0.79, CI (LL = 1.68, UL = 2.50) p-value < 0.001  T1-T2 (2nd month) TC: n = 17, mean = 0.90, SD = 0.53, CI (LL = 0.62, UL = 1.17) FCAPs: n = 17, mean = 0.93, SD = 0.58, CI (LL = 0.63, UL = 1.23) Control: n = 17 mean = 0.94, SD = 0.60, CI (LL = 0.63, UL = 1.24) p-value = 0.975  T2-T3 (3rd month) TC: n = 17, mean = 1.93, SD = 0.68, CI (LL = 1.58, UL = 2.28) FCAPs: n = 17, mean = 1.08, SD = 0.75, CI (LL = 0.70, UL = 1.47) Control: n = 17 mean = 0.79, SD = 0.53, CI (LL = 0.52, UL = 1.06) p-value < 0.001  T3-T4 (the end of canine retraction) TC: n = 17, mean = 0.60, SD = 0.61, CI (LL = 0.29, UL = 0.92) FCAPs: n = 17, mean = 0.34, SD = 0.42, CI (LL = 0.13, UL = 0.56) Control: n = 17 mean = 0.59, SD = 0.30, CI (LL = 0.43, UL = 0.74) p-value = 0.202  …………………………………………………………..  Anchorage loss (mm) T0-T1 (1st month) Comparison group TCG vs. FCAPG : n = 34, mean difference = -0.19, p-value = 0.806  TCG vs. CG : n = 34, mean difference = -1.52, p-value < 0.001  FCAPG vs. CG : n = 34, mean difference = -1.33, p-value < 0.001  T2-T3 (3rd month)  TCG vs. FCAPG : n = 34, mean difference = 0.85, p-value = 0.001  TCG vs. CG : n = 34, mean difference = 1.14, p-value < 0.001  FCAPG vs. CG : n = 34, mean difference = 0.29, p-value =0.587; Other outecome(s) or result(s): Canine rotation (in degrees) (To calculate canine rotation, the angle between the mid-palatal suture and the line through the mesial and distal edges of the maxillary canine is measured) T0-T1 (1st month) TC: n = 17, mean = 4.24̊ , SD = 2.99̊ , CI (LL = 2.7̊ , UL = 5.78̊ ) FCAPs: n = 17, mean = 8.89̊ , SD = 2.67̊ , CI (LL = 7.52̊ , UL = 10.27̊ ) Control: n = 17 mean = 12.95̊ , SD = 3.47̊ , CI (LL = 11.17̊ , UL = 14.74̊ ) p-value < 0.001  T1-T2 (2nd month) TC: n = 17, mean = 7.02̊ , SD = 4.43̊ , CI (LL = 4.74̊ , UL = 9.30̊ ) FCAPs: n = 17, mean = 3.70̊ , SD = 1.67̊ , CI (LL = 2.84̊ , UL = 4.56̊ ) Control: n = 17 mean = 5.30̊ , SD = 1.78̊ , CI (LL = 4.38̊ , UL = 6.22̊ ) p-value = 0.007  T2-T3 (3rd month) TC: n = 17, mean = 2.40̊ , SD = 2.37̊ , CI (LL = 1.18̊ , UL = 3.63̊ ) FCAPs: n = 17, mean = 4.02̊ , SD = 1.16̊ , CI (LL = 3.43̊ , UL = 4.62̊ ) Control: n = 17 mean = 3.29̊ , SD = 1.90̊ , CI (LL = 2.28̊ , UL = 4.30̊ ) p-value = 0.054  T3-T4 (the end of canine retraction) TC: n = 17, mean = 5.76̊ , SD = 3.11̊ , CI (LL = 4.16̊ , UL = 7.37̊ ) FCAPs: n = 17, mean = 2.73̊ , SD = 1.62̊ , CI (LL = 1.90̊ , UL = 3.57̊ ) Control: n = 17 mean = 1.94̊ , SD = 0.91̊ , CI (LL = 1.47̊ , UL = 2.41̊ ) p-value <0.001  Canine rotation (in degrees) T0-T1 (1st month) Comparison group TCG vs. FCAPG : n = 34, mean difference = -4.65̊ , p-value < 0.001 TCG vs. CG : n = 34, mean difference = -8.71̊ , p-value < 0.001  FCAPG vs. CG : n = 34, mean difference = -4.06̊ , p-value = 0.001   T3-T4 (the end of canine retraction) TCG vs. FCAPG : n = 34, mean difference = 3.03̊ , p-value < 0.001 TCG vs. CG : n = 34, mean difference = 3.82̊ , p-value < 0.001  FCAPG vs. CG : n = 34, mean difference = 0.79̊ , p-value =0.823 |
| 6.Al-Hayek et al. (2019) | Alignment improvement percentage: Based on the Irregularity Index at the beginning of the study and at each time interval, the following equation was used:  (Pre-operative value – Post-operative value) / Pre-operative value x 100 | Time interval (day 14):  MOP: N=11, mean=31.2% , SD=13.2%  Control: N=10, mean=27.6% , SD=14.4%  p-value: 0.398  Effect size (d): 0.375  Time interval (1month):  MOP: N=11, mean=62% , SD=19.2%  Control: N=10, mean=41.4% , SD=17.9%  p-value: 0.029 (significant)  Effect size (d):1.084  Time interval (1.5month):  MOP: N=11, mean=83.3% , SD=22%  Control: N=10, mean=58.8% , SD=19.6%  p-value: 0.019 (significant)  Effect size (d):1.177  Time interval (2month):  MOP: N=11, mean=92% , SD=14.1%  Control: N=10, mean=69.8% , SD=19.6%  p-value: 0.007 (significant)  Effect size (d):1.355  Time interval (2.5month):  MOP: N=11, mean=97.1% , SD=6.4%  Control: N=10, mean=78.9% , SD=19.2%  p-value: 0.004 (significant)  Effect size (d):1.439  Time interval (3month):  MOP: N=11, mean=100% , SD=0%  Control: N=10, mean=86.1% , SD=17.7%  p-value: 0.004 (significant)  Effect size (d):1.177  Time interval (3.5month):  MOP: N=11, mean=100% , SD=0%  Control: N=10, mean=92% , SD=15.3%  p-value: 0.024 (significant)  Effect size (d):0.718  Time interval (4month):  MOP: N=11, mean=100% , SD=0%  Control: N=10, mean=94.9% , SD=13.1%  p-value: 0.056  Effect size (d):0.524  Time interval (4.5month):  MOP: N=11, mean=100% , SD=0%  Control: N=10, mean=96.4% , SD=11.4%  p-value: 0.294  Effect size (d):0.170  Time interval (5month):  MOP: N=11, mean=100% , SD=0%  Control: N=10, mean=97.6% , SD=7.6%  p-value: 0.294  Effect size (d):0.170  Time interval (5.5month):  MOP: N=11, mean=100% , SD=0%  Control: N=10, mean=98.5% , SD=4.7%  p-value: 0.294  Effect size (d):0.170  Time interval (6month):  MOP: N=11, mean=100% , SD=0%  Control: N=10, mean=99.5% , SD=1.6%  p-value: 0.294  Effect size (d):0.170  …………………………………………………………...  Comparison between Little’s Irregularity Index  Pre-operative (day 0)  MOP: N=11, mean=8.48 , SD=0.86  Control: N=10, mean=6.4 , SD=2.77  p-value: 0.294  Effect size (d): 0.794  Day 14  MOP: N=11, mean=6.15 , SD=1.45  Control: N=10, mean=4.60 , SD=3.55  p-value: 0.456  Effect size (d): 0.546  1 month  MOP: N=11, mean=3.63 , SD=2.25  Control: N=10, mean=3.87 , SD=3.44  p-value: 0.881  Effect size (d): 0.106  1.5 month  MOP: N=11, mean=2.68 , SD=2.45  Control: N=10, mean=2.5 , SD=2.29  p-value: 0.724  Effect size (d): 0.270  2 months  MOP: N=11, mean=1.95 , SD=1.69  Control: N=10, mean=1.5 , SD=1.8  p-value: 1.000  Effect size (d): 0.000  2.5 months  MOP: N=11, mean=1.42 , SD=0.23  Control: N=10, mean=1.4 , SD=0.85  p-value: 0.564  Effect size (d): 0.535  3 months  MOP: N=11, mean=0 , SD=0  Control: N=10, mean=0.7 , SD=0.99  p-value: 0.414  Effect size (d): 0.535  3.5 months  MOP: N=11, mean=0 , SD=0  Control: N=10, mean=0 , SD=0  p-value: 1.000  Effect size (d): 0.000  ……………………………………………………………  overall time for leveling and alignment (days):  MOP: N=11 , mean ± SD: 57.3 ± 19.9  Control: N=10 , mean ± SD: 109.5 ± 34.7 | Pain/discomfort | After 1 day, the MOP group had higher pain (VAS) scores than the Control group, but no significant differences were found on days 2–7. Vas score Day 1 MOP: n=11 mean:5.5, SD:1.3 Control (N/A) |
| 7.Alikhani et al. (2013) | Canine retraction (mm):  The distance between vertical lines along the palatal surfaces of the canine and lateral incisor at the incisal, middle, and cervical thirds was used to measure canine retraction. | T:28days  Incisal  MOP: N=10, Mean±SD= 1.27±0.15  Control: N=10, Mean±SD= 0.55±0.14  Middle  MOP: N=10, Mean±SD= 1.12±0.12  Control: N=10, Mean±SD= 0.49±0.13  Cervical  MOP: N=10, Mean±SD= 1.05±0.12  Control: N=10, Mean±SD= 0.43±0.11 | 1.Periodontal health: CGF samples were obtained from the distobuccal sides of the canines at different times (T0 (Before Rt), T1 (Day 1) T2 (Day 7), T3 (Day 28))      2.Pain/discomfort: Pain and discomfort levels were assessed using a numeric rating scale from 0 to 10, 0 would mean “no pain” and 10 would mean “worst possible pain.” The participants were asked to assess their level of discomfort. (1-10 has also been mentioned) | Periodontal health:   CCL-2 (pg/mL) T0 (Before Rt) Control: N=10 , Mean±SD= 0.35±0.11 T1 (Day 1) Control: N=10, Mean±SD= 1.4±0.4 MOP: N=10, Mean±SD= 5.6±0.8 T2 (Day 7) Control: N=10, Mean±SD= 0.38±0.19 MOP: N=10, Mean±SD= 0.43±0.16 T3 (Day 28) Control: N=10, Mean±SD= 0.37±0.20 MOP: N=10, Mean±SD= 0.40±0.17  CCL-3 (pg/mL) T0 (Before Rt) Control: N=10 , Mean±SD= 1.8±0.4 T1 (Day 1) Control: N=10, Mean±SD= 3.8±0.3 MOP: N=10, Mean±SD= 8.7±1.0 T2 (Day 7) Control: N=10, Mean±SD= 2.5±0.5 MOP: N=10, Mean±SD= 3.9±0.6 T3 (Day 28) Control: N=10, Mean±SD= 2.6±0.4 MOP: N=10, Mean±SD= 2.9±0.7  CCL-5 (pg/mL) T0 (Before Rt) Control: N=10 , Mean±SD= 7.9±1.3 T1 (Day 1) Control: N=10, Mean±SD= 13.4±1.9 MOP: N=10, Mean±SD= 22.3±2.2 T2 (Day 7) Control: N=10, Mean±SD= 11.4±1.7 MOP: N=10, Mean±SD= 20.0±2.1 T3 (Day 28) Control: N=10, Mean±SD= 9.4±1.0 MOP: N=10, Mean±SD= 10.2±2.2  IL-8 (pg/mL) T0 (Before Rt) Control: N=10 , Mean±SD= 0.55±0.10 T1 (Day 1) Control: N=10, Mean±SD= 3.57±0.48 MOP: N=10, Mean±SD= 7.13±0.79 T2 (Day 7) Control: N=10, Mean±SD= 0.97±0.23 MOP: N=10, Mean±SD= 3.13±0.52 T3 (Day 28) Control: N=10, Mean±SD= 0.52±0.26 MOP: N=10, Mean±SD= 0.71±0.29  IL-1α (pg/mL) T0 (Before Rt) Control: N=10 , Mean±SD= 0.59±0.11 T1 (Day 1) Control: N=10, Mean±SD= 2.8±0.3 MOP: N=10, Mean±SD= 5.1±0.7  T2 (Day 7) Control: N=10, Mean±SD= 1.9±0.6 MOP: N=10, Mean±SD= 3.9±0.8 T3 (Day 28) Control: N=10, Mean±SD= 1.6±0.5 MOP: N=10, Mean±SD= 2.9±0.6  IL-1β (pg/mL) T0 (Before Rt) Control: N=10 , Mean±SD= 0.1±0.02 T1 (Day 1) Control: N=10, Mean±SD= 0.24±0.03 MOP: N=10, Mean±SD= 0.8±0.09 T2 (Day 7) Control: N=10, Mean±SD= 0.18±0.03 MOP: N=10, Mean±SD= 0.43±0.06 T3 (Day 28) Control: N=10, Mean±SD= 0.12±0.04 MOP: N=10, Mean±SD= 0.36±0.04  TNF-α (pg/mL) T0 (Before Rt) Control: N=10 , Mean±SD= 0.8±0.3 T1 (Day 1) Control: N=10, Mean±SD= 2.3±0.4 MOP: N=10, Mean±SD= 4.3±0.2 T2 (Day 7) Control: N=10, Mean±SD= 1.2±0.2 MOP: N=10, Mean±SD= 2.3±0.2 T3 (Day 28) Control: N=10, Mean±SD= 0.8±0.1 MOP: N=10, Mean±SD= 0.9±0.2  IL-6 (pg/mL) T0 (Before Rt) Control: N=10 , Mean±SD= 1.2±0.3 T1 (Day 1) Control: N=10, Mean±SD= 2.3±0.1 MOP: N=10, Mean±SD= 3.5±0.4 T2 (Day 7) Control: N=10, Mean±SD= 1.2±0.2 MOP: N=10, Mean±SD= 1.3±0.2 T3 (Day 28) Control: N=10, Mean±SD= 1.2±0.2 MOP: N=10, Mean±SD= 1.1±0.2;  Pain/discomfort:  T0: the day of canine retraction MOP: N=10, mean ± SD: 1.4 ± 0.2 Control: N=10, mean ± SD: 1.8 ± 0.3 T1: 24 hours after canine retraction MOP: N=10, mean ± SD: 3.1 ± 0.4 Control: N=10, mean ± SD: 3.4 ± 0.5 T2: 7 days after canine retraction MOP: N=10, mean ± SD: 2.2 ± 0.6 Control: N=10, mean ± SD: 2.1 ± 0.7  T3: 14 days after canine retraction MOP: N=10, mean ± SD: 1.4 ± 0.5 Control: N=10, mean ± SD: 1.6 ± 0.5 T4: 28 days after canine retraction MOP: N=10, mean ± SD: 1.2 ± 0.2 Control: N=10, mean ± SD: 1.1 ± 0.4 |
| 8.Alkebsi et al. (2018) | Change in canine movement from baseline (3D digital model measurements) over 3 months: (T1, Baseline to first month; T2, baseline to second month; T3, base line to third month) | Month 1 (T1):  Control: N=32, Mean ± SD = 0.67 ± 0.34  MOP: N=32, Mean ± SD = 0.65 ± 0.26  MD: 0.02 mm, 95% CI: (-0.13, 0.18), P = 0.77  Month 2 (T2)  Control: N=32, Mean ± SD = 1.28 ± 0.50  MOP: N=32, Mean ± SD = 1.36 ± 0.49  MD: -0.08 mm, 95% CI: (-0.33, 0.16), P = 0.50  Month 3 (T3)  Control: N=32, Mean ± SD = 1.88 ± 0.67  MOP: N=32, Mean ± SD = 1.93 ± 0.74  MD: -0.05 mm, 95% CI: (-0.40, 0.29), P = 0.76  Intraoral measurements of canine movement:  T1: Control: N=32, Mean ± SD = 1.17 ± 0.42  MOP: N=32, Mean ± SD =1.23 ± 0.45  MD: -0.06 mm, 95% CI: (-0.28, 0.15), P = 0.56  T2: Control: N=32, Mean ± SD = 2.24 ± 0.63  MOP: N=32, Mean ± SD = 2.27 ± 0.72  MD: -0.03 mm, 95% CI: (-0.36, 0.31), P = 0.88  T3: Control: N=32, Mean ± SD = 3.26 ± 0.81  MOP: N=32, Mean ± SD = 3.18 ± 1.03  MD: 0.07 mm, 95% CI: (-0.39, 0.54), P = 0.74 | 1.Periodontal health: Gingival index and plaque index evaluated at baseline and 3 months. No significant differences between MOP and control sides for either index at any time point.  2.Pain/discomfort  3.Root resorption:  4.Anchorage loss  5.Canine tipping and rotation | Gingival index and plaque index: means, standard deviations, and results of independent t tests T0: gingival index: Control: Mean ± SD = 1.38 ± 0.55 MOP: Mean ± SD = 1.44 ± 0.56 p value= 0.65 plaque index: control: Mean ± SD = 1.25 ± 0.51 MOP: Mean ± SD = 1.28 ± 0.52 p value=0.81  T3: gingival index: control: Mean ± SD = 1.63 ± 0.49 MOP: Mean ± SD = 1.50 ± 0.51 p value= 0.31 plaque index: control: Mean ± SD = 1.27 ± 0.45 MOP: Mean ± SD = 1.27 ± 0.45 p value=1.000;  ,  Pain/discomfort: Pain level was higher in MOP side immediately, 1h, 12h, 24h, but not statistically significant (P>0.05). Pain interference with daily life (eating, sleep, discomfort, swelling) was assessed. Significant difference only for feeling of swelling on day 1 on MOP side (P=0.05). Level of satisfaction was high (mean 8.94/10), ease of procedure was rated as easy (mean 2.71/10, 0=very easy). Pain Perception (VAS 0-10) N (Control) = N (MOP) = 32 Immediate: Control: Mean ± SD = 1.0 ± 1.99 MOP: Mean ± SD = 1.4 ± 1.74 P = 0.388  1 Hour: Control: Mean ± SD = 1.5 ± 2.23 MOP: Mean ± SD = 2.4 ± 2.15 P = 0.092  12 Hours: Control: Mean ± SD = 1.6 ± 2.35 MOP: Mean ± SD = 2.6 ± 2.79 P = 0.100  Day 1: Control: Mean ± SD = 0.8 ± 1.36 MOP: Mean ± SD = 1.2 ± 1.95 P = 0.302  Day 3: Control: Mean ± SD = 0.6 ± 1.29 MOP: Mean ± SD = 0.8 ± 1.41 P = 0.582  Day 5: Control: Mean ± SD = 0.3 ± 1.18 MOP: Mean ± SD = 0.4 ± 1.10 P = 0.743  Day 7: Control: Mean ± SD = 0.3 ± 1.28 MOP: Mean ± SD = 0.2 ± 0.71 P = 0.809  Root resorption: Significant root resorption in both MOP and control groups from baseline to 3 months, but no statistically significant difference between MOP and control sides at baseline or 3 months. Root resorption measured using periapical radiographs. Root resorption: means, standard deviations, and 95% confidence intervals between MOP and control sides at baseline and after third month:  Root Length (mm) Baseline (T0): Control: N = 32, Mean ± SD = 19.66 ± 2.5 MOP: N = 32, Mean ± SD = 19.66 ± 2.2 MD: -0.31 mm (As reported, likely representing MOP - Control) 95% CI: (-1.4, 0.8) P = 0.59  3 Months (T3): Control: N = 32, Mean ± SD = 18.93 ± 2.6 MOP: N = 32, Mean ± SD = 19.35 ± 2.0 MD: -0.42 mm (As reported, likely representing MOP - Control) 95% CI: (-1.6, 0.7) P = 0.48  Anchorage loss: No statistically significant difference in anchorage loss between MOP and control sides at months 1, 2, or 3. Anchorage loss was considered not clinically significant in both groups (<1.5mm). Anchorage Loss (mm) T1 (Month 1): Control: Mean ± SD = 0.08 ± 0.26 MOP: Mean ± SD = 0.16 ± 0.30 MD: 0.08 mm (As reported in Table IV, representing Control - MOP) 95% CI: (-0.22, 0.07) P = 0.29  T2 (Month 2): Control: Mean ± SD = 0.26 ± 0.37 MOP: Mean ± SD = 0.22 ± 0.31 MD: 0.04 mm (As reported in Table IV, representing Control - MOP) 95% CI: (-0.13, 0.21) P = 0.62  T3 (Month 3): Control: Mean ± SD = 0.39 ± 0.54 MOP: Mean ± SD = 0.35 ± 0.43 MD: 0.03 mm (As reported in Table IV, representing Control - MOP) 95% CI: (-0.20, 0.28) P = 0.77  Canine tipping and rotation: No statistically significant differences between MOP and control sides. High percentage of patients willing to repeat MOP (91.2%) and recommend it (94.1%).Canine Tipping (mm)  Tipping Measurement: T-M (Difference of canine displacement from the tip of the canines to the middle of the canine lateral surface): Control: Mean ± SD = 0.25 ± 0.26 MOP: Mean ± SD = 0.25 ± 0.23 MD: 0.001 mm (As reported in Table V, representing Control - MOP) 95% CI: (-0.12, 0.12) P = 0.99  Tipping Measurement: G-M (Difference of canine displacement from the middle of the canine gingival margin to the middle of the canine lateral surface): Control: Mean ± SD = 0.01 ± 0.19 MOP: Mean ± SD = -0.001 ± 0.16 (Note: In the original table it is “-0.001”, I’m keeping it as reported) MD: 0.012 mm (As reported in Table V, representing Control - MOP) 95% CI: (-0.07, 0.10) P = 0.78 Canine Rotation (°)  T0 (Baseline): Control: Mean ± SD = 33.8 ± 9.6 MOP: Mean ± SD = 32.0 ± 6.6 MD: 1.8° (As reported in Table VI, representing Control - MOP) 95% CI: (-2.3, 5.9) P = 0.38  T3 (3 Months): Control: Mean ± SD = 25.4 ± 5.1 MOP: Mean ± SD = 24.6 ± 5.6 MD: 0.8° (As reported in Table VI, representing Control - MOP) 95% CI: (-1.8, 3.5) P = 0.55 |
| 9.Alqadasi et al. (2019) | The distance between canine and second premolar (mm) (The distance from the canine cusp tip to a perpendicular line extending from the buccal cusp tip of the second premolar to the midline) | T0 (before Exp)  MOP: N=8, Mean= 10.83, SD=1.25  Control: N=8, Mean= 10.96, SD=0.76  P-value: 0.808  T1 (2weeks)  MOP: N=8, Mean= 10.32, SD=1.18  Control: N=8, Mean= 10.85, SD=1.06  P-value: 0.545  T2 (1month)  MOP: N=8, Mean= 9.72, SD=1.26  Control: N=8, Mean= 9.79, SD=0.67  P-value: 0.818  T3 (2month)  MOP: N=8, Mean= 9.10, SD=0.89  Control: N=8, Mean= 9.09, SD=1.08  P-value: 0.945  T4 (3month)  MOP: N=8, Mean= 8.70, SD=1.21  Control: N=8, Mean= 9.28, SD= 1.23  P-value: 0.002  post hoc test  (in the following reports confidence intervals (CI), specifically the lower limit (LL) and upper limit (UL), have not been mentioned)  Refrence point: T2(1 month)  MOP:  After 2 months (T3): MD= 0.6225, SE= 0.58230, sig= 0.292  After 2 weeks (T1): MD= -0.5987, SE= 0.58230, sig= 0.311  After 3 months (T4): MD= 1.0225, SE= 0.58230, sig= 0.088  Before Exp (T0): MD= -1.1100, SE= 0.58230, sig= 0.065  Control:  After 2 months (T3): MD= 0.6225, SE= 0.58230, sig= 0.292  After 2 weeks (T1): MD= -0.5987, SE= 0.58230, sig= 0.311  After 3 months (T4): MD= 1.0225, SE= 0.58230, sig= 0.088  Before Exp (T0): MD= -1.1100, SE= 0.58230, sig= 0.065  Refrence point: T3(2 month)  MOP:  After 1 month (T2): MD= -0.6225, SE= 0.58230, sig= 0.292  After 2 weeks (T1): MD= -1.2212, SE= 0.58230, sig= 0.043  After 3 months (T4): MD= 0.4000, SE= 0.58230, sig= 0.497  Before Exp (T0): MD= -1.7325, SE= 0.58230, sig= 0.005  Control:  After 1 month (T2): MD= -0.6225, SE= 0.58230, sig= 0.292  After 2 weeks (T1): MD= -1.2212, SE= 0.58230, sig= 0.043  After 3 months (T4): MD= 0.4000, SE= 0.58230, sig= 0.497  Before Exp (T0): MD= -1.7325, SE= 0.58230, sig= 0.005  Refrence point: T1(2 weeks)  MOP:  After 1 month (T2): MD= 0.5987, SE= 0.58230, sig= 0.311  After 2 months (T3): MD= 1.2212, SE= 0.58230, sig= 0.043  After 3 months (T4): MD= 1.6212, SE= 0.58230, sig= 0.009  Before Exp (T0): MD= -0.5113, SE= 0.58230, sig= 0.386  Control:  After 1 month (T2): MD= 0.5987, SE= 0.58230, sig= 0.311  After 2 months (T3): MD= 1.2212, SE= 0.58230, sig= 0.043  After 3 months (T4): MD= 1.6212, SE= 0.58230, sig= 0.009  Before Exp (T0): MD= -0.5113, SE= 0.58230, sig= 0.386  Refrence point: T4(3 months)  MOP:  After 1 month (T2): MD= -1.0225, SE= 0.58230, sig= 0.088  After 2 months (T3): MD= -0.4000, SE= 0.58230, sig= 0.497  After 2 weeks (T1): MD= -1.6212, SE= 0.58230, sig= 0.009  Before Exp (T0): MD= -2.1325, SE= 0.58230, sig= 0.001  Control:  After 1 month (T2): MD= -1.0225, SE= 0.58230, sig= 0.088  After 2 months (T3): MD= -0.4000, SE= 0.58230, sig= 0.497  After 2 weeks (T1): MD= -1.6212, SE= 0.58230, sig= 0.009  Before Exp (T0): MD= -2.1325, SE= 0.58230, sig= 0.001  Refrence point: T0(Before Exp)  MOP:  After 1 month (T2): MD= 1.1100, SE= 0.58230, sig= 0.065  After 2 months (T3): MD= 1.7325, SE= 0.58230, sig= 0.005  After 2 weeks (T1): MD= 0.5113, SE= 0.58230, sig= 0.386  After 3 months (T4): MD= 2.1325, SE= 0.58230, sig= 0.001  Control:  After 1 month (T2): MD= 1.1100, SE= 0.58230, sig= 0.065  After 2 months (T3): MD= 1.7325, SE= 0.58230, sig= 0.005  After 2 weeks (T1): MD= 0.5113, SE= 0.58230, sig= 0.386  After 3 months (T4): MD= 2.1325, SE= 0.58230, sig= 0.001 | 1.Root resorption: was assessed by measuring the total canine length, determined by the distance between points on the crown tip and root apex.  2:Bone height: was measured as the distance between the cementoenamel junction and the marginal bone crest on the buccal and lingual sides, using lines drawn between these points. Although a figure in the article displays the mean and standard deviation (SD), the data presented does not correspond to the context of the study. The SD for the specific results discussed in the text has not been reported. | Root resorption:  post hoc test T0: MOP: N=8 Mean: 22.71, SD: 0.71 Control: N=8, Mean: 22.76, SD: 1.06 P-value: 0.842  T4 (3months): MOP: N=8 Mean: 22.68, SD: 0.71 Control: N=8, Mean: 22.71, SD: 1.14 P-value: 0.886  T0-T4: MOP: P-value:0.934 Control: P-value: 0.929;  Bone height  MOP: N=8, Mean(buccal)= 0.11  N=8, Mean(palatal)= 0.23 P-value: 1.00  Control: N=8, Mean(buccal)= 0.1 N=8, Mean(palatal)= 0.05 P-value: 0.393 |
| 10. Alqadasi et al. (2024) | Canine Retraction Measurements (Mean (SD) in mm): | Baseline:  Control: N=10, Mean (SD) = 10.95 (0.76), p value=0.521  MOP: N=10, Mean (SD) = 10.66 (1.19), p value=0.368  2 Weeks:  Control: N=10, Mean (SD) = 10.58 (1.02), p value= 0.656  MOP:N=10, Mean (SD) = 10.16 (1.18), p value= 0.927  1 Month:  Control: N=10, Mean (SD) = 9.8 (0.63), p value= 0.352  MOP: N=10, Mean (SD) = 9.59 (1.21), p value= 0.311  2 Months:  Control: N=10, Mean (SD) = 9.09 (1.14), p value= 0.560  Experimental: N=10, Mean (SD) = 9.02 (0.86), p value= 0.955  3 Months:  Control: N=10, Mean (SD) = 9.22 (1.26), p value= 0.876  MOP: N=10, Mean (SD) = 8.61 (1.19)* , p value= 0.966  Net movement (3 Months - Baseline) (Mean (SD) in mm):  Control: N=10, Mean (SD) = -1.73 (0.96), p value= 0.428  MOP: N=10, Mean (SD) = -2.04 (0.98), p value= 0.239  Overall net movement (Experimental - Control) (Mean (SD) in mm):  MOP: Mean (SD) = 0.32 (1.14), p value= 0.606  NOTE: Baseline Canine Retraction” is NOT about actual retraction happening at baseline. It’s about measuring the starting position of the canine before the canine retraction force and acceleration techniques (MOPs) are applied.  NOTE: *: P < 0.001 (the paired samples t-test). The remaining tests were conducted using the independent-samples t-test. | Root resorption: Root resorption assessed using CBCT. No significant differences in root resorption between experimental and control sides. | Root resorption  Baseline: Control: Mean (SD)= 22.84 (0.6), p value= 0.05 MOP: Mean (SD)= 22.76 (0.63), p value=0.007  Final: Control: Mean (SD)= 22.79 (1.03), p value= 0.245 MOP: Mean (SD)= 22.72 (0.64), p value=0.006  Net change: Control: Mean (SD)= −0.06 (0.09), p value= 0.116 MOP: Mean (SD)= −0.04 (0.04), p value= 0.092  Overall change: Mean (SD)= 0.016 (0.1) , p value= 0.087 net change in mm = Final measurement - Baseline measurement; Overall change in mm = Experimental – Controls |
| 11.Attri et al. (2018) | Monthly rate of space closure (mm/month)  (The distance between two perpendicular lines drawn to the mesial surface of the second premolar and the distal surface of the canine from the mid-palatine line was considered as tooth movement) | Right side maxilla  MOP: n=30, mean (SD): 0.89 (0.17)  Control: n=30, mean(SD): 0.63 (0.11)  mean difference: 0.25, SE: 0.037, CI (LL: 0.18 UL: 0.33), P-value:0.000  Left side maxilla  MOP: n=30, mean (SD): 0.88 (0.21)  Control: n=30, mean(SD): 0.53 (0.19)  mean difference: 0.35, SE: 0.052, CI (LL: 0.25 UL: 0.45), P-value:0.000  Right side mandible  MOP: n=30, mean (SD): 0.80 (0.19)  Control: n=30, mean(SD): 0.53 (0.1)  mean difference: 0.27, SE: 0.039, CI (LL: 0.19 UL: 0.35), P-value:0.000  Left side mandible  MOP: n=30, mean (SD): 0.73 (0.1)  Control: n=30, mean(SD): 0.49 (0.1)  mean difference: 0.24, SE: 0.033, CI (LL: 0.17 UL: 0.31), P-value:0.000 | Pain/discomfort: Pain perception was recorded using a 10 mm visual analogue scale (VAS) at three different time intervals. | Median pain scores: MOP: N=30  T0 (24hours): Median=1 , IQR=0.25  T1 (7days): Median=1 , IQR=0  T2 (28days): Median=0 , IQR=0  Statistic=0.505 , P-value=0.609 Control: N/A |
| 12.Babanouri et al. (2020) | Incisal canine movement (mean ± SD)  Vertical reference lines were drawn on the palatal surface of the canine and lateral incisor, and distances were measured at the incisal, middle, and cervical thirds of the crowns. | Incisal canine movement (mean ± SD):  T1(Day 28):  Mop1: n=12 mean= 0.94 ± 0.31  Mop2: n=13 mean= 1.21 ± 0.20  Control: n=25 mean= 0.64 ± 0.12  T2(Day 56):  Mop1: n=12 mean= 0.86 ± 0.19  Mop2: n=13 mean= 1.14 ± 0.18  Control: n=25 mean= 0.66 ± 0.16  T3(Day 84):  Mop1: n=12 mean= 0.76 ± 0.10  Mop2: n=13 mean= 1.0 ± 0.15  Control: n=25 mean= 0.73 ± 0.12  Middle canine movement (mean ± SD):  T1(Day 28):  Mop1: n=12 mean= 0.88 ± 0.26  Mop2: n=13 mean= 1.18 ± 0.20  Control: n=25 mean= 0.61 ± 0.12  T2(Day 56):  Mop1: n=12 mean= 0.86 ± 0.19  Mop2: n=13 mean= 1.11 ± 0.18  Control: n=25 mean= 0.62 ± 0.12  T3(Day 84):  Mop1: n=12 mean= 0.76 ± 0.10  Mop2: n=13 mean= 1.08 ± 0.15  Control: n=25 mean= 0.68 ± 0.12  Cervical canine movement (mean ± SD):  T1(Day 28):  Mop1: n=12 mean= 0.83 ± 0.24  Mop2: n=13 mean= 1.16 ± 0.19  Control: n=25 mean= 0.59 ± 0.11  T2(Day 56):  Mop1: n=12 mean= 0.79 ± 0.16  Mop2: n=13 mean= 1.09 ± 0.19  Control: n=25 mean= 0.61 ± 0.12  T3(Day 84):  Mop1: n=12 mean= 0.66 ± 0.12  Mop2: n=13 mean= 1.08 ± 0.21  Control: n=25 mean= 0.66 ± 0.10  …………………………………………………………  The B-coefficient represents the estimated change in canine movement for each unit change in time or group, indicating the strength and direction of the relationship. A positive value means increased movement, while a negative value means decreased movement compared to the reference.  Beta coefficient of the canine movement:  T1(Day 28): reference  Incial:  T2(Day 56):  Mop1: n=12 B: − 0.08 (95% CI of B: − 0.21, 0.05) p-value= 0.209  Mop2: n=13 B: − 0.07 (95% CI of B: − 0.19, 0.05) p-value= 0.245  Control: n=25 B: 0.02 (95% CI of B: − 0.03, 0.07) p-value= 0.471  T3(Day 84):  Mop1: n=12 B: − 0.21 (95% CI of B: − 0.37, − 0.05) p-value= 0.012  Mop2: n=13 B: − 0.12 (95% CI of B: − 0.22, − 0.01) p-value= 0.034  Control: n=25 B: 0.08 (95% CI of B: 0.03, 0.13) p-value= 0.002  Middle:  T2(Day 56):  Mop1: n=12 B: − 0.05 (95% CI of B: − 0.16, 0.05) p-value= 0.269  Mop2: n=13 B: − 0.06 (95% CI of B: − 0.18, 0.05) p-value= 0.282  Control: n=25 B: 0.00 (95% CI of B: − 0.05, 0.06) p-value= 0.876  T3(Day 84):  Mop1: n=12 B: − 0.18 (95% CI of B: − 0.29, − 0.06) p-value= 0.005  Mop2: n=13 B: − 0.09 (95% CI of B: − 0.19, 0.00) p-value= 0.06  Control: n=25 B: 0.07 (95% CI of B: 0.02, 0.11) p-value= 0.004  Cervical:  T2(Day 56):  Mop1: n=12 B: − 0.03 (95% CI of B: − 0.14, 0.08) p-value= 0.548  Mop2: n=13 B: − 0.07 (95% CI of B: − 0.18, 0.04) p-value= 0.205  Control: n=25 B: 0.02 (95% CI of B: − 0.01, 0.06) p-value= 0.255  T3(Day 84):  Mop1: n=12 B: − 0.16 (95% CI of B: − 0.27, − 0.04) p-value= 0.011  Mop2: n=13 B: − 0.08 (95% CI of B: − 0.18, 0.01) p-value= 0.099  Control: n=25 B: 0.06 (95% CI of B: 0.02, 0.10) p-value= 0.002  Control :refrence  Incial:  T1(Day 28):  Mop1: n=12 B: 0.29 (95% CI of B: 0.15, 0.44) p-value < 0.001  Mop2: n=13 B: 0.57 (95% CI of B: 0.43, 0.71) p-value < 0.001  T2(Day 56):  Mop1: n=12 B: 0.19 (95% CI of B: 0.07, 0.31) p-value = 0.002  Mop2: n=13 B: 0.47 (95% CI of B: 0.36, 0.59) p-value < 0.001  T3(Day 84):  Mop1: n=12 B: − 0.00 (95% CI of B: − 0.09, 0.09) p-value =0.993  Mop2: n=13 B: 0.36 (95% CI of B: 0.27, 0.45) p-value < 0.001  Middle:  T1(Day 28):  Mop1: n=12 B: 0.26 (95% CI of B: 0.13, 0.39) p-value < 0.001  Mop2: n=13 B: 0.56 (95% CI of B: 0.43, 0.69) p-value < 0.001  T2(Day 56):  Mop1: n=12 B: 0.20 (95% CI of B: 0.10, 0.31) p-value < 0.001  Mop2: n=13 B: 0.49 (95% CI of B: 0.39, 0.60) p-value < 0.001  T3(Day 84):  Mop1: n=12 B: 0.01 (95% CI of B: − 0.08, 0.10) p-value =0.790  Mop2: n=13 B: 0.39 (95% CI of B: 0.30, 0.49) p-value < 0.001  Cervical:  T1(Day 28):  Mop1: n=12 B: 0.23 (95% CI of B: 0.11, 0.35) p-value < 0.001  Mop2: n=13 B: 0.56 (95% CI of B: 0.45, 0.68) p-value < 0.001  T2(Day 56):  Mop1: n=12 B: 0.18 (95% CI of B: 0.06, 0.29) p-value = 0.002  Mop2: n=13 B: 0.47 (95% CI of B: 0.36, 0.58) p-value < 0.001  T3(Day 84):  Mop1: n=12 B: 0.00 (95% CI of B: − 0.08, 0.09) p-value =0.879  Mop2: n=13 B: 0.41 (95% CI of B: 0.33, 0.50) p-value < 0.001 | Pain/discomfort: Pain and discomfort were assessed using visual analog scales (VAS) for all subjects at T0, T1 (after interventions). The meanings of the VAS scores were as follows: no pain “0”, severe pain “10”; very comfortable “0”, very uncomfortable “10” | VAS Scores at T0, T1 for Pain and discomfort After interventions at T0: MOP1: N=12, Mean: 3.66, SD: 2.34 MOP2: N=13, Mean: 3.61, SD: 2.59 Control: N=25, Mean: 2.96, SD:2.26  T1(24 Hours after T0): MOP1: N=12, Mean: 1.75, SD: 2.22 MOP2: N=13, Mean: 1.53, SD: 1.85 Control: N=25, Mean: 1.40, SD:1.50 |
| 13.Bansal et al. (2019) | Intergroup Comparison of Amount of Crowding in Mandibular Incisors at Various Time Intervals | T0 (Baseline):; Experimental: N=15, Mean: 5.31, SD: 0.3615; Control: N=15, Mean: 5.35, SD: 0.3046; Mean Difference: −0.04 (95% CI: −0.29, 0.21); P-value: 0.729 (Not Significant); T1 (After 3 weeks):; Experimental: N=15, Mean: 2.76, SD: 0.5681; Control: N=15, Mean: 4.14, SD: 0.3469; Mean Difference: −1.38 (95% CI: −1.732, −1.028); P-value: 0.000 (Highly Significant); T2 (After 6 weeks):; Experimental: N=15, Mean: 1.08, SD: 0.5053; Control: N=15, Mean: 3.08, SD: 0.4579; Mean Difference: −2 (95% CI: −2.361, −1.639); P-value: 0.000 (Highly Significant); T3 (After 9 weeks):; Experimental: N=15, Mean: 0.50, SD: 0.200; Control: N=15, Mean: 1.91, SD: 0.4319; Mean Difference: −1.41 (95% CI: −1.662, −1.158); P-value: 0.000 (Highly Significant); T4 (After 12 weeks):; Experimental: N=15, Mean: Not Available; Control: N=15, Mean: 0.93, SD: 0.2611; T5 (After 15 weeks):; Experimental: N=15, Mean: Not Available; Control: N=15, Mean: 0.38, SD: 0.0449; (T0-T1)/T0 (Baseline to 3 Weeks):; Experimental: 48.14 ± 9.10%; Control: 22.59 ± 3.64%; P-value: 0.000 (Highly Significant); Percentage Change: 113.10%; Times Faster: 2.13; (T0-T2)/T0 (Baseline to 6 Weeks):; Experimental: 79.93 ± 8.51%; Control: 42.30 ± 8.35%; P-value: 0.000 (Highly Significant); Percentage Change: 88.95%; Times Faster: 1.88; (T0-T3)/T0 (Baseline to 9 Weeks):; Experimental: 91.07 ± 3.49%; Control: 64.09 ± 8.1%; P-value: 0.000 (Highly Significant); Percentage Change: 42.09%; Times Faster: 1.42; T0 = Baseline; T1 = After 3 weeks; T2 = After 6 weeks; T3 = After 9 weeks | Periodontal health: The marginal bone levels assessed by CBCT: The marginal bone height loss and volumetric root resorption around mandibular anterior teeth were assessed using CBCT imaging before treatment and after six months. Limited Field of View (5 × 5 cm) CBCT scans were performed with a Carestream CS-9300 imaging system (90 μm voxel size, 80-84 kVp tube voltage, 5-8 mA tube current, and 20-second exposure). DICOM data were processed using Invivo 5.2 software (Anatomage, San Jose, CA, USA) on Microsoft Windows 10. Non-relevant regions were manually segmented and removed, isolating mandibular incisors. The crown and root of each incisor were segmented, and root volumes were calculated using a volume determination tool. An AI-based threshold range ensured standardization, and volumetric root resorption was determined by subtracting pretreatment measurements from post-alignment measurements.  Pain/discomfort: Pain was assessed using a Visual Analog Scale (VAS) from 0 to 100 mm at various time points after appliance placement. Statistically significant differences in VAS scores were observed at 12 and 24 hours, with higher pain levels in the experimental group (P < 0.05, P < 0.01). No significant differences were found at 4 hours, the 3rd day, or between males and females. Pain returned to baseline by the 7th day, and no patients in the MOP group reported using anti-inflammatory medication. Patient satisfaction and discomfort levels showed no significant intergroup differences (P > 0.05). (SD Need to be accessed with contacting its authors)  Root resorption: Root resorption has been assessed volumetric CBCT evaluation: The marginal bone height loss and volumetric root resorption around mandibular anterior teeth were assessed using CBCT imaging before treatment and after six months. Limited Field of View (5 × 5 cm) CBCT scans were performed with a Carestream CS-9300 imaging system (90 μm voxel size, 80-84 kVp tube voltage, 5-8 mA tube current, and 20-second exposure). DICOM data were processed using Invivo 5.2 software (Anatomage, San Jose, CA, USA) on Microsoft Windows 10. Non-relevant regions were manually segmented and removed, isolating mandibular incisors. The crown and root of each incisor were segmented, and root volumes were calculated using a volume determination tool. An AI-based threshold range ensured standardization, and volumetric root resorption was determined by subtracting pretreatment measurements from post-alignment measurements. | Intergroup Comparison of Marginal Bone Loss: Tooth 43 (M): Experimental: Mean: 0.206 mm, SD: 0.2763 Control: Mean: 0.093 mm, SD: 0.0961 P-value: 0.145 (Not Significant) Mean Difference: 0.113 mm (95% CI: −0.041, 0.267) Tooth 42 (D): Experimental: Mean: 0.133 mm, SD: 0.1496 Control: Mean: 0.093 mm, SD: 0.1438 P-value: 0.461 (Not Significant) Mean Difference: 0.04 mm (95% CI: −0.069, 0.149) Tooth 41 (M): Experimental: Mean: 0.040 mm, SD: 0.1056 Control: Mean: 0.093 mm, SD: 0.0961 P-value: 0.159 (Not Significant) Mean Difference: −0.053 mm (95% CI: −0.128, 0.022) Tooth 31 (M): Experimental: Mean: 0.053 mm, SD: 0.1060 Control: Mean: 0.020 mm, SD: 0.1474 P-value: 0.483 (Not Significant) Mean Difference: −0.033 mm (95% CI: −0.063, 0.129) Tooth 32 (D): Experimental: Mean: 0.060 mm, SD: 0.1502 Control: Mean: 0.153 mm, SD: 0.1506 P-value: 0.100 (Not Significant) Mean Difference: −0.093 mm (95% CI: −0.205, 0.019) Tooth 33 (M): Experimental: Mean: 0.147 mm, SD: 0.2416 Control: Mean: 0.100 mm, SD: 0.1604 P-value: 0.538 (Not Significant) Mean Difference: 0.047 mm (95% CI: −0.106, 0.200) Notes: P ≥ 0.05 indicates no significant difference. Measurements are presented as mean marginal bone loss in mm with standard deviation (SD) and confidence intervals (CI).;  Pain/discomfort:  Time intervals: Immediately:  Experimental: 0 mm Control: 0 mm At 4 hours:  Experimental: 23.4 mm Control: 18.13 mm At 12 hours:  Experimental: 45.07 mm Control: 32.13 mm At 24 hours:  Experimental: 23.2 mm Control: 14.93 mm At 3rd day:  Experimental: 8.6 mm Control: 8.6 mm At 7th day:  Experimental: 0 mm Control: 0 mm  Intergroup Comparison of Volumetric Root Resorption: • Tooth number: 42: o Experimental: Mean: 16.87 mm³, SD: 6.368 o Control: Mean: 14.67 mm³, SD: 5.851 o P-value: 0.333 (Not Significant) o Mean Difference: 2.2 mm³ (95% CI: −2.278, 6.678) • Tooth number: 41: o Experimental: Mean: 16.53 mm³, SD: 6.685 o Control: Mean: 14.53 mm³, SD: 7.019 o P-value: 0.431 (Not Significant) o Mean Difference: 2 mm³ (95% CI: −3.127, 7.127) • Tooth number: 31: o Experimental: Mean: 16.27 mm³, SD: 6.076 o Control: Mean: 15.80 mm³, SD: 6.461 o P-value: 0.840 (Not Significant) o Mean Difference: 0.47 mm³ (95% CI: −4.221, 5.161) • Tooth number: 32: o Experimental: Mean: 15.40 mm³, SD: 5.475 o Control: Mean: 13.80 mm³, SD: 5.321 o P-value: 0.424 (Not Significant) o Mean Difference: 1.6 mm³ (95% CI: −2.438, 5.638) Notes: • P ≥ 0.05 indicates no significant difference. • Measurements are presented as mean volumetric root resorption in mm³ with standard deviation (SD) and confidence intervals (CI). |
| 14.Bavikati et al. (2022) | Rate of Tooth Movement | MOP: N=22  At 45 Days:  Mean = 10.31 mm, SD = 0.55 mm  At Completion of Retraction:  Mean = 12.92 mm, SD = 0.30 mm  Total Movement (T0 to Completion):  Mean = 5.14 mm (12.92 - 7.78), SD = Not explicitly reported for total change.  Control: N=22  At 45 Days:  Mean = 8.89 mm, SD = 0.48 mm  At Completion of Retraction:  Mean = 10.31 mm, SD = 0.46 mm  Total Movement (T0 to Completion):  Mean = 2.63 mm (10.31 - 7.68), SD = Not explicitly reported for total change.. | 1.Periodontal health: Interleukin-1β (IL-1β) Levels in Gingival Crevicular Fluid (GCF)  2.Pain/discomfort | Periodontal health:  MOP: N=22 Baseline: Mean = 0.170 pg/μL, SD = 0.06 pg/μL  At 3 Days: Mean = 0.750 pg/μL, SD = 0.09 pg/μL  At 7 Days: Mean = 0.510 pg/μL, SD = 0.07 pg/μL  Control: N=22  Baseline: Mean = 0.150 pg/μL, SD = 0.05 pg/μL  At 3 Days: Mean = 0.280 pg/μL, SD = 0.07 pg/μL  At 7 Days: Mean = 0.150 pg/μL, SD = 0.05 pg/μL;  Pain/discomfort: Reported no post-operative discomfort or pain |
| 15.El awady et al. (2019) | Rate of Canine Movement (mm/month) | Control Group (mm/month):  Month T1-T2: N=12, Mean: 1.10, SD: 0.51, SE: 0.15  Month T2-T3: N=12, Mean: 1.32, SD: 0.45, SE: 0.13  Month T3-T4: N=12, Mean: 1.18, SD: 0.43, SE: 0.13  Month T4-T5: N=12, Mean: 1.21, SD: 0.45, SE: 0.13  Month T5-T6: N=12, Mean: 1.25, SD: 0.27, SE: 0.13  MOPs Group (mm/month):  Month T1-T2: N=12, Mean: 1.51, SD: 0.50, SE: 0.14  Month T2-T3: N=12, Mean: 1.31, SD: 0.41, SE: 0.13  Month T3-T4: N=12, Mean: 1.36, SD: 0.44, SE: 0.13  Month T4-T5: N=12, Mean: 1.07, SD: 0.29, SE: 0.10  Month T5-T6: N=12, Mean: 0.90, SD: 0.05, SE: 0.02  Month T6-T7: N=12, Mean: 0.88, SD: 0.32, SE: 0.14  Test Values and Significance:  T1-T2: P = 0.059 (NS)  T2-T3: P = 0.970 (NS)  T3-T4: P = 0.367 (NS)  T4-T5: P = 0.427 (NS)  T5-T6: P = 0.101 (NS)  T6-T7: P = 0.534 (NS)  Total Rate of Canine Movement (mm/month):  MOPs Group: N=12, Mean: 1.59, SD: 0.49  Control Group: N=12, Mean: 1.50, SD: 0.42  P-value: 0.621 (Not Significant) |  |  |
| 16.Farag et al. (2021) |  | T0:  MOP: N: 10, mean of D (distance between canine tip and MB tip of first molar): 20.47 (SD: 1.04)  Control: N: 10, mean of D: 20.47 (SD: 1.04)  T1 (2 weeks):  MOP: N: 10, mean of D (distance between canine tip and MB tip of first molar): 19.17 (SD: 1.05)  Control: N: 10, mean of D: 19.85 (SD: 1.07), p-value < 0.001  T2 (4 weeks):  MOP: N: 10, mean of D (distance between canine tip and MB tip of first molar): 18.35 (SD: 1.02)  Control: N: 10, mean of D: 19.16 (SD: 1.12), p-value < 0.001  T3 (6 weeks):  MOP: N: 10, mean of D (distance between canine tip and MB tip of first molar): 17.62 (SD: 1.04)  Control: N: 10, mean of D: 18.70 (SD: 1.09), p-value < 0.001  T4 (8 weeks):  MOP: N: 10, mean of D (distance between canine tip and MB tip of first molar): 17.17 (SD: 1.00)  Control: N: 10, mean of D: 18.52 (SD: 1.13), p-value < 0.001  T5 (10 weeks):  MOP: N: 10, mean of D (distance between canine tip and MB tip of first molar): 16.73 (SD: 1.05)  Control: N: 10, mean of D: 17.96 (SD: 1.15), p-value < 0.001  T6 (12 weeks):  MOP: N: 10, mean of D (distance between canine tip and MB tip of first molar): 16.30 (SD: 1.08)  Control: N: 10, mean of D: 17.64 (SD: 1.08), p-value < 0.001 |  |  |
| 17.Fattori et al. (2020) | Space Remaining (mm) | Space Remaining (mm):  S1:  Control Group: N=9, Mean = 3.5, SD = 1.7, Minimum = 2.1, Maximum = 7.5  MOP: N=9, Mean = 5.4, SD = 1.5, Minimum = 3.2, Maximum = 7.4  S2:  Control Group: N=9, Mean = 3.0, SD = 1.7, Minimum = 1.7, Maximum = 6.9  MOP: N=9, Mean = 4.8, SD = 1.5, Minimum = 2.4, Maximum = 6.7  S3:  Control Group: N=9, Mean = 2.3, SD = 1.5, Minimum = 0.7, Maximum = 5.8  MOP: N=9, Mean = 4.1, SD = 1.4, Minimum = 2.1, Maximum = 6.3  S6:  Control Group: N=9, Mean = 0.9, SD = 1.3, Minimum = 0.0, Maximum = 3.2  MOP: N=9, Mean = 2.6, SD = 1.2, Minimum = 1.2, Maximum = 4.8  S9:  Control Group: N=9, Mean = 1.8, SD = 0.5, Minimum = 1.4, Maximum = 2.4  MOP: N=9, Mean = 1.3, SD = 0.7, Minimum = 0.0, Maximum = 2.3  Bonferroni Multiple Comparisons for Average Tooth Movement Values (Space Remaining)  Control Group:  S1 vs S2: Mean Difference = 0.54, Standard Error = 0.13, Degrees of Freedom = 1, P-Value = .002, 95% CI [0.11, 0.97]  S1 vs S3: Mean Difference = 1.18, Standard Error = 0.19, Degrees of Freedom = 1, P-Value = <.001, 95% CI [0.57, 1.79]  S1 vs S6: Mean Difference = 2.75, Standard Error = 0.23, Degrees of Freedom = 1, P-Value = <.001, 95% CI [2.00, 3.50]  S1 vs S9: Mean Difference = 3.31, Standard Error = 0.32, Degrees of Freedom = 1, P-Value = <.001, 95% CI [2.26, 4.35]  S2 vs S3: Mean Difference = 0.64, Standard Error = 0.13, Degrees of Freedom = 1, P-Value = <.001, 95% CI [0.21, 1.07]  S2 vs S6: Mean Difference = 2.22, Standard Error = 0.19, Degrees of Freedom = 1, P-Value = <.001, 95% CI [1.59, 2.84]  S2 vs S9: Mean Difference = 2.77, Standard Error = 0.30, Degrees of Freedom = 1, P-Value = <.001, 95% CI [1.80, 3.73]  S3 vs S6: Mean Difference = 1.57, Standard Error = 0.14, Degrees of Freedom = 1, P-Value = <.001, 95% CI [1.12, 2.03]  S3 vs S9: Mean Difference = 2.13, Standard Error = 0.27, Degrees of Freedom = 1, P-Value = <.001, 95% CI [1.26, 2.99]  S6 vs S9: Mean Difference = 0.55, Standard Error = 0.23, Degrees of Freedom = 1, P-Value = .709, 95% CI [–0.19, 1.30]  MOP:  S1 vs S2: Mean Difference = 0.60, Standard Error = 0.13, Degrees of Freedom = 1, P-Value = <.001, 95% CI [0.17, 1.03]  S1 vs S3: Mean Difference = 1.31, Standard Error = 0.19, Degrees of Freedom = 1, P-Value = <.001, 95% CI [0.71, 1.92]  S1 vs S6: Mean Difference = 2.83, Standard Error = 0.23, Degrees of Freedom = 1, P-Value = <.001, 95% CI [2.09, 3.56]  S1 vs S9: Mean Difference = 4.25, Standard Error = 0.26, Degrees of Freedom = 1, P-Value = <.001, 95% CI [3.40, 5.11]  S2 vs S3: Mean Difference = 0.71, Standard Error = 0.13, Degrees of Freedom = 1, P-Value = <.001, 95% CI [0.28, 1.14]  S2 vs S6: Mean Difference = 2.22, Standard Error = 0.19, Degrees of Freedom = 1, P-Value = <.001, 95% CI [1.62, 2.83]  S2 vs S9: Mean Difference = 3.65, Standard Error = 0.23, Degrees of Freedom = 1, P-Value = <.001, 95% CI [2.90, 4.40]  S3 vs S6: Mean Difference = 1.51, Standard Error = 0.13, Degrees of Freedom = 1, P-Value = <.001, 95% CI [1.08, 1.95]  S3 vs S9: Mean Difference = 2.94, Standard Error = 0.19, Degrees of Freedom = 1, P-Value = <.001, 95% CI [2.31, 3.56]  S6 vs S9: Mean Difference = 1.42, Standard Error = 0.14, Degrees of Freedom = 1, P-Value = <.001, 95% CI [0.97, 1.88]  Control vs MOP (Across Sessions):  S1: Mean Difference = –1.89, Standard Error = 0.68, Degrees of Freedom = 1, P-Value = .261, 95% CI [–4.12, 0.34]  S2: Mean Difference = –1.82, Standard Error = 0.68, Degrees of Freedom = 1, P-Value = .344, 95% CI [–4.05, 0.41]  S3: Mean Difference = –1.75, Standard Error = 0.68, Degrees of Freedom = 1, P-Value = .466, 95% CI [–3.98, 0.48]  S6: Mean Difference = –1.81, Standard Error = 0.69, Degrees of Freedom = 1, P-Value = .365, 95% CI [–4.05, 0.42]  S9: Mean Difference = –0.94, Standard Error = 0.71, Degrees of Freedom = 1, P-Value = >.999, 95% CI [–3.26, 1.38] |  | MOP group had a negative impact on oral health-related quality of life (OHRQoL), particularly psychological aspects, no baseline OHRQoL questionnaire (the data of quality of life are in table 5) |
| 18.Feizbakhsh et al. (2018) |  | MOP: N=20 mean: 1.3 mm in 28 days, (SD have not been mentioned)  Control: N=20, mean: 0.6mm in 28 days, (SD have not been mentioned)  Control:  Maxilla: N = 20, average (mm/d): 0.7480, SD: 0.4052  Mandible: N = 20, average (mm/d): 0.5350, SD: 0.4113  Experimental:  Maxilla: N = 20, average (mm/d): 1.3620, SD: 0.4967  Mandible: N = 20, average (mm/d): 1.2480, SD: 0.4210  We need SD of mean at the end of day28th calculation. (and of course divided data of each jaw) |  |  |
| 19.Golshah et al. (2021) | Change in distal canine position over 5 months | 1 month:  MOP: n=25, mean =1.459, SD= 0,648  Control: n=25, mean = 1,213, SD= 0.794  LL: -0.06, UL=: 0.55 p-value = 0.108  2 months:  MOP: n = 25, mean = 2.737, SD = 1.141  Control: n = 25, mean = 2.555, SD = 1.406  LL = -0.27, UL = 0.64, p-value = 0.416  3 months:  MOP: n = 25, mean = 4.294, SD = 1.664  Control: n = 25, mean = 3.805, SD = 1.568  LL = -0.07, UL = 1.05, p-value = 0.085  4 months:  MOP: n = 25, mean = 5.384, SD = 1.724  Control: n = 25, mean = 4.733, SD = 1.545  LL = -0.10, UL = 1.40, p-value = 0.087  5 months:  MOP: n = 25, mean = 6.042, SD = 1.424  Control: n = 25, mean = 5.469, SD = 1.381  LL = -0.34, UL = 1.49, p-value = 0.200  Change in canine cusp position over 5 months:  1 month:  MOP: n = 25, mean = 1.454, SD = 0.647  Control: n = 25, mean = 1.234, SD = 0.733  LL = -0.09, UL = 0.53, p-value = 0.161  2 months:  MOP: n = 25, mean = 2.709, SD = 1.134  Control: n = 25, mean = 2.540, SD = 1.387  LL = -0.29, UL = 0.62, p-value = 0.452  3 months:  MOP: n = 25, mean = 4.234, SD = 1.611  Control: n = 25, mean = 3.793, SD = 1.539  LL = -0.11, UL = 0.99, p-value = 0.109  4 months:  MOP: n = 25, mean = 5.338, SD = 1.697  Control: n = 25, mean = 4.684, SD = 1.369  LL = -0.10, UL = 1.41, p-value = 0.085  5 months:  MOP: n = 25, mean = 6.102, SD = 1.374  Control: n = 25, mean = 5.398, SD = 1.271  LL = -0.24, UL = 1.64, p-value = 0.131  Change in distal canine position over 5 months:  MD (calculated based on study information):  1m: 0.246, LL: -0.06, UL=: 0.55  2m: 0.18199999999999994, LL = -0.27, UL = 0.64  3m: 0.48899999999999944, LL = -0.07, UL = 1.05  4m: 0.6510000000000007, LL = -0.10, UL = 1.40  5m: 0.5729999999999995, LL = -0.34, UL = 1.49  Change in canine cusp position over 5 months:  MD (calculated based on study information):  1m: MD: 0.21999999999999997, LL: -0.09, UL: 0.53  2m MD: 0.16900000000000004, LL: -0.29, UL: 0.62  3m MD: 0.44099999999999984, LL: -0.11, UL: 0.99  4m MD: 0.6539999999999999, LL: -0.1, UL: 1.41  5m MD: 0.7040000000000006, LL: -0.24, UL: 1.64 | Degree of Canine Tipping: was measured by calculating the angle formed between the longitudinal axis of the canine and a line perpendicular to the occlusal plane. The angle difference between initial and final scans was recorded in degrees. This outcome evaluated the extent of tipping associated with canine movement, providing insight into the mechanical effects of the intervention on tooth angulation | Comparison of Tipping Movement Between the MOP and Control Groups MOP Group (n = 25): Mean tipping movement: 5.34 Standard deviation (SD): 3.11 95% confidence interval (CI): 3.39 to 0.09 P-value: 0.040 Control Group (n = 25): Mean tipping movement: 7.08 Standard deviation (SD): 3.70  Degree of Canine Tipping in Males and Females Across the MOP and Control Groups Females: Control Group (n = 11):  Mean tipping degree: 7.11 Standard deviation (SD): 3.08 MOP Group (n = 11):  Mean tipping degree: 4.55 Standard deviation (SD): 3.24 95% confidence interval (CI): 5.52 to 0.39 P-value: 0.082 Males: Control Group (n = 14):  Mean tipping degree: 7.05 Standard deviation (SD): 4.24 MOP Group (n = 14):  Mean tipping degree: 5.96 Standard deviation (SD): 2.97 95% confidence interval (CI): 3.22 to 1.04 P-value: 0.291 P-values for Comparison Between Sexes: Control group: 0.969 MOP group: 0.266 |
| 20. GOWDA S V et al. (2019) | Canine movement between experimental side and contralateral side of experimental group (mm) | T0-T1(4 weeks)  Incisal  Mop (experimental side): N= 23, Mean= 9.38, SD= 0.84  Control (contralateral side): N= 23, Mean= 8.18, SD= 0.69  Sig< 0.001  Middle  Mop (experimental side): N= 23, Mean= 8.41, SD= 0.64  Control (contralateral side): N= 23, Mean= 7.31, SD= 0.48  Sig< 0.001  Cervical  Mop (experimental side): N= 23, Mean= 7.08, SD= 0.58  Control (contralateral side): N= 23, Mean= 6.15, SD= 0.46  Sig< 0.001  canine movement in contralateral side before and after retraction (mm)  T0-T1(4 weeks)  Incisal  Before: N= 23, Mean= 8.18, SD= 0.69  After: N= 23, Mean= 7.54, SD= 0.59  MD= 0.63  Sig< 0.01  Middle  Before: N= 23, Mean= 7.31, SD= 0.48  After: N= 23, Mean= 6.73, SD= 0.37  MD= 0.58  Sig< 0.01  Cervical  Before: N= 23, Mean= 6.15, SD= 0.46  After: N= 23, Mean= 5.71, SD= 0.45  MD= 0.44  Sig< 0.01  Average of three (incisal, middle, cervical)  Before: N= 23, Mean= 6.66, SD= 0.38  After: N= 23, Mean= 7.21, SD= 0.48  MD= 0.60  Sig< 0.001  canine movement in experimental side before and after retraction (mm)  T0-T1(4 weeks)  Incisal  Before: N= 23, Mean= 9.38, SD= 0.84  After: N= 23, Mean= 7.54, SD= 0.59  MD= 1.84  Sig< 0.001  Middle  Before: N= 23, Mean= 8.39, SD= 0.64  After: N= 23, Mean= 6.73, SD= 0.37  MD= 1.66  Sig< 0.001  Cervical  Before: N= 23, Mean= 7.08, SD= 0.58  After: N= 23, Mean= 5.71, SD= 0.45  MD= 1.37  Sig< 0.001  Average of three (incisal, middle, cervical)  Before: N= 23, Mean= 6.66, SD= 0.38  After: N= 23, Mean= 8.29, SD= 0.61  MD= 1.62  Sig< 0.001  canine movement between right and left side of control group after retraction (mm)  T1(4 weeks)  Incisal  Right: N= 23, Mean= 8.14, SD= 0.71  Left: N= 23, Mean= 8.12, SD= 0.68  Sig> 0.05  Middle  Right: N= 23, Mean= 7.33, SD= 0.47  Left: N= 23, Mean= 7.25, SD= 0.49  Sig> 0.05  Cervical  Right: N= 23, Mean= 6.14, SD= 0.45  Left: N= 23, Mean= 6.18, SD= 0.49  Sig> 0.05  canine movement in control group between before and after retraction  T0-T1(4 weeks)  Incisal  Before: N= 23, Mean= 7.56, SD= 0.60  After: N= 23, Mean= 8.17, SD= 0.71  MD= 0.6  Sig< 0.01  Middle  Before: N= 23, Mean= 6.71, SD= 0.37  After: N= 23, Mean= 7.31, SD= 0.48  MD= 0.59  Sig< 0.001  Cervical  Before: N= 23, Mean= 5.71, SD= 0.45  After: N= 23, Mean= 6.16, SD= 0.45  MD= 0.46  Sig< 0.01  Average of three (incisal, middle, cervical)  Before: N= 23, Mean= 6.6, SD= 0.39  After: N= 23, Mean= 7.21, SD= 0.47  MD= 0.57  Sig< 0.001  canine movement in groups  MOP (Experimental side): N= 23, MD= 1.62  MOP (Contralateral side): N= 23, MD= 0.60  Control: N=23, MD= 0.57  (MOPs increased the rate of canine retraction by 2.7 fold when compared with the control group and contralateral side of the experimental group) | Pain/discomfort: Pain and discomfort levels were assessed using a numerical rating scale ranging from 0 to 10, where '0' indicated no pain and '10' represented the worst possible pain (An scale from 1 to 10 is also indicated, where 1 being the least and 10 being the highest amount of pain). Discomfort levels were evaluated at four time points: T1 (24 hours), T2 (7th day), T3 (14th day), and T4 (28th day) following the initiation of retraction. | Pain/discomfort:.  T1: MOP (Experimental side): N= 23, Mean ± SD= 1.21 ± 0.42 MOP (Contralateral side): N= 23, Mean ± SD= 1.21 ± 0.42 Control: N= 23, Mean ± SD= 1.26 ± 0.44  T2 and T3 and T4: MOP (Experimental side): N= 23, Mean ± SD= 0.00 ± 0.00 MOP (Contralateral side): N= 23, Mean ± SD= 0.00 ± 0.00 Control: N= 23, Mean ± SD= 0.00 ± 0.00 |
| 21.Gulduren et al. (2020) | Rate of Tooth Movement | T0 to T1:  MOP side: N=9, Mean: 0.572 mm, SD: 0.073 mm, P-value (1–2): 0.000*  Control side: N=9, Mean: 0.494 mm, SD: 0.076 mm, P-value (1–3): 0.363  Control group: N=9, Mean: 0.545 mm, SD: 0.046 mm, P-value (2–3): 0.106  T0 to T2:  MOP side: N=9, Mean: 1.126 mm, SD: 0.149 mm, P-value (1–2): 0.001*  Control side: N=9, Mean: 0.970 mm, SD: 0.162 mm, P-value (1–3): 0.621  Control group: N=9, Mean: 1.097 mm, SD: 0.087 mm, P-value (2–3): 0.056  T0 to T3:  MOP side: N=9, Mean: 1.804 mm, SD: 0.308 mm, P-value (1–2): 0.002*  Control side: N=9, Mean: 1.528 mm, SD: 0.310 mm, P-value (1–3): 0.524  Control group: N=9, Mean: 1.732 mm, SD: 0.126 mm, P-value (2–3): 0.085  T0 to T4:  MOP side: N=9, Mean: 2.421 mm, SD: 0.427 mm, P-value (1–2): 0.002*  Control side: N=9, Mean: 2.073 mm, SD: 0.416 mm, P-value (1–3): 0.606  Control group: N=9, Mean: 2.341 mm, SD: 0.154 mm, P-value (2–3): 0.100  Daily Rate of Tooth Movement:  MOP side: N=9, Mean: 0.029 mm/day, SD: 0.005 mm, P-value (1–2): 0.002*  Control side: N=9, Mean: 0.025 mm/day, SD: 0.005 mm, P-value (1–3): 0.719  Control group: N=9, Mean: 0.028 mm/day, SD: 0.002 mm, P-value (2–3): 0.098mm, P-value (1–3): 0.719;  Control group: N= 10, Mean: 0.028 mm/day, SD: 0.002 mm, P-value (2–3): 0.098 | Periodontal health: Periodontal evaluations were conducted by a blinded periodontist at the Near East University, Faculty of Dentistry, for all subjects prior to initial interventions (T0) and at subsequent time points (T1, T2, T3, and T4). Assessments included plaque index, gingival index, pocket depth, gingival bleeding, gingival recession, attachment level, furcation defects, and tooth mobility for the maxillary second premolars, first molars, and second molars on both sides. The same periodontist performed all evaluations to maintain consistency.  1.Plaque Index, Gingival Index, Pocket Depth and BOP for posterior teeth in different time intervals:  2.Plaque Index of Maxillary Posterior Teeth (Descriptive Data)     3.Gingival Index of Maxillary Posterior Teeth (Descriptive Data)    4.Pocket Depth of Maxillary Posterior Teeth (Descriptive Data)      5.Bleeding on Probing of Maxillary Posterior Teeth (Descriptive Data)  6.Pain/discomfort: NOTE: (more than Pain and discomfort the data of eating difficulty and speech problems are available) NOTE: Nonsteroidal anti-inflammatory drugs (NSAIDs) were avoided due to their potential to inhibit orthodontic tooth movement. Pain, discomfort, eating difficulty, and speech problems were assessed using visual analog scales (VAS) for all subjects at T0, T1 (after interventions) and 1, 3, 7, 14, 20 days after these time points. The meanings of the VAS scores were as follows: no pain “0”, severe pain “10”; very comfortable “0”, very uncomfortable “10”; no eating difficulty “0”, very difficult to eat “10”; no speech problems “0”, very hard to speak “10” | Plaque Index of Maxillary Posterior Teeth (Descriptive Data) 2nd Premolar: T0: MOP side: N=10, Mean: 0.296, SD: 0.423 Contralateral side: N=10, Mean: 0.167, SD: 0.333 Control group: N=10, Mean: 0.139, SD: 0.236 p-values: 1–2: 0.505, 1–3: 0.344, 2–3: 0.841 T1: MOP side: N=10, Mean: 0.315, SD: 0.328 Contralateral side: N=10, Mean: 0.667, SD: 0.697 Control group: N=10, Mean: 0.315, SD: 0.452 p-values: 1–2: 0.060, 1–3: 1.000, 2–3: 0.222 T2: MOP side: N=10, Mean: 0.759, SD: 0.965 Contralateral side: N=10, Mean: 0.519, SD: 0.752 Control group: N=10, Mean: 0.454, SD: 0.973 p-values: 1–2: 0.273, 1–3: 0.513, 2–3: 0.876 T3: MOP side: N=10, Mean: 0.778, SD: 0.697 Contralateral side: N=10, Mean: 0.759, SD: 0.760 Control group: N=10, Mean: 0.722, SD: 1.190 p-values: 1–2: 0.842, 1–3: 0.905, 2–3: 0.938 T4: MOP side: N=10, Mean: 0.389, SD: 0.595 Contralateral side: N=10, Mean: 0.352, SD: 0.420 Control group: N=10, Mean: 0.833, SD: 1.171 p-values: 1–2: 0.738, 1–3: 0.325, 2–3: 0.273 1st Molar: T0: MOP side: N=10, Mean: 0.259, SD: 0.392 Contralateral side: N=10, Mean: 0.093, SD: 0.169 Control group: N=10, Mean: 0.157, SD: 0.330 p-values: 1–2: 0.282, 1–3: 0.559, 2–3: 0.607 T1: MOP side: N=10, Mean: 0.648, SD: 0.621 Contralateral side: N=10, Mean: 0.741, SD: 0.890 Control group: N=10, Mean: 0.398, SD: 0.661 p-values: 1–2: 0.540, 1–3: 0.420, 2–3: 0.368 T2: MOP side: N=10, Mean: 0.759, SD: 0.878 Contralateral side: N=10, Mean: 0.463, SD: 0.564 Control group: N=10, Mean: 0.556, SD: 0.944 p-values: 1–2: 0.187, 1–3: 0.642, 2–3: 0.804 T3: MOP side: N=10, Mean: 0.704, SD: 0.605 Contralateral side: N=10, Mean: 0.889, SD: 0.760 Control group: N=10, Mean: 0.843, SD: 1.054 p-values: 1–2: 0.188, 1–3: 0.736, 2–3: 0.916 T4: MOP side: N=10, Mean: 0.500, SD: 0.697 Contralateral side: N=10, Mean: 0.352, SD: 0.452 Control group: N=10, Mean: 0.639, SD: 0.929 p-values: 1–2: 0.409, 1–3: 0.724, 2–3: 0.417 2nd Molar: T0: MOP side: N=10, Mean: 0.516, SD: 0.664 Contralateral side: N=10, Mean: 0.259, SD: 0.345 Control group: N=10, Mean: 0.259, SD: 0.436 p-values: 1–2: 0.232, 1–3: 0.342, 2–3: 1.000 T1: MOP side: N=10, Mean: 0.722, SD: 0.750 Contralateral side: N=10, Mean: 0.537, SD: 0.730 Control group: N=10, Mean: 0.361, SD: 0.406 p-values: 1–2: 0.331, 1–3: 0.222, 2–3: 0.537 T2: MOP side: N=10, Mean: 0.926, SD: 1.146 Contralateral side: N=10, Mean: 0.593, SD: 0.773 Control group: N=10, Mean: 0.648, SD: 0.942 p-values: 1–2: 0.157, 1–3: 0.582, 2–3: 0.893 T3: MOP side: N=10, Mean: 0.833, SD: 0.777 Contralateral side: N=10, Mean: 0.926, SD: 0.717 Control group: N=10, Mean: 0.935, SD: 1.070 p-values: 1–2: 0.629, 1–3: 0.820, 2–3: 0.983 T4: MOP side: N=10, Mean: 0.556, SD: 0.804 Contralateral side: N=10, Mean: 0.926, SD: 0.791 Control group : N=10, Mean: 0.870, SD: 1.043 p-values: 1–2: 0.243, 1–3: 0.484, 2–3: 0.900  Gingival Index of Maxillary Posterior Teeth (Descriptive Data) 2nd Premolar: T0: MOP side: N=10, Mean: 0.056, SD: 0.167 Contralateral side: N=10, Mean: 0.056, SD: 0.167 Control group: N=10, Mean: 0.083, SD: 0.250 p-values: 1–2: 1.000, 1–3: 0.785, 2–3: 0.785 T1: MOP side: N=10, Mean: 0.611, SD: 0.800 Contralateral side: N=10, Mean: 0.296, SD: 0.558 Control group: N=10, Mean: 0.204, SD: 0.295 p-values: 1–2: 0.120, 1–3: 0.182, 2–3: 0.666 T2: MOP side: N=10, Mean: 0.352, SD: 0.598 Contralateral side : N=10, Mean: 0.333, SD: 0.707 Control group: N=10, Mean: 0.120, SD: 0.200 p-values: 1–2: 0.933, 1–3: 0.287, 2–3: 0.407 T3: MOP side: N=10, Mean: 0.519, SD: 0.621 Contralateral side: N=10, Mean: 0.685, SD: 0.704 Control group: N=10, Mean: 0.519, SD: 0.683 p-values: 1–2: 0.067, 1–3: 1.000, 2–3: 0.617 T4: MOP side: N=10, Mean: 0.296, SD: 0.611 Contralateral side: N=10, Mean: 0.278, SD: 0.565 Control group: N=10, Mean: 0.139, SD: 0.220 p-values: 1–2: 0.347, 1–3: 0.484, 2–3: 0.507 1st Molar: T0: MOP side: N=10, Mean: 0.056, SD: 0.167 Contralateral side: N=10, Mean: 0.056, SD: 0.167 Control group: N=10, Mean: 0.139, SD: 0.417 p-values: 1–2: 1.000, 1–3: 0.585, 2–3: 0.585 T1: MOP side: N=10, Mean: 1.019, SD: 0.788 Contralateral side: N=10, Mean: 0.778, SD: 0.804 Control group: N=10, Mean: 0.583, SD: 0.429 p-values: 1–2: 0.273, 1–3: 0.165, 2–3: 0.534 T2: MOP side: N=10, Mean: 0.741, SD: 0.909 Contralateral side: N=10, Mean: 0.648, SD: 0.827 Control group: N=10, Mean: 0.306, SD: 0.497 p-values: 1–2: 0.782, 1–3: 0.231, 2–3: 0.302 T3: MOP side: N=10, Mean: 0.852, SD: 0.888 Contralateral side: N=10, Mean: 1.056, SD: 0.773 Control group: N=10, Mean: 0.815, SD: 0.644 p-values: 1–2: 0.236, 1–3: 0.921, 2–3: 0.483 T4: MOP side : N=10, Mean: 0.685, SD: 0.743 Contralateral side : N=10, Mean: 0.574, SD: 0.678 Control group : N=10, Mean: 0.620, SD: 0.620 p-values: 1–2: 0.438, 1–3: 0.843, 2–3: 0.882 2nd Molar: T0: MOP side: N=10, Mean: 0.000, SD: 0.000 Contralateral side : N=10, Mean: 0.056, SD: 0.167 Control group : N=10, Mean: 0.139, SD: 0.417 p-values: 1–2: 0.347, 1–3: 0.347, 2–3: 0.585 T1: MOP side : N=10, Mean: 0.685, SD: 0.709 Contralateral side : N=10, Mean: 0.482, SD: 0.626 Control group : N=10, Mean: 0.120, SD: 0.225 p-values: 1–2: 0.471, 1–3: 0.047, 2–3: 0.134 T2: MOP side : N=10, Mean: 0.352, SD: 0.704 Contralateral side : N=10, Mean: 0.278, SD: 0.507 Control group : N=10, Mean: 0.139, SD: 0.333 p-values: 1–2: 0.622, 1–3: 0.424, 2–3: 0.502 T3: MOP side : N=10, Mean: 0.833, SD: 0.986 Contralateral side : N=10, Mean: 0.482, SD: 0.664 Control group : N=10, Mean: 0.528, SD: 0.713 p-values: 1–2: 0.034, 1–3: 0.462, 2–3: 0.888 T4: MOP side : N=10, Mean: 0.426, SD: 0.688 Contralateral side : N=10, Mean: 0.315, SD: 0.489 Control group : N=10, Mean: 0.278, SD: 0.507 p-values: 1–2: 0.262, 1–3: 0.610, 2–3: 0.877  Pocket Depth of Maxillary Posterior Teeth (Descriptive Data) 2nd Premolar: T0: MOP side : N=10, Mean: 1.593, SD: 0.487 Contralateral side : N=10, Mean: 1.519, SD: 0.429 Control group : N=10, Mean: 1.546, SD: 0.404 p-values: 1–2: 0.665, 1–3: 0.829, 2–3: 0.889 T1: MOP side : N=10, Mean: 1.815, SD: 0.467 Contralateral side : N=10, Mean: 1.537, SD: 0.361 Control group : N=10, Mean: 1.648, SD: 0.365 p-values: 1–2: 0.166, 1–3: 0.412, 2–3: 0.525 T2: MOP side : N=10, Mean: 1.889, SD: 0.595 Contralateral side : N=10, Mean: 1.611, SD: 0.595 Control group : N=10, Mean: 1.574, SD: 0.488 p-values: 1–2: 0.153, 1–3: 0.238, 2–3: 0.887 T3: MOP side : N=10, Mean: 1.800, SD: 0.415 Contralateral side : N=10, Mean: 1.667, SD: 0.333 Control group : N=10, Mean: 1.482, SD: 0.171 p-values: 1–2: 0.193, 1–3: 0.051, 2–3: 0.157 T4: MOP side : N=10, Mean: 1.722, SD: 0.493 Contralateral side : N=10, Mean: 1.463, SD: 0.247 Control group : N=10, Mean: 1.574, SD: 0.385 p-values: 1–2: 0.100, 1–3: 0.488, 2–3: 0.477 1st Molar: T0: MOP side : N=10, Mean: 1.611, SD: 0.618 Contralateral side : N=10, Mean: 1.407, SD: 0.324 Control group : N=10, Mean: 1.509, SD: 0.478 p-values: 1–2: 0.216, 1–3: 0.701, 2–3: 0.604 T1: MOP side : N=10, Mean: 1.944, SD: 0.400 Contralateral side : N=10, Mean: 1.722, SD: 0.323 Control group : N=10, Mean: 1.639, SD: 0.270 p-values: 1–2: 0.148, 1–3: 0.076, 2–3: 0.561 T2: MOP side : N=10, Mean: 2.074, SD: 0.590 Contralateral side : N=10, Mean: 1.722, SD: 0.300 Control group : N=10, Mean: 1.787, SD: 0.196 p-values: 1–2: 0.106, 1–3: 0.197, 2–3: 0.595 T3: MOP side : N=10, Mean: 2.148, SD: 0.586 Contralateral side : N=10, Mean: 1.889, SD: 0.382 Control group : N=10, Mean: 1.861, SD: 0.470 p-values: 1–2: 0.220, 1–3: 0.268, 2–3: 0.892 T4: MOP side : N=10, Mean: 1.833, SD: 0.612 Contralateral side : N=10, Mean: 1.704, SD: 0.398 Control group : N=10, Mean: 1.982, SD: 0.388 p-values: 1–2: 0.475, 1–3: 0.548, 2–3: 0.153 2nd Molar: T0: MOP side : N=10, Mean: 1.500, SD: 0.441 Contralateral side : N=10, Mean: 1.482, SD: 0.306 Control group : N=10, Mean: 1.426, SD: 0.355 p-values: 1–2: 0.915, 1–3: 0.700, 2–3: 0.726 T1: MOP side : N=10, Mean: 1.630, SD: 0.582 Contralateral side : N=10, Mean: 1.500, SD: 0.417 Control group : N=10, Mean: 1.417, SD: 0.250 p-values: 1–2: 0.439, 1–3: 0.335, 2–3: 0.514 T2: MOP side : N=10, Mean: 1.685, SD: 0.653 Contralateral side : N=10, Mean: 1.389, SD: 0.373 Control group : N=10, Mean: 1.380, SD: 0.382 p-values: 1–2: 0.234, 1–3: 0.248, 2–3: 0.959 T3: MOP side : N=10, Mean: 2.056, SD: 0.731 Contralateral side: N=10, Mean: 1.797, SD: 0.735 Control group: N=10, Mean: 1.389, SD: 0.212 p-values: 1–2: 0.484, 1–3: 0.027, 2–3: 0.130 T4: MOP side: N=10, Mean: 1.537, SD: 0.477 Contralateral side: N=10, Mean: 1.463, SD: 0.582 Control group: N=10, Mean: 1.537, SD: 0.431 p-values: 1–2: 0.758, 1–3: 1.000, 2–3: 0.763  Bleeding on Probing of Maxillary Posterior Teeth (Descriptive Data) 2nd Premolar: T0: MOP side: N=10, Mean: 0.037, SD: 0.111 Contralateral side: N=10, Mean: 0.093, SD: 0.188 Control group: N=10, Mean: 0.000, SD: 0.000 p-values: 1–2: 0.347, 1–3: 0.347, 2–3: 0.179 T1: MOP side: N=10, Mean: 0.111, SD: 0.333 Contralateral side: N=10, Mean: 0.056, SD: 0.118 Control group: N=10, Mean: 0.167, SD: 0.195 p-values: 1–2: 0.500, 1–3: 0.672, 2–3: 0.168 T2: MOP side: N=10, Mean: 0.148, SD: 0.256 Contralateral side: N=10, Mean: 0.130, SD: 0.200 Control group: N=10, Mean: 0.120, SD: 0.167 p-values: 1–2: 0.842, 1–3: 0.789, 2–3: 0.917 T3: MOP side: N=10, Mean: 0.241, SD: 0.335 Contralateral side: N=10, Mean: 0.185, SD: 0.194 Control group: N=10, Mean: 0.130, SD: 0.229 p-values: 1–2: 0.674, 1–3: 0.423, 2–3: 0.586 T4: MOP side: N=10, Mean: 0.000, SD: 0.000 Contralateral side: N=10, Mean: 0.130, SD: 0.286 Control group: N=10, Mean: 0.083, SD: 0.167 p-values: 1–2: 0.211, 1–3: 0.172, 2–3: 0.680 1st Molar: T0: MOP side: N=10, Mean: 0.370, SD: 0.111 Contralateral side: N=10, Mean: 0.056, SD: 0.167 Control group: N=10, Mean: 0.009, SD: 0.028 p-values: 1–2: 0.799, 1–3: 0.477, 2–3: 0.423 T1: MOP side: N=10, Mean: 0.556, SD: 0.391 Contralateral side: N=10, Mean: 0.315, SD: 0.404 Control group: N=10, Mean: 0.398, SD: 0.306 p-values: 1–2: 0.038, 1–3: 0.355, 2–3: 0.628 T2: MOP side: N=10, Mean: 0.482, SD: 0.437 Contralateral side: N=10, Mean: 0.241, SD: 0.237 Control group: N=10, Mean: 0.250, SD: 0.309 p-values: 1–2: 0.044, 1–3: 0.213, 2–3: 0.944 T3: MOP side: N=10, Mean: 0.315, SD: 0.338 Contralateral side: N=10, Mean: 0.463, SD: 0.298 Control group: N=10, Mean: 0.426, SD: 0.376 p-values: 1–2: 0.169, 1–3: 0.519, 2–3: 0.820 T4: MOP side: N=10, Mean: 0.185, SD: 0.282 Contralateral side: N=10, Mean: 0.259, SD: 0.324 Control group: N=10, Mean: 0.426, SD: 0.340 p-values: 1–2: 0.377, 1–3: 0.121, 2–3: 0.303 2nd Molar: T0: MOP side: N=10, Mean: 0.000, SD: 0.000 Contralateral side: N=10, Mean: 0.074, SD: 0.169 Control group: N=10, Mean: 0.019, SD: 0.056 p-values: 1–2: 0.225, 1–3: 0.347, 2–3: 0.363 T1: MOP side: N=10, Mean: 0.148, SD: 0.328 Contralateral side: N=10, Mean: 0.259, SD: 0.374 Control group: N=10, Mean: 0.167, SD: 0.167 p-values: 1–2: 0.576, 1–3: 0.882, 2–3: 0.511 T2: MOP side: N=10, Mean: 0.296, SD: 0.380 Contralateral side: N=10, Mean: 0.130, SD: 0.162 Control group: N=10, Mean: 0.111, SD: 0.182 p-values: 1–2: 0.094, 1–3: 0.206, 2–3: 0.822 T3: MOP side: N=10, Mean: 0.463, SD: 0.447 Contralateral side: N=10, Mean: 0.241, SD: 0.364 Control group: N=10, Mean: 0.204, SD: 0.280 p-values: 1–2: 0.134, 1–3: 0.160, 2–3: 0.812 T4: MOP side : N=10, Mean: 0.130, SD: 0.217 Contralateral side : N=10, Mean: 0.037, SD: 0.073 Control group : N=10, Mean: 0.250, SD: 0.264 p-values: 1–2: 0.095, 1–3: 0.306, 2–3: 0.044; Pain/discomfort: NOTE: (more than Pain and discomfort the data of eating difficulty and speech problems are available) NOTE: Nonsteroidal anti-inflammatory drugs (NSAIDs) were avoided due to their potential to inhibit orthodontic tooth movement. Pain, discomfort, eating difficulty, and speech problems were assessed using visual analog scales (VAS) for all subjects at T0, T1 (after interventions) and 1, 3, 7, 14, 20 days after these time points. The meanings of the VAS scores were as follows: no pain “0”, severe pain “10”; very comfortable “0”, very uncomfortable “10”; no eating difficulty “0”, very difficult to eat “10”; no speech problems “0”, very hard to speak “10”  VAS Scores at T0, T1, and Days After for Pain, Discomfort, and Eating Difficulty Pain: After interventions at T0: MOP side (N=10): Mean: 5.444, SD: 2.963 Contralateral side (N=10): Mean: 2.778, SD: 2.108 Control group (N=10): Mean: 3.833, SD: 3.437 p-values: 1–2: 0.005*, 1–3: 0.303, 2–3: 0.444 1 day after T0: MOP side (N=10): Mean: 3.778, SD: 2.539 Contralateral side (N=10): Mean: 2.000, SD: 1.118 Control group (N=10): Mean: 2.722, SD: 3.124 p-values: 1–2: 0.028, 1–3: 0.443, 2–3: 0.528 3 days after T0: MOP side (N=10): Mean: 2.111, SD: 2.977 Contralateral side (N=10): Mean: 0.889, SD: 1.167 Control group (N=10): Mean: 2.222, SD: 2.694 p-values: 1–2: 0.138, 1–3: 0.935, 2–3: 0.192 7 days after T0: MOP side (N=10): Mean: 1.667, SD: 3.240 Contralateral side (N=10): Mean: 0.333, SD: 0.500 Control group (N=10): Mean: 0.111, SD: 0.333 p-values: 1–2: 0.213, 1–3: 0.189, 2–3: 0.286 14 days after T0: MOP side (N=10): Mean: 0.333, SD: 0.707 Contralateral side (N=10): Mean: 0.111, SD: 0.333 Control group (N=10): Mean: 0.000, SD: 0.000 p-values: 1–2: 0.347, 1–3: 0.195, 2–3: 0.347 20 days after T0: MOP side (N=10): Mean: 0.222, SD: 0.441 Contralateral side (N=10): Mean: 0.111, SD: 0.333 Control group (N=10): Mean: 0.000, SD: 0.000 p-values: 1–2: 0.347, 1–3: 0.169, 2–3: 0.347 ________________________________________ Discomfort: After interventions at T0: MOP side (N=10): Mean: 3.333, SD: 3.123 Contralateral side (N=10): Mean: 3.333, SD: 3.041 Control group (N=10): Mean: 4.556, SD: 2.920 p-values: 1–2: 1.000, 1–3: 0.404, 2–3: 0.397 1 day after T0: MOP side (N=10): Mean: 2.889, SD: 2.667 Contralateral side (N=10): Mean: 2.889, SD: 2.619 Control group (N=10): Mean: 4.111, SD: 2.667 p-values: 1–2: 1.000, 1–3: 0.345, 2–3: 0.341 3 days after T0: MOP side (N=10): Mean: 1.667, SD: 2.291 Contralateral side (N=10): Mean: 1.778, SD: 2.279 Control group (N=10): Mean: 3.333, SD: 2.000 p-values: 1–2: 0.347, 1–3: 0.120, 2–3: 0.143 7 days after T0: MOP side (N=10): Mean: 1.556, SD: 2.351 Contralateral side (N=10): Mean: 1.667, SD: 2.345 Control group (N=10): Mean: 1.889, SD: 1.537 p-values: 1–2: 0.347, 1–3: 0.726, 2–3: 0.815 14 days after T0: MOP side (N=10): Mean: 1.111, SD: 1.764 Contralateral side (N=10): Mean: 1.111, SD: 1.764 Control group (N=10): Mean: 1.222, SD: 1.202 p-values: 1–2: –, 1–3: 0.878, 2–3: 0.878 20 days after T0: MOP side (N=10): Mean: 0.889, SD: 1.364 Contralateral side (N=10): Mean: 0.889, SD: 1.364 Control group (N=10): Mean: 1.111, SD: 1.167 p-values: 1–2: –, 1–3: 0.715, 2–3: 0.715; Other outecome(s) or result(s): Eating difficulty and speech problems |
| 22.Gümüş et al. (2023) | Rate of Tooth Movement | Extraction Space at Baseline (T0)  MOP: N=20, Mean = 6.22 mm, SD = 1.61 mm  Control: N=20, Mean = 6.1 mm, SD = 2.16 mm  p-value: 0.844 (no significant difference at baseline)  Extraction Space After 3 Months (T1)  MOP: N=20, Mean = 3.35 mm, SD = 1.5 mm  Control: N=20, Mean = 3.6 mm, SD = 1.89 mm  p-value: 0.646 (not significant)  Total Space Closure (T0–T1)  MOP: N=20, Mean = 2.88 mm, SD = 1.32 mm  Control: N=20, Mean = 2.51 mm, SD = 1.41 mm  p-value: 0.397 (not significant)  Confidence Intervals  MOP: LL = 1.54 mm, UL = 5.81 mm  Control: LL =0.37 mm, UL = 4.47 mm |  |  |
| 23.Hashem et al. (2024) | Rate of Tooth Movement | Group I (Single MOP Protocol)  Canine Distalization (CD):  Control: N = 9, Mean = 3.644 mm, SD = 1.158 mm  MOP: N = 9, Mean = 3.700 mm, SD = 0.949 mm  Group II (Repeated MOP Protocol)  Canine Distalization (CD):  Control: N = 9, Mean = 3.989 mm, SD = 1.056 mm  MOP: N = 9, Mean = 4.411 mm, SD = 1.505 mm | 1.Anchorage loss  2.Canine Tipping (CT)  3.Canine Rotation (CR)  4.Molar Rotation (MR): | Anchorage loss: Group I (Single MOP Protocol) Anchorage Loss (AL): Control: N = 9, Mean = 0.767°, SD = 0.530° MOP: N = 9, Mean = 0.556°, SD = 0.590°  Group II (Repeated MOP Protocol) Anchorage Loss (AL): Control site: N = 9, Mean = 0.300°, SD = 0.364° MOP: N = 9, Mean = 0.322°, SD = 0.286°;  Group I (Single MOP Protocol) Canine Tipping (CT): Control: N = 9, Mean = 5.989°, SD = 3.783° MOP: N = 9, Mean = 4.089°, SD = 3.558° Canine Rotation (CR): Control: N = 9, Mean = 10.467°, SD = 3.163° MOP: N = 9, Mean = 11.744°, SD = 2.290° Molar Rotation (MR): Control: N = 9, Mean = 1.667°, SD = 0.709° MOP: N = 9, Mean = 1.400°, SD = 0.669°  Group II (Repeated MOP Protocol) Canine Tipping (CT): Control site: N = 9, M = 7.456°, SD = 1.557° MOP: N = 9, Mean = 5.744°, SD = 3.784°  Canine Rotation (CR): Control site: N = 9, Mean= 11.478°, SD = 3.478° MOP: N = 9, Mean = 12.456°, SD = 5.000°  Molar Rotation (MR): Control site: N = 9, Mean = 2.144°, SD = 1.665° MOP: N = 9, Mean = 1.644°, SD = 0.886° |
| 24. Joseph et al. (2022) | Rate of Tooth Movement | T4 (4 months):  Experimental : N=11, Mean± SD = 4.05 mm ± 1.43  Control : N=11, Mean± SD= 2.81 mm ± 1.24 | 1.Periodontal health  2.Root resorption | No significant complications reported.;  Root resorption: T4 (4 months):  Experimental : N=11, Mean± SD = 2.84 ± 1.27 Control : N=11, Mean± SD = 2.47 mm ± 1.72; Other outecome(s) or result(s): LLLT(low level laser therapy): Mean tooth movement = 4.00 ± 0.82 mm. |
| 25. Kilinc et al. (2023) | Rate of Alignment | Rate of Alignment in the MOP Group  Little’s Irregularity Index (Mean ± SD)  MOP (N=14):  Baseline (T0): 7.71 ± 1.40 mm  T1 (4 weeks): 4.61 ± 1.27 mm  T2 (8 weeks): 2.77 ± 1.82 mm  T3 (12 weeks): 1.47 ± 1.07 mm  T4 (16 weeks): 0.74 ± 0.91 mm  Total Improvement (T0 to T4): 6.97 ± 1.73 mm  Control (N=15):  Baseline (T0): 6.93 ± 1.25 mm  T1 (4 weeks): 4.56 ± 1.38 mm  T2 (8 weeks): 2.95 ± 1.13 mm  T3 (12 weeks): 1.97 ± 0.93 mm  T4 (16 weeks): 0.99 ± 0.80 mm  Total Improvement (T0 to T4): 5.95 ± 1.28 mm | 1.Plaque Index (PI)  2.Gingival Index (GI)  3.Probing Pocket Depth (PPD)  4.VAS (Visual Analog Scale) | Plaque Index (PI)  MOP:  N=14  Baseline (T0): 0.81 ± 0.51  After 16 weeks (T4): 1.27 ± 0.42  Total Change (T0 to T4): 0.46 ± 0.57  Control:  N=15  Plaque Index (PI)  Baseline (T0): 0.72 ± 0.48  After 16 weeks (T4): 1.16 ± 0.51  Total Change (T0 to T4): 0.44 ± 0.55  Gingival Index (GI)  Mop:  Baseline (T0): 0.34 ± 0.22  After 16 weeks (T4): 1.06 ± 0.50  Total Change (T0 to T4): 0.72 ± 0.40  Control group:  Baseline (T0): 0.49 ± 0.40  After 16 weeks (T4): 0.91 ± 0.46  Total Change (T0 to T4): 0.42 ± 0.50  Probing Pocket Depth (PPD)  Mop:  Baseline (T0): 1.24 ± 0.15 mm  After 16 weeks (T4): 1.73 ± 0.21 mm  Total Change (T0 to T4): 0.50 ± 0.21 mm  Control:  Baseline (T0): 1.41 ± 0.30 mm  After 16 weeks (T4): 1.45 ± 0.32 mm  Total Change (T0 to T4): 0.03 ± 0.27 mm  VAS (Visual Analog Scale) is a subjective measure of pain intensity.  Which ranges from 0 to 100, where:  (0 = No pain, 100 = Worst imaginable pain)  MOP  N = 14  Day 1: 67.65 ± 22.17  Day 2: 51.97 ± 22.26  Day 3: 37.41 ± 27.49  Day 4: 21.98 ± 25.48  Day 7: 4.61 ± 6.21  Control  N = 15  Day 1: 42.05 ± 8.25  Day 2: 32.78 ± 11.21  Day 3: 22.50 ± 10.15  Day 4: 11.20 ± 9.01  Day 7: 1.10 ± 0.75 |
| 26.Kumar et al. (2024) | Rate of Tooth Movement | Maxillary Arch:  MOP: N=10  T0–T1: Mean = 1.15 mm, SD = 0.16 mm  T1–T2: Mean = 1.11 mm, SD = 0.13 mm  T2–T3: Mean = 1.02 mm, SD = 0.09 mm  T3–T4: Mean = 0.95 mm, SD = 0.16 mm  Overall Rate (T0–T4): M = 1.06 mm/month, SD = 0.34 mm  Control Group: N=10  T0–T1: Mean = 0.73 mm, SD = 0.06 mm  T1–T2: Mean = 0.69 mm, SD = 0.07 mm  T2–T3: Mean = 0.69 mm, SD = 0.09 mm  T3–T4: Mean = 0.65 mm, SD = 0.05 mm  Overall Rate (T0–T4): M = 0.69 mm/month, SD = 0.22 mm  Mandibular Arch  MOP: N=10  T0–T1: Mean = 0.97 mm, SD = 0.12 mm  T1–T2: Mean = 0.98 mm, SD = 0.13 mm  T2–T3: Mean = 0.93 mm, SD = 0.11 mm  T3–T4: Mean = 0.82 mm, SD = 0.24 mm  Overall Rate (T0–T4): M = 0.92 mm/month, SD = 0.39 mm  Control: N=10  T0–T1: Mean = 0.67 mm, SD = 0.04 mm  T1–T2: Mean = 0.66 mm, SD = 0.04 mm  T2–T3: Mean = 0.70 mm, SD = 0.20 mm  T3–T4: Mean = 0.61 mm, SD = 0.05 mm  Overall Rate (T0–T4): M = 0.66 mm/month, SD = 0.26 mm | 1.Periodontal health  2.Pain/discomfort: VAS Scores (0-10) | Healthy periodontal status maintained  Pain/discomfort: VAS Scores (0-10) MOP: N=10 24 hours: Mean = 5.0, SD = 1.24 7 days: Mean = 2.10, SD = 0.99 14 days: Mean = 0.80, SD = 1.13 Control Group: N=10 24 hours: Mean = 1.5, SD = 0.80 7 days: Mean = 0.70, SD = 0.40 14 days: Mean = 0.40, SD = 0.20 |
| 27.Kundi et al. (2018) | Canine Tip Movement | Canine Tip Movement at 28 days:  MOP: N=28, Mean ± SD = 1.52 ± 0.12mm, 95% CI: (0.96, 1.07)  Control: N=28, Mean ± SD = 0.51 ± 0.07mm, 95% CI: (0.31, 0.95)  The independent samples t-test revealed a statistically significant difference in canine tip retraction between the Perforation and Conventional groups (p < 0.001)  Canine Cervical Movement at 28 days:  MOP: N=28, Mean ± SD = 1.33 ± 0.13mm, 95% CI: N/A  Control: N=28, Mean ± SD = 0.42 ± 0.06mm, 95% CI: N/A  The independent samples t-test revealed a statistically significant difference in canine cervical retraction between the Perforation and Conventional groups (p < 0.001) |  |  |
| 28.Li et al. (2022) | Rate of Tooth Movement | Control:  N = 17  Mean (M) = 2.84 mm  Standard Deviation (SD) = 0.84 mm  Standard Error (SE) = 0.20 mm  MOP:  N = 17  Mean (M) = 3.52 mm  Standard Deviation (SD) = 0.98 mm  Standard Error (SE) = 0.24 mm  Mean Difference (MD) Between Groups  MD = 0.69 mm  SD = 0.58 mm  SE = 0.14 mm  95% Confidence Interval (CI) = (-0.39, 0.99)  T1  N = 17, mean =1.28, SD = 0.56  N = 17, mean =1.16, SD = 0.66  T2  N = 17, mean =2.40, SD = 0.84  N = 17, mean =2.18, SD = 1.11  T3  N = 17, mean =3.52, SD = 0.98  N = 17, mean =2.84, SD = 0.84 | 1.Periodontal health  2.Pain/discomfort: VASs score (0-10)  3.Root resorption | Periodontal health: No adverse effects observed  Pain/discomfort: VASs score (0-10) (Numbers are available for days 0,1,3 and 7) Control: N = 18 Mean (M) = 3.06 (VAS score) Standard Deviation (SD) = 5.72 Standard Error (SE) = 1.35 95% Confidence Interval (CI) = (0.21, 5.90) Minimum = 0 Maximum = 20  MOP: N = 18 Mean (M) = 3.89 (VAS score) Standard Deviation (SD) = 6.98 Standard Error (SE) = 1.64 95% Confidence Interval (CI) = (0.42, 7.36) Minimum = 0 Maximum = 20  Pain Score Difference Between Groups (Day 7) Mean Difference (MD) = 0.83 (VAS score) P Value = 0.70 (Not significant); Root resorption: Slight increase in resorption on the MOP side  Slight increase in resorption on the MOP side |
| 29.Mahmoudi et al. (2016) | Rate of Tooth Movement | TAD-U3D (mm) (TAD to maxillary canine distal curvature)  T0-T2 (3months)  Control: N= 10, Mean=2.25, SD=1.43, SE=0.45, CI (LL:1.22, UL:3.28)  MOP: N= 10, Mean=1.81, SD=0.87, SE=0.27, CI (LL:1.18, UL:2.43)  degree of freedom:9, t(9)=0.92 ,P-value=0.19  U5M-U3D (mm) (maxillary second premolar mesial curvature to maxillary canine distal curvature)  T0-T2 (3months)  Control: N= 10, Mean=2.36, SD=1.56, SE=0.49, CI (LL:1.24, UL:3.47)  MOP: N= 10, Mean=2.66, SD=0.82, SE=0.26, CI (LL:2.07, UL:3.25)  degree of freedom:9, t(9)=-0.62 ,P-value=0.72  U3 Cusp tip- U5 cusp tip (mm) (distance between canine and second premolar cusp tips in maxilla)  T0-T2 (3months)  Control: N= 10, Mean=2.83, SD=1.09, SE=0.34, CI (LL:2.04, UL:3.62)  MOP: N= 10, Mean=2.97, SD=1.05, SE=0.33, CI (LL:2.21, UL:3.73), degree of freedom:9, t(9)=-0.4 ,P-value=0.65  U5M-U3D (mm) (extraction space between canine distal wall and second premolar mesial wall in maxilla)  T0-T2 (3months)  Control: N= 10, Mean=2.55, SD=1.21, SE=0.38, CI (LL:1.68, UL:3.42)  MOP: N= 10, Mean=2.31, SD=1.00, SE=0.31, CI (LL:1.59, UL:3.03),  degree of freedom:9, t(9)=0.68 ,P-value=0.25  U2 Midpoint- U3 Cusp tip (mm) (distance from lateral incisor midpoint to canine cusp tip in maxilla)  T0-T2 (3months)  Control: N= 10, Mean=1.96, SD=0.69, SE=0.21, CI (LL:1.46, UL:2.45)  MOP: N= 10, Mean=2.20, SD=1.18, SE=0.37, CI (LL:1.36, UL:3.04)  degree of freedom:9, t(9)=-0.8 ,P-value=0.77  U6 MP Cusp Tip- U3 Cusp Tip (mm) (distance from first molar mesio-palatal cusp tip to canine cusp tip in maxilla)  T0-T2 (3months)  Control: N= 10, Mean=2.84, SD=0.93, SE=0.29, CI (LL:2.16, UL:3.51)  MOP: N= 10, Mean=3.02, SD=0.67, SE=0.21, CI (LL:2.54, UL:3.50)  degree of freedom:9, t(9)=-0.53 ,P-value=0.69 |  |  |
| 30.Martina et al. (2022) | Rate of Tooth Movement | For 3MOP:  T1 (30 days):  Group 3MOPs: N=10, Mean: 1.235, SD: 0.441, SE: 0.098, P-value: 0.001 Control: N=10, Mean: 0.600, SD: 0.265, SE: 0.059  T2 (60 days):  Group 3MOPs: N=10, Mean: 1.050, SD: 0.256, SE: 0.057, P-value: 0.001  Control: N=10, Mean: 0.410, SD: 0.224, SE: 0.050  T3 (90 days):  Group 3MOPs: N=10, Mean: 1.235, SD: 0.210, SE: 0.047, P-value: 0.001  Control: N=10, Mean: 0.620, SD: 0.190, SE: 0.042  For 2MOP:  T1 (30 days):  Group 2MOPs: N= 10, Mean: 1.020, SD: 0.223, SE: 0.050, P-value: 0.438  Control: N= 10, Mean: 0.960, SD: 0.153, SE: 0.034  T2 (60 days):  Group 2MOPs: N= 10, Mean: 0.930, SD: 0.180, SE: 0.040, P-value: 0.583  Control: N= 10, Mean: 0.850, SD: 0.153, SE: 0.034  T3 (90 days):  Group 2MOPs= N: 10, Mean: 0.910, SD: 0.144, SE: 0.032, P-value: 0.832  Control: N= 10, Mean: 0.885, SD: 0.122, SE: 0.027 | Pain/discomfort | Pain/discomfort: Pain perception was recorded using a 10 mm visual analogue scale (VAS) at three different time intervals, i.e. at 24 hrs, 48 hrs and at the 7th day after MOP. Patients were strictly instructed not to take any analgesics during the study period. (24h=1.2/ 48h=1/ 7days= 1) (has not mentioned SD or CI or STE) (has not categorized between 2 and 3 MOPs groups). (NSAIDs) were avoided. |
| 31.Mordente et al. (2024) | Incisor Retraction Rate | MOP:  N= 19  Mean:  Incisal level displacement (mm): 0.40 (14 days), 0.79 (1 month), 1.47 (2 months), 2.09 (3 months), 2.62 (4 months)  Cervical level displacement (mm): 0.28 (14 days), 0.41 (1 month), 0.89 (2 months), 1.36 (3 months), 1.73 (4 months)  SD:  Incisal level displacement: 0.27 (14 days), 0.37 (1 month), 0.63 (2 months), 0.68 (3 months), 0.75 (4 months)  Cervical level displacement: 0.24 (14 days), 0.27 (1 month), 0.40 (2 months), 0.43 (3 months), 0.38 (4 months)  P-value: No significant difference between groups (P > 0.05)  Control:  N= 18  Mean:  Incisal level displacement (mm): 0.50 (14 days), 0.77 (1 month), 1.41 (2 months), 1.88 (3 months), 2.29 (4 months)  Cervical level displacement (mm): 0.30 (14 days), 0.32 (1 month), 0.61 (2 months), 1.10 (3 months), 1.39 (4 months)  SD:  Incisal level displacement: 0.60 (14 days), 0.67 (1 month), 0.84 (2 months), 0.74 (3 months), 1.09 (4 months)  Cervical level displacement: 0.59 (14 days), 0.21 (1 month), 0.27 (2 months), 0.38 (3 months), 0.61 (4 months)  P-value: No significant difference between groups (P > 0.05)  Mean Difference: Not statistically significant | 1.Periodontal health  2. Incisor Inclination  3.Root Length Shortening  4.Anchorage Loss | Periodontal health: No adverse effects were observed during the trial.; Anchorage loss: Anchorage Loss (in mm) MOP: 0.04 (4 months) Control: -0.16 (4 months) P-value: No significant difference;  Incisor Inclination (degrees) MOPG: UR1: 6.86 ± 3.84 UL1: 6.07 ± 3.60 CG: UR1: 5.66 ± 4.70 UL1: 5.29 ± 4.60 P-value: No significant difference  Root Length Shortening (mm) MOPG: UR1: 23.16 → 22.29 (Mean Difference: -0.87, P < 0.05) UL1: 22.76 → 21.98 (Mean Difference: -0.78, P < 0.05) CG: UR1: 23.87 → 23.12 (Mean Difference: -0.77, P < 0.05) UL1: 23.77 → 23.01 (Mean Difference: -0.76, P < 0.05) P-value (Intergroup): No significant difference  Anchorage Loss (in mm)  MOP: 0.04 (4 months)  Control: -0.16 (4 months)  P-value: No significant difference |
| 32.Ozkan et al. (2021) | Rate of Tooth Movement (Canine Distalization) | Rate of Tooth Movement (Canine Distalization)  MOP(4mm Group):  N = 12  Mean = 1.22 mm/month  SD = 0.29 mm  p-value = 0.016 (statistically significant)  95% CI: LL = 1.03 mm, UL = 1.41 mm  MOP(7mm Group):  N = 12  Mean = 1.29 mm/month  SD = 0.31 mm  p-value = 0.016 (statistically significant)  95% CI: LL = 1.09 mm, UL = 1.49 mm  Control:  N = 12  Mean = 0.88 mm/month  SD = 0.19 mm | 1.Pain/discomfort: VAS (Pain Perception) Scores  2.Anchorage loss: Molar Mesialization | Pain/discomfort: VAS (Pain Perception) Scores MOP(4mm Group): N = 12 Mean (M) = 42.75 mm Standard Deviation (SD) = 14.52 mm Minimum = 23.50 mm Maximum = 65.75 mm p-value = 0.749 (not statistically significant)  MOP(7mm Group) N = 12 Mean (M) = 47.25 mm Standard Deviation (SD) = 16.64 mm Minimum = 24.75 mm Maximum = 76.50 mm p-value = 0.749 (not statistically significant)  Anchorage loss: Molar Mesialization MOP(4mm Group): Mean = 0.17 mm/month p-value > 0.05 (not significant)  MOP(7mm Group): Mean = 0.19 mm/month p-value > 0.05 (not significant)  Control: Mean = 0.18 mm/month |
| 33.RAGHAV et al. (2020) | Rate of Tooth Movement | (T0: Baseline, T1 : 4 weeks, T2 : 8 weeks, T3 : 12 weeks, T4 : 16 weeks)  T0 to T1:  MOP : N=28, Mean ± SD = 1.1164 mm ± 0.486 mm, (p = 0.0272)  Control : N=28, Mean ± SD = 0.8224 ± 0.423mm ,p value< 0.05 (Significant)  T1-T2 :  MOP: N=28, Mean ± SD = 0.896 ± 0.5110mm, (p = 0.3754)  Control : N=28, Mean ± SD = 1.0428± 0.6202 ,p > 0.05 and non-significant (NS)  T2- T3:  MOP: N=28, Mean ± SD = 1.1068 ± 0.5878, (p = 0.7864)  Control : N=28, Mean ± SD = 1.1548±0.6314 , p > 0.05 and (NS)  T3-T4:  MOP: N=28, Mean ± SD = 0.9996 ± 0.4128, (p = 0.6420)  Control : N=28, Mean ± SD = 0.9332±0.5590 , p > 0.05 and (NS) |  | There was a statistically significant difference in the rate of canine re traction only after the first 4 weeks. Subsequently there was no statistically significant difference from the eighth to the sixteenth weeks  between MOPs and control group. There was a statistically significant difference in alkaline and acid phosphates activity in GCF between MOPs and control groups during the initial 4 weeks of canine retraction. |
| 34. RASHEED. V et al. (2018) | Distance: (The distance between the vertical lines drawn along the long axis of the canine and lateral incisors was measured at the incisal edge) | MOP  T0 (start of retraction):  N=10, Mean±SD= 7.78±0.45  T1 (45 days):  N=10, Mean±SD= 10.31±0.55  T2 (completion: 114.5±9.5 days)  N=10, Mean±SD= 12.92±0.30  F: 694.767 , P-value: 0.00  Control  T0 (start of retraction):  N=10, Mean±SD= 7.78±0.45  T1 (45 days):  N=10, Mean±SD= 8.89±0.48  T2 (completion: 114.5±9.5 days)  N=10, Mean±SD= 10.31±0.46  F: 1387.294 , P-value: 0.00  Distance:  T0 (start of retraction):  MOP: N=10, Mean±SD= 7.78±0.45  Control: N=10, Mean±SD= 7.78±0.45  MD= 0.00, t= - , P= -  T1 (45 days):  MOP: N=10, Mean±SD= 10.310±0.55  Control: N=10, Mean±SD= 8.890±0.48  MD= 1.4200, t= 6.062, P-value= 0.00  T2 (completion: 114.5±9.5 days)  MOP: N=10, Mean±SD= 12.920±0.30  Control: N=10, Mean±SD= 10.310±0.46  MD= 2.6100, t= 14.837, P-value= 0.00 | Periodontal health: GCF was collected before canine retraction, on the 3rd day, 7th day, and after2 months from the distal side of the canine on both sides Using a 5µl micropipette. | Periodontal health:   Level of IL-1β MOP T0 (beginning): N=10, Mean±SD= 0.17±0.06 T1 (3rd day): N=10, Mean±SD= 0.75±0.09 T2 (7th day): N=10, Mean±SD= 0.51±0.07 T3 (2 months): N=10, Mean±SD= 0.15±0.05 F: 185.876, P-value: 0.00 Control T0 (beginning): N=10, Mean±SD= 0.15±0.05 T1 (3rd day): N=10, Mean±SD= 0.28±0.07 T2 (7th day): N=10, Mean±SD= 0.15±0.05 T3 (2 months): N=10, Mean±SD= 0.14±0.05 F: 11.377, P-value: 0.00  Level of IL-1β: T0 (start of retraction): MOP: N=10, Mean±SD= 0.170±0.06 Control: N=10, Mean±SD= 0.150±0.05 MD= 0.02, t=0.73 , P=0.47  T1 (3rd days): MOP: N=10, Mean±SD= 0.750±0.09 Control: N=10, Mean±SD= 0.280±0.07 MD= 0.47, t= 11.87, P-value= 0.00  T2 (7th days) MOP: N=10, Mean±SD= 0.510±0.07 Control: N=10, Mean±SD= 0.150±0.05 MD= 0.36, t= 12.55, P-value= 0.00  T3 (2 months) MOP: N=10, Mean±SD= 0.150±0.05 Control: N=10, Mean±SD= 0.140±0.05 MD= 0.01, t= 0.42, P-value= 0.67 |
| 35.Rashid et al. (2018) | Amount of enmasse retraction (Distance between the canine and second premolar was calculated) in right and left (experimental group) Right | (T0, T1 (4weeks), T2 (8weeks), T3 (12weeks), T4 (16weeks), T5 (20weeks), T6 (24weeks))  Amount of enmasse retraction (Distance between the canine and second premolar was calculated) in right and left (experimental group)  Right:  D1 (T0-T1): N=10, Mean= 1.7420, SD= 0.04492  D2 (T1-T2): N=10, Mean= 1.6390, SD= 0.03381  D3 (T2-T3): N=10, Mean= 1.3660, SD= 0.01350  D4 (T3-T4): N=10, Mean= 1.3260, SD= 0.01350  D5 (T4-T5): N=10, Mean= 0.9820, SD= 0.41507  D6 (T5-T6): N=10, Mean= 0.2150, SD= 0.31585  F: 550.985  P-value: 0.000  Left  D1 (T0-T1): N=10, Mean= 1.7420, SD= 0.03795  D2 (T1-T2): N=10, Mean= 1.6390, SD= 0.02846  D3 (T2-T3): N=10, Mean= 1.3670, SD= 0.01767  D4 (T3-T4): N=10, Mean= 1.3190, SD= 0.01853  D5 (T4-T5): N=10, Mean= 0.9810, SD= 0.42120  D6 (T5-T6): N=10, Mean= 0.2240, SD= 0.33060  F: 549.388  P-value: 0.000  …………………………………………………………...  Intra group comparison (experimental)  Right  D1-D2: N= 10, MD= 0.10300, SE= 0.09584, sig= 0.889, LL: - 0.1802, UL: 0.3862  D1-D3: N= 10, MD= 0.37600, SE= 0.09584, sig= 0.003, LL: 0.0928, UL: 0.6592  D1-D4: N= 10, MD= 0.41600, SE= 0.09584, sig= 0.001, LL: 0.1328, UL: 0.6992  D1-D5: N= 10, MD= 0.76000, SE= 0.09584, sig= 0.000, LL: 0.4768, UL: 1.0432  D1-D6: N= 10, MD= 1.52700, SE= 0.09584, sig= 0.000, LL: 1.2438, UL: 1.8102  D2-D3: N= 10, MD= 0.27300, SE= 0.09584, sig= 0.065, LL: -0.0102, UL: 0.5562  D2-D4: N= 10, MD= 0.31300, SE= 0.09584, sig= 0.022, LL: 0.0298, UL: 0.5962  D2-D5: N= 10, MD= 0.65700, SE= 0.09584, sig= 0.000, LL: 0.3738, UL: 0.9402  D2-D6: N= 10, MD= 1.42400, SE= 0.09584, sig= 0.000, LL: 1.1408, UL: 1.7072  D3-D4: N= 10, MD= 0.04000, SE= 0.09584, sig= 0.998, LL: -0.2432, UL: 0.3232  D3-D5: N= 10, MD= 0.38400, SE= 0.09584, sig= 0.002, LL: 0.1008, UL: 0.6672  D3-D6: N= 10, MD= 1.15100, SE= 0.09584, sig= 0.000, LL: 0.8678, UL: 1.4342  D4-D5: N= 10, MD= 0.34400, SE= 0.09584, sig= 0.009, LL: 0.0608, UL: 0.6272  D4-D6: N= 10, MD= 1.11100, SE= 0.09584, sig= 0.000, LL: 0.8278, UL: 1.3942  D5-D6: N= 10, MD= 0.76700, SE= 0.09584, sig= 0.000, LL: 0.4838, UL: 1.0502  Left  D1-D2: N= 10, MD= 0.10300, SE= 0.09825, sig= 0.889, LL: - 0.1873, UL: 0.3933  D1-D3: N= 10, MD= 0.37500, SE= 0.09825, sig= 0.004, LL: 0.0847, UL: 0.6653  D1-D4: N= 10, MD= 0.42300, SE= 0.09825, sig= 0.001, LL: 0.1327, UL: 0.7133  D1-D5: N= 10, MD= 0.76100, SE= 0.09825, sig= 0.000, LL: 0.4707, UL: 1.0513  D1-D6: N= 10, MD= 1.51800, SE= 0.09825, sig= 0.000, LL: 1.2277, UL: 1.8083  D2-D3: N= 10, MD= 0.27200, SE= 0.09825, sig= 0.078, LL: -0.0183, UL: 0.5623  D2-D4: N= 10, MD= 0.32000, SE= 0.09825, sig= 0.023, LL: 0.0297, UL: 0.6103  D2-D5: N= 10, MD= 0.65800, SE= 0.09825, sig= 0.000, LL: 0.3677, UL: 0.9483  D2-D6: N= 10, MD= 1.41500, SE= 0.09825, sig= 0.000, LL: 1.1247, UL: 1.7053  D3-D4: N= 10, MD= 0.04800, SE= 0.09825, sig= 0.996, LL: -0.2423, UL: 0.3383  D3-D5: N= 10, MD= 0.38600, SE= 0.09825, sig= 0.003, LL: 0.0957, UL: 0.6763  D3-D6: N= 10, MD= 1.14300, SE= 0.09584, sig= 0.000, LL: 0.8527, UL: 1.4333  D4-D5: N= 10, MD= 0.33800, SE= 0.09584, sig= 0.014, LL: 0.0477, UL: 0.6283  D4-D6: N= 10, MD= 1.09500, SE= 0.09584, sig= 0.000, LL: 0.8047, UL: 1.3853  D5-D6: N= 10, MD= 0.75700, SE= 0.09584, sig= 0.000, LL: 0.4667, UL: 1.0473  ……………………………………………………………  Amount of enmasse retraction in right and left (control group)  Right:  D1 (T0-T1): N=10, Mean= 1.0860, SD= 0.08872  D2 (T1-T2): N=10, Mean= 1.0410, SD= 0.06740  D3 (T2-T3): N=10, Mean= 1.0220, SD= 0.05996  D4 (T3-T4): N=10, Mean= 0.9970, SD= 0.05539  D5 (T4-T5): N=10, Mean= 0.9750, SD= 0.05421  D6 (T5-T6): N=10, Mean= 0.9440, SD= 0.05835  F: 652.014  P-value: 0.000  Left  D1 (T0-T1): N=10, Mean= 1.0720, SD= 0.09520  D2 (T1-T2): N=10, Mean= 1.0260, SD= 0.06703  D3 (T2-T3): N=10, Mean= 1.0070, SD= 0.05774  D4 (T3-T4): N=10, Mean= 0.9830, SD= 0.05755  D5 (T4-T5): N=10, Mean= 0.9550, SD= 0.05442  D6 (T5-T6): N=10, Mean= 0.9170, SD= 0.05397  F: 670.079  P-value: 0.000  ……………………………………………………………  Intra group comparison (control)  Right  D1-D2: N= 10, MD= 0.04500, SE= 0.02911, sig= 0.637, LL: - 0.0410, UL: 0.1310  D1-D3: N= 10, MD= 0.06400, SE= 0.02911, sig= 0.255, LL: -0.0220, UL: 0.1500  D1-D4: N= 10, MD= 0.08900, SE= 0.02911, sig= 0.039, LL: 0.0030, UL: 0.1750  D1-D5: N= 10, MD= 0.11100, SE= 0.02911, sig= 0.005, LL: 0.0250, UL: 0.1970  D1-D6: N= 10, MD= 0.14200, SE= 0.02911, sig= 0.000, LL: 0.0560, UL: 0.2280  D2-D3: N= 10, MD= 0.01900, SE= 0.02911, sig= 0.986, LL: -0.0670, UL: 0.1050  D2-D4: N= 10, MD= 0.04400, SE= 0.02911, sig= 0.658, LL: -0.0420, UL: 0.1300  D2-D5: N= 10, MD= 0.06600, SE= 0.02911, sig= 0.225, LL: -0.0200, UL: 0.1520  D2-D6: N= 10, MD= 0.09700, SE= 0.02911, sig= 0.018, LL: 0.0110, UL: 0.1830  D3-D4: N= 10, MD= 0.02500, SE= 0.02911, sig= 0.955, LL: -0.0610, UL: 0.1110  D3-D5: N= 10, MD= 0.04700, SE= 0.02911, sig= 0.593, LL: -0.0390, UL: 0.1330  D3-D6: N= 10, MD= 0.07800, SE= 0.02911, sig= 0.096, LL: -0.0080, UL: 0.1640  D4-D5: N= 10, MD= 0.02200, SE= 0.02911, sig= 0.974, LL: -0.0640, UL: 0.1080  D4-D6: N= 10, MD= 0.05300, SE= 0.02911, sig= 0.461, LL: -0.0330, UL: 0.1390  D5-D6: N= 10, MD= 0.03100, SE= 0.02911, sig= 0.893, LL: -0.0550, UL: 0.1170  Left  D1-D2: N= 10, MD= 0.04600, SE= 0.02948, sig= 0.628, LL: -0.0411, UL: 0.1331  D1-D3: N= 10, MD= 0.06500, SE= 0.02948, sig= 0.253, LL: -0.0221, UL: 0.1521  D1-D4: N= 10, MD= 0.08900, SE= 0.02948, sig= 0.043, LL: 0.0019, UL: 0.1761  D1-D5: N= 10, MD= 0.11700, SE= 0.02948, sig= 0.003, LL: 0.0299, UL: 0.2041  D1-D6: N= 10, MD= 0.15500, SE= 0.02948, sig= 0.000, LL: 0.0679, UL: 0.2421  D2-D3: N= 10, MD= 0.01900, SE= 0.02948, sig= 0.987, LL: -0.0681, UL: 0.1061  D2-D4: N= 10, MD= 0.04300, SE= 0.02948, sig= 0.691, LL: -0.0441, UL: 0.1301  D2-D5: N= 10, MD= 0.07100, SE= 0.02948, sig= 0.172, LL: -0.0161, UL: 0.1581  D2-D6: N= 10, MD= 0.10900, SE= 0.02948, sig= 0.006, LL: 0.0219, UL: 0.1961  D3-D4: N= 10, MD= 0.02400, SE= 0.02948, sig= 0.964, LL: -0.0631, UL: 0.1111  D3-D5: N= 10, MD= 0.05200, SE= 0.02948, sig= 0.497, LL: -0.0351, UL: 0.1391  D3-D6: N= 10, MD= 0.9000, SE= 0.02948, sig= 0.039, LL: 0.0029, UL: 0.1771  D4-D5: N= 10, MD= 0.02800, SE= 0.02948, sig= 0.931, LL: -0.0591, UL: 0.1151  D4-D6: N= 10, MD= 0.06600, SE= 0.02948, sig= 0.238, LL: -0.0211, UL: 0.1531  D5-D6: N= 10, MD= 0.03800, SE= 0.02948, sig= 0.790, LL: -0.0491, UL: 0.1251  ……………………………………………………………  Inter group comparison  D1 (T0-T1)  Right:  Experimental: N=10, Mean= 1.7420, SD= 0.04492  Control: N=10, Mean= 1.0860, SD= 0.08872  T= 20.861, P-value= 0.024  Left:  Experimental: N=10, Mean= 1.7420, SD= 0.03795  Control: N=10, Mean= 1.0720, SD= 0.09520  T= 20.674, P-value= 0.005  D2 (T1-T2)  Right:  Experimental: N=10, Mean= 1.6390, SD= 0.03381  Control: N=10, Mean= 1.0410, SD= 0.06740  T= 25.077, P-value= 0.029  Left:  Experimental: N=10, Mean= 1.6390, SD= 0.02846  Control: N=10, Mean= 1.0260, SD= 0.06703  T= 26.619, P-value= 0.004  D3 (T2-T3)  Right:  Experimental: N=10, Mean= 1.3660, SD= 0.01350  Control: N=10, Mean= 1.0220, SD= 0.05996  T= 17.699, P-value= 0.000  Left:  Experimental: N=10, Mean= 1.3670, SD= 0.01767  Control: N=10, Mean= 1.0070, SD= 0.05774  T= 18.852, P-value= 0.001  D4 (T3-T4)  Right:  Experimental: N=10, Mean= 1.3260, SD= 0.01350  Control: N=10, Mean= 0.9970, SD= 0.05539  T= 18.250, P-value= 0.001  Left:  Experimental: N=10, Mean= 1.3190, SD= 0.01853  Control: N=10, Mean= 0.9830, SD= 0.05755  T= 17.574, P-value= 0.000  D5 (T4-T5)  Right:  Experimental: N=10, Mean= 0.9820, SD= 0.41507  Control: N=10, Mean= 0.9750, SD= 0.05421  T= 0.053, P-value= 0.003  Left:  Experimental: N=10, Mean= 0.9810, SD= 0.42120  Control: N=10, Mean= 0.9550, SD= 0.05442  T= 0.194, P-value= 0.002  D6 (T5-T6)  Right:  Experimental: N=10, Mean= 0.2150, SD= 0.31585  Control: N=10, Mean= 0.9440, SD= 0.05835  T= -7.177, P-value= 0.014  Left:  Experimental: N=10, Mean= 0.2240, SD= 0.33060  Control: N=10, Mean= 0.9170, SD= 0.05397  T= -6.542, P-value= 0.011  ……………………………………………………………  Mean rate of enmasse retraction(mm)/month  Experimental: N=10, Mean±SD= 1.5175±0.018  Control: N=10, Mean±SD= 1.0292±0.063  P< 0.001 | 1.Pain/discomfort: In the present study, the pain was assessed using visual analogue scale (VAS). In VAS, the questionnaires were asked to the patients after the MOP procedure and scores were given from 0 to 10. Here in the study, all the patients gave the score of 0 and 1, that is, mild pain after the procedure.;  2.Anchorage loss: Assessment of Anchor Loss: Lateral cephalometric radiographs were obtained both before and after the retraction phase. Cephalometric tracings were performed, and the maxilla was superimposed to measure the mesial displacement of the first molar. | Pain/discomfort:  Here in the study, all the patients gave the score of 0 and 1, that is, mild pain after the procedure.  Anchorage loss:  Experimental: Pre: N=10, Mean=20.80, SD=1.549 Post: N=10, Mean=21.60, SD=2.011 T: -0.997, P-value: 0.487  Control: Pre: N=10, Mean=20.6000, SD=1.50555 Post: N=10, Mean=21.6000, SD=2.01108 T: -1.259, P-value: 0.432  Difference between pre and post: Experimental: N=10, Mean=0.8000, SD= 0.78881 Control: N=10, Mean= 1.0000, SD=0.94281 T:-0.514, P-value: 0.400 |
| 36.Sahin et al. (2023) | Rate of Tooth Movement | MOP:  N = 14  Mean (M) = 4.27 mm (T0-T1)  SD = 1.75 mm  Total Tooth Movement (T0-T4) = 9.13 ± 2.53 mm  (T4= fourth month)  P-Value = 0.103 (Not statistically significant over total duration)  Control:  N = 14  Mean (M) = 2.82 mm (T0-T1)  SD = 0.87 mm  Total Tooth Movement (T0-T4) = 7.56 ± 1.46 mm  (T4= fourth month)  Baseline to M1:  N = 14, mean = 4.27, SD = 1.75  N = 14, mean = 2.82, SD = 0.87  Baseline to M2:  N = 14, mean = 6.35, SD = 2.24  N = 14, mean = 5.13, SD = 1.57  Baseline to M3:  N = 14, mean = 8.28, SD = 2.51  N = 14, mean = 6.68, SD = 1.62  Baseline to M4:  N = 14, mean = 9.13, SD = 2.53  N = 14, mean = 7.56, SD = 1.46 | 1.Periodontal health: Gingival Index (GI))  2.Bleeding on Probing (BOP)  3.Pain/discomfort: VAS Scale Used: 0-10 (No Pain to Unbearable Pain)  4.Anchorage loss: (Molar Mesialization) | Periodontal health: Gingival Index (GI) MOP: N = 14 T0 (Baseline): 0.34 ± 0.10 T1 (1st Month): 0.44 ± 0.08 p-value = 0.000 (Statistically significant increase) Control Group (N = 14) T0 (Baseline): 0.41 ± 0.14 T1 (1st Month): 0.54 ± 0.11 p-value = 0.008 (Statistically significant increase)  Bleeding on Probing (BOP) MOP Group: N = 14 T0 (Baseline): 7.78 ± 3.75 T1 (1st Month): 12.76 ± 4.95 p-value = 0.001 (Statistically significant increase) Control Group (N = 14) T0 (Baseline): 10.97 ± 6.57 T1 (1st Month): 18.37 ± 8.30 p-value = 0.039 (Statistically significant increase)  Pain/discomfort: VAS Scale Used: 0-10 (No Pain to Unbearable Pain) MOP: N = 14 T0 (Baseline): 5.96 ± 2.93 T1 (1st Month): 5.32 ± 2.51 T2 (2nd Month): 4.25 ± 2.01 T3 (3rd Month): 1.57 ± 1.55 T4 (4th Month): 0.64 ± 1.15 Final Follow-up (T5, End of Study): 0.14 ± 0.36 p-value (T0-T1): 0.002 (Statistically significant difference in initial pain) Control: N = 14 T0 (Baseline): 2.64 ± 1.98 T1 (1st Month): 4.14 ± 2.14 T2 (2nd Month): 6.25 ± 2.33 T3 (3rd Month): 1.82 ± 1.50 T4 (4th Month): 0.96 ± 1.31 Final Follow-up (T5, End of Study): 0.36 ± 0.60 p-value (T0-T1): 0.193 (Not statistically significant)  Anchorage loss: Anchorage Loss (Molar Mesialization) MOP: N = 14 Intercanine Distance Increase = 1.96 ± 1.39 mm Intermolar Distance Increase = 0.74 ± 1.07 mm p-value (Intercanine Distance) = 0.000 (Statistically significant) p-value (Intermolar Distance) = 0.022 (Statistically significant) Control: N = 14 Intercanine Distance Increase = 1.29 ± 1.06 mm Intermolar Distance Increase = 0.27 ± 0.43 mm p-value (Intercanine Distance) = 0.001 (Statistically significant) p-value (Intermolar Distance) = 0.033 (Statistically significant) |
| 37.Singh et al. (2023) | Distance between the canine and lateral incisor at different time intervals at the incisal, middle, and cervical | MOP1 (3 Buccal MOPs):  Day 1 (T0)  Incisal:  MOP: N=10, Mean= 8.09, SD= 0.551  Control: N=10, Mean=8.182, SD= 1.383  Mean difference= ‑0.091, p value= 0.85  Middle:  MOP: N=10, Mean= 7.599, SD= 0.501  Control: N=10, Mean= 7.693, SD= 0.797  Mean difference= ‑0.094, p= 0.76  Cervical:  MOP: N=10, Mean= 6.970, SD= 0.778  Control: N=10, Mean= 6.676, SD= 0.864  Mean difference= 0.294, p= 0.43  Day 28 (T1):  Incisal:  MOP: N=10, Mean= 9.227, SD= 0.516  Control: N=10, Mean= 8.664, SD= 1.218  Mean difference= 0.563,p= 0.20  Middle:  MOP: N=10, Mean= 8.624, SD= 0.517  Control: N=10, Mean= 7.952, SD= 0.747  Mean difference= 0.672, p= 0.03* signif  Cervical:  MOP: N=10, Mean= 7.740, SD= 0.752  Control: N=10, Mean= 7.008, SD= 0.873  Mean difference=0.732 ,p=0.04*signif  Day 56 (T2)  Incisal:  MOP: N=10, Mean= 9.918, SD= 0.710  Control: N=10, Mean= 9.213,SD= 1.170  Mean difference= 0.705, p= 0.12  Middle:  MOP: N=10, Mean= 9.199, SD= 0.574  Control: N=10, Mean= 8.459, SD= 0.626  Mean difference = 0.740. p=0.01*signif  Cervical:  MOP: N=10, Mean= 8.319,SD= 0.671  Control: N=10, Mean= 7.385, SD= 0.763  Mean difference= 0.934. p= 0.009  Mean rate of canine retraction at different time intervals at the incisal, middle, and cervical  MOP2 (3 Buccal + 3 Palatal MOPs):  Day 1 (T0):  Incisal:  MOP: N=10, Mean= 7.983, SD= 0.341  Control: N=10, Mean= 8.332, SD= 0.742  Mean difference = ‑0.349, p= 0.19  Middle:  MOP: N=10, Mean= 7.187, SD= 0.392  Control: N=10, Mean= 7.644,SD= 0.599  Mean difference = ‑0.457, p= 0.07  Cervical:  MOP: N=10, Mean= 6.300,SD= 0.483  Control: N=10, Mean= 6.527,SD= 0.759  Mean difference = ‑0.227,p= 0.44  Day 28 (T1):  Incisal:  MOP: N=10, Mean= 9.282,SD= 0.415  Control: N=10, Mean= 8.755, SD= 0.748  Mean difference = 0.527,p= 0.07  Middle:  MOP: N=10, Mean= 8.149, SD= 0.433  Control: N=10, Mean= 8.027, SD= 0.752  Mean difference = 0.122. p= 0.66  Cervical:  MOP: N=10, Mean= 7.103, SD= 0.365  Control: N=10, Mean= 6.849, SD= 0.807  Mean difference = 0.254, p= 0.38  Day 56 (T2):  Incisal:  MOP: N=10, Mean= 9.964, SD= 0.402  Control: N=10, Mean= 9.225, SD= 0.728  Mean difference = 0.739, p= 0.01*  Middle:  MOP: N=10, Mean= 8.717, SD= 0.436  Control: N=10, Mean= 8.319,SD= 0.836  Mean diff= 0.398. p= 0.20  Cervical:  MOP: N=10, Mean= 7.647,SD= 0.439  Control:Mean= 7.171.SD= 0.977  Mean difference = 0.476. p= 0.18  Comparison of Mean rate of overall( average of incisal, middle, and cervical parts) canine retraction(distance between the canine and lateral incisor) of MOP and Control side at different time intervals using independent Student’s t‑test for MOP1 and MOP2:  MOP1 (3 Buccal MOPs): N=10  Day 1 (T0):  MOP: Mean= 7.550, SD=0.515  Control: Mean= 7.515, SD= 0.859  MD= 0.035. p=0.91  Day 28 (T1):  MOP: Mean= 8.528, SD= 0.484  Control: Mean= 7.861,SD= 0.844  MD= 0.667,p=0.04*signific  Day 56 (T2):  MOP: Mean= 9.141,SD= 0.515  Control: Mean= 8.349, SD= 0.752  MD= 0.792. p=0.01*  MOP2 (3 Buccal + 3 Palatal MOPs): N=10  Day 1 (T0)  MOP: Mean= 7.146, SD= 0.284  Control:Mean= 7.498, SD= 0.618  MD= ‑0.352 .p =0.12  Day 28 (T1):  MOP: Mean= 8.180, SD= 0.287  Control:Mean= 7.867, SD= 0.684  MD= 0.313 ,p=0.20  Day 56 (T2):  MOP: Mean= 8.773, SD= 0.284  Control: Mean= 8.241,SD= 0.767  MD= 0.532. p=0.04*signif  Table 5: Comparison of difference in overall Mean canine retraction between MOP and Control side…  MOP1 (3 Buccal MOPs):  Time Interval T1-T0 (0-28 Days):  MOP: N=10, Mean = 0.978 mm, SD = 0.087 mm  Control: N=10, Mean = 0.346 mm, SD = 0.214 mm  Mean Difference (MOP - Control): MD =0.632 mm  p-value: <0.001 (Statistically Significant)  Time Interval T2-T1 (28-56 Days):  MOP: N=10, Mean = 0.613 mm, SD = 0.202 mm  Control: N=10, Mean = 0.488 mm, SD = 0.223 mm  Mean Difference (MOP - Control): MD = 0.125 mm  p-value: 0.33 (Not Statistically Significant)  Time Interval T2-T0 (0-56 Days):  MOP: Mean = N=10, 1.591 mm, SD = 0.223 mm  Control Side: N=10, Mean = 0.834 mm, SD = 0.378 mm  Mean Difference (MOP - Control): MD= 0.757 mm  p-value: <0.001 (Statistically Significant)  MOP2 (3 Buccal + 3 Palatal MOPs):  Time Interval T1-T0 (0-28 Days):  MOP : N=10, Mean = 1.034 mm, SD = 0.125 mm  Control: N=10, Mean = 0.369 mm, SD = 0.176 mm  Mean Difference (MOP - Control): MD = 0.665 mm  p-value: <0.001 (Statistically Significant)  Time Interval T2-T1 (28-56 Days):  MOP: N=10, Mean = 0.593 mm, SD = 0.100 mm  Control: N=10, Mean = 0.374 mm, SD = 0.155 mm  Mean Difference (MOP - Control): MD = 0.219 mm  p-value: 0.002 (Statistically Significant)  Time Interval T2-T0 (0-56 Days):  MOP : N=10, Mean = 1.627 mm, SD = 0.132 mm  Control: N=10, Mean = 0.743 mm, SD = 0.193 mm  Mean Difference (MOP - Control): MD= 0.884 mm  p-value: <0.001 (Statistically Significant) | Pain/discomfort | Pain/discomfort: Mild to moderate pain reported only in first 72 hours. VAS scores not significantly different between Experimental and Control, but statistically significant increase in pain from baseline to 1hr, 24hr, 72hr within each group. “Patient reported local discomfort at the site of the MOP procedure that was bearable, and no medication was needed.” |
| 38.Thomas et al. (2021) | Intergroup comparison of mean amount of canine movement between MOP and control sides at different time intervals | MOP: N = 30, Mean ± SD= 0.71 mm ± 0.2 mm  Control: N = 30, Mean ± SD= 0.41 mm ± 0.3 mm  Mean difference: 0.23 mm  p-value < 0.001  Time interval (15–30 days):  MOP: N = 30, Mean ± SD= 0.61 mm ± 0.3 mm  Control: N = 30, Mean ± SD= 0.45 mm ± 0.2 mm  Mean difference: 0.16 mm  p-value = 0.02  Time interval (1–30 days):  MOP: N = 30, Mean ± SD= 1.32 mm ± 0.4 mm  Control: N = 30, Mean ± SD= 0.86 mm ± 0.4 mm  Mean difference: 0.45 mm  p-value < 0.001  Time interval (30–45 days):  MOP: N = 30, Mean ± SD= 0.73 mm ± 0.2 mm  Control: N = 30, Mean ± SD= 0.49 mm ± 0.2 mm  Mean difference: 0.23 mm  p-value < 0.001  Time interval (1–45 days):  MOP: N = 30, Mean ± SD= 2.05 mm ± 0.4 mm  Control: N = 30, Mean ± SD= 1.36 mm ± 0.5 mm  Mean difference: 0.69 mm  p-value < 0.001  Time interval (45–60 days):  MOP: N = 30, Mean ± SD= 0.56 mm ± 0.2 mm  Control: N = 30, Mean ± SD= 0.49 mm ± 0.2 mm  Mean difference: 0.07 mm  p-value = 0.22  Time interval (1–60 days):  MOP: N = 30, Mean ± SD= 2.61 mm ± 0.6 mm  Control: N = 30, Mean ± SD= 1.85 mm ± 0.5 mm  Mean difference: 0.76 mm  p-value < 0.001  Time interval (60–75 days):  MOP: N = 30, Mean ± SD= 0.52 mm ± 0.3 mm  Control: N = 30, Mean ± SD= 0.48 mm ± 0.1 mm  Mean difference: 0.03 mm  p-value = 0.55  Time interval (1–75 days):  MOP: N = 30, Mean ± SD= 3.12 mm ± 0.7 mm  Control: N = 30, Mean ± SD= 2.33 mm ± 0.5 mm  Mean difference: 0.79 mm  p-value < 0.001  Time interval (75–90 days):  MOP: N = 30, Mean ± SD= 0.48 mm ± 0.2 mm  Control: N = 30, Mean ± SD= 0.49 mm ± 0.2 mm  Mean difference: -0.01 mm  p-value = 0.94  Time interval (1–90 days):  MOP: N = 30, Mean ± SD= 3.61 mm ± 0.8 mm  Control: N = 30, Mean ± SD= 2.82 mm ± 0.6 mm  Mean difference: 0.79 mm  p-value < 0.001  Inter-group comparison of molar tube hook to micro-implant distance between MOP and control sides at different time points (± are SD)  1st day  MOP: N = 30, Mean ± SD= 5.78 mm ± 1.1 mm  Control: N = 30, Mean ± SD= 5.73 mm ± 1.1 mm  Mean difference: 0.05 mm  t-value = 0.17, p-value = 0.86    15th day:  MOP: N = 30, Mean ± SD= 5.75 mm ± 1.1 mm  Control: N = 30, Mean ± SD= 5.68 mm ± 1.1 mm  Mean difference: 0.07 mm  t-value = 0.23, p-value = 0.82  30th day:  MOP: N = 30, Mean ± SD= 5.75 mm ± 1.1 mm  Control: N = 30, Mean ± SD= 5.68 mm ± 1.1 mm  Mean difference: 0.07 mm  t-value = 0.23, p-value = 0.81    45th day:  MOP: N = 30, Mean ± SD= 5.78 mm ± 1.1 mm  Control: N = 30, Mean ± SD= 5.74 mm ± 1.0 mm  Mean difference: 0.04 mm  t-value = 0.14, p-value = 0.89    60th day:  MOP: N = 30, Mean ± SD= 5.73 mm ± 1.1 mm  Control: N = 30, Mean ± SD= 5.68 mm ± 1.0 mm  Mean difference: 0.05 mm  t-value = 0.17, p-value = 0.87  75th day:  MOP: N = 30, Mean ± SD= 5.72 mm ± 1.2 mm  Control: N = 30, Mean ± SD= 5.66 mm ± 1.1 mm  Mean difference: 0.06 mm  t-value = 0.19, p-value = 0.85  90th day:  MOP: N = 30, Mean ± SD= 5.73 mm ± 1.2 mm  Control: N = 30, Mean ± SD= 5.67 mm ± 1.1 mm  Mean difference: 0.06 mm  t-value = 0.21, p-value = 0.84 | 1.Periodontal health: Periodontal status, including PD (probing depth), RAL (relative attachment level), and ABL (alveolar bone level), was assessed on the 1st and 90th days of retraction.  2.Root resorption | Periodontal health: Periodontal status, including PD (probing depth), RAL (relative attachment level), and ABL (alveolar bone level), was assessed on the 1st and 90th days of retraction. A significant increase in PD was observed on both experimental and control sides, while RAL showed no significant changes. ABL changes were insignificant overall, except for the distal surface. Probing Depth (mm) (Measured on 1st and 90th Days):  Experiment: N = 30, Mean (1st Day) = 1.31, SD = 0.4; Mean (90th Day) = 1.72, SD = 0.4; Mean Difference = -0.42; t-value = -5.73; P < 0.001 (significant).  Control: N = 30, Mean (1st Day) = 1.26, SD = 0.3; Mean (90th Day) = 1.47, SD = 0.3; Mean Difference = -0.2; t-value = -2.11; P = 0.04 (significant).  Relative Attachment Level (mm) (Measured on 1st and 90th Days): Experiment: N = 30, Mean (1st Day) = 8.30, SD = 1.1; Mean (90th Day) = 8.34, SD = 1.1; Mean Difference = -0.04; t-value = -0.14; P = 0.88 (not significant). Control: N = 30, Mean (1st Day) = 8.25, SD = 1.1; Mean (90th Day) = 8.28, SD = 1.1; Mean Difference = -0.03; t-value = -0.11; P = 0.91 (not significant).  Intergroup Comparison of Mean Change in Alveolar Bone Level (ABL) Following 90 Days of Canine Retraction (mm)(Measured on 1st and 90th Days): Buccal (R1-L1): Experiment: N = 30, Mean change = 0.09 mm, SD = 0.3, Median = 0.1, Q1 = 0, Q3 = 0.2. Control: N = 30, Mean change = 0.12 mm, SD = 0.4, Median = 0.2, Q1 = -0.08, Q3 = 0.4. P-value = 0.39. Lingual (R1-L2): Experiment: N = 30, Mean change = 0.12 mm, SD = 0.4, Median = 0.1, Q1 = -0.1, Q3 = 0.2. Control: N = 30, Mean change = 0.14 mm, SD = 0.4, Median = 0.1, Q1 = -0.2, Q3 = 0.38. P-value = 0.98. Mesial (R2-L3): Experiment: N = 30, Mean change = 0.01 mm, SD = 0.4, Median = 0.05, Q1 = -1, Q3 = 0.3. Control: N = 30, Mean change = 0.06 mm, SD = 0.5, Median = 0.15, Q1 = -0.18, Q3 = 0.3. P-value = 0.46. Distal (R2-L4): Experiment: N = 30, Mean change = -0.02 mm, SD = 0.3, Median = 0, Q1 = -0.1, Q3 = 0.1. Control: N = 30, Mean change = 0.17 mm, SD = 0.4, Median = 0.2, Q1 = -0.1, Q3 = 0.4. P-value = 0.03*  Root resorption: Root Length (mm) (Measured on 1st and 90th Days):  Experiment: N = 30, Mean (1st Day) = 17.16, SD = 1.0; Mean (90th Day) = 16.92, SD = 1.0; Mean Difference = 0.24; t-value = 5.21; P < 0.001 (significant).  Control: N = 30, Mean (1st Day) = 17.23, SD = 0.9; Mean (90th Day) = 16.93, SD = 0.9; Mean Difference = 0.30; t-value = 6.86; P < 0.001 (significant). |
| 39.Venkatachalapathy et al. (2022) | Rate of Tooth Movement | Maxilla:  T0 = Baseline  T1 (28th day):  MOP: N=20, Mean ± SD = 0.65 ± 0.2mm  Control: N=20, Mean ± SD = 0.37 ± 0.09mm  T2 (56th day):  MOP: N=20, Mean ± SD = 0.74 ± 0.23mm  Control: N=20, Mean ± SD = 0.43 ± 0.11mm  T3 (84th day):  MOP: N=20, Mean ± SD = 0.87 ± 0.27mm  Control: N=20, Mean ± SD = 0.47 ± 0.11mm  p-value = 0.000  Mandible:  T0 = Baseline  T1 (28th day):  MOP: N=20, Mean ± SD = 0.57 ± 0.12mm  Control: N=20, Mean ± SD = 0.34 ± 0.08mm  T2 (56th day):  MOP: N=20, Mean ± SD = 0.68 ± 0.21mm  Control: N=20, Mean ± SD = 0.40 ± 0.15mm  T3 (84th day)  MOP: N=20, Mean ± SD = 0.67 ± 0.10mm  Control: N=20, Mean ± SD = 0.40 ± 0.13mm  p-value = 0.000  The mean differences (MD) between MOP and control sides were statistically significant (p < 0.000) at all time intervals in both maxillary and mandibular dentition. |  |  |

# Appendix Table 6. Risk of Bias Assessment (Quality Assessment)

| Study |  | Selection bias | Performance bias | Detection bias | Attrition bias | Reporting bias | Other bias |
| --- | --- | --- | --- | --- | --- | --- | --- |
|  |  | Allocation concealment | Blinding of participants and personnel | Blinding of outcome assessment | Incomplete outcome data | Selective reporting | Anything else, prespecified |
| 1.Clinical Evaluation of Single Versus Repeated Micro-Osteoperforations During Orthodontic Canine Retraction: A Randomized Clinical Trial, Hashem et al. , 2024 | Assessment | unclear | high | low | low | low | low |
|  | justification | Although randomization was done via coin toss, the authors did not specify clearly if there were procedures ensuring that neither participants nor researchers could foresee the treatment assignment before the patient was enrolled, leaving uncertainty about the allocation concealment adequacy. | The study explicitly stated that blinding of participants and operators was not possible due to the nature of the interventions (single vs. repeated micro-osteoperforations), thus indicating high potential for performance bias. | Blinding was maintained at the stage of outcome assessment. The investigators used coded 3D digital models to conceal group allocation from the evaluator, thus adequately preventing detection bias. | All randomized participants (18 patients) completed the study without dropouts or missing data, indicating that incomplete outcome data were not a concern in this study. | All prespecified primary and secondary outcomes (amount of canine distal movement, canine rotation, tipping, anchorage loss, and molar rotation) described in the methods were reported in the results clearly and completely, with no evidence of selective reporting. | The study appears methodologically sound overall, with clearly described procedures and controls for confounding factors such as age, initial extraction timing, force application standardization, and anchorage management. There is no evidence of other obvious sources of bias that might compromise the validity of the study. |
| 2. Comparison of Rate of Tooth Movement, Root Resorption and Pulp Vitality during En masse Anterior Retraction with Micro-osteoperforation and Low Level Laser Therapy: A Randomised Clinical Trial, joseph et al. , 2022 | Assessment | low | high | low | Low | low | low |
|  | justification | Allocation concealment was adequately described; sealed envelopes were used to prevent prediction of assignments by participants and researchers before enrollment, indicating appropriate concealment. | While the study described itself as "double-blinded," due to the nature of interventions (MOP and LLLT) it would be impossible for patients and operators to remain truly blinded regarding which intervention side was treated, leading to a high risk of performance bias. | The outcome evaluators were explicitly blinded to experimental and control sides during outcome measurement, indicating a proper strategy to minimize detection bias. | All included participants (n=11) completed the study, and there was no reported missing data or dropouts, indicating that incomplete outcome data was not an issue. | All prespecified primary outcomes (rate of tooth movement, root resorption, pulp vitality) described in the methods were clearly reported in the results, without evidence of selective reporting. | The authors considered multiple potential confounding factors (e.g., patient age, biological variation, force standardization, and methodological clarity in interventions and assessments). There were no additional identified methodological issues or sources of bias. |
| 3.Comparison of the effectiveness of piezocision and microosteoperforation in leveling mandibular anterior teeth, kilinc et al. 2023 | Assessment | unclear | high | low | low | low | low |
|  | justification | There is no clear information about how the allocation sequence was concealed from patients and researchers. Since the concealment method is not mentioned explicitly, the risk remains unclear. | Given the surgical nature of the interventions (piezocision and micro-osteoperforation), it is highly likely that both participants and personnel were aware of the allocated intervention, increasing the risk of performance bias significantly. | The authors explicitly stated that outcome measurements (Little’s irregularity index) were performed by a researcher who was blinded to the interventions, which reduces the risk of detection bias. | Only one participant dropped out, clearly reported, and the reasons were appropriately disclosed. Thus, attrition bias is unlikely to have significantly affected the study outcomes. | All outcomes stated in methods (tooth movement, periodontal health, patient comfort, pain assessment) were reported in results consistently, indicating no evidence of selective outcome reporting. | No additional methodological concerns or biases were identified; the procedures, outcome measures, and statistical analysis appeared robust and well-described. |
| 4. Effect of micro osteo-perforations (MOP) on the rate of tooth movement and levels of interleukin-1β – a split mouth study, VN Bavikati et al. 2022 | Assessment | unclear | high | low | low | low | low |
|  | justification | No information was provided regarding allocation concealment procedures. Without explicit details on concealment methods, it is unclear whether assignments could have been foreseen or influenced by investigators. | Given the nature of the intervention (surgical MOP vs. non-surgical control), blinding of patients and clinical personnel would have been impossible. Therefore, the risk of performance bias is inherently high. | The study explicitly mentioned that outcome measurements were taken by a blinded assessor, thereby adequately minimizing detection bias. | There was no report of participant attrition or missing data, and all outcomes seem accounted for throughout the study duration. Thus, the risk of bias due to incomplete outcome data appears low. | All pre-specified primary and secondary outcomes (rate of tooth movement, IL-1β levels in GCF) were reported consistently in the results, suggesting no selective reporting bias. | No other apparent methodological concerns were identified; the split-mouth design appropriately controls for individual variability, and the study protocols were clearly described and executed without evident additional bias. |
| 5. Effect of micro-osteoperforations (MOPs) on the rate of en masse orthodontic tooth retraction: A randomized controlled trial, kumar et al., 2024 | Assessment | low | high | low | low | low | low |
|  | justification | Allocation concealment was adequately described using sequentially numbered, opaque, sealed envelopes that were opened only after baseline assessments were complete, ensuring concealment of allocation. | Due to the invasive nature of the intervention (micro-osteoperforations), blinding of participants and personnel was not feasible, thereby leading to a high risk of performance bias. | Blinding of outcome assessors was clearly stated; outcomes were measured by blinded investigators on digital models, minimizing detection bias effectively. | All randomized patients completed the study with no reported dropouts or exclusions, indicating no concerns related to attrition bias or incomplete outcome data. | All prespecified outcomes (rate of orthodontic tooth movement, patient pain experience) were systematically reported in detail, with no indication of selective outcome reporting. | No additional methodological issues were identified. The study explicitly controlled for relevant confounders (such as age, malocclusion type, and intervention consistency) and included clear protocols for interventions and measurements. |
| 6. Effect of micro-osteoperforations on the moving mandibular molar into long term edentulous area (chinese), tengfei et al,, 2019 | Assessment | unclear | high | low | low | low | low |
|  | justification | The allocation concealment method was not explicitly described, leaving uncertainty regarding the concealment process and thus unclear risk of bias. | Given the nature of the intervention (micro-osteoperforations), participants and clinical personnel were inevitably aware of the treatment, which indicates a high risk of performance bias. | Outcome assessment was done by an evaluator blinded to group allocation, clearly minimizing the risk of detection bias. | All participants completed the study without attrition, and data from all enrolled patients were included in the final analysis, indicating no issues with incomplete outcome data. | All prespecified outcomes (rate of molar movement, root resorption, alveolar bone levels, tooth angulation) described were fully reported, suggesting no selective reporting bias. | No other methodological issues or evident additional sources of bias were identified. The study design was robust and clearly described, controlling appropriately for potential confounding factors. |
| 7.Effect of Micro-osteoperforations on the Rate of Orthodontic Tooth Movement: A Randomized Controlled Trial, Mehta at al., 2020 | Assessment | unclear | high | low | low | low | low |
|  | justification | Although randomization was properly conducted, explicit details on allocation concealment (such as the use of opaque sealed envelopes or independent allocation management) were not provided, leaving this domain unclear. | Participants could not remain blinded after intervention (MOPs), and personnel performing the procedure inevitably knew the group assignments, inherently causing a high risk of performance bias. | Blinded assessors performed the measurements on study models and cephalograms to evaluate outcomes, clearly minimizing detection bias. | Three subjects were excluded due to developing unilateral chewing habits, and this exclusion was transparently reported and justified, suggesting no relevant bias due to incomplete data. | All prespecified outcomes—rate of tooth movement, pain, discomfort, and anchorage loss—were fully reported without selective omission, supporting a low risk of selective outcome reporting. | No additional methodological issues or biases were evident. The study methods, intervention protocol, and data reporting appeared comprehensive and robustly designed, eliminating other obvious sources of bias. |
| 8. Effect of mini-implant facilitated micro-osteoperforations on the alignment of mandibular anterior crowding: A randomised controlled clinical trial, al attar et al., 2022 | Assessment | low | high | low | low | low | low |
|  | justification | Allocation concealment was adequately maintained through the use of identical opaque sealed envelopes, ensuring that neither participants nor clinicians could foresee the assignment, representing a Low. | Participants and clinicians could not be blinded to the interventions due to the invasive nature of the MI-MOPs procedure, inherently resulting in a high risk of performance bias. | Outcome assessors were clearly blinded, as all study models and radiographs were coded and analyzed without knowledge of the treatment assignment, effectively minimizing detection bias. | Dropouts were explicitly reported (two participants withdrew), and clear reasons were provided, with minimal risk of attrition bias affecting the results. | All prespecified outcomes (rate of alignment, pain perception, root resorption) were fully reported, with clear and transparent presentation of results, indicating a low risk of selective reporting bias. | The study was methodologically sound, and no additional sources of significant bias were evident. Appropriate methodological precautions were taken, including controlling the orthodontic treatment protocol across groups and clear reporting of potential limitations. |
| 9. Effects of miniscrew-facilitated micro-osteoperforations on the rate of orthodontic tooth movement, gumus et al., 2023 | Assessment | high | high | unclear | low | low | low |
|  | justification | Although randomization was performed adequately, the study clearly stated that randomization was not blinded; thus, the assignments were openly known by the investigator and patient at the time of allocation, indicating a high risk for selection bias related to concealment | Given the invasive nature of the MOP procedure, blinding of participants and personnel was explicitly reported as not feasible, thus indicating a high risk of performance bias. | There was no clear indication that the assessors measuring the outcomes (digital study models analysis) were blinded to treatment assignment. Without explicit reporting, the risk of detection bias remains unclear. | All enrolled patients completed the study, and no attrition or missing data were reported. This suggests a Low due to incomplete outcome data. | The prespecified primary outcome (rate of orthodontic tooth movement) was reported fully and consistently as planned. There was no indication of selective reporting bias. | No additional sources of bias or methodological concerns were clearly identified; the split-mouth design and controlled procedures provided robustness against other biases. |
| 10. The effect of different micro-osteoperforation depths on the rate of orthodontic tooth movement: A single-center, single-blind, randomized clinical trial, Ozkan et al. 2021 | Assessment | low | high | low | low | low | low |
|  | justification | Allocation concealment was achieved by using opaque, sealed envelopes to allocate interventions randomly, ensuring the concealment of group assignment until the moment of allocation. | Blinding participants and clinical operators was not possible due to the surgical nature of the intervention (MOPs at different depths). Participants were likely aware of the type of intervention received, introducing a high risk of performance bias. | Outcome assessments were clearly blinded; measurements and analyses were performed by an investigator unaware of group assignments, effectively minimizing detection bias. | All participants completed the study, with no dropouts or missing data, indicating a Low related to incomplete outcome data. | The prespecified outcomes (rate of tooth movement, molar mesialization, pain perception) were fully reported, clearly aligning with the methods, which indicates no evidence of selective reporting. | No other apparent methodological concerns or biases were identified. The experimental design, execution, and analysis appeared rigorous, controlling adequately for known confounders. |
| 11. The effect of micro-osteoperforation on the rate of tooth movement during the alignment stage in patients with mandibular crowding: a randomised controlled trial, sahin et al,. 2023 | Assessment | low | high | low | low | low | low |
|  | justification | Allocation concealment was explicitly reported as being maintained through the use of numbered, sealed, and opaque envelopes, which is considered adequate and reduces the risk of bias significantly. | Blinding of participants and personnel was clearly reported as not feasible due to the surgical nature of the intervention (MOP), inherently increasing the risk of performance bias. | Outcome assessors were blinded, as explicitly stated, ensuring that the evaluation of outcomes such as tooth alignment and periodontal measurements were not influenced by knowledge of group assignment. | There were no reported dropouts or missing data; all participants completed the study, thus indicating low risk of attrition bias. | All prespecified primary and secondary outcomes (rate of tooth movement, periodontal health, patient satisfaction, pain) were comprehensively reported according to the methods described, showing no evidence of selective reporting. | No additional methodological concerns or biases were identified. The study design, execution, and reporting appeared robust, with clearly described methods and transparent data handling. |
| 12. The effect of micro-osteoperforations on orthodontic space closure investigated over 12 weeks: a split-mouth, randomized controlled clinical trial, li et al. 2022 | Assessment | low | high | low | low | low | low |
|  | justification | Allocation concealment was maintained using sequentially numbered, opaque, sealed envelopes that were opened at the time of treatment, which meets the criteria for Low. | Due to the invasive nature of the MOP procedure, blinding of participants and clinical personnel was explicitly stated as impossible, leading to a high risk of performance bias. | Outcome assessments (measurements from dental casts) were performed by a blinded assessor, effectively reducing the risk of detection bias. | All 20 participants completed the study, and no missing data or attrition were reported, suggesting a Low due to incomplete outcome data. | All pre-specified outcomes (rate of tooth movement and pain assessment) were reported fully, clearly aligning with the stated methodology, indicating no selective reporting bias. | The study was methodologically robust, with no additional obvious sources of bias identified. Explicit methodological details and appropriate controls (split-mouth design, standardized protocols) were thoroughly described. |
| 13. The effect of micro-osteoperforations on the rate of maxillary incisors' retraction in orthodontic space closure a randomized controlled clinical trial. Mordente, 2024 | Assessment | low | high | low | low | low | unclear |
|  | justification | Allocation concealment was maintained by using sequentially numbered, opaque, sealed envelopes that were opened only at the time of allocation, adequately preventing selection bias. | Blinding was not possible due to the invasive nature of the intervention (MOP procedure), meaning participants and clinical personnel were aware of the treatment assignments, thus indicating a high risk of performance bias. | Outcome assessors were blinded during measurements. Digital models and CBCT scans were coded to ensure that the assessor did not know the group allocation, thus effectively reducing detection bias. | Attrition was transparently reported (initially 42 participants, with 37 analyzed due to clear, justifiable reasons such as irregular attendance, pregnancy, and equipment failure). This minimal attrition is unlikely to have significantly affected outcomes, representing a Low. | All pre-specified outcomes—rate of incisors' retraction, anchorage loss, root resorption, and tooth inclination—were comprehensively reported and clearly aligned with the study protocol, indicating no evidence of selective reporting. | Two participants were non-randomly reassigned from the MOP group to the control group due to anatomical constraints (risk of root injury). Although this decision was justified ethically, it introduces potential bias into group comparability and represents an unclear risk of bias. |
| 14. Comparison of the effectiveness of piezocision-aided canine retraction augmented with micro-osteoperforation: a randomized controlled trial, Husain et al. ,2024 | Assessment | low | high | low | low | low | unclear |
|  | justification | Allocation concealment was ensured using opaque, sealed envelopes, which is a standard, low-risk method that prevents foreseeing the assignment. | The authors explicitly state that blinding of patients and operators was not possible due to the nature of the interventions. This lack of blinding could have introduced bias in performance, particularly in perception or reporting of subjective outcomes. | Outcome assessors were blinded through data concealment during measurement and analysis. Since outcomes were digital and radiographic measurements, the risk of detection bias is low when assessors are blinded. | Although 2 out of 25 participants were lost to follow-up, an intent-to-treat analysis was performed, and reasons for dropout were not linked to outcomes, minimizing risk of bias due to missing data. | The outcomes specified in the methods section (rate of canine retraction, angular changes, and bone thickness) were all reported in the results. The study appears to report all prespecified outcomes in full. | While the study design is solid and well-documented, a potential crossover effect in the split-mouth design is acknowledged by the authors but not quantitatively addressed. This might introduce bias but is not sufficient for a high-risk judgment without additional data. |
| 15. Effects of micro-osteoperforations on intraoral miniscrew anchored maxillary molar distalization: A randomized clinical trial , Gulduren et al. ,2020 | Assessment | low | high | low | low | low | low |
|  | justification | Allocation concealment was achieved using opaque, sealed envelopes drawn randomly by participants. This process ensures that treatment allocation was not predictable or visible in advance. | Due to the invasive nature of MOP procedures and their physical sensation, it is unlikely that participants and personnel were blinded. The paper confirms that blinding was not feasible for participants or treating clinicians, which could introduce performance bias. | The study explicitly states that all digital models were coded to blind investigators during outcome assessment, and a blinded periodontist conducted the clinical evaluations. This minimizes the risk of detection bias. | Out of 20 randomized participants, 18 completed the study. The dropouts were equally distributed between groups and were due to missing follow-ups, not related to outcomes. No differential attrition or impact on data integrity was evident. | All prespecified outcomes mentioned in the methods (rate of tooth movement, pain, periodontal parameters, etc.) were fully reported in the results with appropriate statistics and discussion. No indication of selective reporting was found. | The study used a split-mouth design and added a parallel control group to control for crossover effects. The sample was well-matched at baseline, and all procedures were standardized. No other design or execution concerns were identified. |
| 16. Effects of mini-implant facilitated micro-osteoperforations in alleviating mandibular anterior crowding: A randomized controlled clinical trial , Bansal et al. ,2019 | Assessment | low | high | low | low | low | low |
|  | justification | Allocation concealment was properly conducted using sealed, opaque, similar-looking envelopes shuffled before selection by participants. This prevents foreknowledge of group assignments. | Participants and the treating operator were aware of the group assignments, which is explicitly stated. Due to the nature of MOP procedures, blinding was not feasible, posing a high risk of performance bias. | The assessors were blinded during measurements of all primary and secondary outcomes (e.g., LII, CBCT analyses). Blinding was ensured via coded models and patient identifiers, reducing detection bias. | All 30 enrolled participants completed the study with no dropouts or exclusions reported. Complete follow-up data were obtained and analyzed for every participant. | The study reported all prespecified outcomes (alignment time, root resorption, bone height loss, pain, satisfaction). Trial registration and protocol adherence were mentioned, supporting transparency. | The groups were comparable at baseline, and interventions were standardized. No unusual deviations, early stopping, or financial conflicts were reported. No other apparent risks were identified. |
| 17. Efficacy of micro-osteoperforation of the alveolar bone by using mini-screw for acceleration of maxillary canine retraction in young adult orthodontic patients: A split-mouth randomized clinical trial, Golshah et al., 2021 | Assessment | low | high | low | low | low | unclear |
|  | justification | Allocation concealment was ensured using opaque, sealed envelopes, opened only after randomization. The envelopes were prepared and handled by an independent observer, making foreknowledge of allocation unlikely. | Blinding of participants and the clinician was not feasible due to the surgical nature of MOP, and the authors acknowledge this. This presents a high risk for performance bias, especially for any subjective assessments or care decisions. | Data collection and analysis were conducted by a blinded assessor who was unaware of the study hypothesis and group allocation. This effectively reduces detection bias for the main outcome measurements. | All 25 patients completed the trial, and outcome data for the entire cohort were analyzed. There were no dropouts reported, minimizing risk from missing data. | The study was registered prospectively, and both primary and secondary outcomes (canine movement and tipping) were reported as planned. There is no evidence of missing or selectively reported outcomes. | Although the study is well-conducted, the use of a split-mouth design introduces the potential for crossover effects, which is not fully addressed. Additionally, the sole use of one operator and single-center design could limit external validity. |
| 18. Efficacy of micro-osteoperforations on the rate of maxillary canine retraction: A randomized controlled trial, El Awady et al. ,2019 | Assessment | unclear | high | unclear | low | unclear | low |
|  | justification | While coin tossing was used to allocate interventions, the study does not describe how allocation concealment was maintained. There is no mention of sealed envelopes or central randomization, so concealment cannot be confirmed. | There is no indication that participants or clinicians were blinded, and given the physical nature of the MOP intervention, blinding would be difficult. This opens the door to performance bias. | The same investigator conducted the outcome measurements using calipers, but the study does not specify whether this individual was blinded to the intervention side. This lack of clarity limits the ability to assess detection bias confidently. | All 12 participants completed the study, and no dropouts or missing data were reported. The full dataset was analyzed, indicating a low risk from attrition. | There is no mention of a registered protocol or trial registry. Although the outcomes mentioned in the methods were reported, the lack of protocol prevents verification of selective reporting. | The split-mouth design reduces inter-subject variability, and the procedures appear standardized. There are no apparent methodological issues like early stopping or extreme baseline imbalances. |
| 19. Evaluation of physiodispenser assisted micro-osteoperforation on the rate of tooth movement and associated periodontal tissue status during individual canine retraction in first premolar extraction cases, Thomas et al. ,2021 | Assessment | low | high | low | low | low | low |
|  | justification | Allocation sequence was concealed using sequentially numbered opaque sealed envelopes, ensuring that the allocation was not predictable or revealed before assignment. | Due to the physical and invasive nature of MOP, blinding of participants and clinicians was not possible. The authors acknowledge the lack of blinding, which increases risk of performance bias. | The investigator who collected and calibrated the outcome data, as well as the statistician, were both blinded to group assignment. This approach minimizes detection bias for outcome measurement. | Three participants were excluded for irregular attendance, but 30 out of 33 participants completed the study, with no evidence of differential attrition or bias from missing data. Reasons for dropout were reported and not related to outcomes. | The trial was registered prospectively (CTRI/2019/07/020403), and the reported outcomes match those stated in the objectives and methods, suggesting full and transparent reporting. | The study used rigorous CBCT-based measurements, standardized methods, and minimized bias from anchorage loss. There were no indications of baseline imbalances, early stopping, or other methodological flaws. |
| 20. Evaluation of the Effect of Micro-osteoperforations versus Piezopuncture on the Rate of Orthodontic Tooth Movement Associated with Canine Retraction", Farag et al. ,2021 | Assessment | unclear | high | unclear | low | unclear | low |
|  | justification | While randomization was done via computer software, there is no mention of how allocation was concealed (e.g., sealed envelopes, central allocation). Without this information, we cannot confirm whether allocation was free from foreknowledge. | Given the physical and surgical nature of both MOPs and piezocision interventions, blinding of participants and operators was not possible. The study does not mention any attempt at performance blinding, which poses a High. | Outcome assessments were conducted via direct intraoral measurements by digital caliper. The study does not specify whether the assessors were blinded to group assignments, leaving detection bias risk unclear. | All 20 enrolled patients completed the study with no dropouts reported. This complete dataset indicates a low risk from incomplete outcome data. | The study does not reference a pre-registered protocol or trial registry, which makes it difficult to verify if all planned outcomes were reported. Although results seem comprehensive, the absence of protocol transparency renders the risk unclear. | The split-mouth design minimizes inter-patient variability, and anchorage control was well-managed using miniscrews. No significant design flaws, baseline imbalances, or early terminations were reported. |
| 21. Micro-osteoperforation effectiveness on tooth movement rate and impact on oral health–related quality of life: A randomized clinical trial ,Fattori et al. ,2020 | Assessment | low | high | low | low | low | low |
|  | justification | Allocation concealment was maintained by coding participants with letter-number combinations before randomization by an independent investigator, preventing selection bias. | Blinding of participants and personnel was not possible due to the nature of the MOP intervention. The authors acknowledge the lack of blinding, which may have influenced subjective outcomes such as quality of life. | Outcome assessors and data analysts were blinded to group allocation. This reduces the likelihood of detection bias, especially for objective 3D digital measurements and OHIP-14 responses. | Out of 24 initially enrolled participants, 18 completed the study for the primary outcome, and 22 completed the quality-of-life assessment. Dropouts were reported and handled appropriately without imbalance between groups. | The trial was pre-registered on ClinicalTrials.gov (NCT02416297), and all prespecified outcomes (tooth movement rate and OHRQoL) were reported in detail. There was no indication of selective reporting. | The groups were comparable at baseline, and procedures were standardized. No unusual methodology issues, early trial stoppage, or significant confounding factors were identified. |
| 22. "Mini-implant supported canine retraction with micro-osteoperforation: A split-mouth randomized clinical trial" by Sivarajan et al. (2019) | Assessment | low | high | low | low | low | low |
|  | justification | Allocation to intervention groups and control sides was performed using coded randomization and drawing lots, limiting foreknowledge of assignment and maintaining adequate concealment in this split-mouth design. | Due to the surgical nature of MOP, participants and clinicians were not blinded. The study explicitly notes that blinding at this stage was not feasible, introducing a high risk of performance bias. | Data were coded and processed by blinded outcome assessors and statisticians. This ensured that detection bias during measurement and analysis was minimized. | All 30 enrolled participants completed the study with no reported dropouts. The completeness of data eliminates attrition-related bias. | The trial was registered (DF CD1412/0089P), and all planned outcomes (rate of canine retraction, pain, and impact on daily function) were reported transparently. No signs of unreported prespecified outcomes were noted. | Groups were balanced, measurements had excellent calibration, and intervention application was standardized. No evidence of early trial stopping, baseline imbalances, or methodological flaws was found. |
| 23. Osseous evidence behind micro-osteoperforation technique in accelerating orthodontic tooth movement: A 3-month study" by Asif et al. (2020) | Assessment | unclear | high | low | low | low | low |
|  | justification | The intervention involved visible and invasive procedures (MOP), making blinding of participants and clinicians not feasible. The lack of blinding could influence both perception and behavior, leading to performance bias. | The intervention involved visible and invasive procedures (MOP), making blinding of participants and clinicians not feasible. The lack of blinding could influence both perception and behavior, leading to performance bias. | The BV/TV analysis was conducted using coded images by two blinded examiners. The outcome assessors were unaware of MOP frequency allocation, which sufficiently mitigates detection bias. | The study initially enrolled 30 participants to account for dropouts and completed the study with 24. The dropouts were anticipated, and analysis was completed on all remaining participants without missing data, indicating a low attrition bias. | All prespecified outcomes—canine movement and BV/TV ratio—were fully reported, including subgroup analyses by MOP frequency. The report appears comprehensive, and the study objectives align with the reported results. | Calibration and reliability testing were performed for measurements, and standardized methods were used throughout. No baseline imbalances, early stoppages, or other methodological concerns were identified. |
| 24. "The use of micro-osteoperforation concept for accelerating differential tooth movement" by Feizbakhsh et al. (2018) | Assessment | unclear | high | low | low | unclear | low |
|  | justification | Although randomization is described, the study does not mention whether allocation was concealed (e.g., through sealed envelopes or third-party assignment). Without this detail, allocation concealment cannot be confidently assessed. | Given the surgical nature of MOPs and the use of physical perforations, neither participants nor clinicians were blinded. No steps to mitigate this issue are mentioned, and performance bias is therefore likely. | The operator who conducted the outcome measurements was blinded to which side was experimental or control. The use of 3D scanned models and digital software further reduces detection bias. | All 20 participants completed the study, and no dropouts or missing data are reported. Full outcome data were obtained and analyzed, suggesting low attrition bias. | While all outcomes mentioned in the methods are reported in the results, there is no indication of trial registration or access to a study protocol to verify whether all prespecified outcomes were reported. | Baseline conditions were well controlled (e.g., same surgeon, identical appliances, similar occlusal conditions), and no early stopping or financial conflicts were noted. The design appears otherwise robust. |
| 25."To evaluate the rate of canine retraction and pain perception following micro-osteoperforation – a split-mouth clinical study" by Martina et al. (2022) | Assessment | unclear | high | low | low | low | low |
|  | justification | The study does not specify how the allocation sequence was concealed (e.g., sealed envelopes, third-party allocation). Without details on concealment, the risk of selection bias cannot be ruled out. | Blinding of participants and clinicians was not possible due to the invasive nature of MOPs, and the authors clearly state this. The lack of blinding could have influenced subjective reporting or clinical behavior. | Although operators and patients were unblinded, the data analyst was blinded to both group assignment and time intervals. This blinding of the outcome assessment process reduces risk of detection bias. | All 40 enrolled patients completed the study without loss to follow-up, and complete data were reported and analyzed. No attrition bias is apparent. | The trial was registered (CTRI/2022/03/XXXXXX), and all planned primary (canine retraction) and secondary (pain perception) outcomes were fully reported. There is no evidence of selective reporting. | The study used a sound split-mouth design with standardized protocols, appliance systems, force application, and anchorage. No significant methodological flaws or imbalances were reported. |
| 26. Accelerated Orthodontic Tooth Movement in Adult Patients by Micro-perforations of Cortical Bone, Tina Mahmoudi, Monica Schneider, Glen Minah, Bhoomika Ahuja, Eung Kwon Pae, 2016 | Assessment | Unclear | High risk | Low risk | Low risk | Unclear | Low risk |
|  | justification | The paper provides no information about how allocation was concealed from investigators enrolling participants, such as using sealed opaque envelopes or central allocation. | Due to the nature of the intervention (micro-osteoperforation), the personnel performing the procedure could not be blinded, and participants likely knew which side received the intervention since it involved local anesthesia and actual perforations. | The study explicitly states that “All landmarks were digitized and measurements were performed by two separate operators who were blinded to the experimental procedures involved” and outcome assessors were blinded to which side received the intervention. | The study reported that although 14 patients were initially recruited, 4 did not start the experimental phase for reasons unrelated to the intervention. All 10 participants who initiated the experimental phase completed the study with no loss to follow-up, as stated: “The ten remaining subjects initiated the experimental phase and completed the study with no loss to follow up.” | There is no reference to a pre-registered protocol or pre-specified outcomes, making it impossible to determine if all planned outcomes were reported. | The study appears free of other major sources of bias. Although there are limitations related to sample size and variability in response, these are acknowledged and do not appear to introduce systematic bias. |
| 27. Clinical Evaluation of the Effect of Micro-Osteoperforations on the Rate of Tooth Movement During Alleviation of Mandibular Anterior Crowding, Haya Al-Hayek, Islam Tarek Abbas, Noha Ezzat Sabet, 2019 | Assessment | Unclear | High risk | Unclear | Unclear | Unclear | Unclear |
|  | justification | No information is provided about how the allocation sequence was concealed from investigators enrolling participants, making it impossible to determine if foreknowledge of assignments was prevented. | Due to the nature of MOPs (surgical intervention), participants and operators could not be blinded to group assignment. This may introduce performance bias, especially in pain perception reporting. | While assessment of alignment was “assessed visually by 2 operators,” the study doesn’t specify whether these assessors or those evaluating CBCT images for root resorption were blinded to group allocation. | Five participants (19%) were lost to follow-up (3 from control, 2 from MOP group), but no reasons were provided for these losses, making it impossible to determine if the missing data might relate to treatment outcomes. | The study does not mention a pre-registered protocol, so it is unclear whether all pre-specified outcomes were reported. However, the main outcomes (rate of tooth movement, pain perception, and root resorption) appear to be reported comprehensively. | Baseline characteristics were not fully reported (e.g., initial crowding severity per group). Potential imbalance in covariates could influence results, but insufficient data to confirm |
| 28. Comparison of rate of tooth movement and pain perception during accelerated tooth movement associated with conventional fixed appliances with micro-osteoperforations – a randomised controlled trial, Sonal Attri, Rashmi Mittal, Puneet Batra, Saurabh Sonar, Karan Sharma, Sreevatsan Raghavan, Kriti Sharma Rai, 2018 | Assessment | Unclear | High risk | Low risk | Low risk | Unclear | Low risk |
|  | justification | The study states that sequences were concealed in envelopes chosen by the patient, independently of the investigators. However, it does not specify whether the envelopes were sequentially numbered, opaque, and sealed. | The authors explicitly state that blinding of participants and treating clinicians was not possible due to the nature of the MOP intervention (receiving or performing the perforations). | The data analyser, responsible for measuring tooth movement on coded digital models and assessing coded VAS scores, was blinded to group allocation. This blinding minimizes the risk of detection bias in outcome assessment. | All 60 randomized participants completed the study follow-up and were included in the analysis, as stated in the results and shown in the CONSORT flow diagram. | Although the trial was registered and reported outcomes align with those described in the methods, there’s insufficient information about the pre-registered protocol content to verify complete reporting of all planned outcomes. | Baseline characteristics were comparable between groups, no conflicts of interest were declared, and the study protocol appears sound. No other significant issues like inappropriate early stopping or major design flaws were apparent. |
| 29. Effect of micro-osteoperforation on the rate of canine retraction: a split-mouth randomized controlled trial,  Amira A. Aboalnaga, Mona M. Salah Fayed, Noha A. El-Ashmawi, Sanaa A. Soliman, 2019 | Assessment | Low risk | High risk | Low risk | Low risk | Unclear | Low risk |
|  | justification | Allocation was concealed using opaque, sealed envelopes containing the subject numbers linked to the random sequence. Participants only selected an envelope immediately before the intervention, preventing foreknowledge of the assigned treatment side. | Blinding of participants is inherently impossible due to the nature of the MOP intervention (a minor surgical procedure on one side versus none on the control side). The study does not mention blinding of the personnel applying the orthodontic forces or managing the patient during follow-up. | The study explicitly states that the primary assessor responsible for measuring outcomes from digital models and CBCT scans was blinded to the allocation group. | The report clearly states that all participants who were initially enrolled completed the entire 4-month study duration. There is no mention of dropouts or missing data for the assessed outcomes. | Although the trial was registered, the registration occurred after the trial had concluded. While the paper reports on primary and secondary outcomes consistent with the abstract and methods, the late registration prevents verification against a pre-specified protocol. Therefore, it cannot be confirmed with certainty that all pre-specified outcomes were reported as planned. | The study employed an appropriate split-mouth design, performed a sample size calculation, used suitable statistical methods based on data normality checks, and declared no conflicts of interest or external funding. While the retrospective trial registration is a procedural issue (addressed under selective reporting), no other major concerns like extreme baseline imbalance or inappropriate analyses that would introduce significant bias were apparent. |
| 30. Effect of micro-osteoperforations on the rate of tooth movement, Mani Alikhani, Markos Raptis, Billie Zoldan, Chinapa Sangsuwon, Yoo B. Lee, Bandar Alyami, Corey Corpodian, Luz M. Barrera,Sarah Alansari, Edmund Khoo, Cristina Teixeirak, 2013 | Assessment | Unclear | High risk | Low risk | Low risk | Unclear | Low risk |
|  | justification | The paper does not describe the mechanism used to conceal the allocation sequence before or during participant enrollment. It is unclear whether the investigators enrolling participants could have foreseen the assignments. Lack of description regarding concealment methods (like central randomization or sealed opaque envelopes) makes it impossible to judge if it was adequate. | The study explicitly states that both the participants and the residents administering the orthodontic treatment (personnel) were not blinded to the group assignments or the side receiving MOPs. Given that the intervention involves a noticeable procedure and the outcome involves patient experience (discomfort) and potentially operator influence, this lack of blinding presents a high risk of performance bias. The authors acknowledge this lack of blinding directly. | The investigators who performed the outcome measurements (tooth movement on dental casts) and conducted the data analysis were blinded to the group assignments. | The study clearly reports that all twenty patients who were initially recruited completed the study. There was no loss to follow-up mentioned. | No protocol is mentioned, but all expected outcomes (tooth movement, inflammatory markers, pain) are reported. However, without a protocol, selective reporting cannot be ruled out. | No other sources of bias (e.g., extreme baseline imbalance, funding influence) were identified. Conflicts of interest were disclosed (Propel tools purchased, no direct funding). |
| 31. Effect of Micro-Osteoperforation on the Rate of Tooth Movement, PARAMESH GOWDA S V, MAHESH KUMAR Y, 2019 | Assessment | Unclear | High risk | Unclear | Low risk | Unclear | Low risk |
|  | justification | The study provides no information about allocation concealment methods, making it impossible to determine if assignments could have been foreseen before or during enrollment. | Blinding of participants and personnel delivering the intervention was not described and appears impossible given the intervention’s nature (physical MOP procedure vs. no procedure). Participants receiving MOPs would be aware, as would the clinician performing it. | The primary outcome (canine retraction) was measured on dental casts using a digital caliper, a relatively objective method. However, the text does not state whether the individual performing these measurements was unaware of the group or side (MOP vs. non-MOP) allocation for each cast. | The study enrolled 46 participants and provides baseline demographics for this number, seemingly including all in the analysis. The results are presented without mentioning any participant dropouts, withdrawals, or exclusions during the 28-day study period. | The study reports results for canine movement (the primary outcome described) and pain/discomfort levels, which seem appropriate based on the methodology. However, the document does not mention a pre-registered protocol or pre-specified primary/secondary outcomes. Without access to a protocol, it cannot be confirmed whether all intended outcomes were reported or if analyses were conducted exactly as planned. | The study includes a sample size calculation, uses a relevant comparison (split-mouth and separate control), and seems free from obvious issues like inappropriate early stopping or major baseline imbalances (based on reported age/sex). The short follow-up (28 days) is consistent with evaluating the initial rate acceleration and doesn’t necessarily introduce bias for that specific question. No other significant sources of bias are apparent. |
| 32. Effect of Micro-Osteoperforations on Orthodontic Tooth Movement with Clear Aligner Treatment, DoBin Choi, Peter Ngan, Osama Mukdadi, Chris Martin, 2017 | Assessment | High risk | High risk | Unclear | Low risk | Unclear | High risk |
|  | justification | As a retrospective study examining previously treated patients, allocation concealment wasn’t implemented during the original treatment assignment. The study mentions “Each subject was given a random identification number” only for de-identification during analysis, not for allocation concealment during treatment. | Due to the nature of the intervention and the significantly different aligner wear schedules (3 vs 14 days), neither the original participants nor the treating personnel could have been blinded during the treatment phase. | The study used CloudCompare software for objective measurements and mentioned intra-operator reliability testing (98.8%), but doesn’t explicitly state whether the person performing measurements was blinded to group allocation. The de-identification process suggests some attempt at blinding, but it’s insufficiently described. | The study reports complete data for all included cases, with no missing outcomes or exclusions after enrollment. Reasons for exclusion (e.g., incomplete scans) are clearly stated. | The study doesn’t mention a pre-registered protocol or pre-specified outcomes, making it difficult to assess if all planned outcomes were reported. However, they do report comprehensive results for all measured parameters across various analyses. | Major confounding factors exist: (1) Different aligner wear protocols between groups (3 days vs. 14 days); (2) Baseline differences in age (control: 29.3 years vs. experimental: 34.1 years) and gender distribution; (3) Inherent limitations of the retrospective design with potential selection bias. The most significant concern is that the experimental group not only received MOPs but also had a dramatically different aligner change protocol (3 days vs. 14 days), making it impossible to attribute differences in outcomes solely to MOPs. |
| 33. Assessment of Rate and Type of Tooth Movement in Enmass Retraction with Micro-Osteoperforation Aided by Excellerator Device, AKSHATHA et al., 2018 | Assessment | Unclear | High risk | Low risk | Low risk | Unclear | Low risk |
|  | justification | While a random sequence was generated using a lottery, the description does not specify how the allocation was concealed from those enrolling participants before assignment. It’s unclear if the person overseeing the patient picking the slip could have influenced the process or foreseen the assignment. There is insufficient detail to judge whether concealment was adequate. | The intervention involves a noticeable procedure (MOPs) for the experimental group versus no intervention for the control group. This makes blinding of participants and the personnel administering the treatment impossible. This lack of blinding could potentially influence participant reporting or co-interventions, despite the outcome assessor being blinded. | The study explicitly states that the person analyzing the outcome data (digital lateral cephalograms) was blinded. Cephalograms were coded before tracing and measurement by an assistant, preventing the analyzer from knowing the group allocation. This is sufficient for blinding the assessment of the primary and secondary outcomes measured via cephalograms. | The study clearly reports that all 14 randomized participants completed the study and were included in the final analysis. There were no dropouts or loss to follow-up mentioned. Therefore, there are no missing outcome data. | The study reports results for the outcomes mentioned in the methods (rate of retraction and incisor inclination). However, the text does not mention a pre-published protocol or trial registration. Without access to a protocol, it is impossible to definitively determine if all pre-specified outcomes were reported as planned. | The study appears free from other major threats to validity, such as extreme baseline imbalances, fraudulent claims, or inappropriate methods. Baseline characteristics between groups were matched, and no significant issues were identified. |
| 34. Effects of mini-screw-facilitated micro-osteoperforation on the rate of orthodontic tooth movement: a single-center, split-mouth, randomized, controlled trial, Babanouri et al, 2020 | Assessment | Low risk | Low risk | Low risk | Low risk | Low risk | Low risk |
|  | justification | The study used sealed opaque envelopes for allocation concealment, preventing investigators from foreseeing group assignments. | Although the study attempted to blind both participants and the treating orthodontist by creating similar insertions in the gingival tissue on the control side, true blinding was ultimately impossible due to the inherent nature of the intervention. | The outcome assessor (second author) and the statistician were blinded to the intervention allocation, ensuring unbiased measurement and analysis of outcomes. | Although there were 3 dropouts (out of 28 participants), the reasons were clearly stated, unrelated to treatment outcomes, and relatively balanced between groups. | The trial was prospectively registered, and the published report includes results for all pre-specified primary (rate of canine movement) and secondary (pain assessment) outcomes mentioned in the methods section. | The study appears free of other major sources of bias; baseline characteristics were similar between groups, and appropriate statistical methods were used. Although the authors note a limitation regarding pain assessment in a split-mouth design, this is unlikely to significantly bias the primary outcome, and no other significant issues were apparent. |
| 35. Effectiveness of Flapless Cortico-Alveolar Perforations Using Mechanical Drills Versus Traditional Corticotomy on the Retraction of Maxillary Canines in Class II Division 1 Malocclusion: A Three-Arm Randomized Controlled Clinical Trial, Alfailany et al. ,2023 | Assessment | Low risk | High risk | Low risk | Low risk | Low risk | Low risk |
|  | justification | Allocation was concealed using sequentially numbered, opaque, and locked envelopes, which prevents foreknowledge of treatment assignments. Furthermore, the allocation sequence was managed by an independent academic specialist not involved in patient recruitment or treatment. This method meets the criteria for adequate concealment. | The study explicitly states that blinding of participants and practitioners was not possible due to the distinct nature of the surgical interventions (traditional corticotomy flap vs. flapless perforations vs. no surgery). This lack of blinding could potentially influence behavior or care provided (performance bias), even if the primary outcome measurement itself is objective. The outcome measurement is unlikely to be influenced, but performance bias cannot be ruled out. | The investigators explicitly state that the outcome assessor was blinded to the treatment group allocation. The outcomes were measured from dental casts, which is a relatively objective method. Blinding the assessor minimizes the risk of detection bias during outcome measurement. | The study reported that no patients dropped out during the trial, and all 51 randomized participants were included in the final analysis. The CONSORT flow diagram also confirms this. As there was no missing outcome data, the risk of attrition bias is low. | The trial was pre-registered ( NCT03659188), allowing for verification of outcomes. The paper clearly defines the primary and secondary outcomes (canine retraction, anchorage loss, canine rotation) and reports results for these outcomes at the specified time points. Based on the provided registration, there is no evidence suggesting selective outcome reporting | The study appears methodologically sound with appropriate design, reported sample size calculation, and balanced baseline characteristics. Conflicts of interest and funding sources were declared. No other significant sources of bias, such as early stopping for data-dependent reasons or extreme baseline imbalance, were apparent from the study. |
| 36. Evaluation of Modified Microosteoperforation on the Rate of En Masse Retraction. A Prospective Clinical Study, Rashid, 2018 | Assessment | Unclear | High risk | Unclear | Low risk | Unclear | Low risk |
|  | justification | The paper does not provide any information on whether the allocation sequence was concealed from those enrolling participants or the participants themselves prior to assignment. It is unknown if the researchers could foresee the group assignment for the next participant. | Blinding of participants and personnel was not feasible due to the nature of the intervention; one group received MOPs (a surgical procedure), and the control group did not. This knowledge could influence participant reporting (e.g., pain) or subtle variations in clinician care or adjustments. | The study does not specify whether the outcome assessors measuring tooth movement (using calipers on models) and anchor loss (using cephalometric tracings) were blinded to the group allocation. Pain assessment was done via patient self-report (VAS), and patients were not blinded. | The study recruited 20 participants (10 per group) and appears to report results for all participants throughout the 24-week follow-up period. There is no mention of dropouts, exclusions after randomization, or missing data in the results section. | The study reports on the outcomes stated in its objectives/summary (rate of tooth movement, anchor loss) and also reports on pain assessment. However, the paper does not refer to a pre-published protocol or trial registration. Without a pre-specified protocol, it’s impossible to verify if all intended outcomes were reported as planned, making the risk of selective reporting unclear. | The study includes a sample size calculation, uses standard statistical analyses, and defines clear inclusion/exclusion criteria. Potential confounders like extraction timing were addressed. No significant baseline imbalances were reported, and no other major flaws (e.g., inappropriate study design, early stopping) are apparent that would suggest a high risk of other bias. |
| 37. Evaluation of the Rate of Individual Canine Retraction and Gingival Crevicular Fluid Levels of Interleaukin-1β with Micro-Osteo Perforations – A Split Mouth Study, MUHAMMED ABDUL RASHEED. V, ADUSUMILLI GOPINATH, 2018 | Assessment | High risk | High risk | Unclear | Low risk | Unclear | Low risk |
|  | justification | As the allocation of intervention was fixed (Right=MOPs, Left=Control), it was inherently unconcealed. Both the investigators enrolling participants (if applicable after initial patient selection) and the participants themselves could foresee the assignment for each side. | Blinding of participants and personnel delivering the intervention was not feasible due to the overt nature of the MOPs procedure (requiring local anaesthesia and perforations on one side only). Participants were aware of which side received the MOPs, and the personnel performing the MOPs and potentially subsequent monitoring were also unblinded. This lack of blinding could influence subjective reporting (if any) or differential care/attention, potentially affecting outcomes. | The study does not explicitly state whether the outcome assessors were blinded to the treatment allocation. While measuring canine retraction on dental casts using a digital caliper could potentially be done blindly, it is not mentioned. Similarly, it is not stated if the laboratory personnel performing the ELISA for IL-1β were unaware of the sample origin | The study reports starting with 10 patients (20 samples) and presents results consistently based on this number throughout the tables. There is no mention of participant dropouts or missing outcome data for the reported analyses. Therefore, attrition bias seems unlikely. | The study does not mention referencing a pre-published protocol or trial registration. Without a protocol, it is impossible to determine if all intended outcomes were reported as pre-specified. The reported outcomes (canine retraction rate, IL-1β levels) align with the study’s stated objectives. | The study appears free of other major design-related biases. The split-mouth design inherently reduces variability, and limitations such as sample size and generalizability are acknowledged without introducing systematic bias. |
| 38. The Effectiveness of Micro-osteoperforations during Canine Retraction A Three-dimensional Randomized Clinical Trial, Basema Alqadasi, Khalid Aldhorae, Esam Halboub, Nasrin Mahgoub, Akram Alnasri, Ali Assiry, Hou Y. Xia, 2019 | Assessment | Low risk | High risk | Low risk | Low risk | High risk | Low risk |
|  | justification | The use of opaque, sealed envelopes that were shuffled prior to the participant’s selection ensured that neither the participant nor the investigators could foresee the assignment. | The nature of the MOP intervention (physical perforations on one side) makes blinding of participants inherently impossible. Similarly, the personnel administering the MOPs and subsequent orthodontic adjustments would be aware of the assigned intervention side. | The study explicitly states that blinding was employed during the data collection and analysis stages. Assessing tooth movement on 3D digital models and measurements on CBCT scans are objective outcomes that can be reliably assessed by a blinded evaluator. | The study reports starting with eight patients and presents results seemingly based on all eight participants through the 3-month assessment period. There is no mention of dropouts, withdrawals, or missing outcome data in the article. | The abstract lists several secondary outcomes (anchorage loss, canine tipping, canine rotation, plaque index, gingival index) that are not quantitatively reported in the results section. While primary outcomes and some secondary outcomes (root resorption, pain) are reported, the omission of others mentioned raises concern for selective reporting, especially without a referenced protocol. | The study utilized a split-mouth design, controlling for inter-individual variability, and reported comparable baseline characteristics between the intervention and control sides. Appropriate non-parametric statistical tests were used for the small sample size, and conflicts of interest were declared absent. Although the sample size (N=8) is very small, limiting statistical power and generalizability, no other major sources of bias (e.g., inadequate design elements beyond sample size, baseline imbalance in reported measures, declared conflicts) were apparent. |
| 39. A CBCT assessment of bone density changes after accelerated orthodontic retraction of canine by microosteoperforations,Alfawazan et al. (2022) | Assessment | High | High | unclear | Low | unclear | High |
|  | justification | As there was no sequence of randomization generated, allocation concealment was not feasible. Pre-decided allocation of intervention (MOPs) on the right and control on the left and prior knowledge to investigators and participants regarding the same at and during enrollment allow potential selection bias. | The nature of the intervention (MOPs performed on one side) makes blinding of participants and the personnel performing the procedure impossible. The study does not mention any attempts at blinding these groups. This lack of blinding could introduce performance bias | The protocol clarifies the CBCT measurement process and refers to intra-examiner reliability testing but does not indicate whether the outcome assessor was blinded to group allocation (treatment or control side) for image analysis. Absence of blinding to outcome could result in detection bias. Consequently, the associated risk is unclear. | The trial involved 40 participants in total, and all were analyzed with reference to the set 3-week follow-up time points (T0, T1, T2). There were no participant dropouts or missing data on the primary outcome (bone density) reported. Therefore, the risk of attrition bias for the reported data appears to be low. | The trial specifically outlined its primary outcome (change in bone density) according to the stated aims and methods. However, the protocols outline tooth movement measurement, yet quantitative findings for this outcome are not reported, merely discussed qualitatively. Without an openly available protocol, it is uncertain if all pre-specified outcomes were reported as planned. | the primary issue here, other than those already mentioned, is the use of a fixed, non-random assignment within the split-mouth design (right side always MOP, left side always control). This systematic assignment carries a High, as possible inherent differences between right and left sides were not accounted for by randomization. The short follow-up (3 weeks) is also listed as a limitation by the authors, but perhaps less of a bias for the specific short-term outcome measured. |
| 40. Accelarated Orthodontic Treatment Using Microosteoperforations: A Comparative Study, Sinha et al. (2024) | assessment | unclear | High | unclear | Low | High | Low |
|  | justification | The authors state that “Group allocation was concealed until the beginning of treatment”. However, they do not describe the specific method used to ensure concealment. Without this methodological detail, the adequacy of concealment cannot be confirmed. | The intervention is a surgical procedure (MOPs) that is performed on one group while no such intervention is performed on the other, making it virtually impossible to blind both participants and personnel administering the treatment. The study does not indicate any effort to blind them. The lack of blinding will most likely lead to performance bias. | The manuscript does not report whether outcome assessors measuring the treatment duration and rate of tooth movement were blinded to group allocation. Assessor blinding is necessary, especially for measurements like the rate of tooth movement, to prevent the possibility of detection bias. Insufficient information is provided to make a judgment. | The study recruited 60 participants and the results seem to be based on the analysis of all participants (30 per group), as presented in the tables. There is no mention of dropouts, exclusions after randomization, or missing data. Therefore, the risk of attrition bias appears low. | Patient discomfort was included as an outcome measure, and the abstract/conclusion levels indicate MOPs did not cause additional discomfort. However, the results section does not provide quantitative results, the measurement scale used, or statistical comparison for this outcome. This partial reporting of a reported outcome is a source of concern for selective reporting bias. | he trial appears to be a standard parallel-group RCT of MOPs+orthodontics compared to orthodontics only. There are no significant concerns like large imbalances at baseline (demographics described as similar), wrong study design for question, or other specific concerns which can be appreciated from the given text. Thus, the possibility of other biases does not look significant. |
| 41. Effect of Flapless Cortical Perforation on Canine Retraction Rate: A Randomized Clinical Trial, kundi et Al (2018) | assessment | Low | High | High | Low | unclear | unclear |
|  | justification | The authors clearly mentioned that they used “central concealment” for assigning participants. This strong method prevents anyone enrolling participants from predicting the next assignment, effectively reducing the risk of selection bias during recruitment. | The authors acknowledged that blinding the patients and operators wasn’t possible. Since everyone involved knew the group assignments, there’s a higher chance of performance bias, which could affect behaviors or additional treatments during the study. | The article notes that the first author conducted the measurements, with blinding only applied during the analysis phase. This means the assessor knew which groups the participants belonged to while measuring outcomes, increasing the risk of detection bias, as their knowledge could unintentionally affect the results. | The study reports that all participants completed the trial and were included in the 28-day outcome analysis, with no dropouts. This means there’s little risk of bias from missing data, known as attrition bias. | The study clearly identifies its primary outcome—canine movement—and provides results for it. However, since there’s no mention of a pre-registered protocol, it’s unclear if all planned outcomes were reported as originally intended. This raises the possibility of selective reporting bias. | The study highlights that there were more females in the conventional group than the experimental group, creating a baseline imbalance. While the authors claim gender differences didn’t affect results, they didn’t formally analyze this impact, leaving an uncertain risk of bias. |
| 42. Effect of Frequency of Micro-osteoperforation on Miniscrewsupported Canine Retraction: A Single-centered, Split-mouth Randomized Controlled Trial, Venkatachalapathy et al. (2022) | assessment | unclear | High | Low | Low | unclear | Low |
|  | justification | The article does not provide information about how the allocation sequence was concealed before or during the assignment of the intervention side. This makes it unclear whether the operator or person enrolling participants could anticipate which side would receive MOPs. The lack of detail on concealment methods limits the ability to confidently assess the risk of selection bias. | The lack of blinding for both participants and the orthodontist performing MOPs introduces a high risk of performance bias. Knowledge of group assignments could influence behaviors or the application of co-interventions. | The outcome assessor was blinded, as stated in the study. Measurements were taken from dental casts, further reducing detection bias. Additionally, intra- and inter-observer errors were evaluated. | No loss to follow-up was reported, with all 20 patients contributing data at each specified time point. This consistency minimizes attrition bias. | Ethical approval was mentioned, but there’s no reference to a pre-registered protocol. Without access to the protocol, it’s unclear if all intended outcomes were reported, leaving room for potential selective reporting bias. | The split-mouth design appropriately controls for inter-patient variability. Issues such as miniscrew loosening were managed effectively and are unlikely to introduce significant bias. |
| 43. Effect of micro-osteoperforations on the rate of orthodontic tooth movement and expression of biomarkers: a randomized controlled clinical trial, Raghav et al. (2022) | assessment | Low | High | Low | Low | Low | Low |
|  | justification | To keep the treatment assignments secret, the study used sealed opaque envelopes shuffled by a faculty member before being handed to the operator. This method effectively prevents anyone from knowing the treatment allocation in advance, reducing selection bias. | The authors acknowledged that blinding patients and the orthodontist wasn’t feasible. Without blinding, there’s a higher risk of performance bias, as knowing the intervention could influence behavior or additional treatments. | Blinding was applied during the measurement phase. A separate investigator, unaware of the group assignments, performed the measurements for both canine movement and biomarker analysis. This ensures objective results and minimizes detection bias. | Only 2 out of 30 participants dropped out (about 6.7%), and their reasons for leaving weren’t related to the study outcomes. Because the dropouts were balanced across both sides in the split-mouth design, the remaining data (from 28 participants) is unlikely to be biased. | The study followed a pre-registered protocol (CTRI number provided) and reported all planned outcomes, including the primary (canine movement) and secondary (biomarker levels) measures. This minimizes the risk of selective reporting bias. | The study’s split-mouth design is well-suited for controlling variability between patients. The authors also took steps to address potential confounding factors, and the limitations mentioned don’t significantly undermine the study’s validity. Overall, the study appears robust and free from major design flaws. |
| 44. Effect of Mini‑implant assisted Micro‑osteoperforation on the rate of orthodontic tooth movement—A randomized clinical trial, Singh et al. (2023) | assessment | unclear | High | Low | Low | Low | Low |
|  | justification | Although the study mentions sealed envelopes were used to conceal group assignments, it doesn’t clarify whether the envelopes were opaque or sequentially numbered—important details for ensuring true concealment. Without this information, it’s unclear if the allocation process was fully secure. | While the study claims triple blinding, there’s a potential issue: the operator performing the MOPs (the first author) likely knew the group assignments, even though participants were blinded using a sham procedure. This knowledge introduces a high risk of performance bias, despite efforts to blind participants. | The investigator measuring outcomes and the statistician analyzing the data were blinded. Measurements were taken from coded dental casts, ensuring objectivity and minimizing detection bias for the primary outcome. | Two participants (9%) dropped out after randomization for reasons unrelated to the intervention, like debonding or irregular attendance. Since the dropouts were balanced between groups, the low attrition rate is unlikely to affect the study’s results or introduce bias. The analysis was performed on the remaining 20 participants. | The study adhered to a registered protocol (CTRI number provided) and reported all planned outcomes, including the primary (canine movement) and secondary (pain via VAS). This transparency minimizes the risk of selective reporting bias. | The split-mouth design was appropriate, and the study controlled for potential confounders like timing, anchorage, and force standardization. Although the operator wasn’t blinded, the use of a sham procedure was a strength. No major issues affecting the study’s validity are apparent. |
| 45. Effectiveness of microosteoperforations in accelerating alignment of maxillary anterior crowding in adults: A randomized controlled clinical trial, Shahrin et al. (2021) | assessment | Low | High | Low | Low | Low | unclear |
|  | justification | They did a good job hiding the group assignments using special envelopes (sequentially numbered, opaque, and sealed) managed by a central coordinator. This ensured that the people enrolling participants couldn’t know or influence which group a participant would join, keeping the process fair. | The study openly states that neither the patients nor the person performing the MOPs procedure could be kept unaware of the treatment. Because both knew who was getting what, there’s a high chance their behavior or expectations could have influenced the results (performance bias). | The person measuring the results (using study casts) didn’t know which group the casts belonged to, thanks to renumbering by the coordinator. This blinding was effective and prevents personal biases from affecting the measurements (detection bias). | Only two participants (one from each group) dropped out, and their reasons (pregnancy, missed appointments) weren’t related to the treatment itself. This dropout rate is low and balanced, meaning it’s unlikely to have skewed the final results (low risk of attrition bias). | The trial was registered indicating a publicly available protocol. The primary and secondary outcomes described in the methods are reported in the results. This suggests a low risk of selective outcome reporting. | A potential issue is that different dentists provided the orthodontic care, even though they were supervised and followed protocols. This variation between providers could have influenced how long treatment took, aside from the effect of the MOPs, making it a bit harder to be certain about the MOPs’ specific impact (unclear risk of bias). |
| 46. Evaluation of Micro‑Osteoperforations in Expediting Orthodontic Tooth Movement in Adult Patients, Alam & Alahmari (2024) | assessment | unclear | High | unclear | Low | unclear | unclear |
|  | justification | The manuscript provides no details regarding the mechanism for concealing group allocation prior to assignment. Consequently, it is impossible to determine whether foreknowledge of assignments by those enrolling participants was adequately prevented. | The absence of a sham procedure for the control group resulted in unblinded participants. This, combined with the likely unblinding of personnel administering orthodontic forces, introduces a substantial risk of performance bias affecting behaviours and co-interventions. | While the use of digital models for outcome measurement presents an opportunity for blinding, the study does not explicitly state whether the outcome assessor was masked to group allocation. This lack of information leaves the risk of detection bias uncertain. | The study reports results for the initially recruited sample size (N=30, 15 per group) without mentioning participant withdrawal or exclusion. Assuming complete data reporting, the risk of attrition bias is considered low. | Although the reported outcomes align with those stated in the methods, the absence of a reference to trial registration or a pre-published protocol prevents confirmation that all intended outcomes were measured and reported, leaving a risk of selective reporting. | Insufficient detail regarding key methodological aspects (e.g., precise MOP protocol, measurement specifics) hinders a comprehensive evaluation of the study’s conduct. Furthermore, the limited sample size (n=15 per group) raises concerns about statistical power and the generalizability of the findings, contributing to an unclear risk from other potential biases. |
| 47. Evaluation of the Effect of Combined Low Energy Laser Application and Micro-Osteoperforations versus the Effect of Application of Each Technique Separately On the Rate of Orthodontic Tooth Movement, Abdelhameed & Refai (2018) | assessment | unclear | High | unclear | Low | unclear | Low |
|  | justification | While the method of sequence generation is described, the manuscript fails to detail the mechanism employed for concealing the allocation sequence prior to assignment. This omission makes it impossible to ascertain whether investigators could have anticipated assignments, leaving the risk related to concealment unclear. | The nature of the distinct interventions (MOPs, LLLT, Combined vs. control side) and the absence of reported sham procedures precluded blinding of participants and personnel. This inherent lack of blinding introduces a high risk of performance bias, potentially influencing participant responses or operator actions. | The outcome was assessed via direct intra-oral measurements. However, the study does not report whether the individual performing these measurements was blinded to the group or side allocation. Without this confirmation, the potential for detection bias remains uncertain. | Minimal attrition (one dropout, 3.3%) and low levels of missing data were reported. The analysis appeared robust to these minor data losses. Consequently, the risk of significant attrition bias is considered low. | The primary outcome specified in the methods was reported. Nonetheless, the absence of trial registration or reference to a publicly accessible protocol prevents verification that all prospectively defined outcomes were reported, resulting in an unclear risk of selective reporting bias. | The chosen study design (parallel groups with split-mouth controls) is suitable for the research question, and appropriate measures like mini-screw anchorage were implemented. Based on the provided description, no other substantial threats to the study’s validity were identified. |
| 48. The effects of micro-osteoperforation on upper first molar root resorption and bone density after distalization by miniscrewsupported Fast Back appliance in adults: A CBCT randomized controlled trial, Alkasaby et al. (2022) | assessment | Low | High | Low | Low | unclear | Low |
|  | justification | Concealment was achieved through sequentially numbered, opaque, sealed envelopes selected by participants, linking them to the pre-generated sequence. | The study explicitly acknowledges the impossibility of blinding participants or the operator performing the intervention. This inherent lack of blinding creates a significant risk of performance bias influencing participant behaviour or operator conduct. | Outcome assessors evaluating CBCT images were successfully blinded through file coding. This measure adequately protects against detection bias potentially influencing the assessment of root resorption and bone density outcomes. | All participants randomized into the study completed the trial and were included in the final analysis, as confirmed by the CONSORT diagram. The absence of attrition minimizes the risk of bias arising from incomplete outcome data. | While a registration identifier is provided, its non-standard format hinders access to and verification of a pre-registered protocol. Although reported outcomes appear consistent with stated objectives, the inability to confirm adherence to a pre-specified plan leaves an unclear risk of selective outcome reporting. Other Potential Threat | The study employed appropriate methodologies (e.g., CBCT imaging, standardized mechanics) and reported no major baseline imbalances. While specific inclusion criteria (females, age range) affect external validity, they do not introduce significant bias to the internal validity of the trial. No other substantial threats were apparent. |
| 49. The use of micro-osteoperforation concept for accelerating differential tooth movement, Ahsan et al. (2021) | assessment | unclear | High | High | Low | unclear | High |
|  | justification | The study does not clarify whether allocation was concealed (e.g., sealed envelopes, central randomization). Since this was a split-mouth design with fixed sides (right for MOP, left for control), bias could arise if researchers or participants anticipated assignments. | Given the nature of MOP (a surgical intervention on one side only), neither patients nor clinicians could be blinded. This introduces performance bias, particularly if subjective assessments were involved. | The same clinician measured tooth movement, and there is no mention of blinding. Since measurements were taken clinically (Vernier calipers), assessor knowledge of the intervention side may have influenced results. | All 30 participants completed the study without dropouts, ensuring complete outcome data. Thus, attrition bias is unlikely. | No protocol was referenced, making it unclear whether all planned outcomes were reported. While the primary outcome (tooth movement rate) was presented, we cannot rule out unreported secondary measures. | The study was labeled “quasi-experimental,” and the intervention side (right) was non-randomly fixed for all participants. Additionally, the short follow-up (3 weeks) limits assessment of long-term effects, potentially skewing results. |
| 50. Three-dimensional assessment of accelerating orthodontic tooth movement - microosteoperforations versus piezocision: A randomized, parallel group and split-mouth controlled clinical trial, Alqadasi et al. (2024) | assessment | Low | High | Low | Low | unclear | unclear |
|  | justification | The team successfully concealed the upcoming assignments using opaque, sealed envelopes. This method effectively shielded the treatment allocation from both researchers enrolling participants and the participants themselves until the point of intervention, thus protecting the randomization process. | Due to the nature of the MOPs and Piezocision procedures, it was impossible to keep participants or the clinical team unaware of which treatment was being applied to which side. This lack of blinding inevitably introduces a high risk of performance bias, as expectations could influence behaviors or responses during treatment. | Assessors measuring the outcomes (tooth movement, root resorption, bone height) were kept unaware of the treatment assignments. This was achieved by using coded digital models and CBCT scans, ensuring that measurements were objective and free from detection bias. | Although three patients dropped out, this represented a small fraction (12.5%) of the group, and their reasons (missed appointments) seem unrelated to the study outcomes. The dropout rate was similar between groups, and the analysis accounted for the missing data, making significant attrition bias unlikely. | While the study reported on the outcomes clearly stated in its methods section, it didn’t reference a pre-registered protocol. Without access to a protocol, we cannot be completely certain that all pre-specified outcomes and analyses were reported, leaving an unclear risk of selective reporting bias. | The study noted some significant differences between the MOPs and Piezo groups at the start of the trial (e.g., baseline bone height). Even though the split-mouth design helps control for individual patient variation, these initial imbalances could potentially confound the direct comparison between the two techniques, creating an unclear risk of bias. |
| 51. Three-dimensional assessment of the effect of micro-osteoperforations on the rate of tooth movement during canine retraction in adults with Class II malocclusion: A randomized controlled clinical trial, Alkebsi et al. (2018) | assessment | Low | High | Low | Low | Low | Low |
|  | justification | The team effectively concealed the allocation sequence using opaque, sealed envelopes, which participants picked themselves. This is a strong method for preventing anyone involved from knowing the upcoming assignment in advance, thereby protecting the randomization and minimizing selection bias. | As acknowledged by the authors, keeping the participants and the clinician administering the treatment unaware of which side received the MOPs was impossible. This inherent lack of blinding creates a high potential for performance bias, where expectations could influence participant-reported outcomes or subtle aspects of care. | Crucially, those measuring the study’s main outcomes (like tooth movement on 3D models) were kept unaware of which side had received the MOPs. They achieved this by coding the data, ensuring objective measurements free from potential detection bias. | Only a small number of participants (3 out of 35) did not complete the study, and their reasons for leaving were unrelated to the treatment’s effects. Such minimal and balanced attrition is unlikely to skew the overall findings, indicating a Low from incomplete data. | The study was registered prospectively (ClinicalTrials.gov), providing a public record of intended outcomes before results were known. The publication thoroughly reports on the primary and all secondary outcomes outlined in the methods section, suggesting comprehensive and unbiased reporting. | the study appears free from other significant concerns. It used a robust split-mouth design, took steps to control potential confounding factors (like standardizing procedures and addressing occlusal interferences), and was transparent about funding. |

# Appendix Figure 1. Change of SMD from Baseline to 4th week Forest Plot

# Appendix Figure 2. Change of SMD from Baseline to Last Measurements Forest Plot

# Appendix Figure 3. Subgroup Analysis Based on Countries (WHO Classification) Forest Plot

# Appendix Figure 4. Subgroup Analysis Based on Study Designs Forest Plot

# Appendix Figure 5. Subgroup Analysis Based on Instrument that is Used for MOP Forest Plot

# Appendix Figure 6. Subgroup Analysis Based on Instruments that is used for Distance Measurement Forest Plot

# Appendix Figure 7. Subgroup Analysis Based on Type of Malocclusion Forest Plot

# Appendix Figure 8. Subgroup Analysis Based on Measured Distance Forest Plot

# Appendix Figure 9. Subgroup Analysis Based on Brackets Forest Plot

**Forest Plot Categories**

(Wires Dimensions (inch × inch)):

A: 0.019 × 0.025

B: 0.017 × 0.025

C: 0.016 × 0.022

D: 0.018 × 0.025

# Appendix Figure 10. Subgroup Analysis Based on Wires Dimensions Forest Plot

# Appendix Figure 11. Subgroup Analysis Based on Jaw Forest Plot

# Appendix Figure 12. Subgroup Analysis Based on Type of Orthodontic Tooth Movement Forest Plot

# Appendix Figure 13. Subgroup Analysis Based on Jaw and Type of Orthodontic Tooth Movement Forest Plot

# Appendix Figure 14. Subgroup Analysis Based on Palatal Perforations Forest Plot

# Appendix Figure 15. Subgroup Analysis Based on Depth of Perforations Forest Plot

# Appendix Figure 16. Subgroup Analysis Based on Percentage of Females Forest Plot

# Appendix Figure 17. Subgroup Analysis Based on Number of Perforations Forest Plot

# Appendix Figure 18. Subgroup Analysis Based on Depth of Perforations and the Instrument that is Used for MOP Forest Plot

# Appendix Figure 19. Subgroup Analysis Based on Depth of Perforations and the Jaw(s) Forest Plot

# Appendix Figure 20. Subgroup Analysis Based on Depth and Total number of Perforations Forest Plot

# Appendix Figure 21. Subgroup Analysis Based on Total Number of Perforations and Study Designs Forest Plot

# Appendix Figure 22. Subgroup Analysis Based on Number of Total Perforations and Jaw(s) Forest Plot

# Appendix Figure 23. Subgroup Based on Total Number and Depth of Perforations and Jaw(s) Forest Plot

# Appendix Figure 24. Subgroup Analysis Based on Repeat of MOP Forest Plot

# Appendix Figure 25. Subgroup Analysis Based on ROB Domain (Random Sequence Generation Domain) Forest Plot

# Appendix Figure 26. Subgroup Analysis Based on ROB Domain (Allocation Concealment) Forest Plot

# Appendix Figure 27. Subgroup Analysis Based on ROB Domain (Blinding of Outcome Assessment) Forest Plot

# Appendix Figure 28. Subgroup Analysis Based on ROB Domain (Incomplete Outcome Data) Forest Plot

# Appendix Figure 29. Subgroup Analysis Based on ROB Domain (Selective Reporting) Forest Plot

# Appendix Table 7. Subgroup Analyses

| **Subgroup** | **Items** | **Study numbers** | **Change of SMD** | **CI (95%)** | **I^2^ (%)** | **p-value**  **(Between Groups)** |
| --- | --- | --- | --- | --- | --- | --- |
| **Based on WHO Classifications** | EMRO | 11 | 2.10 | [1.16 to 3.04] | 94.37 | Raw: < 0.001  B*: < 0.022 |
|  | EURO | 4 | 0.73 | [0.21 to 1.26] | 42.47 |  |
|  | PAHO | 3 | 2.41 | [-0.17 to 4.99] | 94.22 |  |
|  | SEARO | 10 | 0.52 | [0.39 to 0.66] | 86.10 |  |
|  | WPRO | 3 | 0.05 | [-0.40 to 0.51] | 0 |  |
| **Study Design** | Parallel Arms | 17 | 2.27 | [1.50 to 3.04] | 92.26 | Raw: 0.01  B: 0.220 |
|  | Split Mouth | 14 | 1.08 | [0.55 to 1.61] | 87.70 |  |
| **Instrument for MOP** | Propel | 7 | 2.97 | [1.44 to 4.49] | 95.72 | Raw: 0.08  B: 1.000 |
|  | Miniscrew/Mini-implant | 22 | 1.52 | [1.05 to 1.99] | 88.81 |  |
| **OTM Measurement** | Digital Caliper | 16 | 2.43 | [1.70 to 3.16] | 92.64 | Raw: < 0.001  B: < 0.022 |
|  | Digital Scanning | 14 | 0.97 | [0.46 to 1.48] | 83.41 |  |
|  | Combined | 1 | -0.07 | [-0.55 to 0.42] | - |  |
| **Malocclusion** | Bimax protrusion | 1 | 2.69 | [1.84 to 3.53] | - | Raw: 0.03  B: 0.660 |
|  | Class 1 only | 3 | 2.12 | [0.74 to 3.50] | 86.57 |  |
|  | Class 2 only | 7 | 3.06 | [1.33 to 4.80] | 96.40 |  |
|  | Crowding | 5 | 1.71 | [0.64 to 2.78] | 86.83 |  |
|  | Mixed/Multiple | 11 | 1.21 | [0.64 to 1.78] | 85.97 |  |
|  | Other | 4 | 1.05 | [0.10 to 2.00] | 86.63 |  |
| **Type of Bracket** | Edgewise | 1 | 0.17 | [-0.47 to 0.80] | - | Raw: < 0.001  B: < 0.022 |
|  | MBT | 17 | 1.69 | [1.01 to 2.37] | 91.19 |  |
|  | Mini Master | 1 | 1.02 | [0.25 to 1.79] | - |  |
|  | Roth | 5 | 0.51 | [0.17 to 0.85] | 0 |  |
|  | Self-ligating Speed | 1 | 0.19 | [-0.47 to 0.85] | - |  |
| **Working Wire Dimension** | 0.019 × 0.025 | 18 | 1.72 | [1.18 to 2.26] | 89.27 | 0.01  B: 0.220 |
|  | 0.017 × 0.025 | 4 | 1.04 | [0.12 to 1.97] | 87.10 |  |
|  | 0.016 × 0.022 | 2 | 1.03 | [0.58 to 1.47] | 0 |  |
|  | 0.018 × 0.025 | 1 | -0.08 | [-0.92 to 0.76] | - |  |
| **Jaws** | Maxilla | 20 | 1.43 | [0.86 to 2.00] | 91.64 | 0.49  B: 1.000 |
|  | Mandible | 6 | 2.07 | [0.93 to 3.21] | 89.78 |  |
|  | Maxilla and Mandible | 5 | 1.95 | [0.92 to 2.98] | 86.85 |  |
| **Type of OTM** | Alignment | 8 | 1.58 | [0.64 to 2.51] | 89.06 | Raw: < 0.001  B: < 0.022 |
|  | Canine Retraction | 22 | 1.75 | [1.20 to 2.31] | 92.06 |  |
|  | Molar Distalization | 1 | 0.06 | [-0.78 to 0.90] | - |  |
| **Jaws and Type of OTM** | Mandibular Alignment | 5 | 1.71 | [0.64 to 2.78] | 86.83 | Raw: < 0.001  B: < 0.022 |
|  | Mandibular Canine Retraction | 1 | 3.89 | [2.84 to 4.93] | - |  |
|  | Maxillary Alignment | 1 | 0.17 | [-0.47 to 0.80] | - |  |
|  | Maxillary Canine Retraction | 18 | 1.60 | [0.99 to 1.66] | 92.17 |  |
|  | Maxillary and Mandibular Alignment | 2 | 2.24 | [-2.14 to 6.62] | 95.50 |  |
|  | Maxillary and Mandibular Canine Retraction | 3 | 1.91 | [1.27 to 2.55] | 60.01 |  |
|  | Maxillary Molar Distalization | 1 | 0.06 | [-0.78 to 0.90] | - |  |
| **Perforation on palatal** | Yes | 5 | 1.87 | [0.56 to 3.19] | 93.75 | Raw: 0.70 B: 1.000 |
|  | No | 26 | 1.60 | [1.10 to 2.10] | 90.73 |  |
| **Depth** | < 5mm | 19 | 1.77 | [1.25 to 2.29] | 88.86 | Raw: 0.01  B: 0.220 |
|  | >= 5mm | 9 | 0.63 | [0.00 to 1.26] | 83.58 |  |
| **Female** | <= 60% | 18 | 1.77 | [1.14 to 2.40] | 92.01 | Raw: 0.35  B: 1.000 |
|  | > 60% | 9 | 1.30 | [0.55 to 2.05] | 88.94 |  |
| **Total perforation** | <= 3 | 18 | 1.45 | [0.86 to 2.05] | 91.40 | Raw: 0.37  B: 1.000 |
|  | > 3 | 13 | 1.89 | [1.15 to 2.64] | 90.59 |  |
| **Depth and Instrument** | < 5mm with Miniscrew/Mini-implant | 15 | 1.78 | [1.22 to 2.33] | 87.93 | Raw: 0.05  B: 1.000 |
|  | < 5mm with Propel | 4 | 2.00 | [0.45 to 3.56] | 93.03 |  |
|  | >= 5mm with Miniscrew/Mini-implant | 6 | 0.75 | [-0.19 to 1.70] | 88.85 |  |
|  | >= 5mm with Propel | 2 | 0.58 | [-0.23 to 1.39] | 61.15 |  |
| **Depth and Jaws** | < 5mm in Mandible | 3 | 2.26 | [0.36 to 4.46] | 92.10 | Raw: < 0.001  B: < 0.022 |
|  | < 5mm in Maxilla | 12 | 1.42 | [0.81 to 2.03] | 88.53 |  |
|  | < 5mm in Maxilla and Mandible | 4 | 2.36 | [1.44 to 3.29] | 79.61 |  |
|  | >= 5mm in Mandible | 2 | 2.43 | [-0.38 to 5.24] | 94.67 |  |
|  | >= 5mm in Maxilla | 6 | 0.27 | [-0.02 to 0.56] | 0 |  |
|  | >= 5mm in Maxilla and Mandible | 1 | 0.06 | [-0.82 to 0.94] | - |  |
| **Depth and total perforations** | < 5mm and <= 3 | 10 | 1.44 | [0.83 to 2.05] | 85.95 | Raw: < 0.001  B: < 0.022 |
|  | < 5mm and > 3 | 9 | 2.11 | [1.18 to 3.03] | 91.41 |  |
|  | >= 5mm and <= 3 | 6 | 0.27 | [-0.01 to 0.56] | 0 |  |
|  | >= 5mm and > 3 | 3 | 1.63 | [-0.41 to 3.67] | 93.83 |  |
| **Study design and total perforations** | <= 3 in parallel arms study | 8 | 3.05 | [1.71 to 4.39] | 94.36 | Raw: < 0.001  B: < 0.022 |
|  | <= 3 in split mouth study | 10 | 0.67 | [0.19 to 1.14] | 81.26 |  |
|  | > 3 in parallel arms study | 9 | 1.76 | [0.83 to 2.68] | 89.98 |  |
|  | > 3 split mouth study | 4 | 2.21 | [0.77 to 3.64] | 93.25 |  |
| **Total perforations and jaw** | <= 3 in Maxilla | 15 | 1.58 | [0.88 to 2.27] | 92.41 | Raw: 0.16  B: 1.000 |
|  | <= 3 in Maxilla and Mandible | 3 | 1.14 | [0.23 to 2.05] | 79.68 |  |
|  | > 3 in Mandible | 6 | 2.07 | [0.93 to 3.21] | 89.78 |  |
|  | > 3 in Maxilla | 5 | 1.13 | [0.09 to 2.16] | 90.62 |  |
|  | > 3 in Maxilla and Mandible | 2 | 3.47 | [1.68 to 5.25] | 73.98 |  |
| **Jaw and total perforations and depth** | <= 3, < 5mm in Maxilla | 8 | 1.45 | [0.68 to 2.21] | 87.85 | Raw: < 0.001  B: < 0.022 |
|  | <= 3, < 5mm in Maxilla and Mandible | 2 | 1.62 | [1.17 to 2.07] | 0 |  |
|  | <= 3, >= 5mm in Maxilla | 5 | 0.30 | [-0.01 to 0.60] | 0 |  |
|  | <= 3, >= 5mm in Maxilla and Mandible | 1 | 0.06 | [-0.82 to 0.94] | - |  |
|  | > 3, < 5mm in Mandible | 3 | 2.26 | [0.36 to 4.16] | 92.10 |  |
|  | > 3, < 5mm in Maxilla | 4 | 1.39 | [0.17 to 2.61] | 92.13 |  |
|  | > 3, < 5mm in Maxilla and Mandible | 2 | 3.47 | [1.68 to 5.25] | 73.98 |  |
|  | > 3, >= 5mm in Mandible | 2 | 2.34 | [-0.38 to 5.24] | 94.67 |  |
|  | > 3, >= 5mm in Maxilla | 1 | 0.06 | [-0.78 to 0.90] | - |  |
| **Repeat of MOP**** | Yes | 9 | 1.04 | [0.44 to 1.64] | 78.64 | Raw: 0.07  B: 1.000 |
|  | No | 30 | 1.73 | [1.28 to 2.17] | 90.05 |  |

* B: Bonferroni‑adjusted p

** Despite of all other rows which belongs to change of SMD from baseline to 4weeks, this sub group belongs to baseline to last measurement

# Appendix Table 8. Subgroup Analyses Based on Tooth movement

| **Subgroup** | **Items** | **Study numbers** | **Change of MD** | **CI (95%)** | **I^2^ (%)** | **p-value**  **(Between Groups)** |
| --- | --- | --- | --- | --- | --- | --- |
| **Measured Distance** | Canine–Second Premolar | 7 | 1.05 | [0.43 to 1.66] | 77.66 | Raw: < 0.001  B*: < 0.022 |
|  | Canine–Lateral Incisor | 6 | 4.94 | [3.09 to 6.79] | 94.84 |  |
|  | Model-based custom references | 8 | 0.63 | [0.10 to 1.16] | 82.11 |  |
|  | C-TAD/Appliance | 2 | 0.65 | [-0.40 to 1.70] | 77.66 |  |
|  | Extraction space | 3 | 1.96 | [0.08 to 3.84] | 91.65 |  |
|  | LII Little’s Irregularity Index | 5 | 1.71 | [0.64 to 2.78] | 86.83 |  |

* B: Bonferroni‑adjusted p

# Appendix Table 9. Meta Regression for Different Covariates that Affect Relation of MOP and OTM

| **Covariate** | **K*** | **Residual I² (%)** | **R² (%)** | **Coefficient** | **CI (95%)** | **p-value** |
| --- | --- | --- | --- | --- | --- | --- |
| Duration*** (weeks) | 39 | 86.25 | 26.20 | -0.168 | [-0.228 to -0.096] | Raw: < 0.001  B**: <0.007 |
| Duration*** (weeks, if MOP was repeated) | 9 | 75.34 | 37.45 | −0.137 | [−0.250 to -0.024] | Raw: 0.018  B: 0.126 |
| Duration*** (weeks, if MOP was *not* repeated) | 30 | 88.30 | 21.83 | −0.170 | [−0.250 to -0.087] | Raw: < 0.001  B: <0.007 |
| Number of repeats***  (For baseline to last measurement) | 6 | 56.85 | 41.19 | 0.536 | [0.042 to 1.031] | Raw: 0.034  B: 0.238 |
| age (years)**** | 31 | 91.15 | 0.00 | 0.170 | [0.028 to 0.0312] | Raw: 0.019  B: 0.133 |
| diameter (mm)**** | 27 | 91.23 | 3.14 | -2.762 | [-5.185 to 0.338] | Raw: 0.026  B: 0.182 |
| distance apart (mm)**** | 11 | 90.82 | 0.00 | -0.431 | [-1.100 to 0.239] | 0.207 |
| force (g)**** | 19 | 92.04 | 5.34 | -0.037 | [-0.057 to -0.018] | Raw: < 0.001  B: <0.007 |

*K: number of Studies

**Bonferroni adjustment

*** from baseline to last measurement

**** from baseline to 4^th^ week

# Appendix Figure 30. Bubble Plot: SMD and Number of Repeats

# Appendix Figure 31. Bubble Plot: SMD and Age of Participants (years)

# Appendix Figure 32. Bubble Plot: SMD and Perforations' Distance Apart (mm)

# Appendix Figure 33. Bubble Plot: SMD and Diameters of Perforations (mm)

# Appendix Figure 34. Bubble Plot: SMD and Force Magnitutde for Orthodontic Treatment (gr)

# Appendix Table 10. Secondary Outcomes

| **Secondary Outcome** | **Studies number** | **Effect Size** | **Point Estimate** | **95 % CI** | **I² (%)** | **p-value** |
| --- | --- | --- | --- | --- | --- | --- |
| **Gingival Index** | 4 | MD | 0.03 | –0.30 to 0.37 | 67.5 | 0.78 |
|  |  | SMD | 0.06 | –0.79 to 0.91 | 58.8 | 0.83 |
| **Pocket Depth** | 3 | MD | 0.19 | –0.63 to 1.00 | 83.9 | 0.43 |
|  |  | SMD | 0.60 | –2.00 to 3.20 | 87.1 | 0.42 |
| **Immediate VAS** | 8 | MD | 0.96 | –0.08 to 1.99 | 83.1 | 0.43 |
|  |  | SMD | 0.37 | –0.35 to 1.08 | 81.7 | 0.27 |
| **After 24h VAS change** | 7 | MD | –0.13 | –0.63 to 0.36 | 15.1 | 0.53 |
|  |  | SMD | –0.15 | –0.44 to 0.14 | 0.0 | 0.25 |
| **After ddays VAS change** | 4 | MD | 0.79 | –5.28 to 6.85 | 98.4 | 0.71 |
|  |  | SMD | 0.16 | –2.34 to 2.66 | 93.5 | 0.85 |
| **Root Resorption** | 6 | MD | 0.01 | –0.04 to 0.07 | 0.0 | 0.53 |
|  |  | SMD | 0.02 | –0.03 to 0.07 | 0.0 | 0.30 |
| **Attachment Loss** | 6 | MD | –0.26 | –0.86 to 0.33 | 88.7 | 0.31 |
|  |  | SMD | –0.36 | –1.28 to 0.56 | 83.0 | 0.36 |

*Analyses use a random-effects model (Restricted Maximum Likelihood) with Knapp–Hartung standard-error adjustment. (Due to number of included studies)*

# Appendix Figure 35. Change MD of Gingival Index Forest Plot

# Appendix Figure 36. Change SMD of Gingival Index Forest Plot

# Appendix Figure 37. Change MD of Pocket Depth Forest Plot

# Appendix Figure 38. Change SMD of Pocket Depth Forest Plot

# Appendix Figure 39. Immediate Pain (VAS) SMD Forest Plot

# Appendix Figure 40. Immediate Pain (VAS) MD Forest Plot

# Appendix Figure 41. Change MD of Pain After 24hours Forest Plot

# Appendix Figure 42. Change SMD of Pain After 24hours Forest Plot

# Appendix Figure 43. Change SMD of Pain After 7Days Forest Plot

# Appendix Figure 44. Change MD of Pain After 7Days Forest Plot

# Appendix Figure 45. Change MD of Root Resorption Forest Plot

# Appendix Figure 46. Change SMD of Root Resorption Forest Plot

# Appendix Figure 47. Change SMD of Attachment Loss Forest Plot

# Appendix Figure 48. Change MD of Attachment Loss Forest Plot

# Appendix Table 11. Immediate VAS: Subgroup Analyses

| **Subgroup factor** | **Level** | **Number of studies** | **Mean [95 % CI]** | **I² (%)** | **Between groups p-value** |
| --- | --- | --- | --- | --- | --- |
| **Palatal Perforation** | Yes | 2 | 0.63 (0.19 to 1.06) | 0.0 | 0.49 |
|  | No | 6 | 1.14 (-0.39 to 2.67) | 88.1 |  |
| **Jaw** | Mandible | 3 | 2.05 (-1.44 to 5.54) | 76.9 | 0.03 |
|  | Maxilla | 5 | 0.14 (-0.59 to 0.87) | 49.0 |  |
| **Study design** | Parallel-arms | 4 | 1.65 (-1.08 to 4.37) | 88.1 | 0.23 |
|  | Split-mouth | 4 | 0.49 (0.26 to 0.72) | 0.0 |  |
| **Instrument** | Mini-screw / mini-implant | 4 | 0.57 (0.11 to 1.04) | 0.0 | 0.42 |
|  | Propel | 4 | 1.33 (-1.47 to 4.12) | 91.8 |  |

*All effects are mean differences (MD) expressed in immediate postoperative VAS units; negative values would favour the control, positive values favour the intervention. No adjustment for multiple comparisons was applied.*

# Appendix Table 12. Immediate VAS: Meta-regression

| **Covariate** | **k** | **Residual I² (%)** | **R² (%)** | **Coefficient** | **95 % CI** | **p-value** |
| --- | --- | --- | --- | --- | --- | --- |
| Female (percentage) | 7 | 86.6 | 0.0 | −0.039 | −0.157 to 0.079 | 0.516 |
| Age (years) | 8 | 67.0 | 40.9 | −0.186 | −0.369 to −0.003 | Raw: 0.046 |
|  |  |  |  |  |  | Bonferroni: 0.138 |
| Total perforations (numbers) | 8 | 35.8 | 87.7 | 0.463 | 0.249 to 0.677 | Raw: < 0.001 |
|  |  |  |  |  |  | Bonferroni: 0.0015 |

# Appendix Figure 49. Subgroup Analysis for Immediate Pain (VAS) Based on Palatal Perforation Forest Plot

# Appendix Figure 50. Subgroup Analysis for Immediate Pain (VAS) Based on Jaw(s) Forest Plot

# Appendix Figure 51. Funnel Plot

# Appendix Table 13. Egger Test

| **Statistic** | **Value** |
| --- | --- |
| Intercept (β₁) | 8.91 |
| Standard Error | 0.978 |
| z-value | 9.11 |
| p-value | < 0.0001 |

# Appendix Table 14. Trim-And-Fill analysis

| **Imputation side (trim-and-fill)** | **Observed studies (n)** | **Imputed studies (n)** | **Hedges’s g (observed)** | **95 % CI (observed)** | **Hedges’s g (observed + imputed)** | **95 % CI (observed + imputed)** |
| --- | --- | --- | --- | --- | --- | --- |
| **Left** (imputing on the left) | 39 | 0 | 1.128 | 0.994 – 1.262 | 1.128 | 0.994 – 1.262 |
| **Right** (imputing on the right) | 39 | 0 | 1.128 | 0.994 – 1.262 | 1.128 | 0.994 – 1.262 |

# Appendix Table 15. Leave-one-out analysis

| **Omitted Study** | **Hedges's g** | **95% CI (Lower)** | **95% CI (Upper)** | **p-value** |
| --- | --- | --- | --- | --- |
| Aboalga et al., 2019 | 1.697 | 1.223 | 2.171 | 0.000 |
| Ahsan et al., 2021 | 1.626 | 1.155 | 2.098 | 0.000 |
| Al-Attar et al., 2022 | 1.585 | 1.124 | 2.046 | 0.000 |
| Alfailany et al., 2023 | 1.588 | 1.127 | 2.050 | 0.000 |
| Al-Hayek et al., 2019 | 1.675 | 1.201 | 2.149 | 0.000 |
| Alikhani et al., 2013 | 1.521 | 1.075 | 1.966 | 0.000 |
| Alkebsi et al., 2018 | 1.706 | 1.238 | 2.174 | 0.000 |
| Alqadasi et al., 2019 | 1.701 | 1.232 | 2.170 | 0.000 |
| Alqadasi et al., 2024 | 1.703 | 1.234 | 2.172 | 0.000 |
| Attri et al., 2018 | 1.649 | 1.171 | 2.127 | 0.000 |
| Babanouri et al., 2020 | 1.634 | 1.161 | 2.107 | 0.000 |
| Bansal et al., 2019 | 1.593 | 1.130 | 2.056 | 0.000 |
| El Awady et al., 2019 | 1.679 | 1.204 | 2.154 | 0.000 |
| Fattori et al., 2020 | 1.699 | 1.229 | 2.169 | 0.000 |
| Feizbakhsh et al., 2018 | 1.656 | 1.180 | 2.133 | 0.000 |
| Golshah et al., 2021 | 1.697 | 1.220 | 2.174 | 0.000 |
| GOWDA S V et al., 2019 | 1.632 | 1.160 | 2.105 | 0.000 |
| Gulduren et al., 2020 | 1.700 | 1.230 | 2.170 | 0.000 |
| Kilinc and Baka, 2023 | 1.689 | 1.214 | 2.163 | 0.000 |
| Kumar et al., 2024 | 1.561 | 1.104 | 2.018 | 0.000 |
| Kundi et al., 2018 | 1.402 | 1.002 | 1.803 | 0.000 |
| Li et al., 2022 | 1.699 | 1.227 | 2.172 | 0.000 |
| Martina et al., 2022 | 1.673 | 1.195 | 2.152 | 0.000 |
| Mordente et al., 2024 | 1.700 | 1.228 | 2.173 | 0.000 |
| Ozkan et al., 2021 | 1.659 | 1.185 | 2.133 | 0.000 |
| RAGHAV et al., 2020 | 1.691 | 1.210 | 2.172 | 0.000 |
| Rashid et al., 2018 | 1.661 | 1.188 | 2.135 | 0.000 |
| Sahin et al., 2023 | 1.672 | 1.196 | 2.148 | 0.000 |
| Singh et al., 2023 | 1.559 | 1.106 | 2.012 | 0.000 |
| Thomas et al., 2021 | 1.675 | 1.192 | 2.158 | 0.000 |
| Venkatachalapathy et al., 2022 | 1.605 | 1.140 | 2.070 | 0.000 |

# Appendix Figure 52. Leave-one-out Analysis Forest Plot

# Appendix Table 16. Cumulative meta-analysis

| **Study** | **Hedges's g** | **95% CI (Lower)** | **95% CI (Upper)** | **p-value** | **Year** |
| --- | --- | --- | --- | --- | --- |
| Alikhani et al., 2013 | 9.030 | 6.109 | 11.952 | 0.000 | 2013 |
| Alkebsi et al., 2018 | 4.364 | -4.547 | 13.274 | 0.337 | 2018 |
| Attri et al., 2018 | 2.788 | 0.476 | 5.101 | 0.018 | 2018 |
| Feizbakhsh et al., 2018 | 2.223 | 0.631 | 3.815 | 0.006 | 2018 |
| Kundi et al., 2018 | 4.448 | 2.205 | 6.691 | 0.000 | 2018 |
| Rashid et al., 2018 | 3.787 | 1.936 | 5.638 | 0.000 | 2018 |
| Aboalga et al., 2019 | 3.129 | 1.604 | 4.654 | 0.000 | 2019 |
| Al-Hayek et al., 2019 | 2.760 | 1.430 | 4.091 | 0.000 | 2019 |
| Alqadasi et al., 2019 | 2.394 | 1.189 | 3.599 | 0.000 | 2019 |
| Bansal et al., 2019 | 2.472 | 1.322 | 3.622 | 0.000 | 2019 |
| El Awady et al., 2019 | 2.266 | 1.228 | 3.304 | 0.000 | 2019 |
| GOWDA S V et al., 2019 | 2.218 | 1.265 | 3.171 | 0.000 | 2019 |
| Babanouri et al., 2020 | 2.173 | 1.296 | 3.050 | 0.000 | 2020 |
| Fattori et al., 2020 | 2.004 | 1.174 | 2.834 | 0.000 | 2020 |
| Gulduren et al., 2020 | 1.856 | 1.070 | 2.643 | 0.000 | 2020 |
| RAGHAV et al., 2020 | 1.736 | 1.021 | 2.451 | 0.000 | 2020 |
| Ahsan et al., 2021 | 1.756 | 1.074 | 2.437 | 0.000 | 2021 |
| Golshah et al., 2021 | 1.652 | 1.016 | 2.289 | 0.000 | 2021 |
| Ozkan et al., 2021 | 1.624 | 1.018 | 2.231 | 0.000 | 2021 |
| Thomas et al., 2021 | 1.574 | 1.010 | 2.138 | 0.000 | 2021 |
| Al-Attar et al., 2022 | 1.664 | 1.101 | 2.227 | 0.000 | 2022 |
| Li et al., 2022 | 1.585 | 1.044 | 2.127 | 0.000 | 2022 |
| Martina et al., 2022 | 1.548 | 1.034 | 2.063 | 0.000 | 2022 |
| Venkatachalapathy et al., 2022 | 1.601 | 1.093 | 2.109 | 0.000 | 2022 |
| Alfailany et al., 2023 | 1.669 | 1.162 | 2.176 | 0.000 | 2023 |
| Kilinc and Baka, 2023 | 1.616 | 1.128 | 2.105 | 0.000 | 2023 |
| Sahin et al., 2023 | 1.586 | 1.116 | 2.057 | 0.000 | 2023 |
| Singh et al., 2023 | 1.679 | 1.200 | 2.157 | 0.000 | 2023 |
| Alqadasi et al., 2024 | 1.616 | 1.147 | 2.086 | 0.000 | 2024 |
| Kumar et al., 2024 | 1.700 | 1.228 | 2.173 | 0.000 | 2024 |
| Mordente et al., 2024 | 1.644 | 1.183 | 2.104 | 0.000 | 2024 |

# Appendix Figure 53. Cumulative Meta-Analysis Forest Plot

# Appendix Figure 54. Calculated Vs. Available Change Forest Plot

# Appendix Figure 55. Calculated Vs. Available Average (1) Forest Plot

# Appendix Figure 56. Calculated Vs. Available Average (2) Forest Plot
